# Supplementary material for: PTPN1/PTPN2 inhibition improves NK cancer therapy by enhancing IL-2 and mitigating TGFβ1 responses
Source: EMBO Rep. 2026 Apr 15;27(10):2581–613. doi: 10.1038/s44319-026-00745-0 (PMC13219468; doi:10.1038/s44319-026-00745-0)
Supplement: Supplementary file 1 — Appendix [file 44319_2026_745_MOESM1_ESM.pdf]

Appendix for PTPN1/PTPN2 inhibition improves NK cancer therapy by enhancing IL-2 and mitigating TGFβ1 responses

TABLE OF CONTENTS

Appendix Figure S1. HLA expressions on K562 and Reh tumor target cells 2

Appendix Figure S2. Dual inhibition of PTPN1 and PTPN2 enhances p-AKT but not p-p38 MAPK signaling in NK-92 cells following IL-2 or IL-15 stimulation 3

Appendix Figure S3. Response to cytokines IL-4 or TGFβ1 combined treatment with dual-inhibition of PTPN1/PTPN2 in NK-92 cells 5

Appendix Figure S4. Flow cytometry gating strategies of immunophenotype and cytolytic granules analysis in NK-92 and CB NK cells 7

Appendix Figure S5. Flow cytometry gating strategies of CB NK cell subsets, purity and NK-92 cells degranulation 9

Appendix Figure S6. Body weights of NSG mice from healthy or tumor-bearing background in response to NK cell treatment with or without dual-inhibition of PTPN1/PTPN2 10

APPENDIX FIGURES

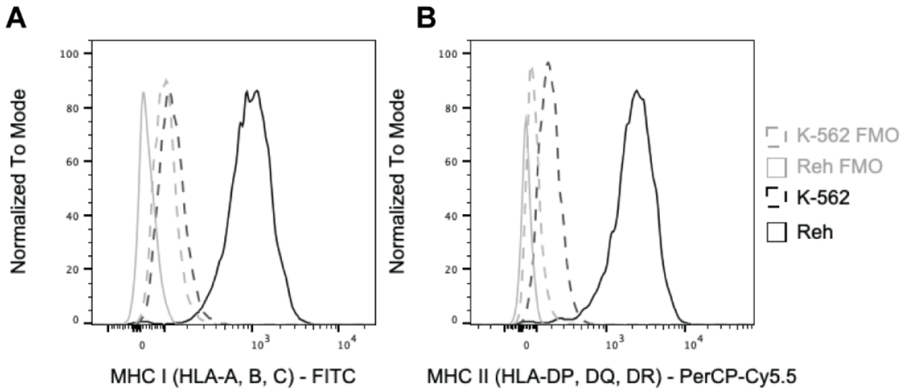

Appendix Figure S1. HLA expressions on K562 and Reh tumor target cells

(A)- (B) Flow cytometry analysis of MHC class I (HLA-A, -B, -C) and MHC class II (HLA-DP, -DQ, -DR) expression on tumor target K-562 and Reh cells, as shown in panels (A) and (B), respectively. Expression levels were quantified by mean fluorescence intensity (MFI). Representative data from n = two biological replicates; each tested in three technical replicates, is shown.

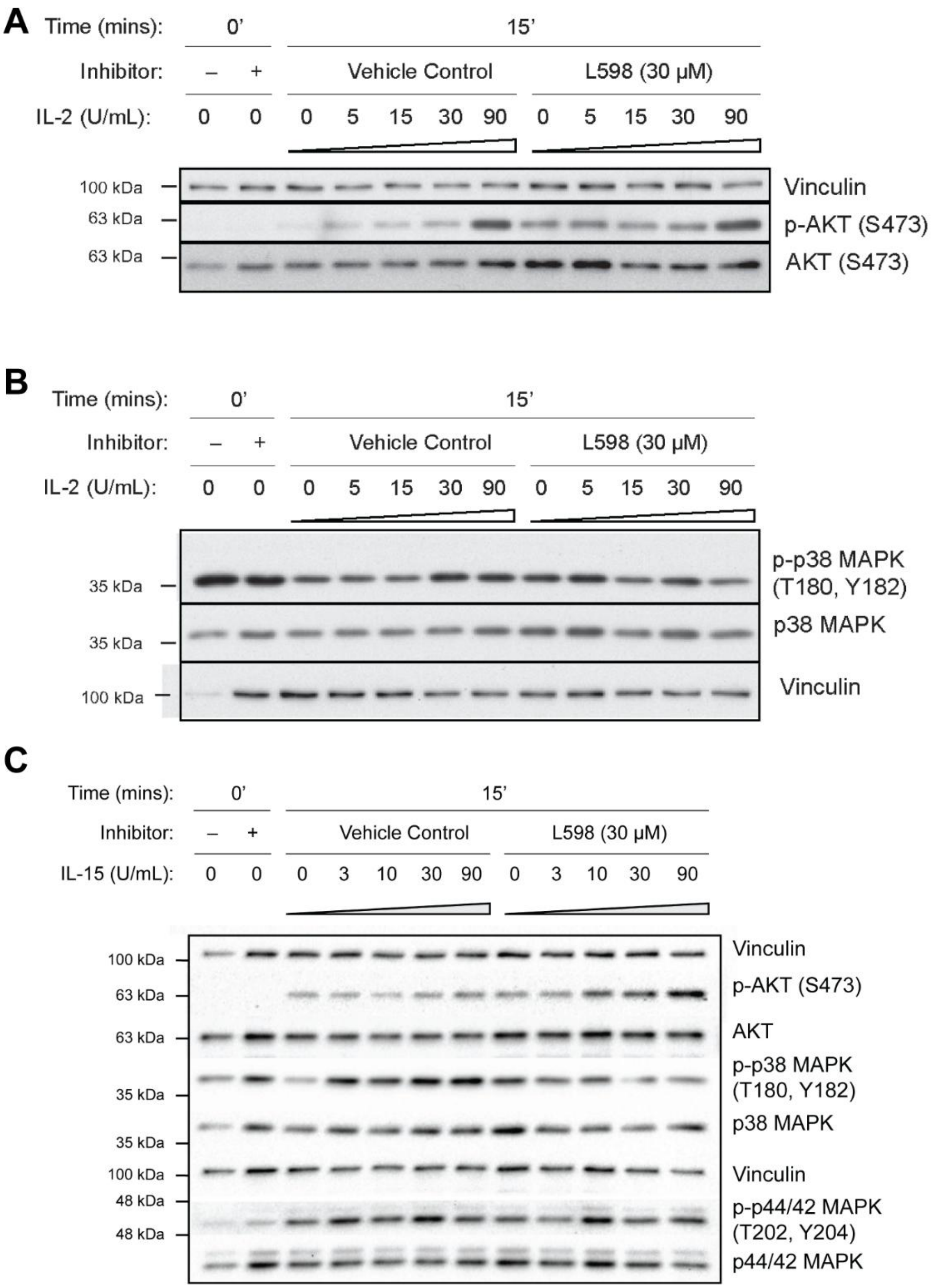

Appendix Figure S2. Dual inhibition of PTPN1 and PTPN2 enhances p-AKT but not p-p38 MAPK signaling in NK-92 cells following IL-2 or IL-15 stimulation

(A) – (B) Western blot analysis of AKT and MAPK pathways in L598 (30 μM) or vehicle control pre-treated NK-92 cells in response to IL-2 stimulation at an increasing concentration (0- 90IU/mL) for 15 mins. NK-92 cells were serum-starved for six hours in the presence of L598 or vehicle control before IL-2 stimulation. A representative figure from n = two biological replicates is shown.

The same membrane was probed for (p)-STAT5 (>75 kDa) (Figure 4B) and (p)-AKT (~63 kDa) (Appendix Figure S2A); therefore, the same vinculin protein loading control is shown in (A). Likewise, the same membrane was probed for (p)-STAT4 (>75 kDa) (Figure 4B) and (p)-p38 MAPK (~35 kDa) (Appendix Figure S2B); therefore, the same vinculin protein loading control is shown in (B). **(C)** Western blot analysis of AKT and MAPK pathway activation in NK-92 cells stimulated with increasing doses of IL-15 (0–90 IU/mL) for 15 minutes. Cells were pre-treated with dual inhibitor L598 (30 μM) or vehicle control. A representative figure of n = two biological replicates is shown.

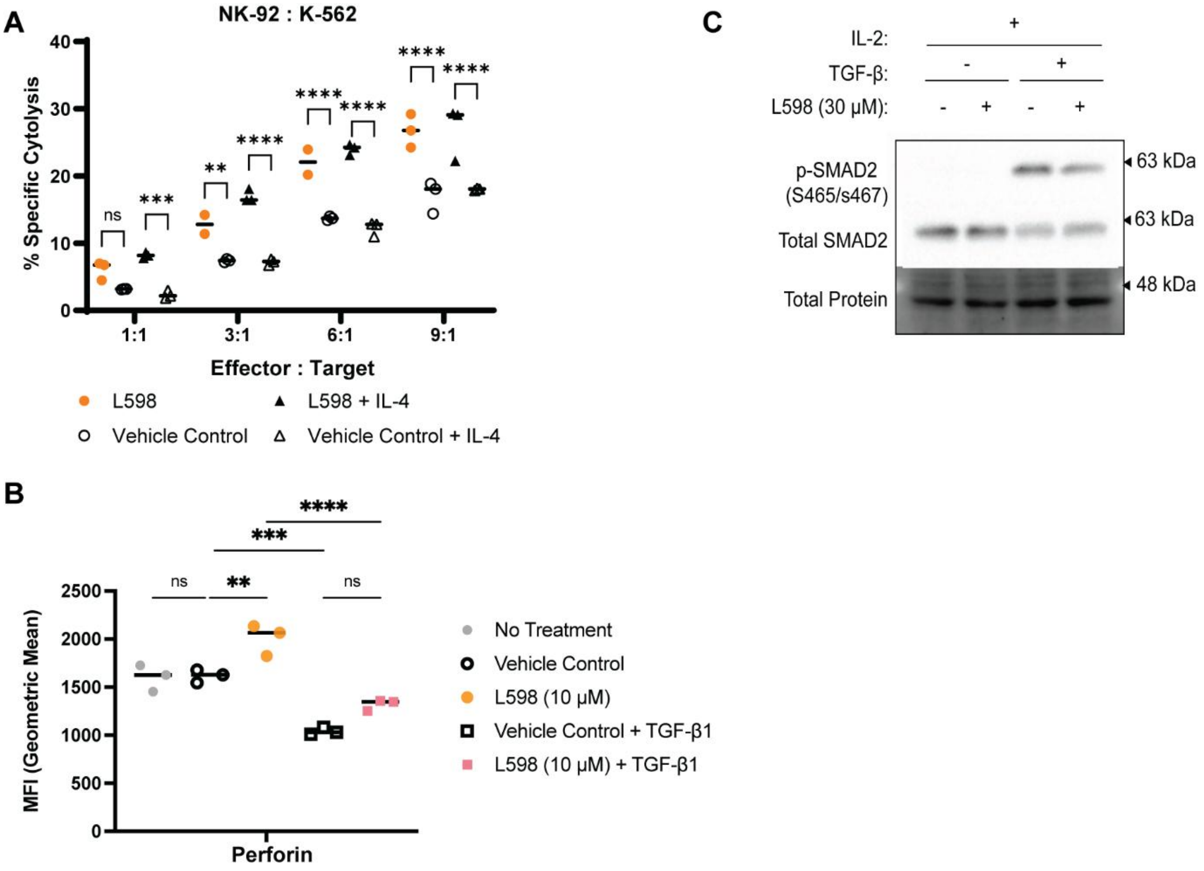

Appendix Figure S3. Response to cytokines IL-4 or TGFβ1 combined treatment with dual-inhibition of PTPN1/PTPN2 in NK-92 cells

(A) Anti-tumor cytolysis assay against K-562 across various E: T ratios by NK-92 cells treated with IL-4 (10 ng/mL) along with L598 (10 μM) or vehicle control. Data information: Data from n = two biological replicates, each tested in three technical replicates, is shown as a representative scatter plot with the median indicated. P-values (E: T = 1: 1): p = 0.1434 (ns), p = 0.0006 (***); P-values (E: T = 3: 1): p = 0.0051 (**), p ≤ 0.0001 (****); P-values (E: T = 6: 1): p ≤ 0.0001 (****); P-values (E: T = 9: 1): p ≤ 0.0001 (****). (Two-way ANOVA with Tukey's multiple comparison test). **(B)** Flow cytometry analysis of intracellular cytolytic granules perforin in NK-92 cells treated with L598 (10μM) or vehicle control along with TGFβ1 (10ng/mL). Data information: Data from n = two biological replicates, each tested in three technical replicates, is shown as a representative scatter plot with the median indicated. P-values: p = 0.9997 (ns) for no treatment vs vehicle control, p = 0.0546 (ns) for vehicle control + TGFβ1 vs L598 + TGFβ1, p = 0.0071 (**) for vehicle control vs L598, p = 0.0004 (***) for vehicle control vs vehicle control + TGFβ1, p ≤ 0.0001 (****) for L598 vs L598 + TGFβ1. (One-way ANOVA with Tukey' multiple

comparison test). **(C)** TGFβ1 (10ng/mL) treatment activated phosphorylation of SMAD2 (S465/467), however, there is no significant difference between L598 (30 μM) and vehicle control treatment groups. A representative figure of n = three biological replicates is shown.

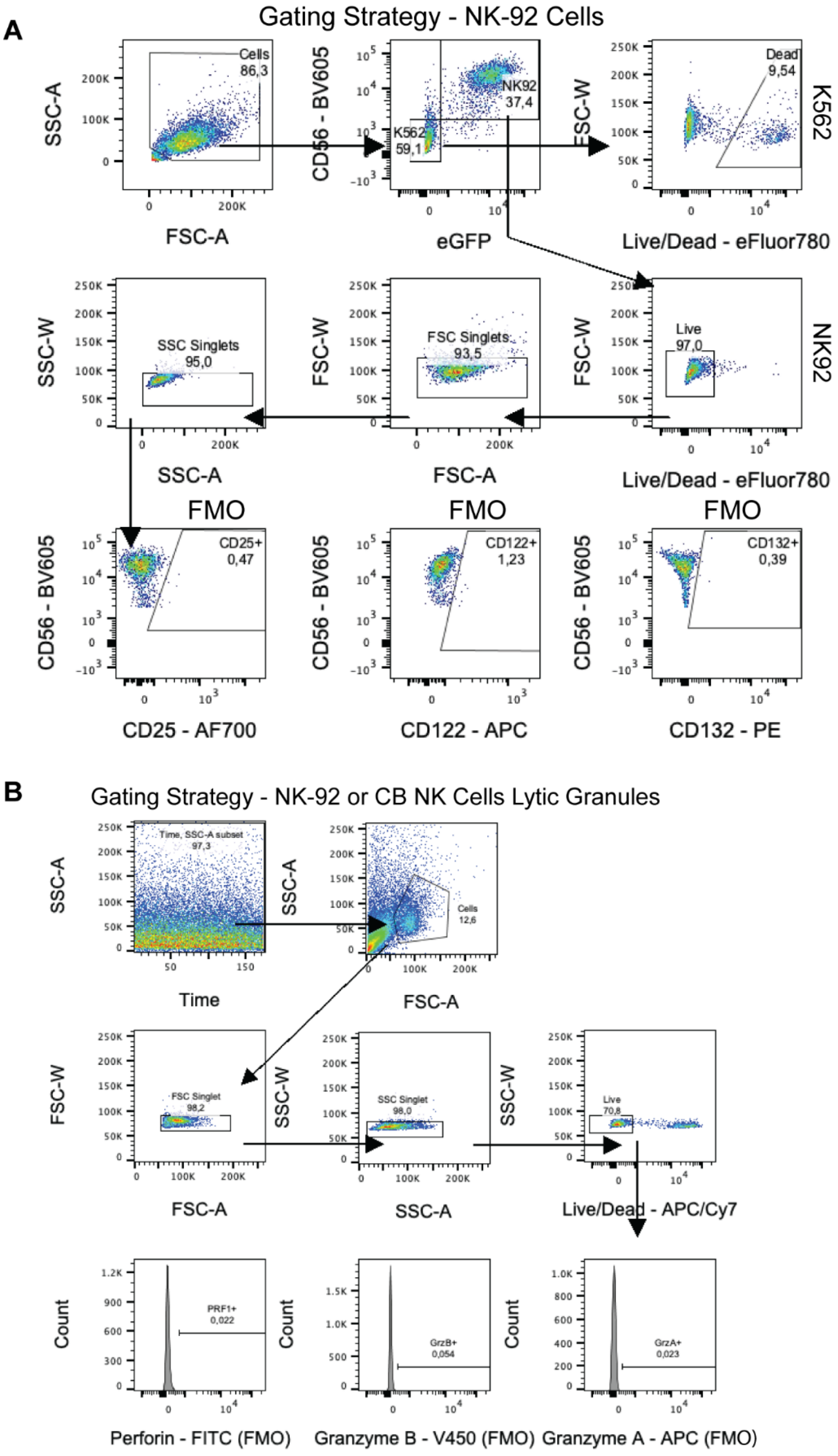

Appendix Figure S4. Flow cytometry gating strategies of immunophenotype and cytolytic granules analysis in NK-92 and CB NK cells

(A) an example for analyzing surface expressions of CD25 and CD122 on live and single shRNA modified NK-92 cells (CD56^{+}, GFP^{+}) with tumor target K-562 cells (CD56^{-}, GFP^{-}) at E: T = 1: 1 stimulations. FMO controls were used for gating. **(B)** Gating strategy for intracellular cytolytic granules analysis in live and single NK-92 or CB NK cells (gated as CD45^{+}, CD34^{-}, DUMP^{-} cells as shown in Appendix Figure S5 A).

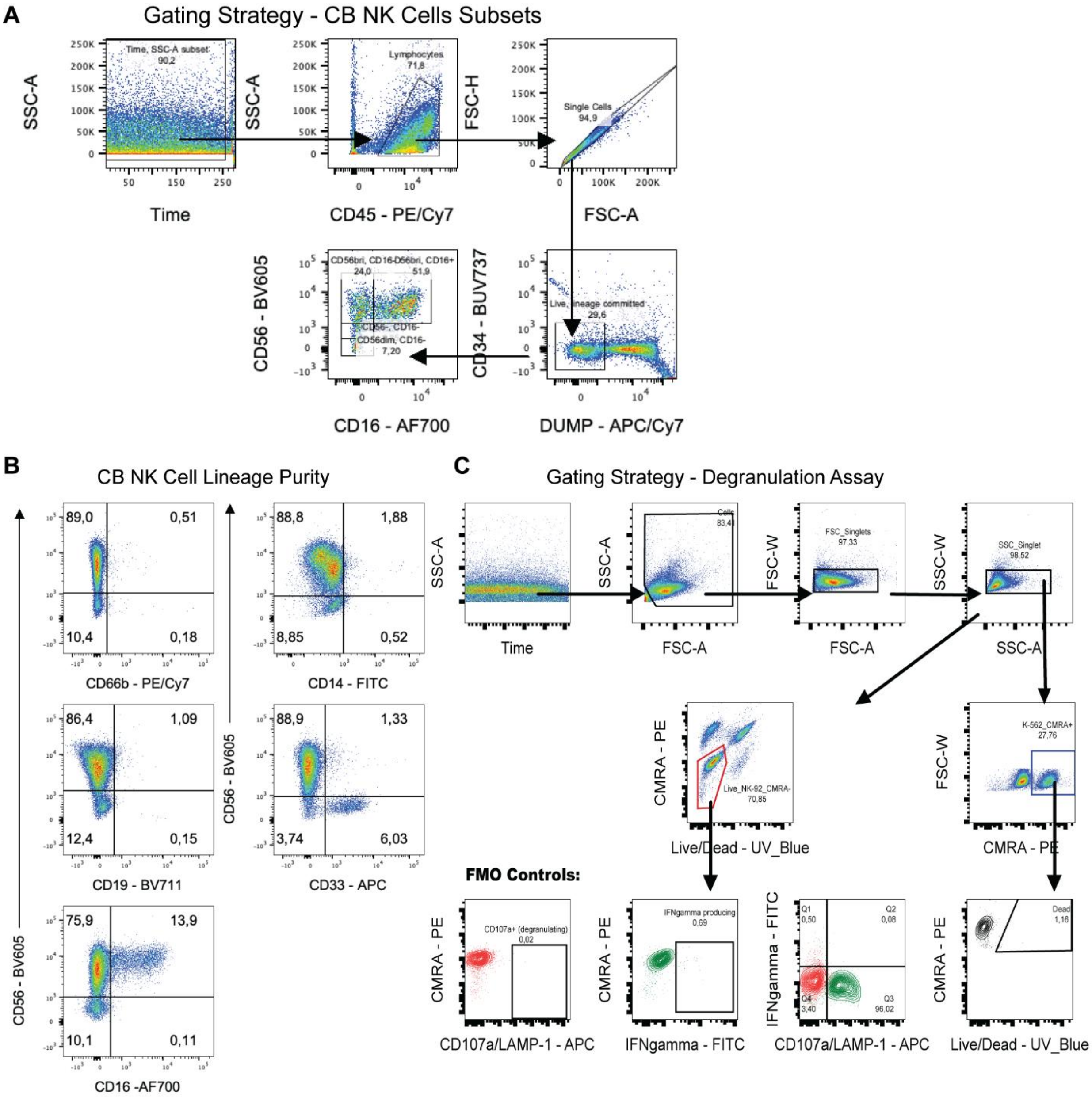

Appendix Figure S5. Flow cytometry gating strategies of CB NK cell subsets, purity and NK-92 cells degranulation

(A) an example of analyzing CB NK cells subset gating that was applied for surface receptor CD69 and CD25 analysis. **(B)** purity of CB NK cells was measured with cell lineage markers: CD66b, CD14, CD19, and CD33 after *ex vivo* expansion. **(C)** an example of analyzing degranulating (CD107a^{+}) and IFN-γ producing NK-92 cells with tumor K562 cell (CMRA^{+}) stimulations.

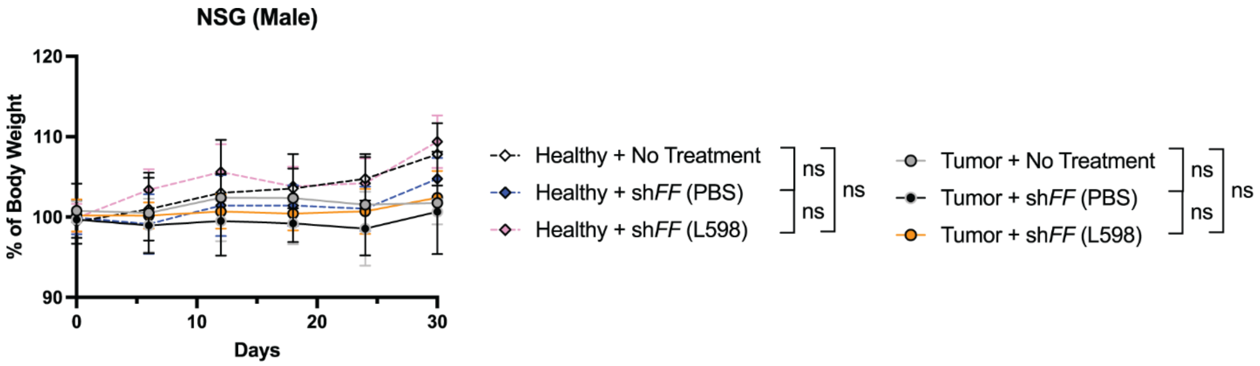

Appendix Figure S6. Body weights of NSG mice from healthy or tumor-bearing background in response to NK cell treatment with or without dual-inhibition of PTPN1/PTPN2

Changes in body weight in healthy or U-87 transplanted mice that received sh*FF* + L598 treatment (n = 5 biological replicates), sh*FF* + vehicle control treatment(n = 7 biological replicates) or no treatment (n = 8 biological replicates) over 34 days. Data information: Body weight of tumor groups was adjusted after subtracting tumor weight. P-values: $p > 0.1234$ (ns) (Tukey's multiple comparisons test).
