# Supplementary figures and images for: PTPN1/PTPN2 inhibition improves NK cancer therapy by enhancing IL-2 and mitigating TGFβ1 responses (part 1 of 3)
Source: EMBO Rep. 2026 Apr 15;27(10):2581–613. doi: 10.1038/s44319-026-00745-0 (PMC13219468; doi:10.1038/s44319-026-00745-0)

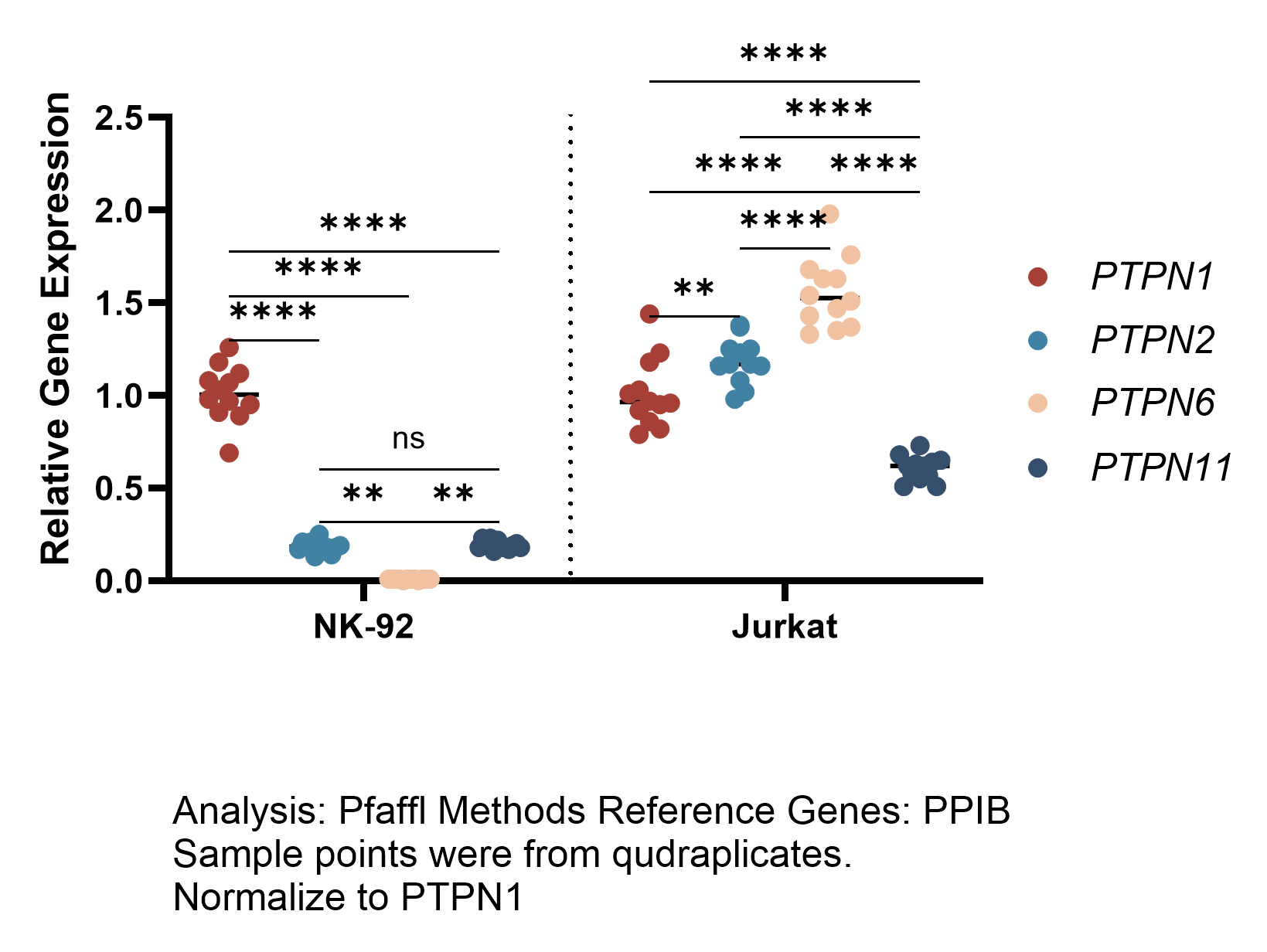

Supplement: Supplementary file 3 — Source data Fig. 1 [file 44319_2026_745_MOESM3_ESM.zip › Figure 1/1A/1A.tif]

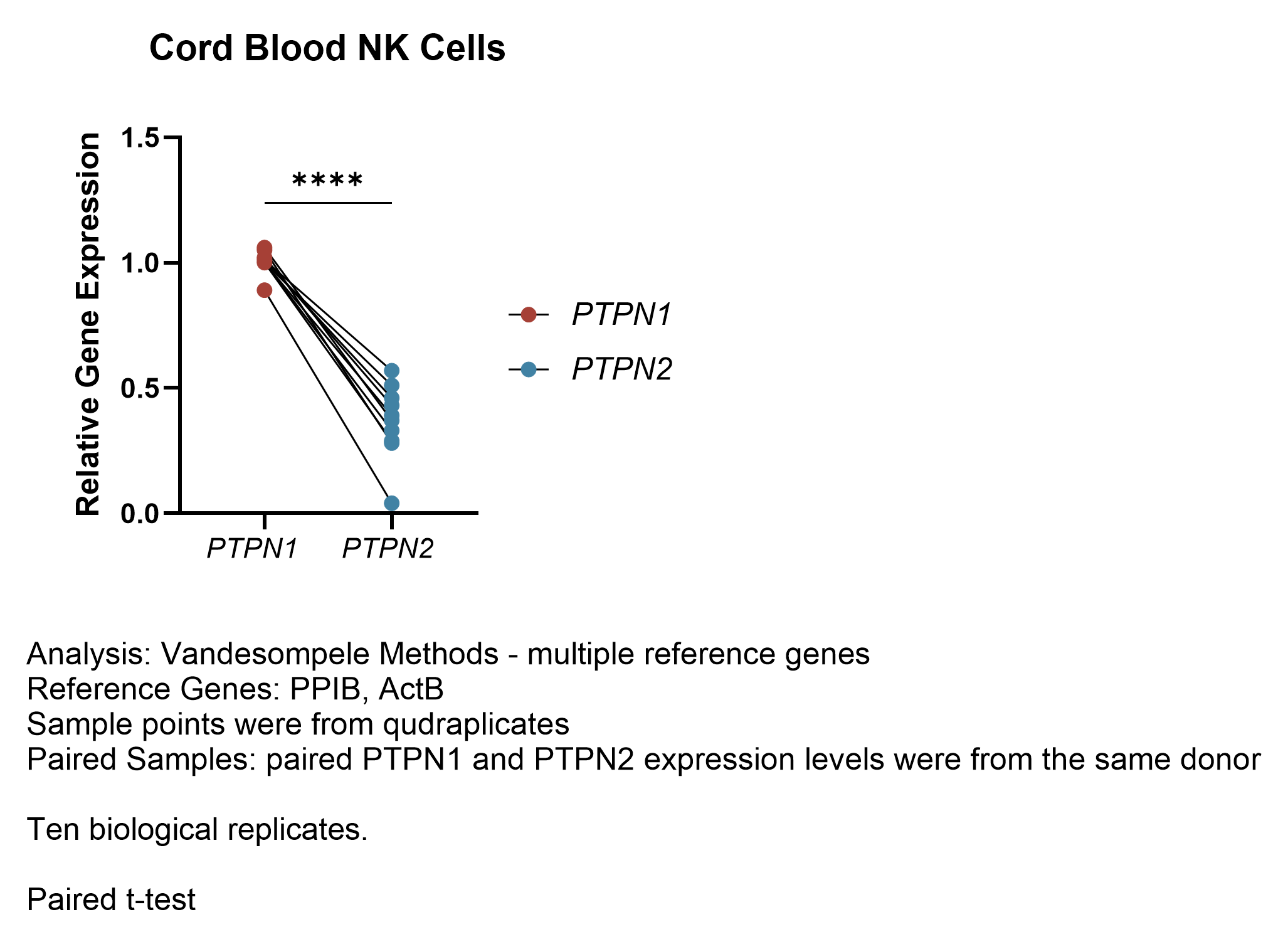

Supplement: Supplementary file 3 — Source data Fig. 1 [file 44319_2026_745_MOESM3_ESM.zip › Figure 1/1B/1B.tif]

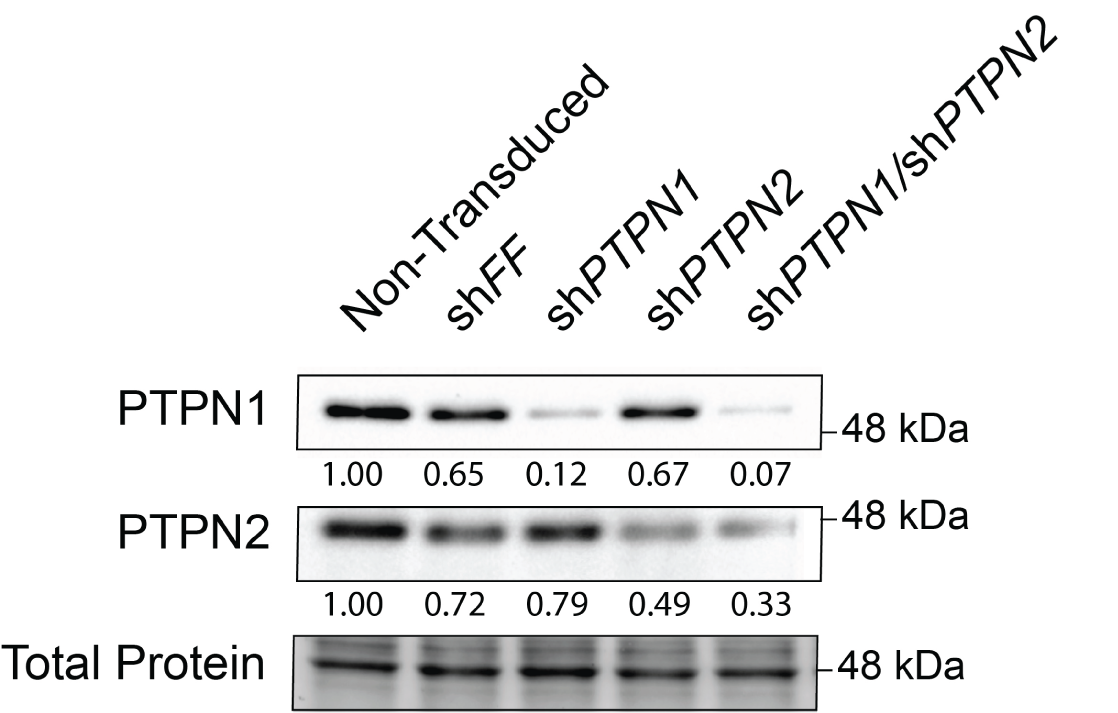

Supplement: Supplementary file 3 — Source data Fig. 1 [file 44319_2026_745_MOESM3_ESM.zip › Figure 1/1C/1C.tif]

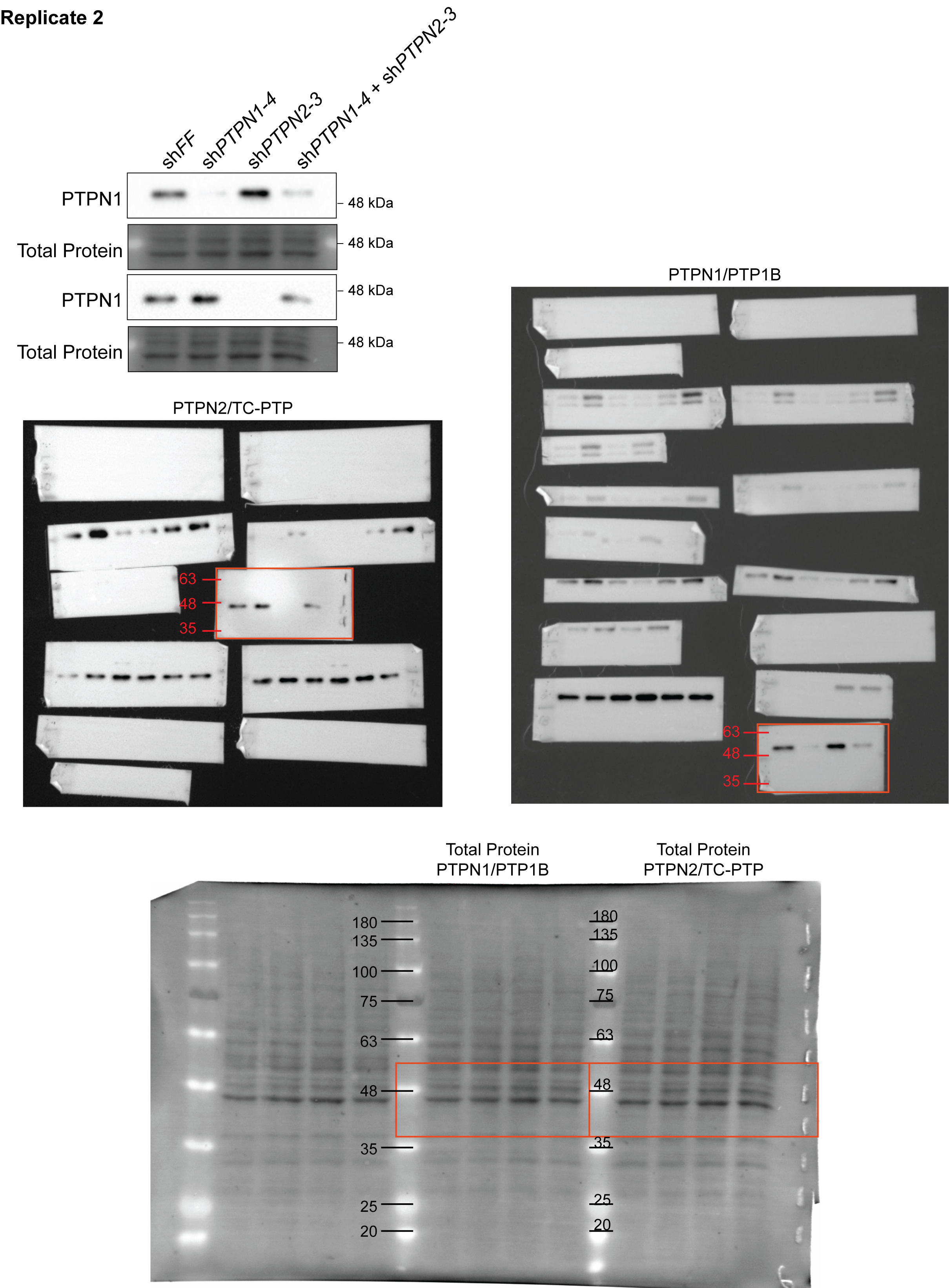

Supplement: Supplementary file 3 — Source data Fig. 1 [file 44319_2026_745_MOESM3_ESM.zip › Figure 1/1C/Replicates/1C_Exp3.tif]

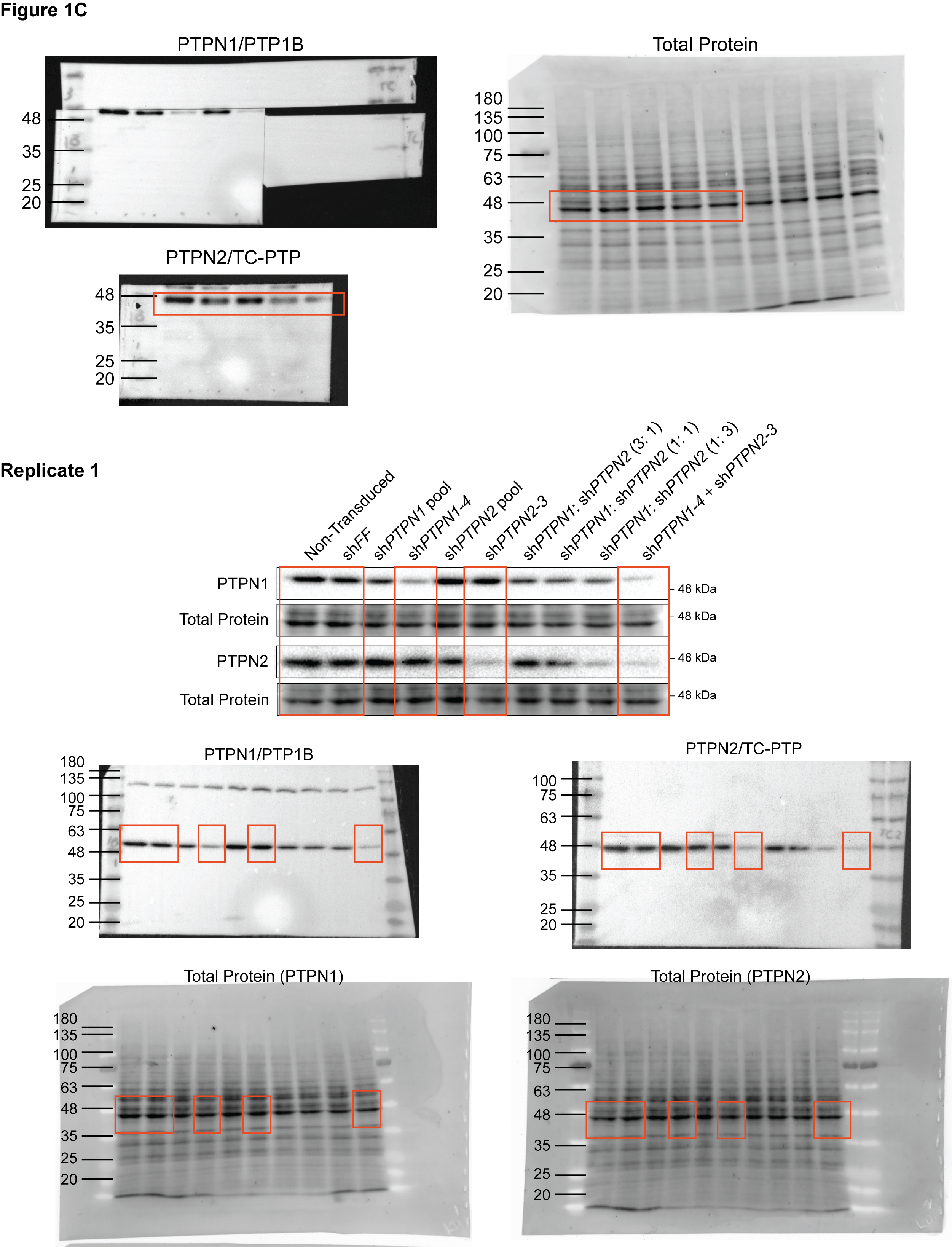

Supplement: Supplementary file 3 — Source data Fig. 1 [file 44319_2026_745_MOESM3_ESM.zip › Figure 1/1C/Replicates/1C_EXP1+EXP2.tif]

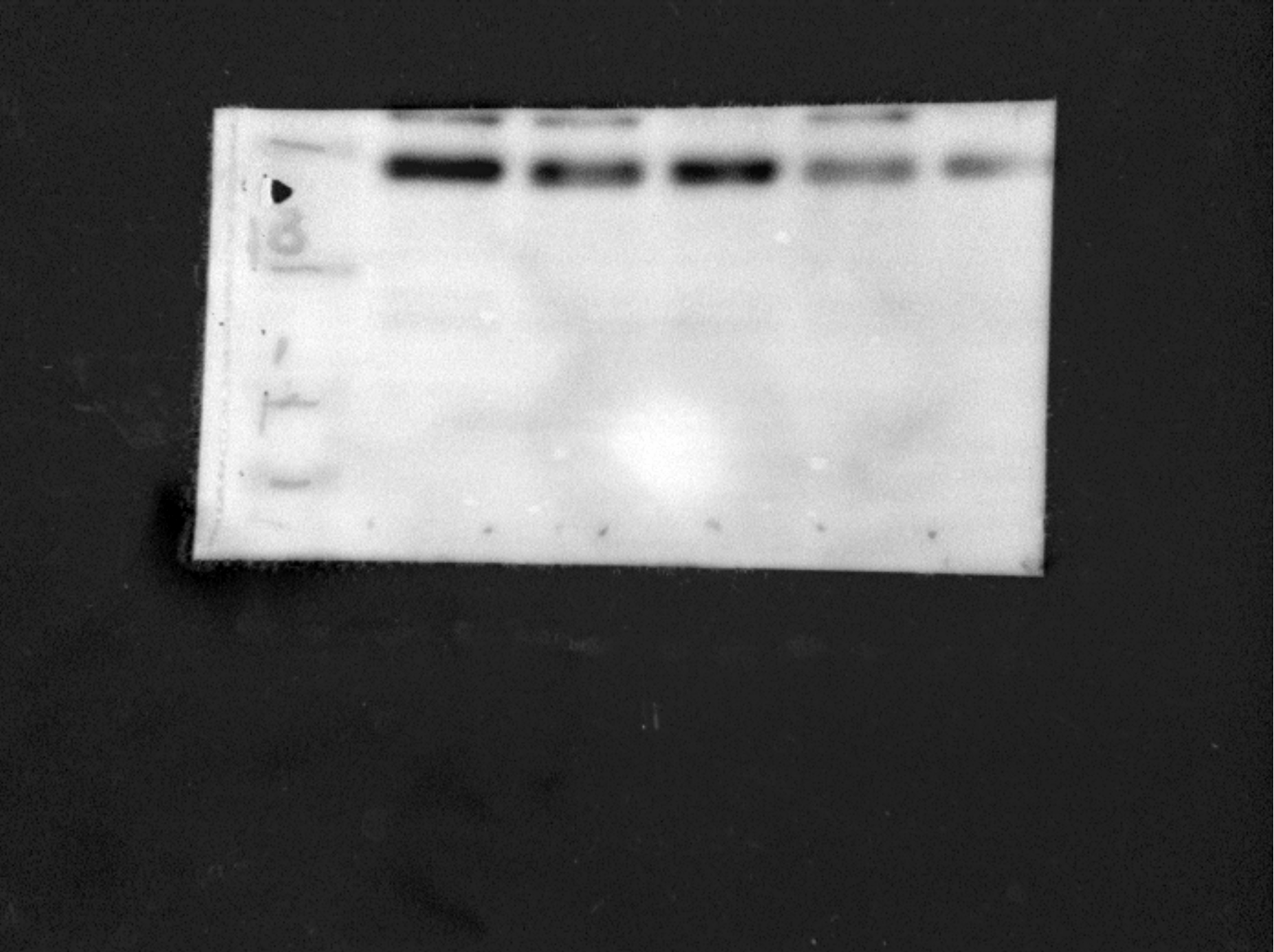

Supplement: Supplementary file 3 — Source data Fig. 1 [file 44319_2026_745_MOESM3_ESM.zip › Figure 1/1C/Replicates/Raw Data/1C_EXP1_PTPN2 Verification_9.3sec+colori.tif]

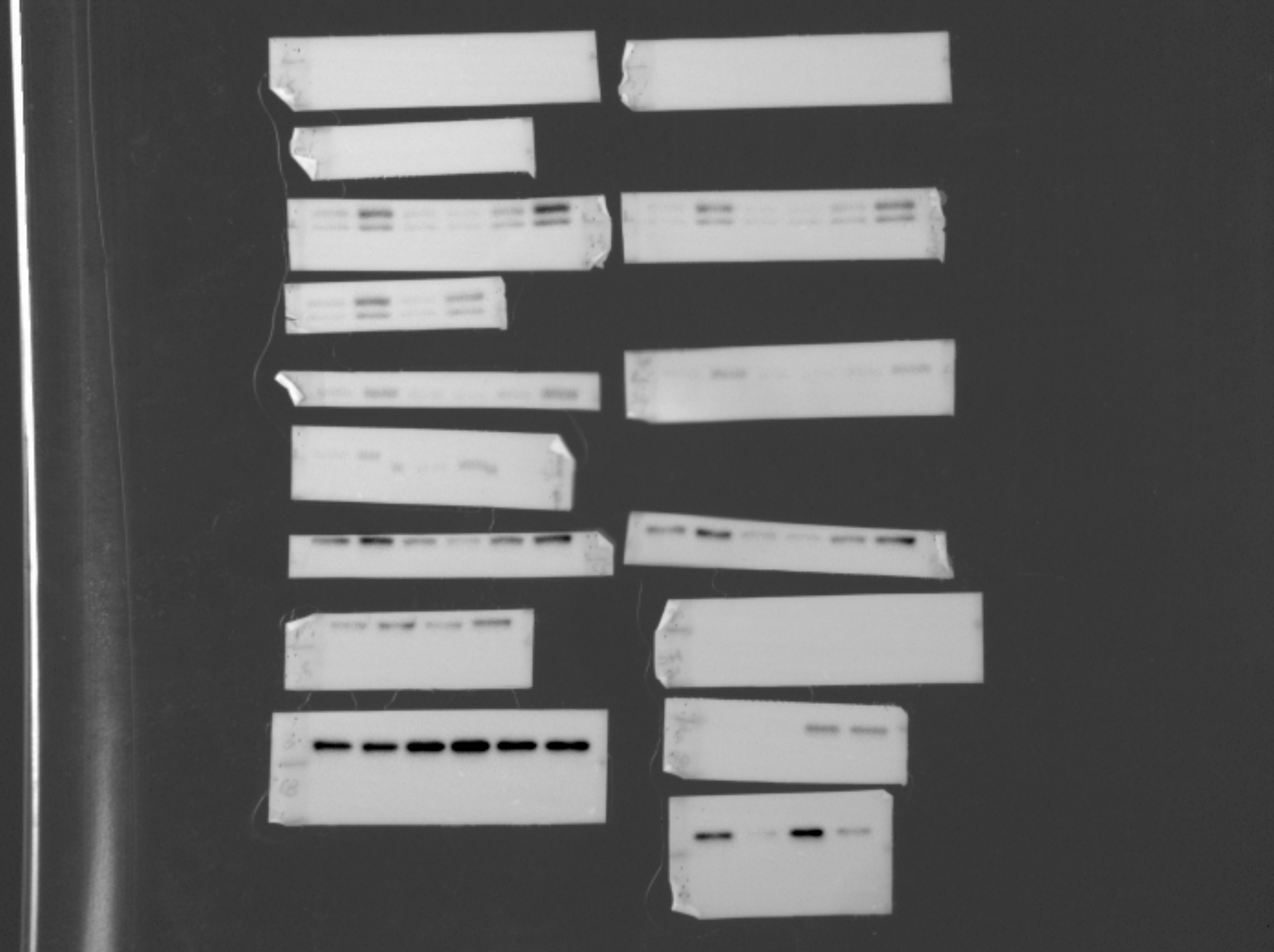

Supplement: Supplementary file 3 — Source data Fig. 1 [file 44319_2026_745_MOESM3_ESM.zip › Figure 1/1C/Replicates/Raw Data/1C_EXP3_PTPN1 Verification_colori+13.1sec.tif]

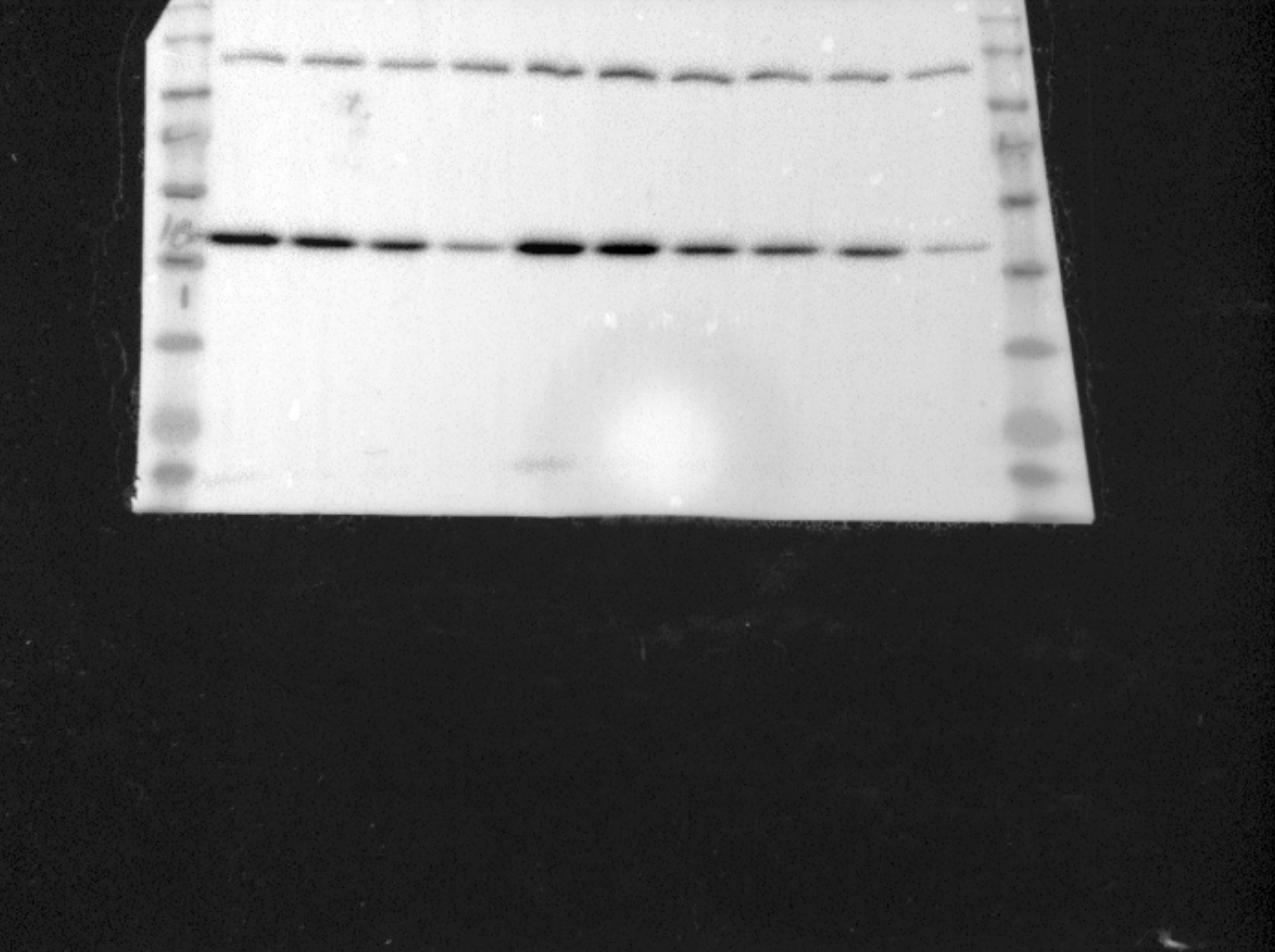

Supplement: Supplementary file 3 — Source data Fig. 1 [file 44319_2026_745_MOESM3_ESM.zip › Figure 1/1C/Replicates/Raw Data/1C_EXP2_PTPN1_Verification_19.4sec+colori.tif]

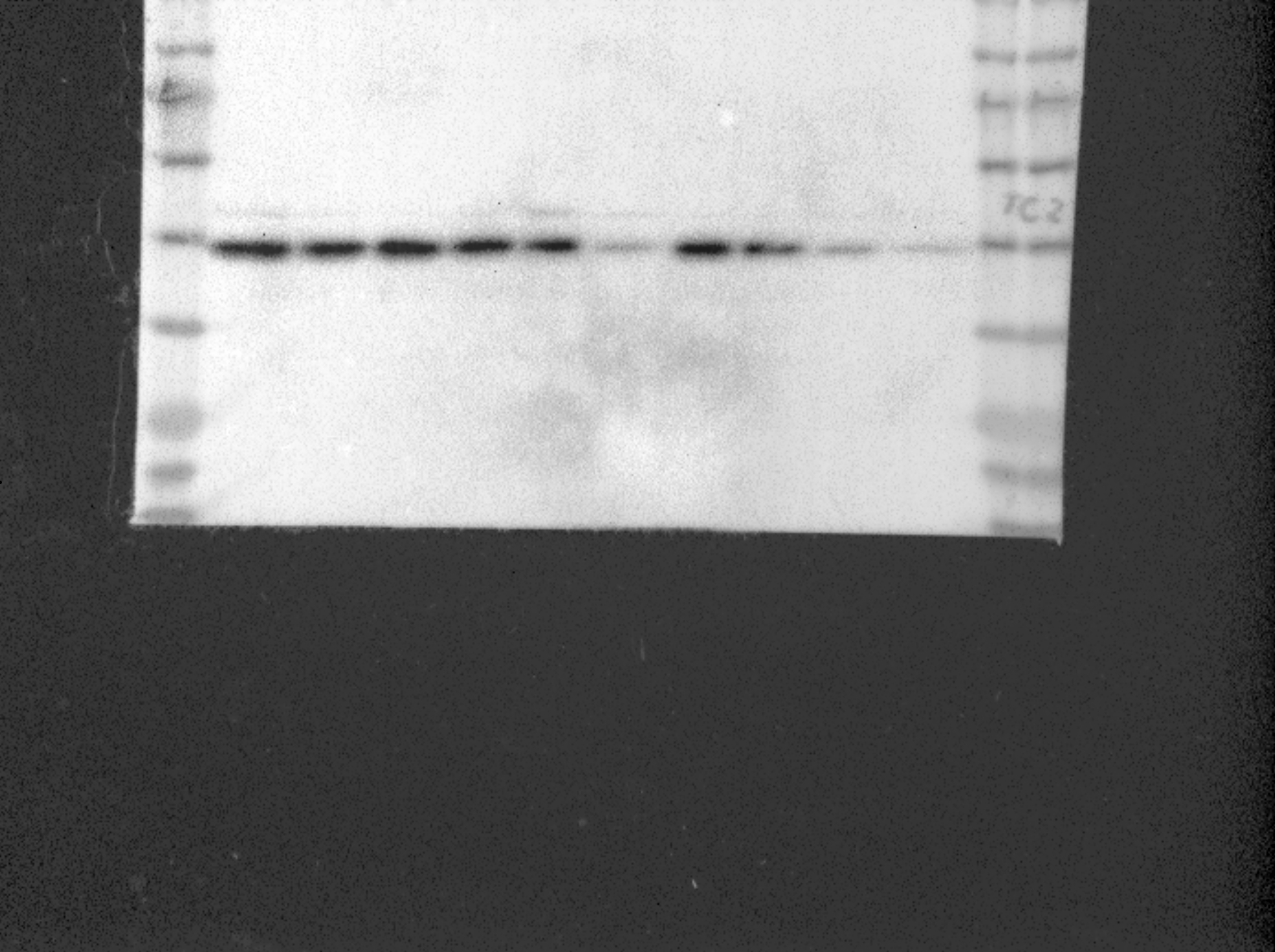

Supplement: Supplementary file 3 — Source data Fig. 1 [file 44319_2026_745_MOESM3_ESM.zip › Figure 1/1C/Replicates/Raw Data/1C_EXP2_PTPN2 Verification 301.5sec+colori.tif]

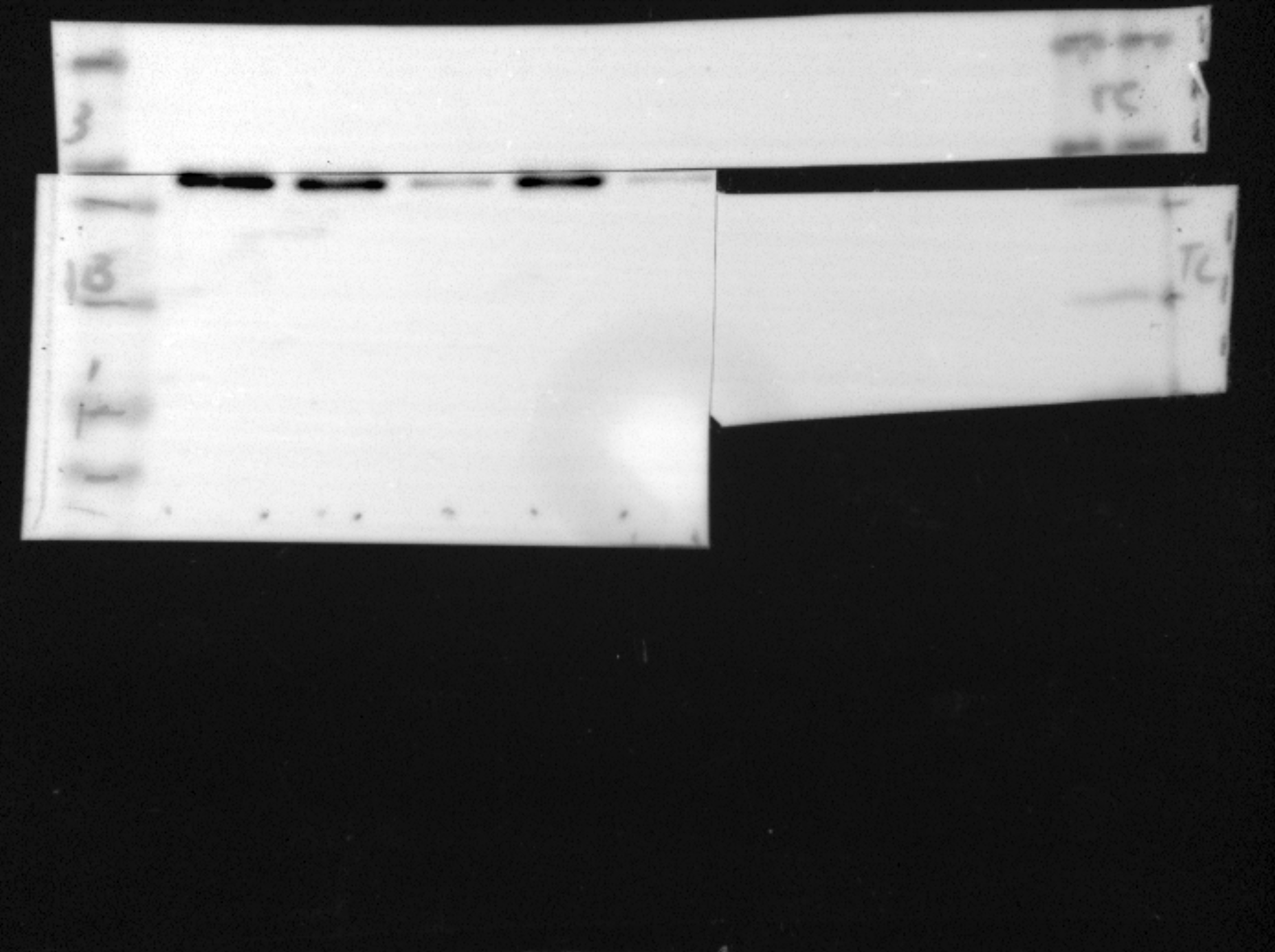

Supplement: Supplementary file 3 — Source data Fig. 1 [file 44319_2026_745_MOESM3_ESM.zip › Figure 1/1C/Replicates/Raw Data/1C_EXP1_PTPN1_43.3sec+colori.tif]

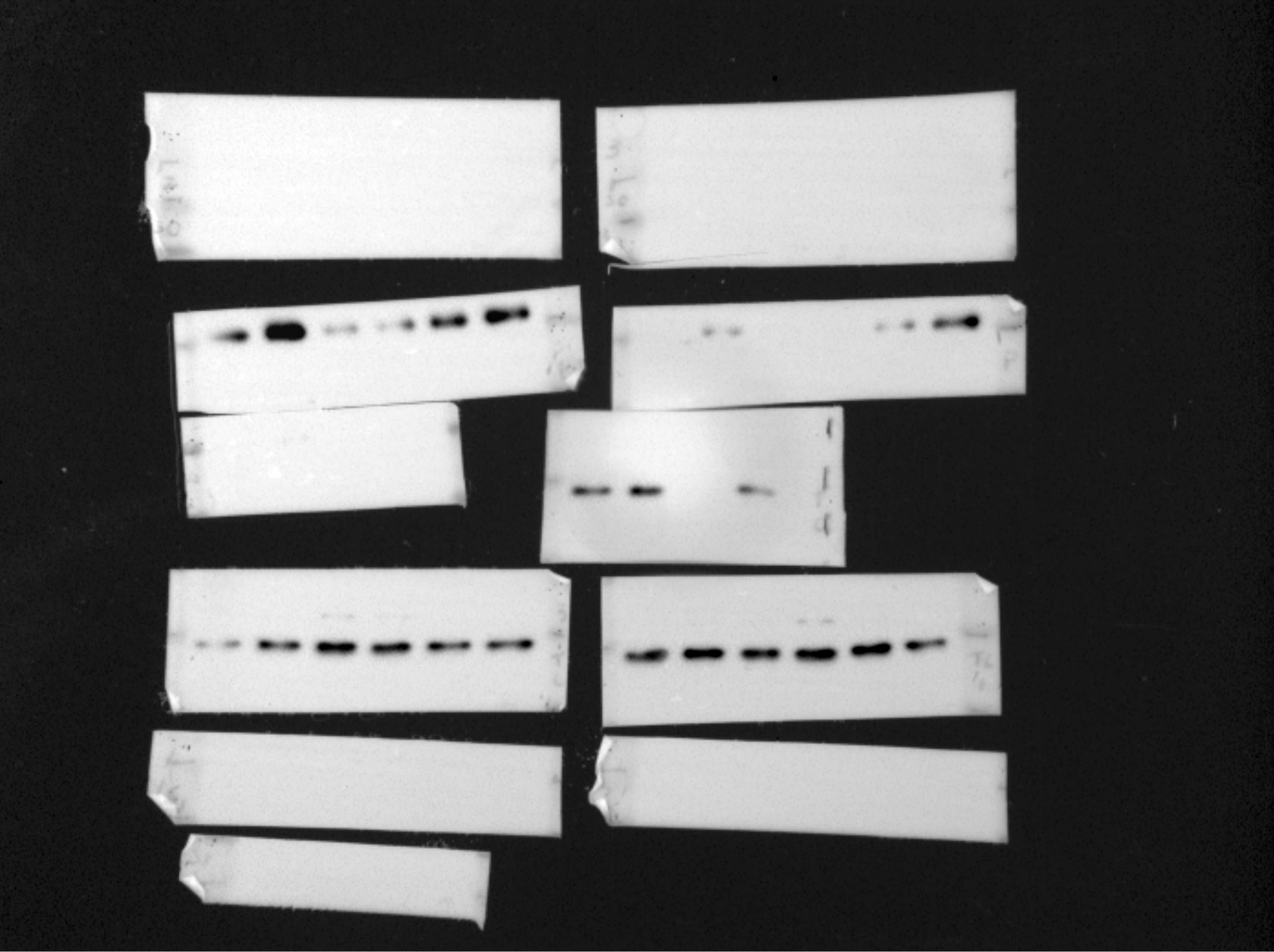

Supplement: Supplementary file 3 — Source data Fig. 1 [file 44319_2026_745_MOESM3_ESM.zip › Figure 1/1C/Replicates/Raw Data/1C_EXP3_PTPN2 Verification _376.4sec+COLORI.tif]

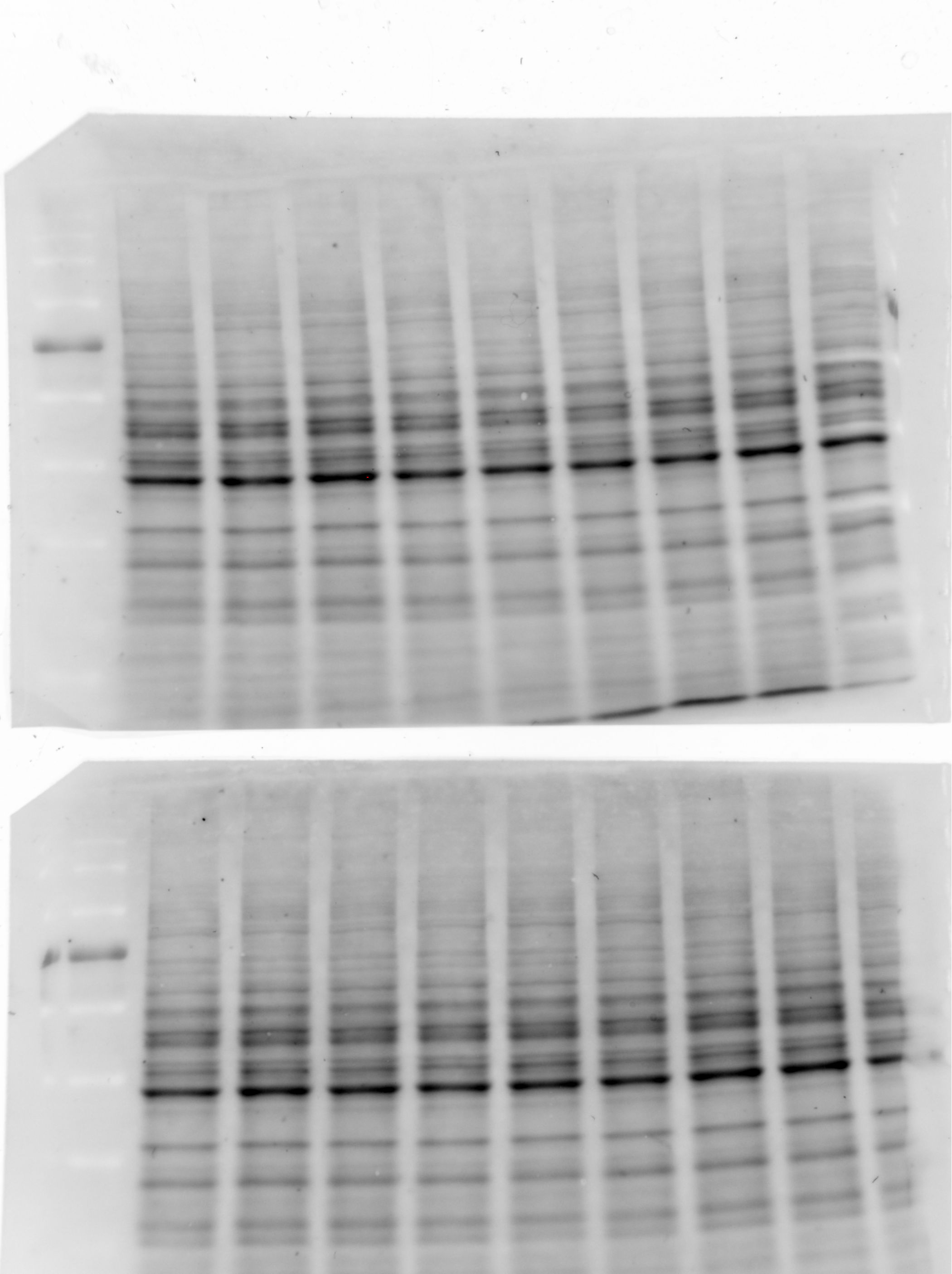

Supplement: Supplementary file 3 — Source data Fig. 1 [file 44319_2026_745_MOESM3_ESM.zip › Figure 1/1C/Replicates/Raw Data/1C_EXP1_Loading Control_Total Protein_Top Gel.tif]

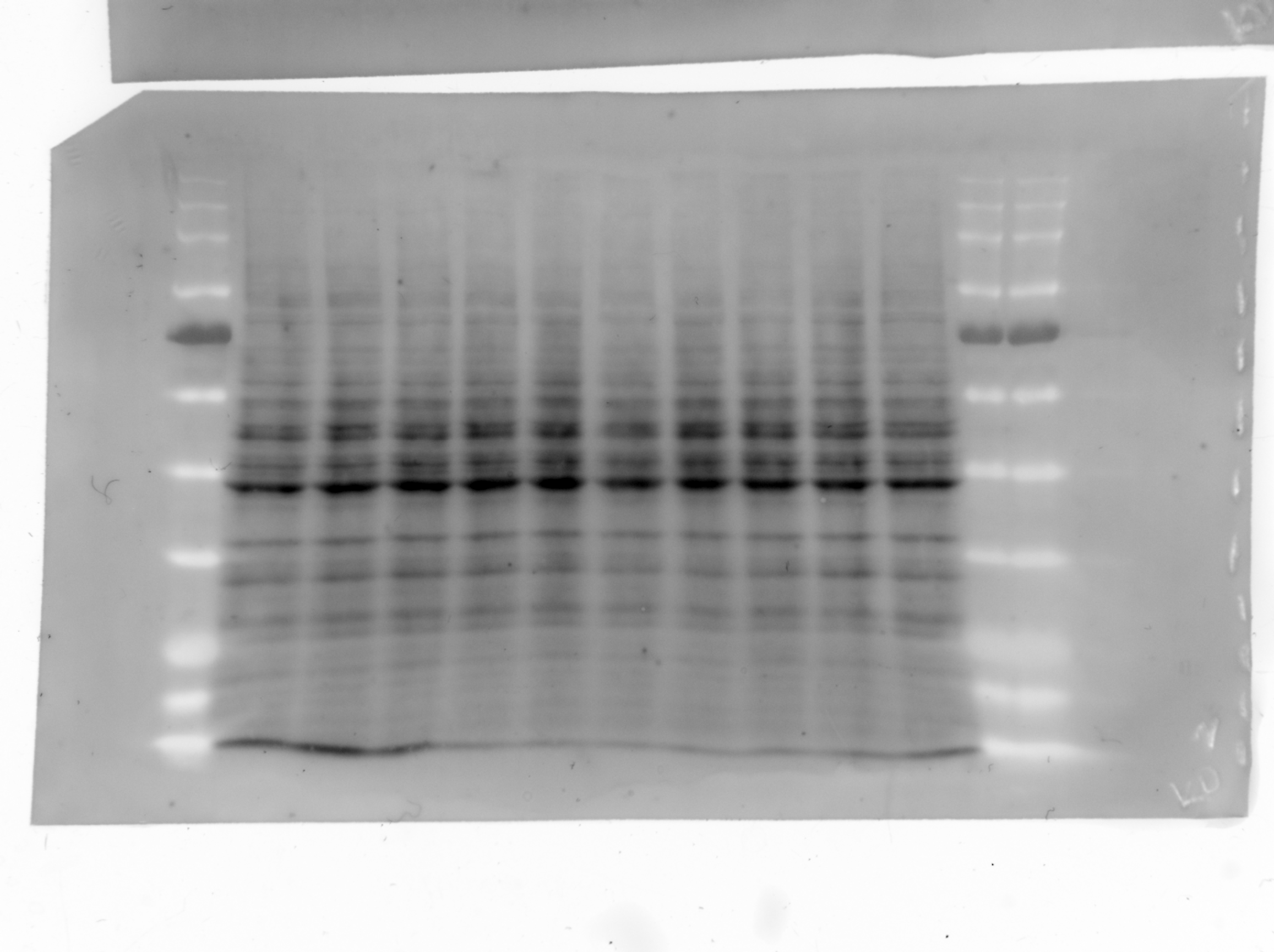

Supplement: Supplementary file 3 — Source data Fig. 1 [file 44319_2026_745_MOESM3_ESM.zip › Figure 1/1C/Replicates/Raw Data/1C_EXP2_Loading Control_PTPN2_Total Protein.tif]

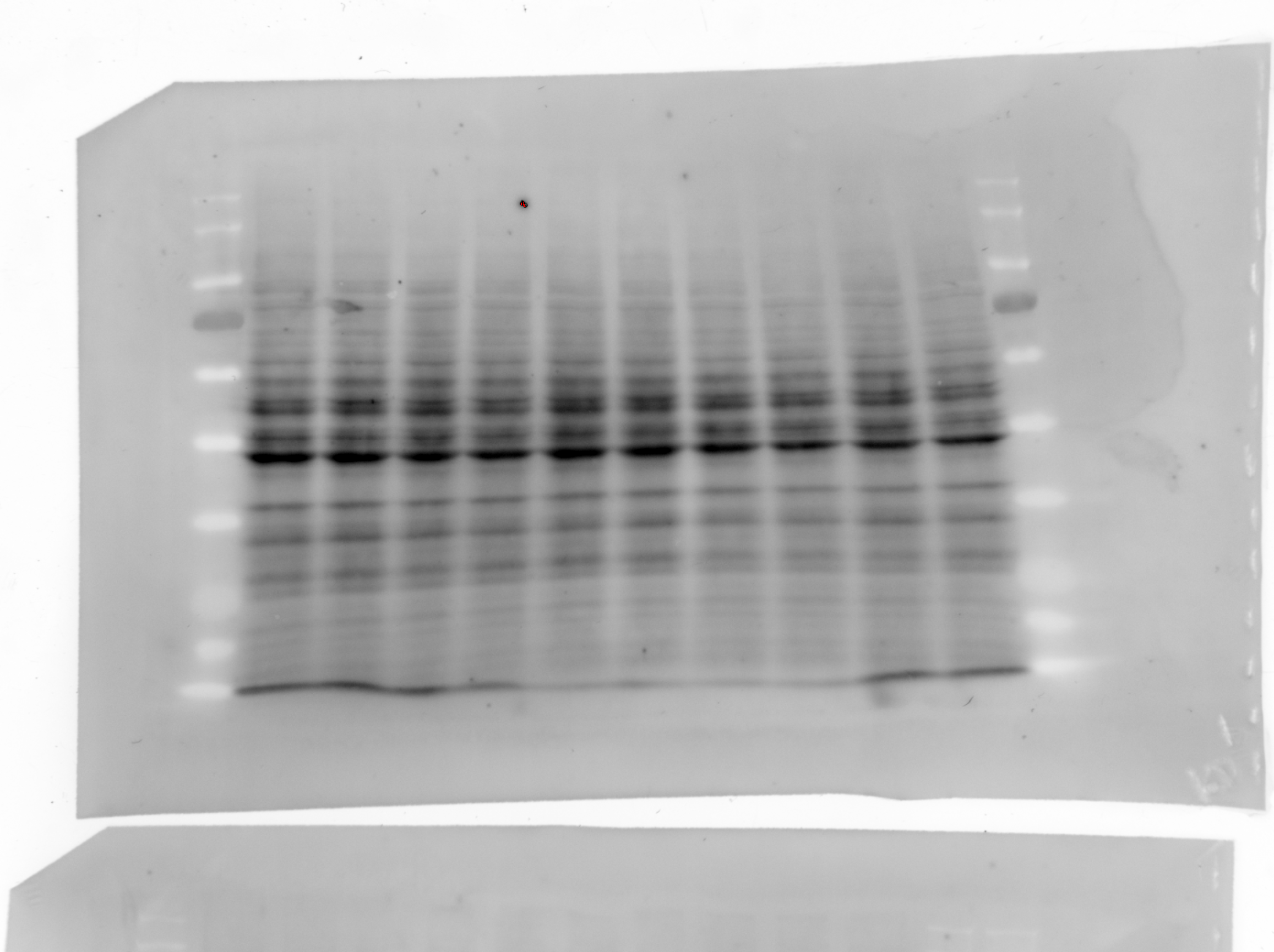

Supplement: Supplementary file 3 — Source data Fig. 1 [file 44319_2026_745_MOESM3_ESM.zip › Figure 1/1C/Replicates/Raw Data/1C_EXP2_Loading Control_PTPN1_Total Protein.tif]

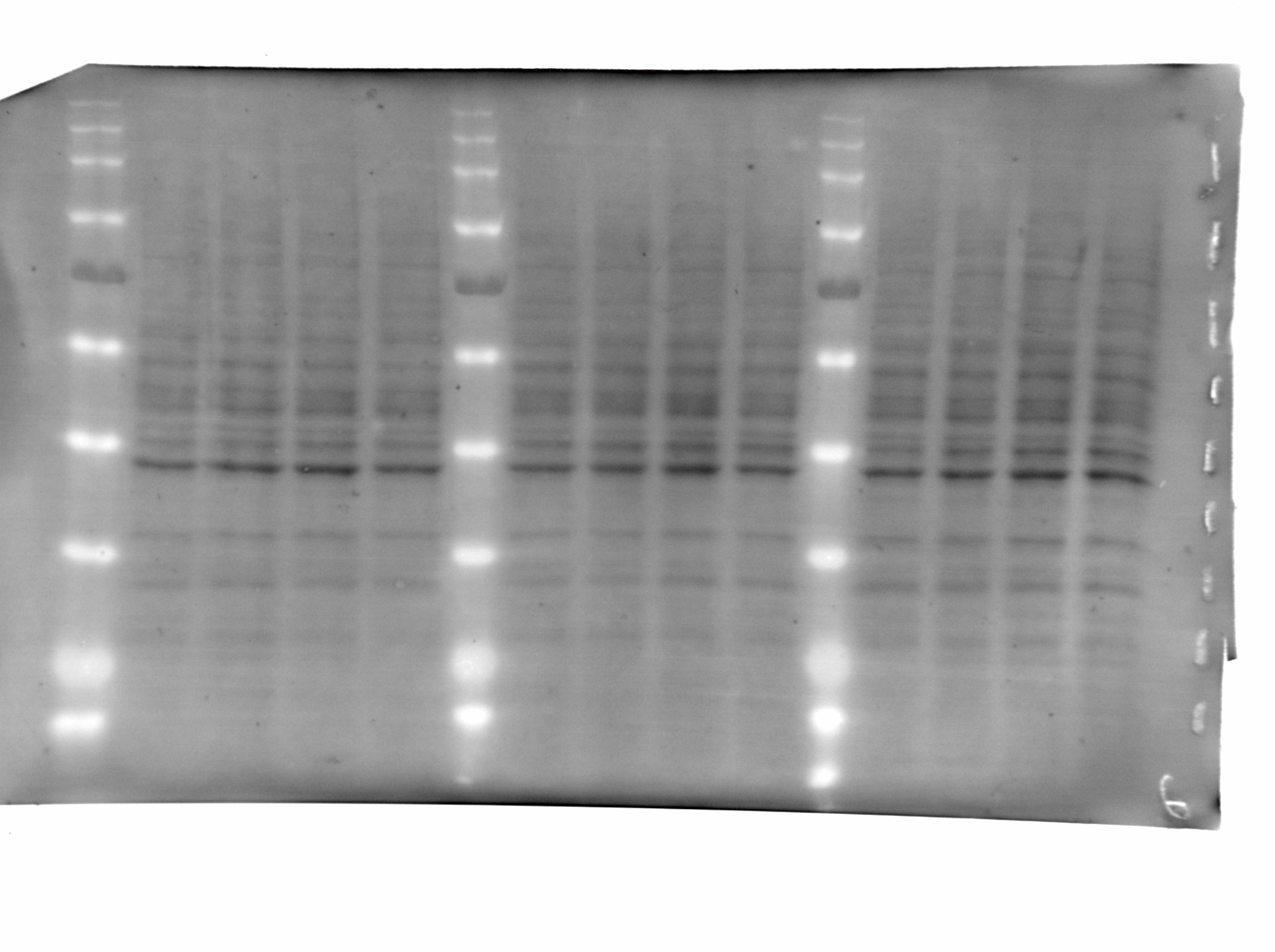

Supplement: Supplementary file 3 — Source data Fig. 1 [file 44319_2026_745_MOESM3_ESM.zip › Figure 1/1C/Replicates/Raw Data/1C_EXP3_Loading Control_Total Protein_PTPN1 Middle GEL_PTPN2 Right.tif]

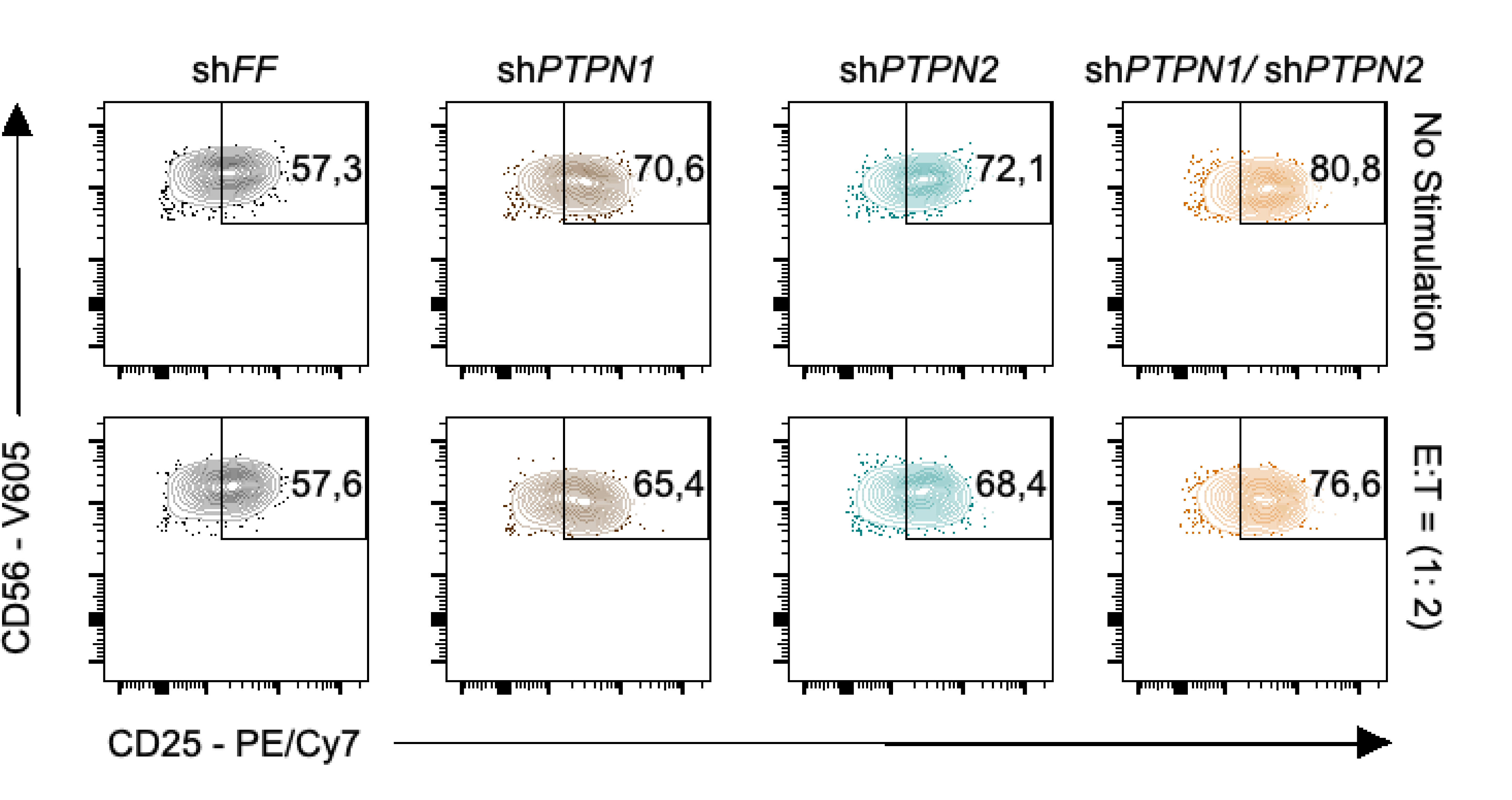

Supplement: Supplementary file 3 — Source data Fig. 1 [file 44319_2026_745_MOESM3_ESM.zip › Figure 1/1D/1D_Exp.1_CD25_20220529.tif]

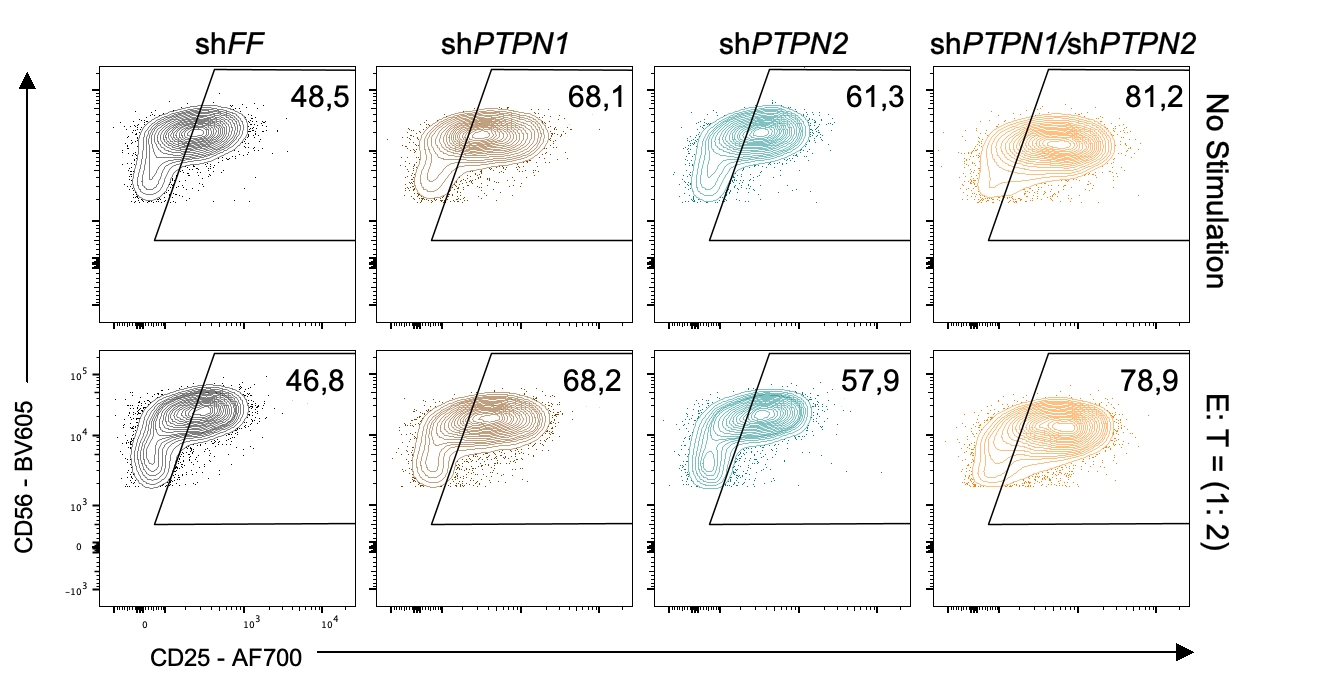

Supplement: Supplementary file 3 — Source data Fig. 1 [file 44319_2026_745_MOESM3_ESM.zip › Figure 1/1D/Replicate Experiment/1D_Exp.2_CD25_29-Nov-2022.jpg]

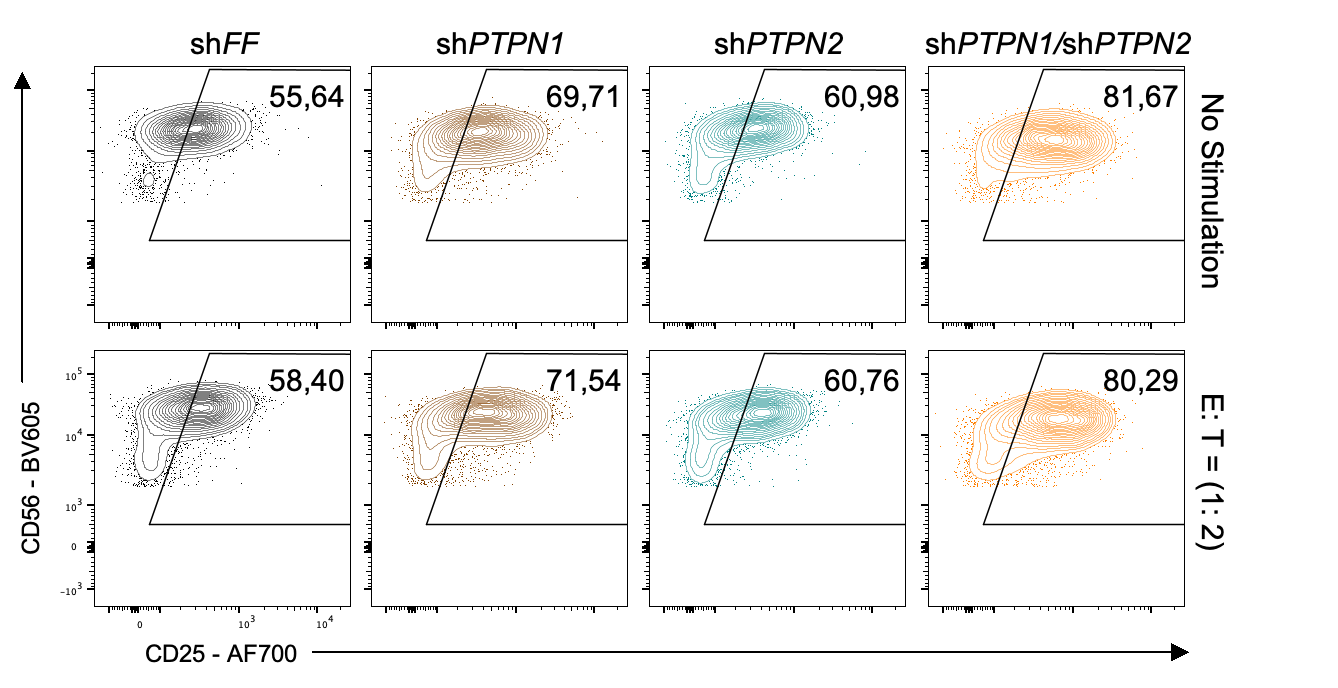

Supplement: Supplementary file 3 — Source data Fig. 1 [file 44319_2026_745_MOESM3_ESM.zip › Figure 1/1D/Replicate Experiment/1D_Exp.3_CD25.tiff]

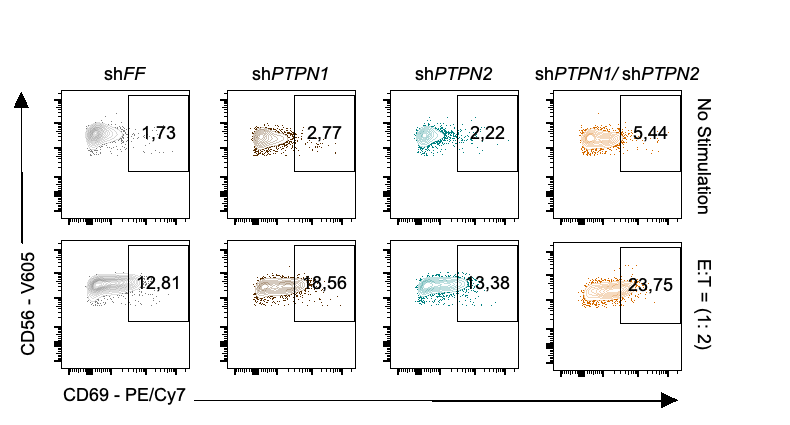

Supplement: Supplementary file 3 — Source data Fig. 1 [file 44319_2026_745_MOESM3_ESM.zip › Figure 1/1E/1E.tiff]

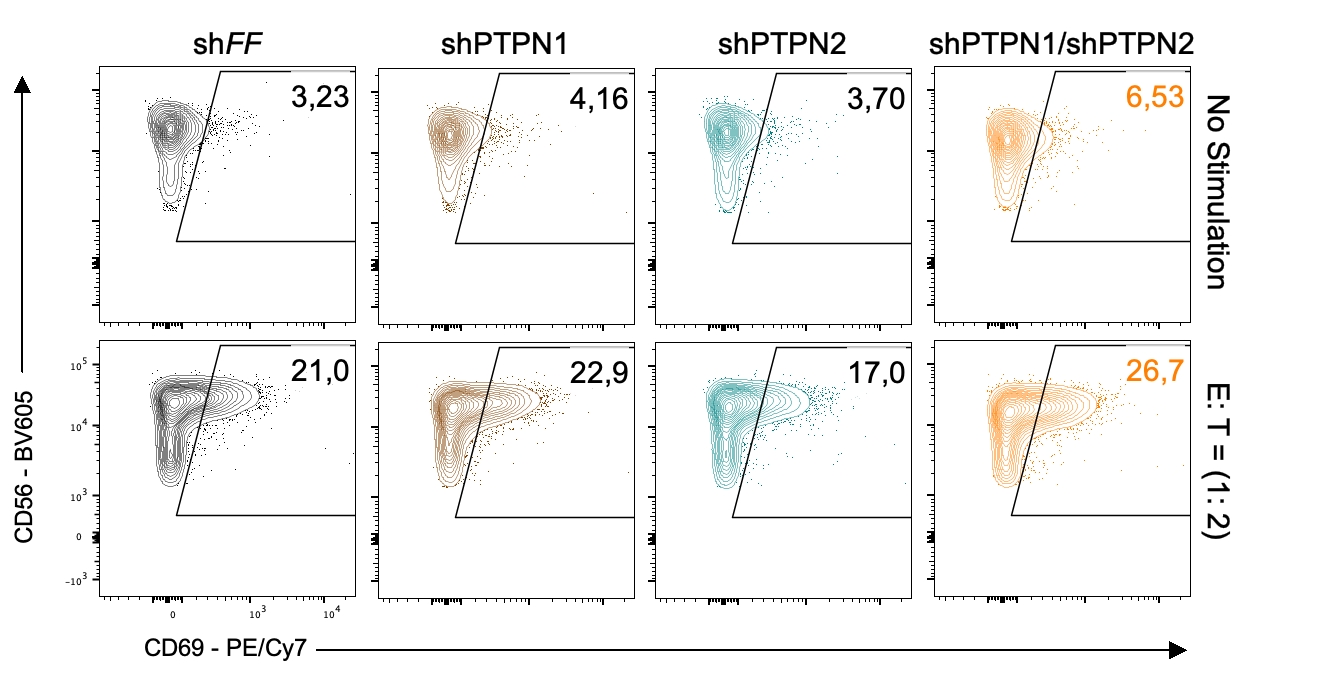

Supplement: Supplementary file 3 — Source data Fig. 1 [file 44319_2026_745_MOESM3_ESM.zip › Figure 1/1E/Replicate Experiments/1E_Exp.3_CD69_29-Nov-2022.jpg]

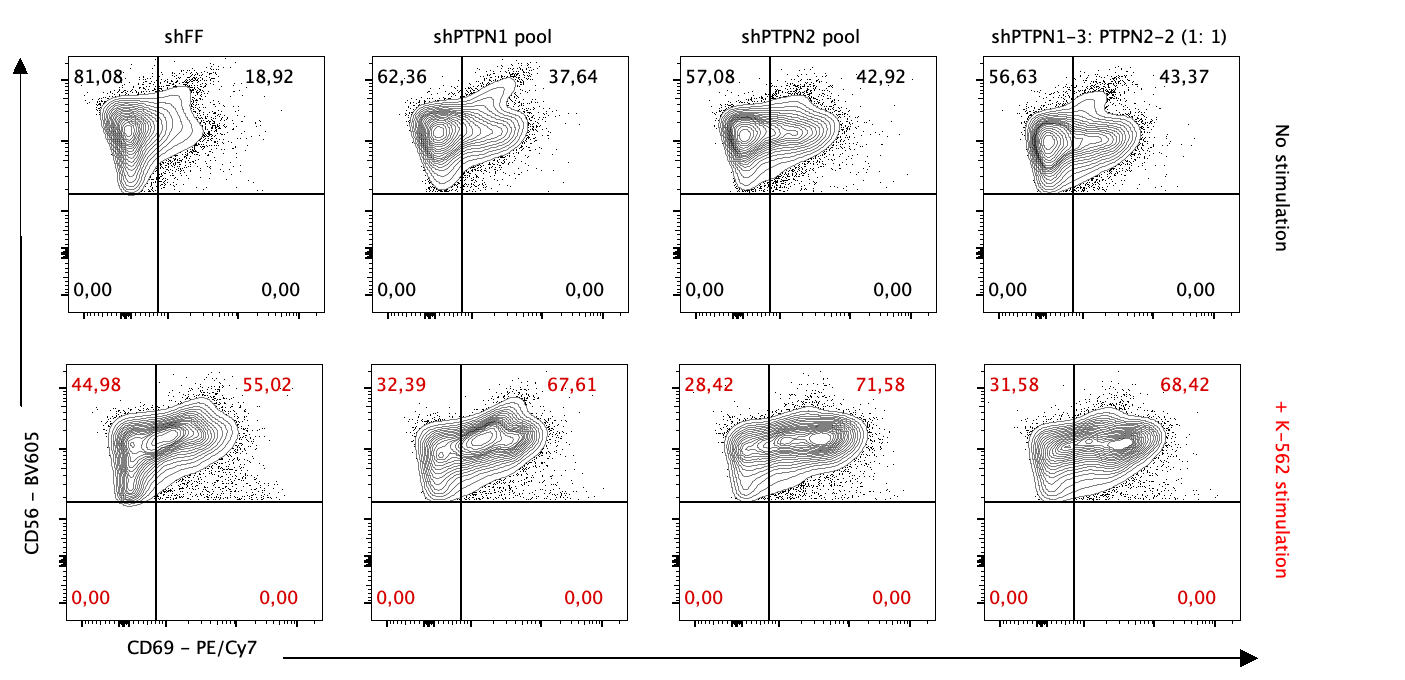

Supplement: Supplementary file 3 — Source data Fig. 1 [file 44319_2026_745_MOESM3_ESM.zip › Figure 1/1E/Replicate Experiments/1E EXP2_10-Feb-2021-CD69.png]

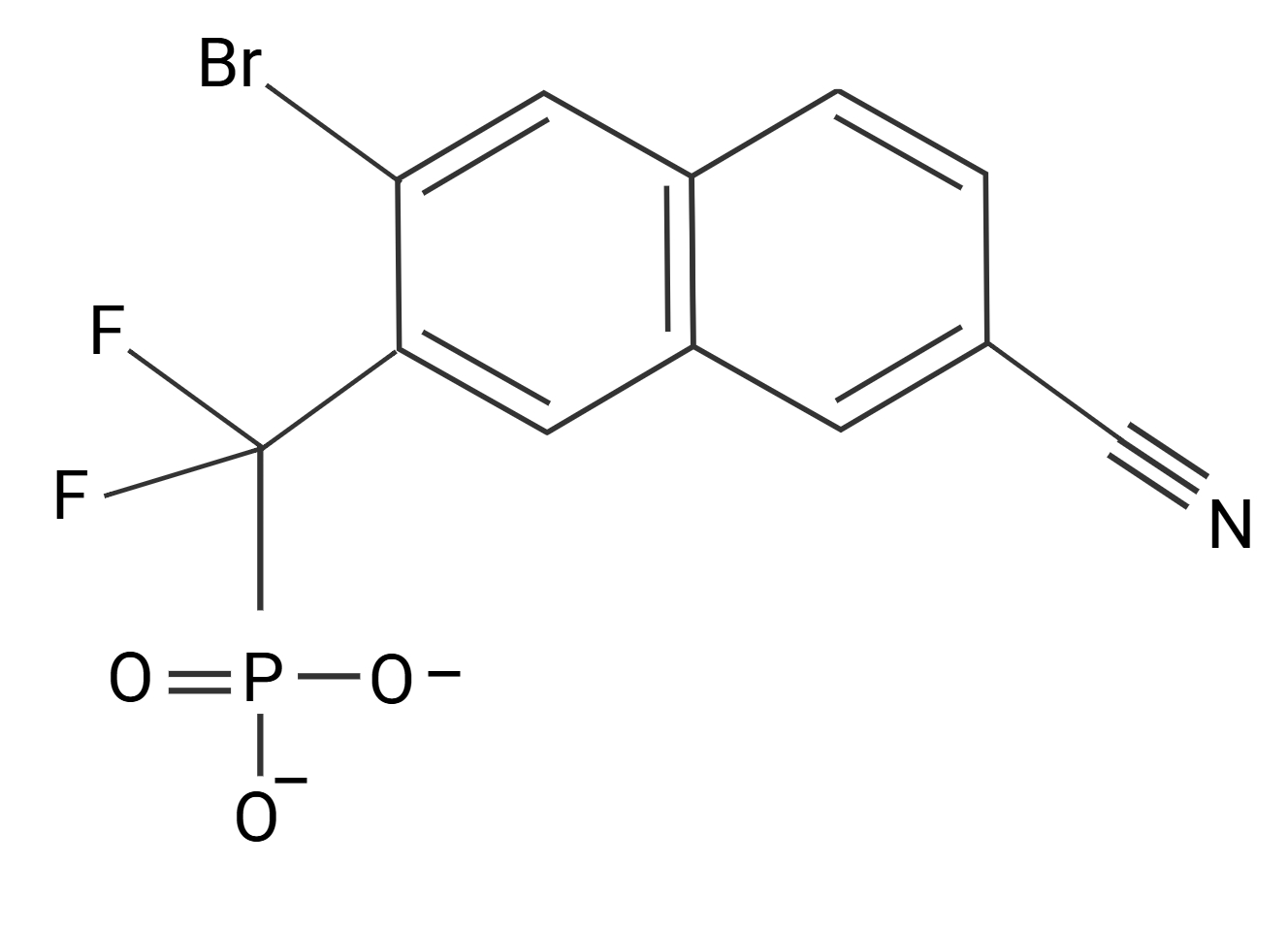

Supplement: Supplementary file 3 — Source data Fig. 1 [file 44319_2026_745_MOESM3_ESM.zip › Figure 1/1F/L598.jpeg]

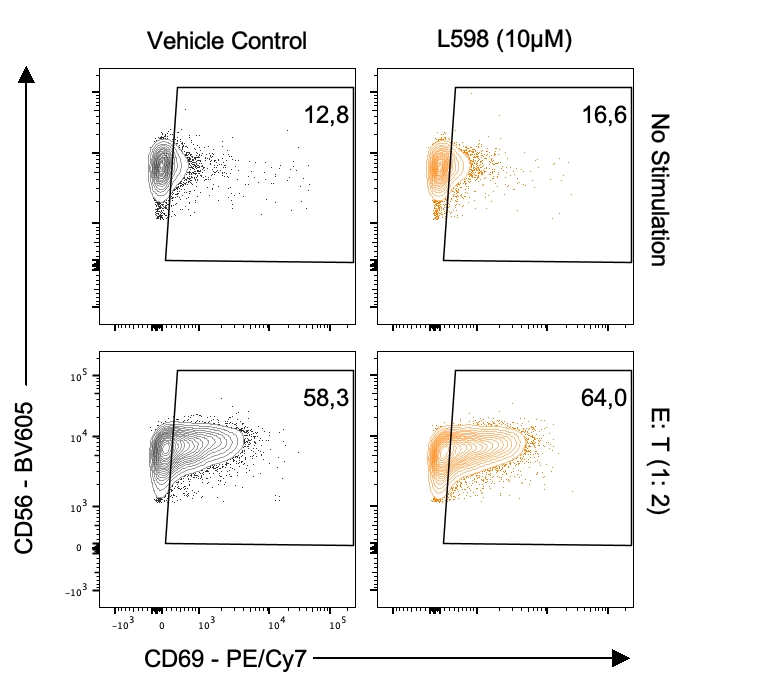

Supplement: Supplementary file 3 — Source data Fig. 1 [file 44319_2026_745_MOESM3_ESM.zip › Figure 1/1I/1I.jpg]

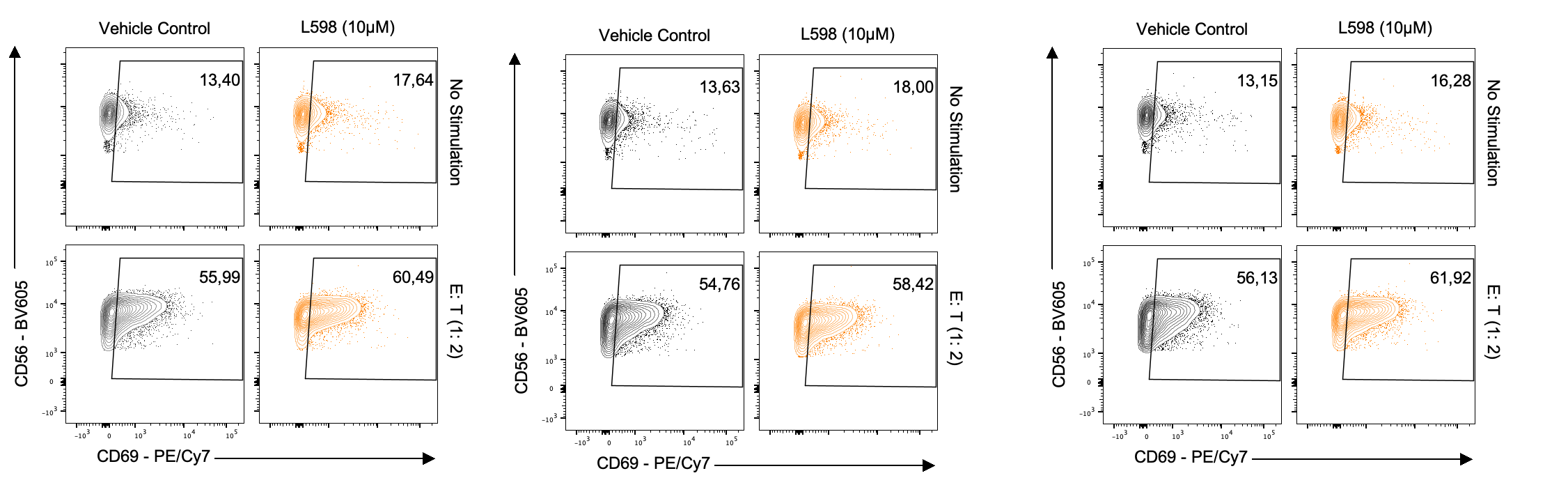

Supplement: Supplementary file 3 — Source data Fig. 1 [file 44319_2026_745_MOESM3_ESM.zip › Figure 1/1I/Repeats/1I_EXP2.tiff]

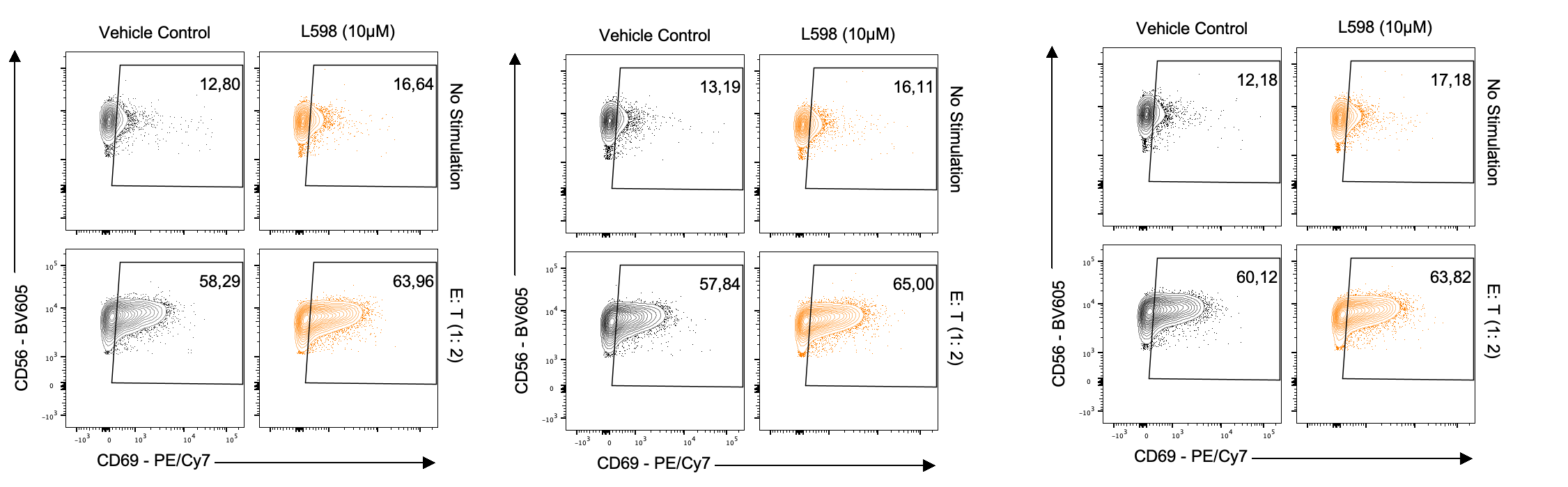

Supplement: Supplementary file 3 — Source data Fig. 1 [file 44319_2026_745_MOESM3_ESM.zip › Figure 1/1I/Repeats/1I_EXP3.tiff]

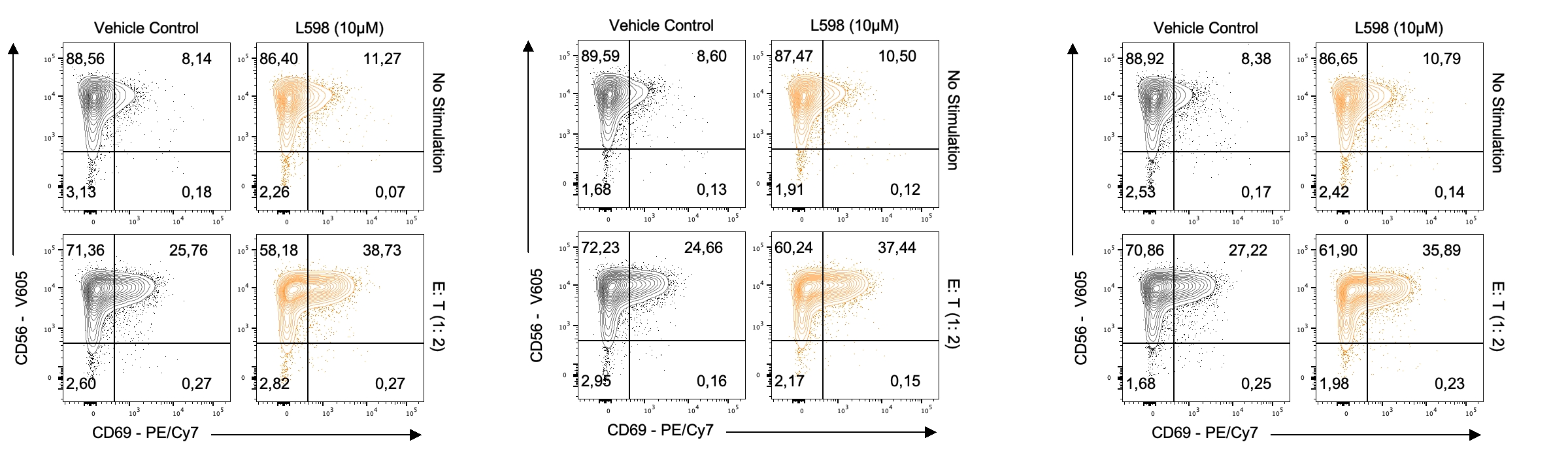

Supplement: Supplementary file 3 — Source data Fig. 1 [file 44319_2026_745_MOESM3_ESM.zip › Figure 1/1I/Repeats/1I_EXP.1.jpg]

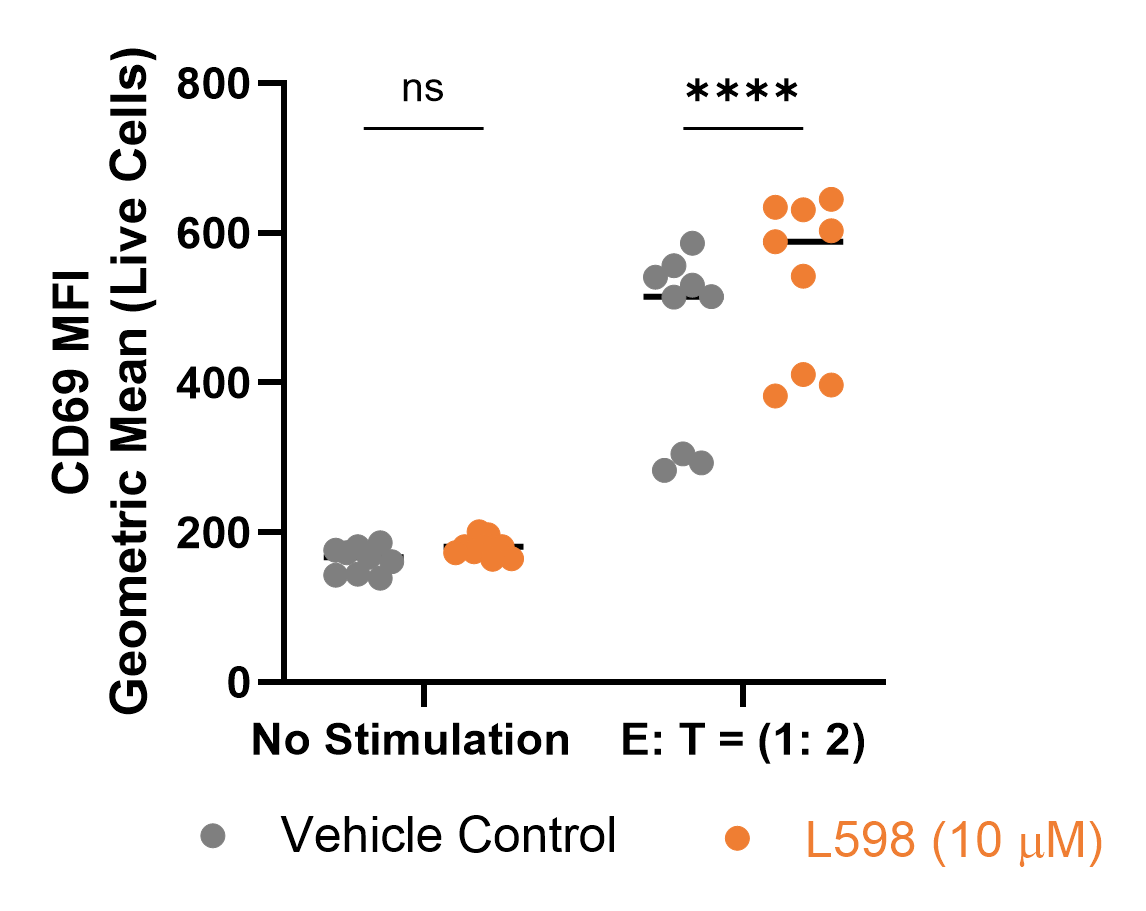

Supplement: Supplementary file 3 — Source data Fig. 1 [file 44319_2026_745_MOESM3_ESM.zip › Figure 1/1J/1J.tif]

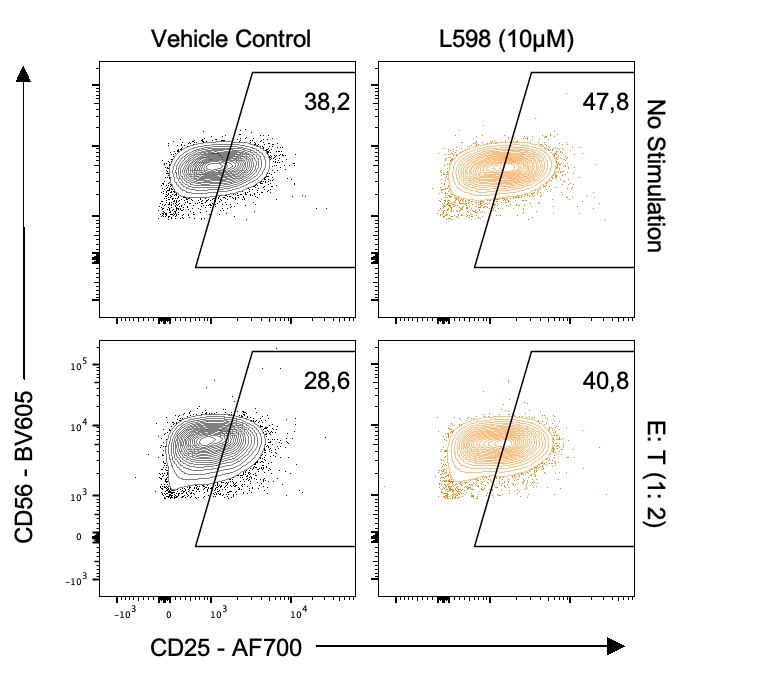

Supplement: Supplementary file 3 — Source data Fig. 1 [file 44319_2026_745_MOESM3_ESM.zip › Figure 1/1G/1G.jpg]

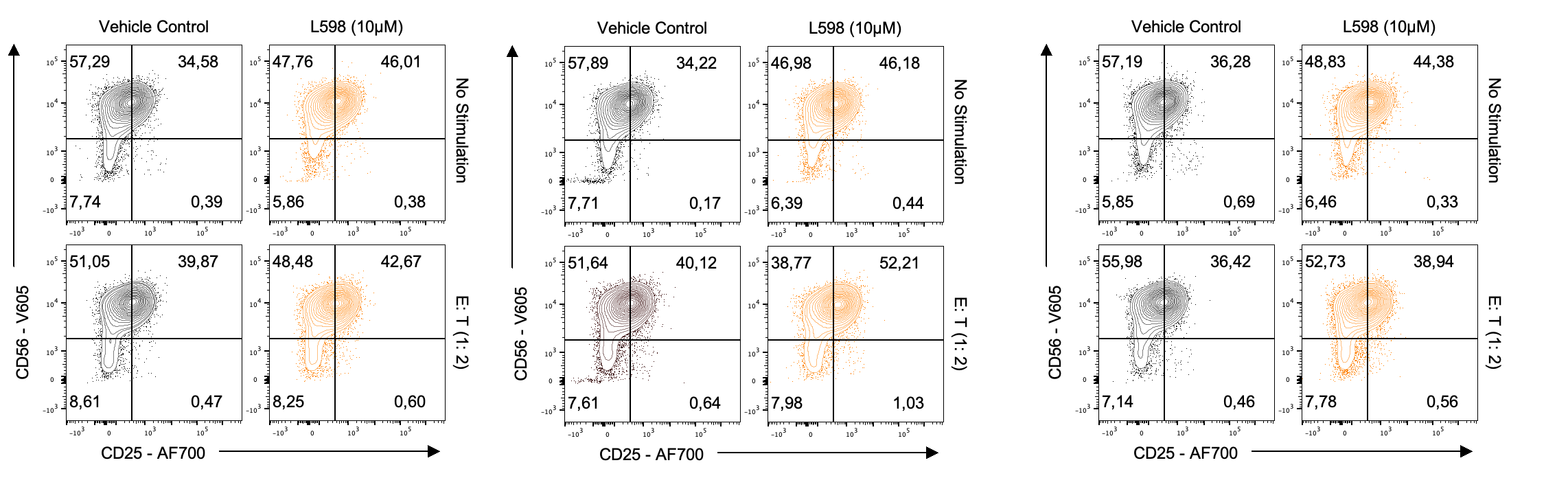

Supplement: Supplementary file 3 — Source data Fig. 1 [file 44319_2026_745_MOESM3_ESM.zip › Figure 1/1G/Repeats/1G_EXP1.tiff]

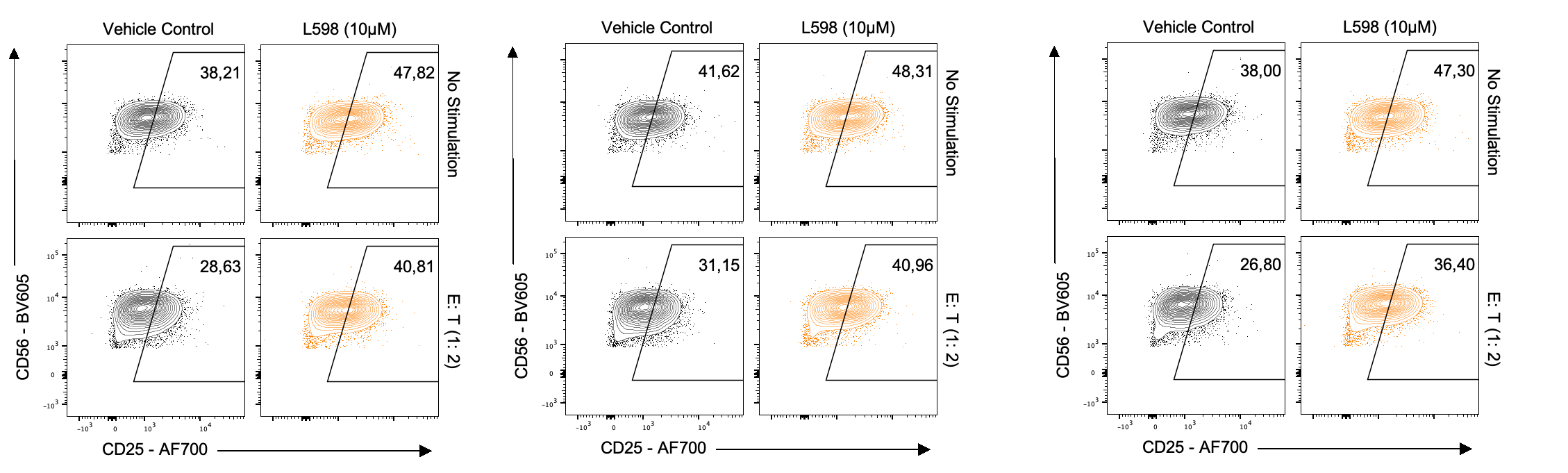

Supplement: Supplementary file 3 — Source data Fig. 1 [file 44319_2026_745_MOESM3_ESM.zip › Figure 1/1G/Repeats/1G_EXP3.tiff]

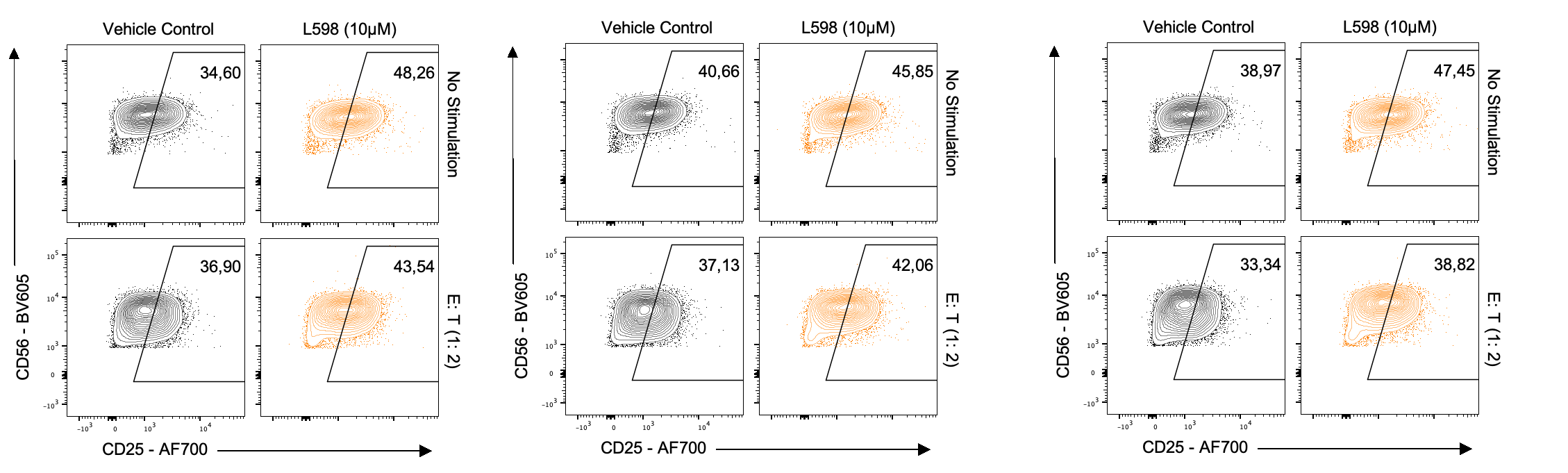

Supplement: Supplementary file 3 — Source data Fig. 1 [file 44319_2026_745_MOESM3_ESM.zip › Figure 1/1G/Repeats/1G_EXP2.tiff]

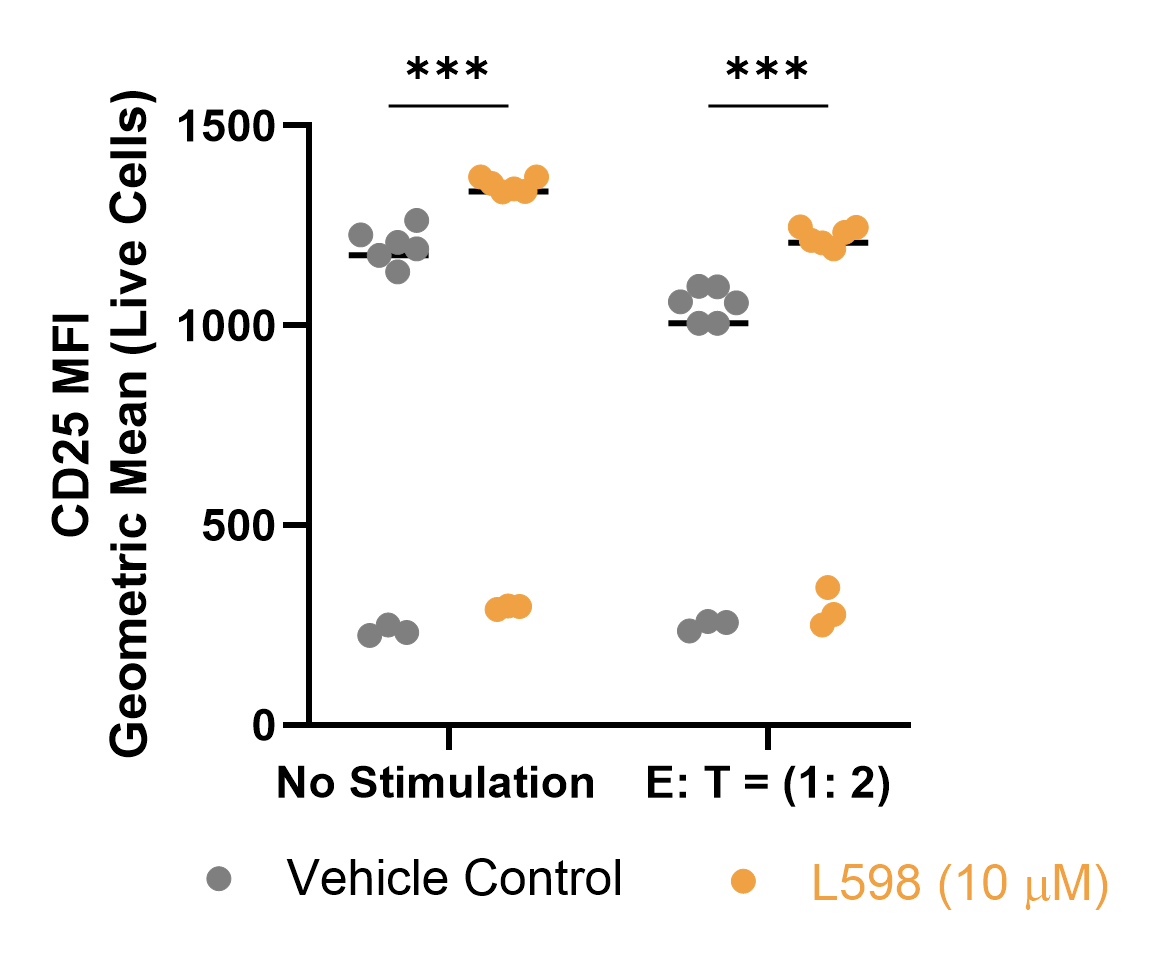

Supplement: Supplementary file 3 — Source data Fig. 1 [file 44319_2026_745_MOESM3_ESM.zip › Figure 1/1H/1H.tif]

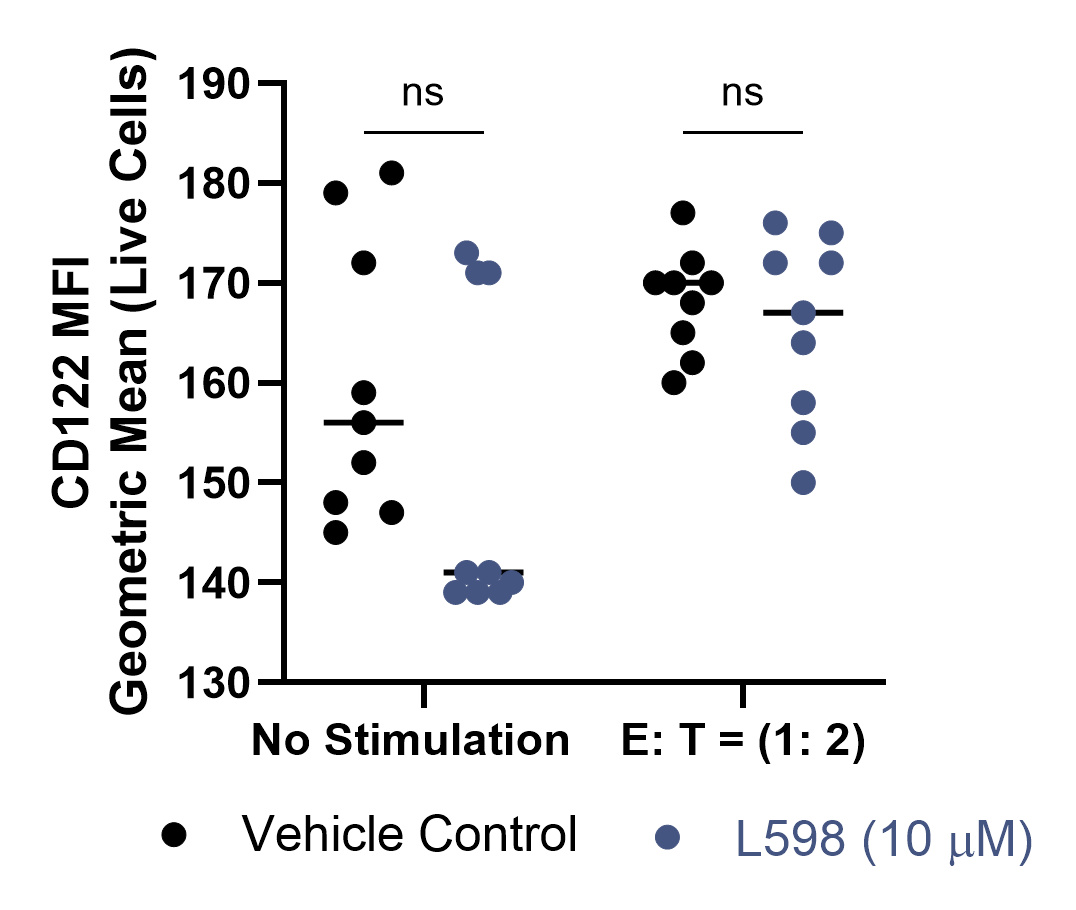

Supplement: Supplementary file 3 — Source data Fig. 1 [file 44319_2026_745_MOESM3_ESM.zip › Figure 1/1K/1K CD122 Experiment 1+2+3.tif]

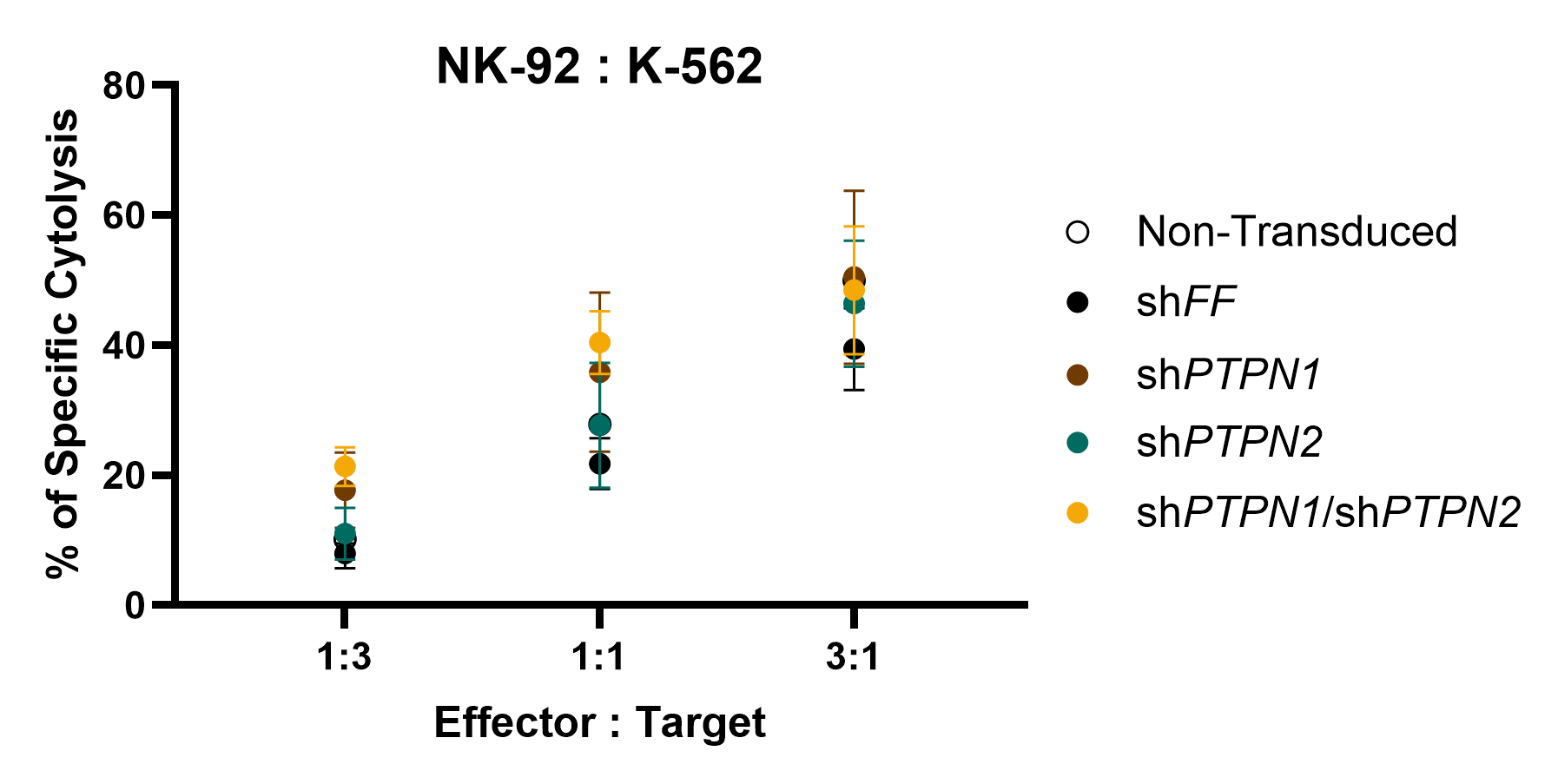

Supplement: Supplementary file 4 — Source data Fig. 2 [file 44319_2026_745_MOESM4_ESM.zip › Figure 2/2A/2A_combined 2 experiments.tif]

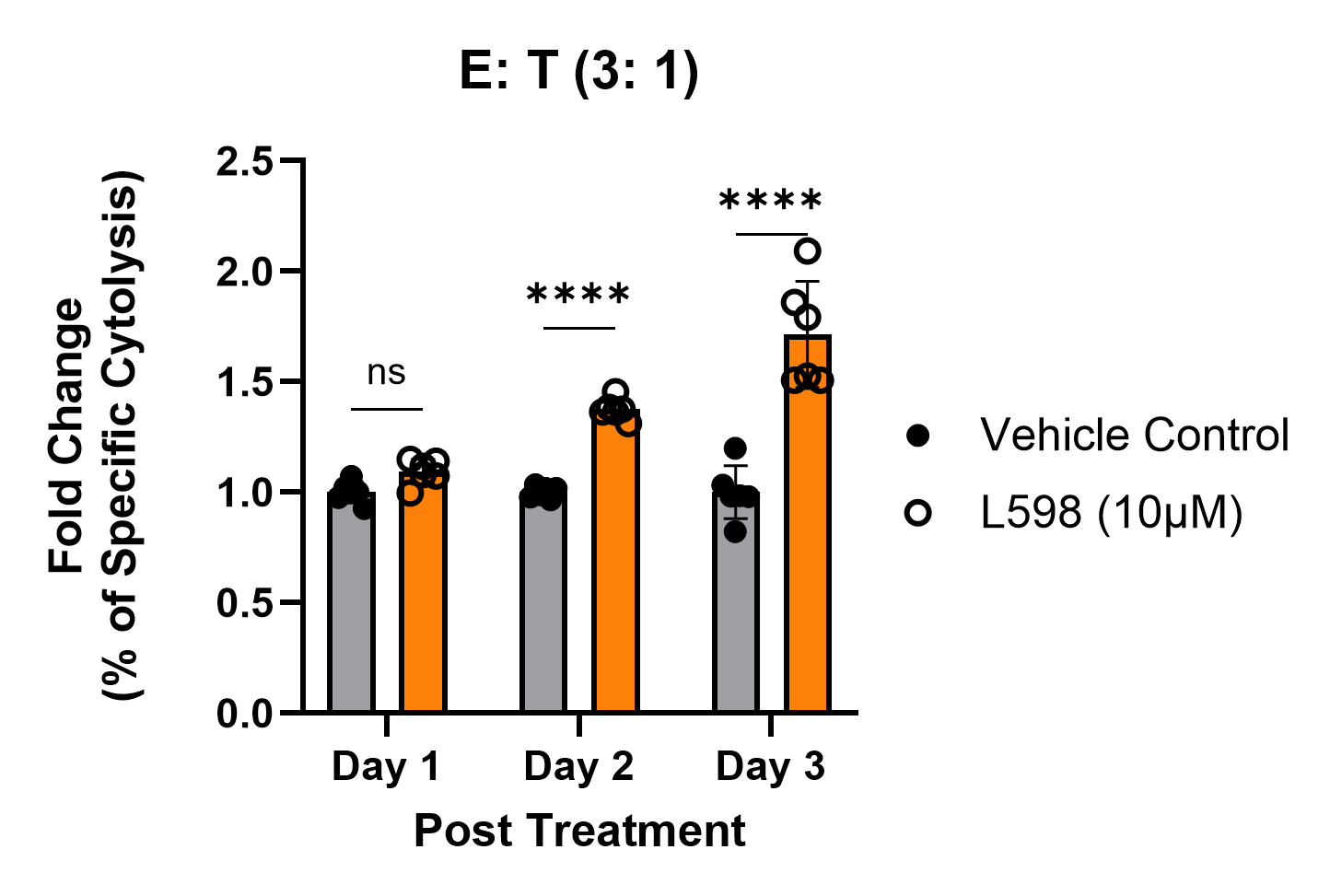

Supplement: Supplementary file 4 — Source data Fig. 2 [file 44319_2026_745_MOESM4_ESM.zip › Figure 2/2B/2B.tif]

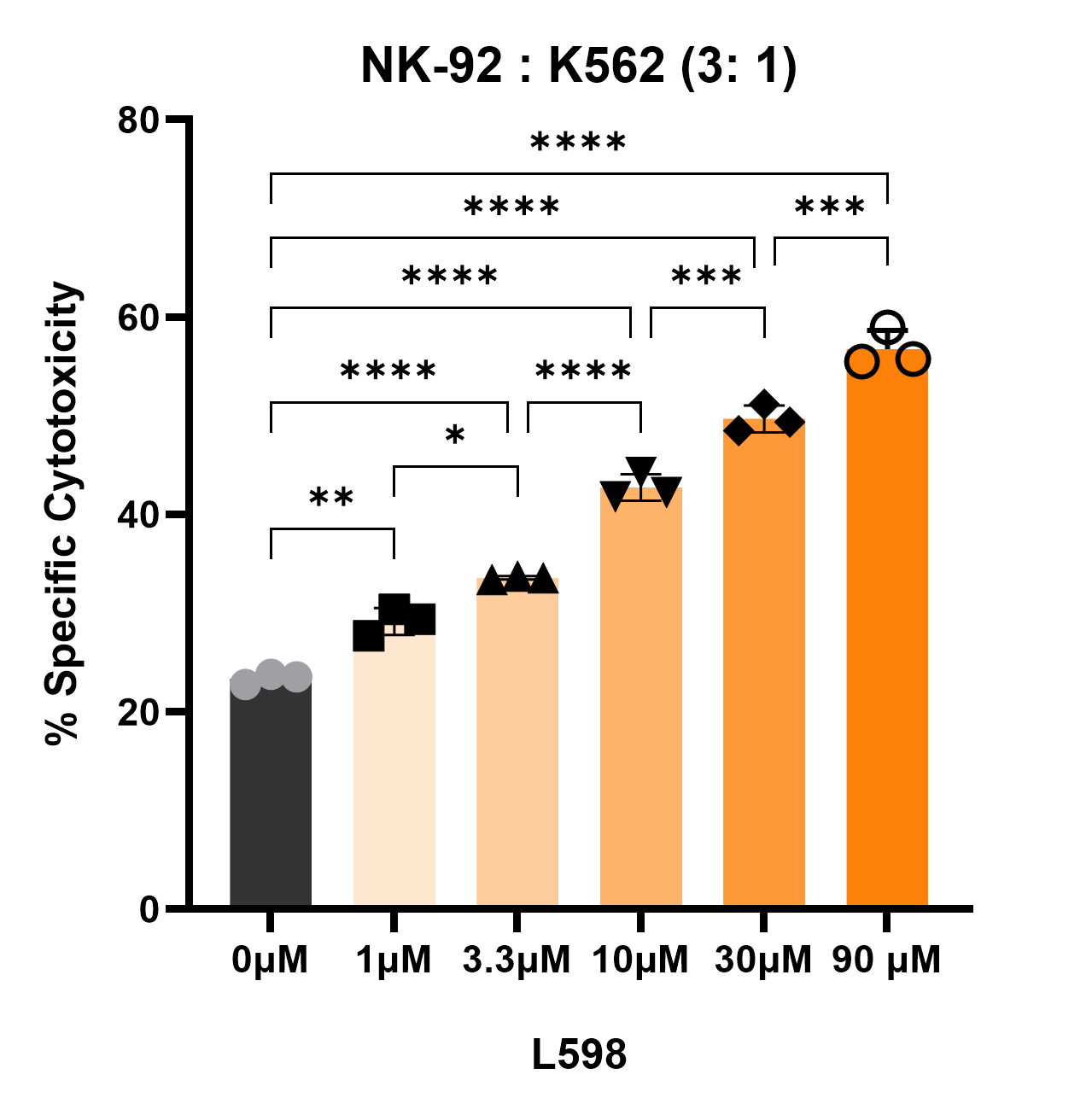

Supplement: Supplementary file 4 — Source data Fig. 2 [file 44319_2026_745_MOESM4_ESM.zip › Figure 2/2C/2C.tif]

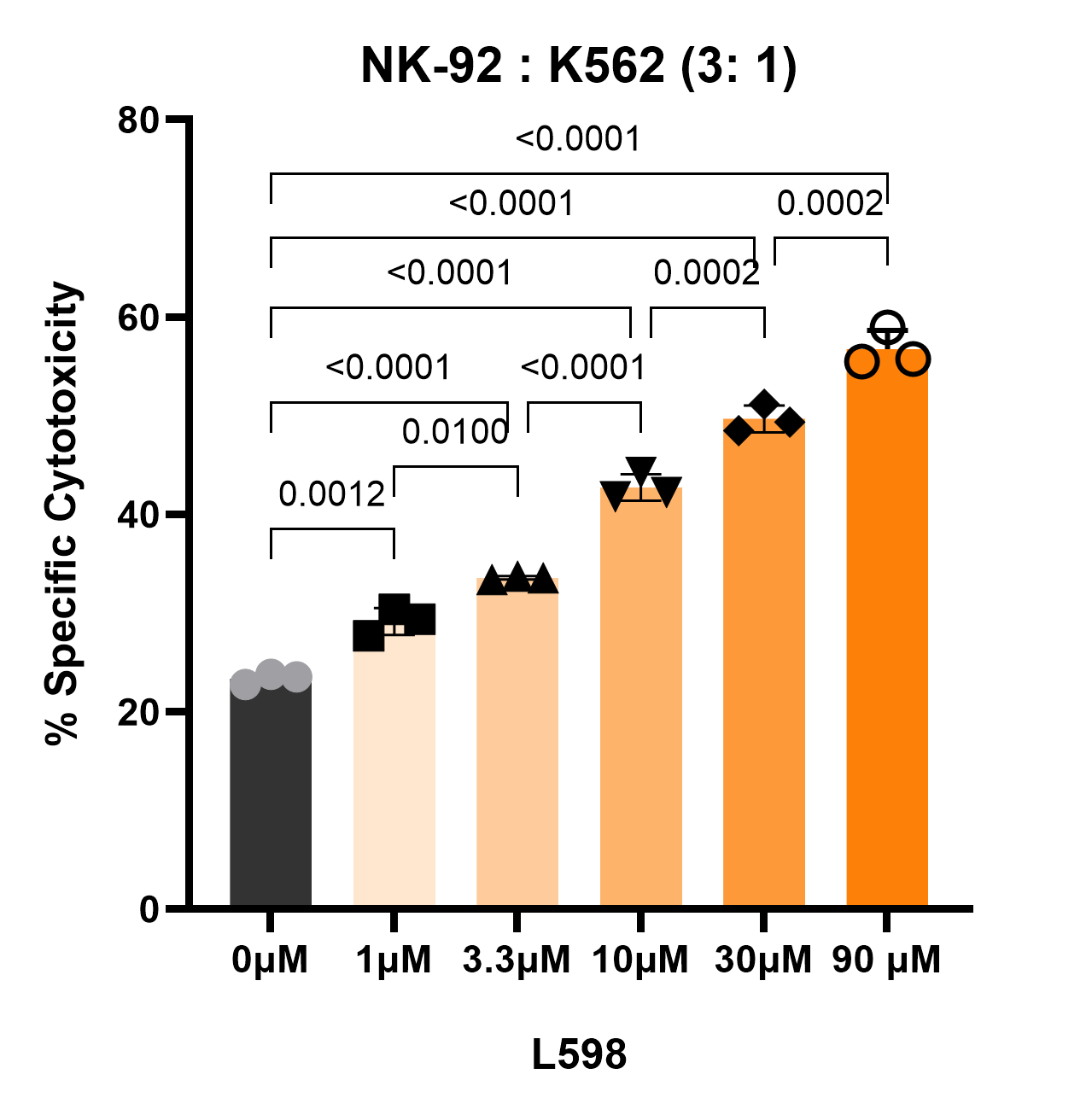

Supplement: Supplementary file 4 — Source data Fig. 2 [file 44319_2026_745_MOESM4_ESM.zip › Figure 2/2C/2C P values.tif]

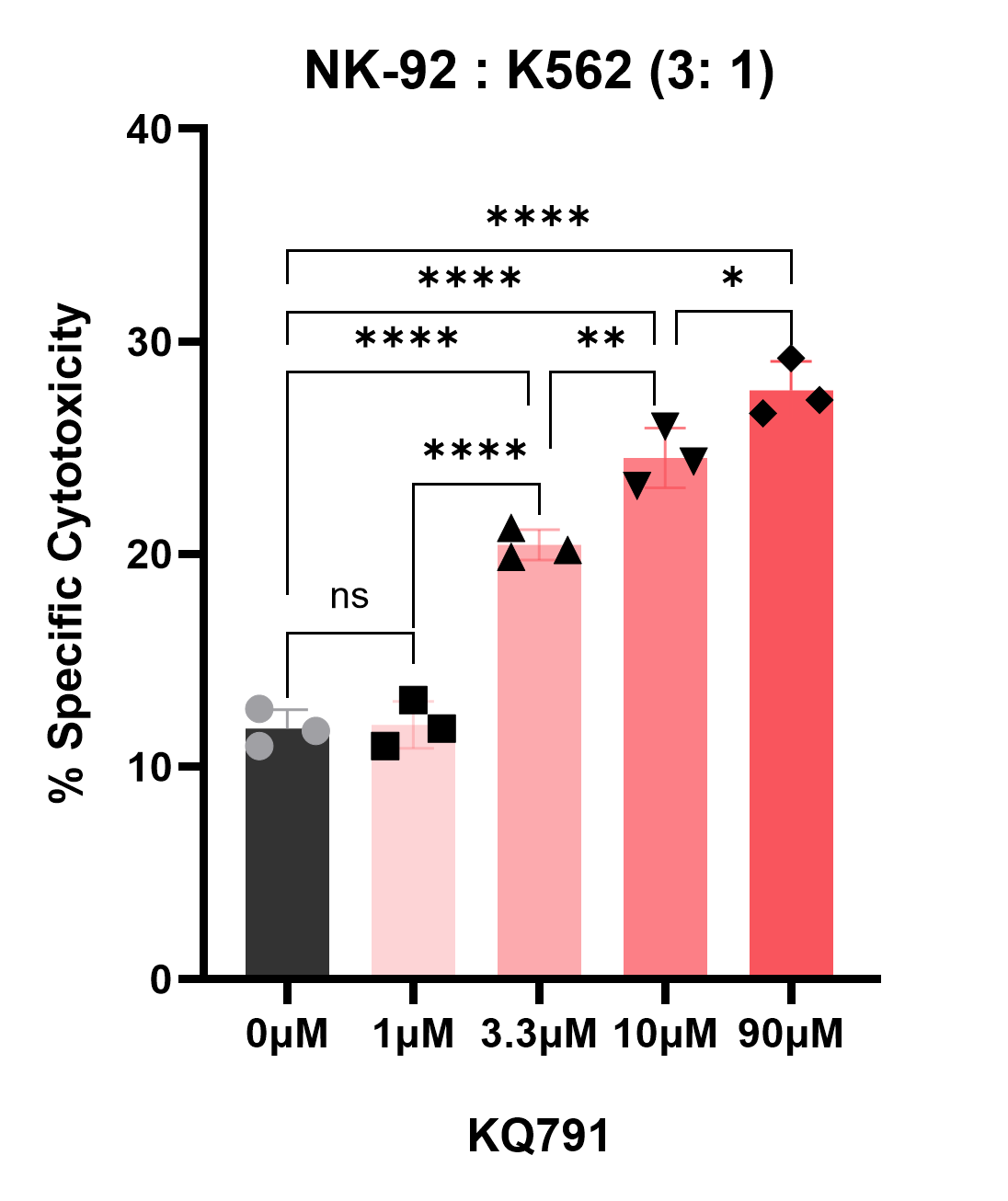

Supplement: Supplementary file 4 — Source data Fig. 2 [file 44319_2026_745_MOESM4_ESM.zip › Figure 2/2D/2D.tif]

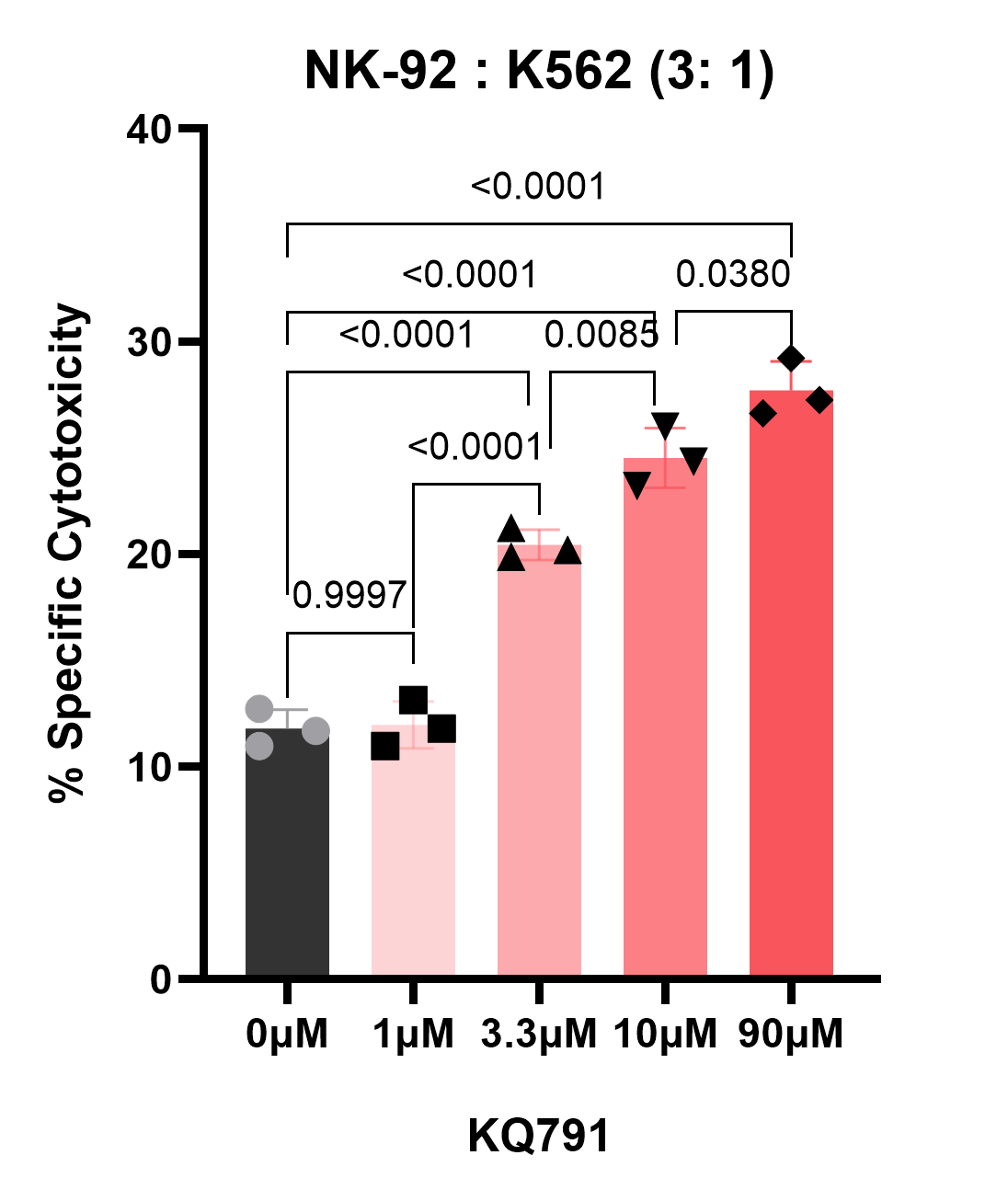

Supplement: Supplementary file 4 — Source data Fig. 2 [file 44319_2026_745_MOESM4_ESM.zip › Figure 2/2D/2D P-values.tif]

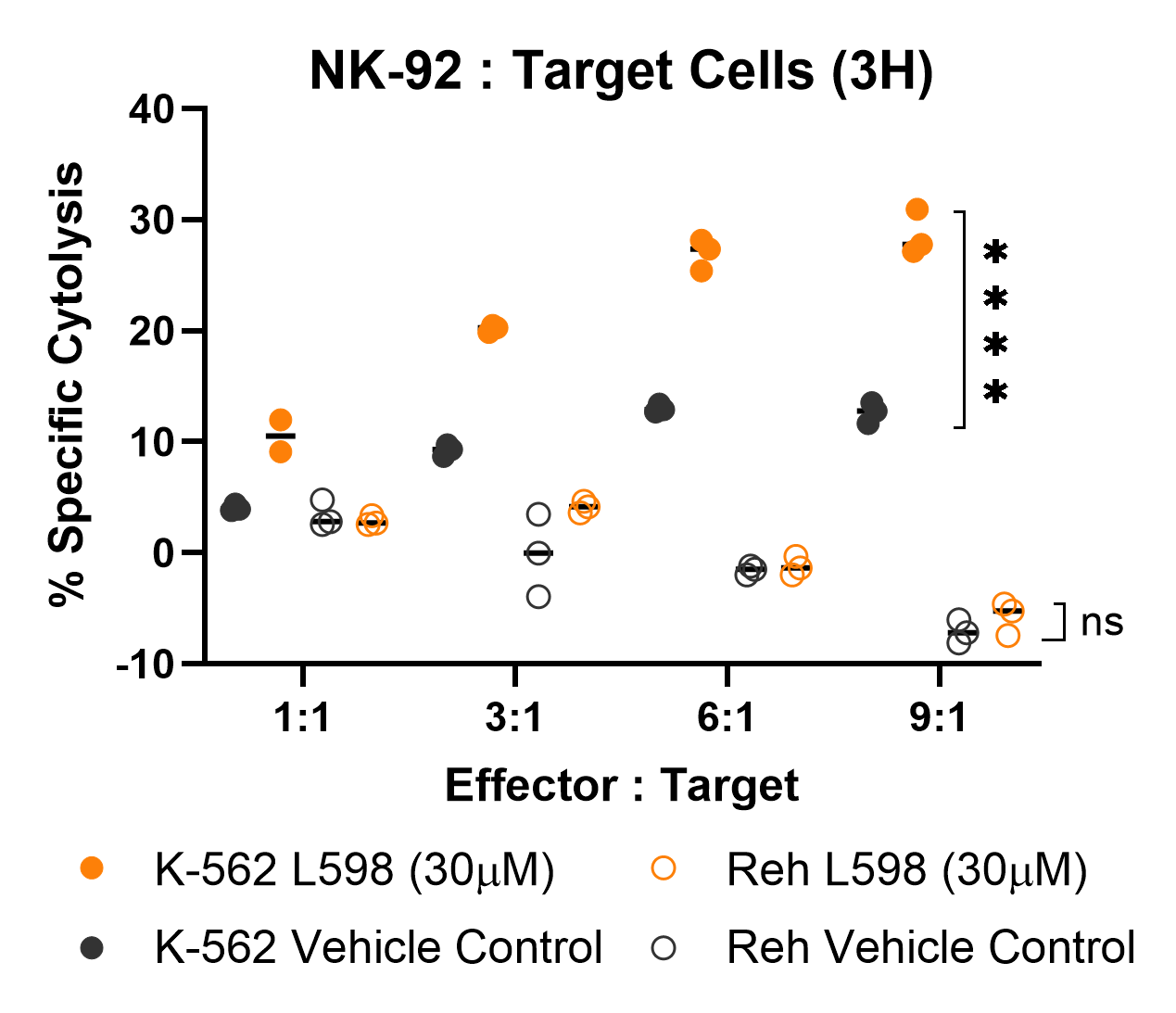

Supplement: Supplementary file 4 — Source data Fig. 2 [file 44319_2026_745_MOESM4_ESM.zip › Figure 2/2E/2E.tif]

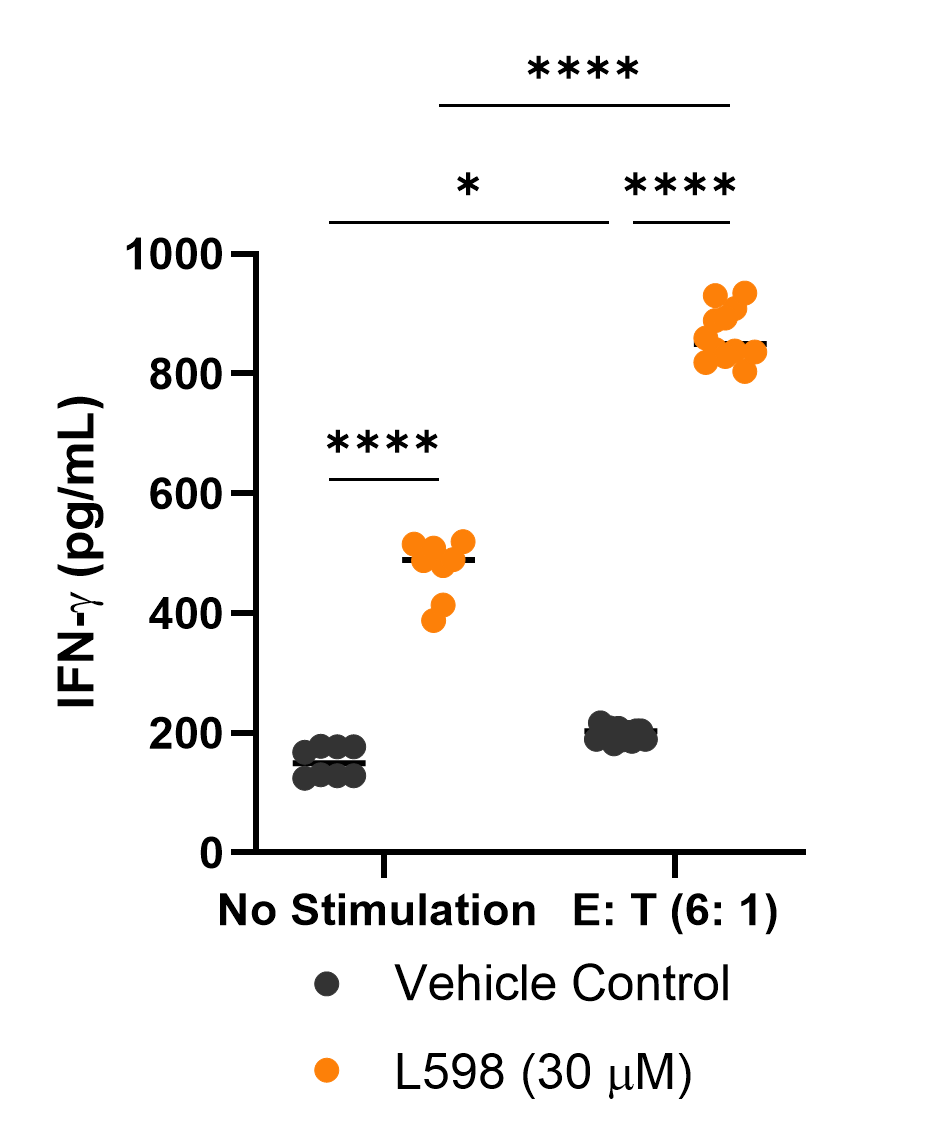

Supplement: Supplementary file 4 — Source data Fig. 2 [file 44319_2026_745_MOESM4_ESM.zip › Figure 2/2F/2F.tif]

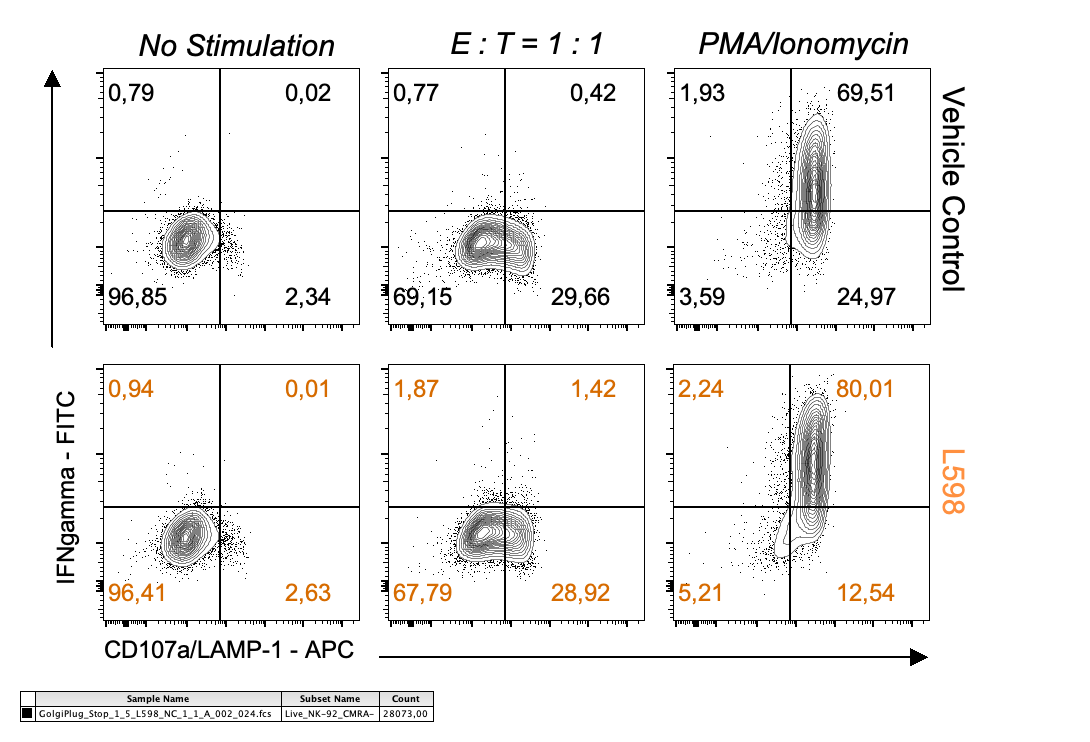

Supplement: Supplementary file 4 — Source data Fig. 2 [file 44319_2026_745_MOESM4_ESM.zip › Figure 2/2G/2G.tiff]

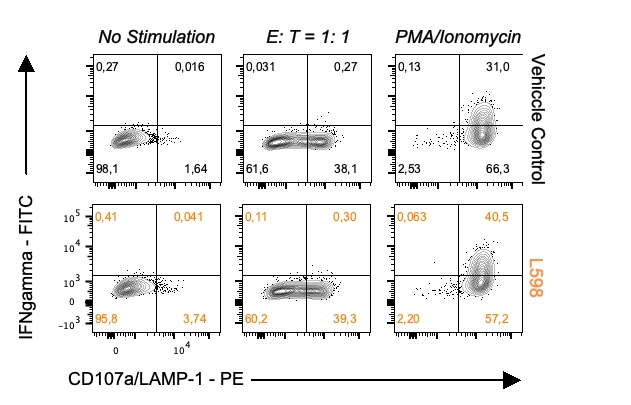

Supplement: Supplementary file 4 — Source data Fig. 2 [file 44319_2026_745_MOESM4_ESM.zip › Figure 2/2G/Repeats/2G_Exp2.jpg]

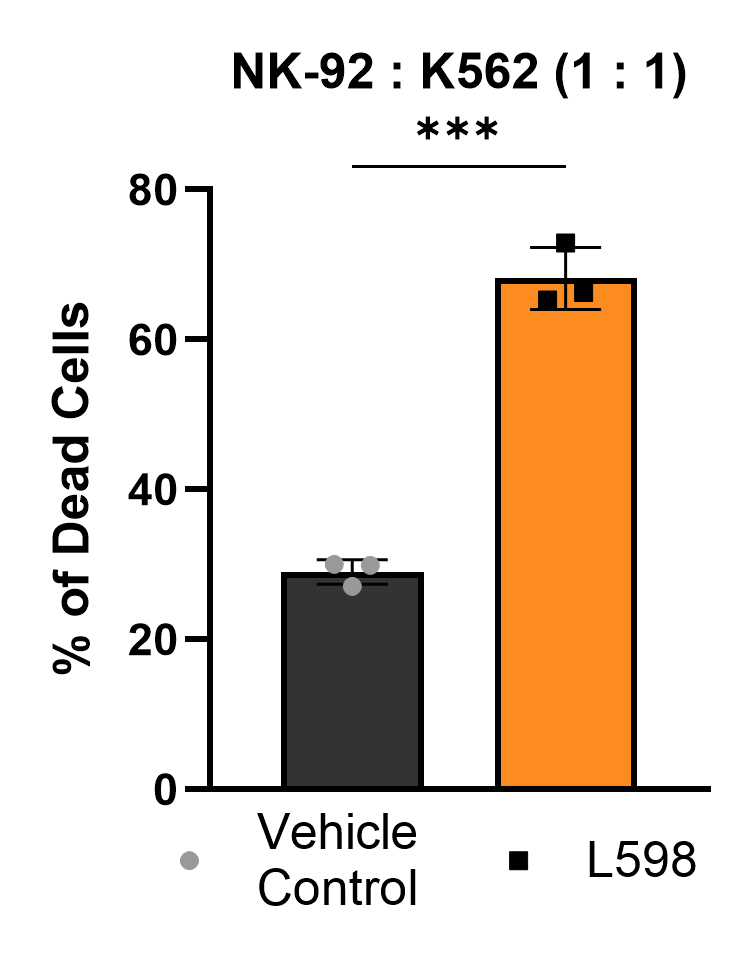

Supplement: Supplementary file 4 — Source data Fig. 2 [file 44319_2026_745_MOESM4_ESM.zip › Figure 2/2H/2H.tif]

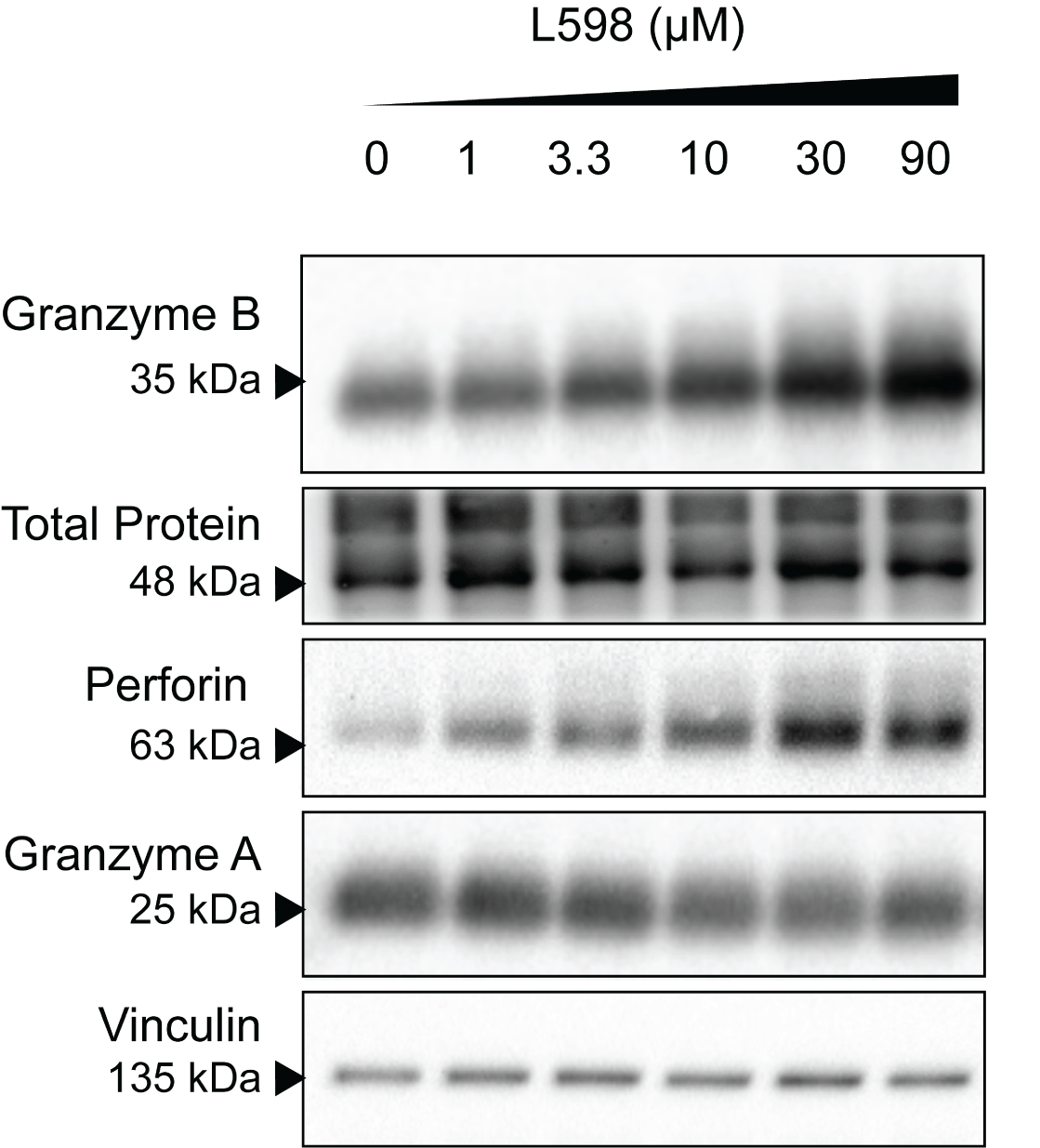

Supplement: Supplementary file 4 — Source data Fig. 2 [file 44319_2026_745_MOESM4_ESM.zip › Figure 2/2I/2I.tif]

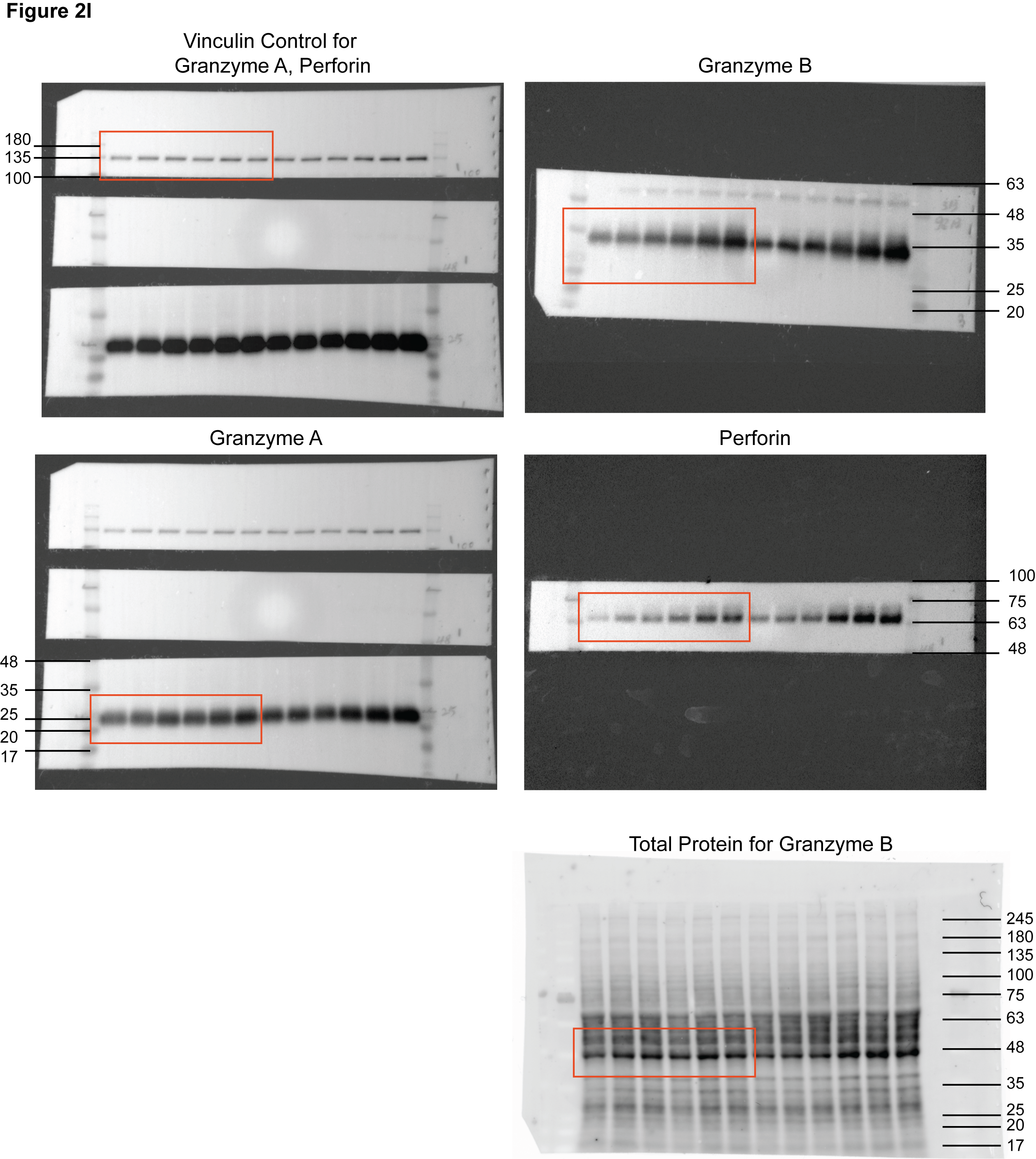

Supplement: Supplementary file 4 — Source data Fig. 2 [file 44319_2026_745_MOESM4_ESM.zip › Figure 2/2I/Replicates/2I_EXP1.tif]

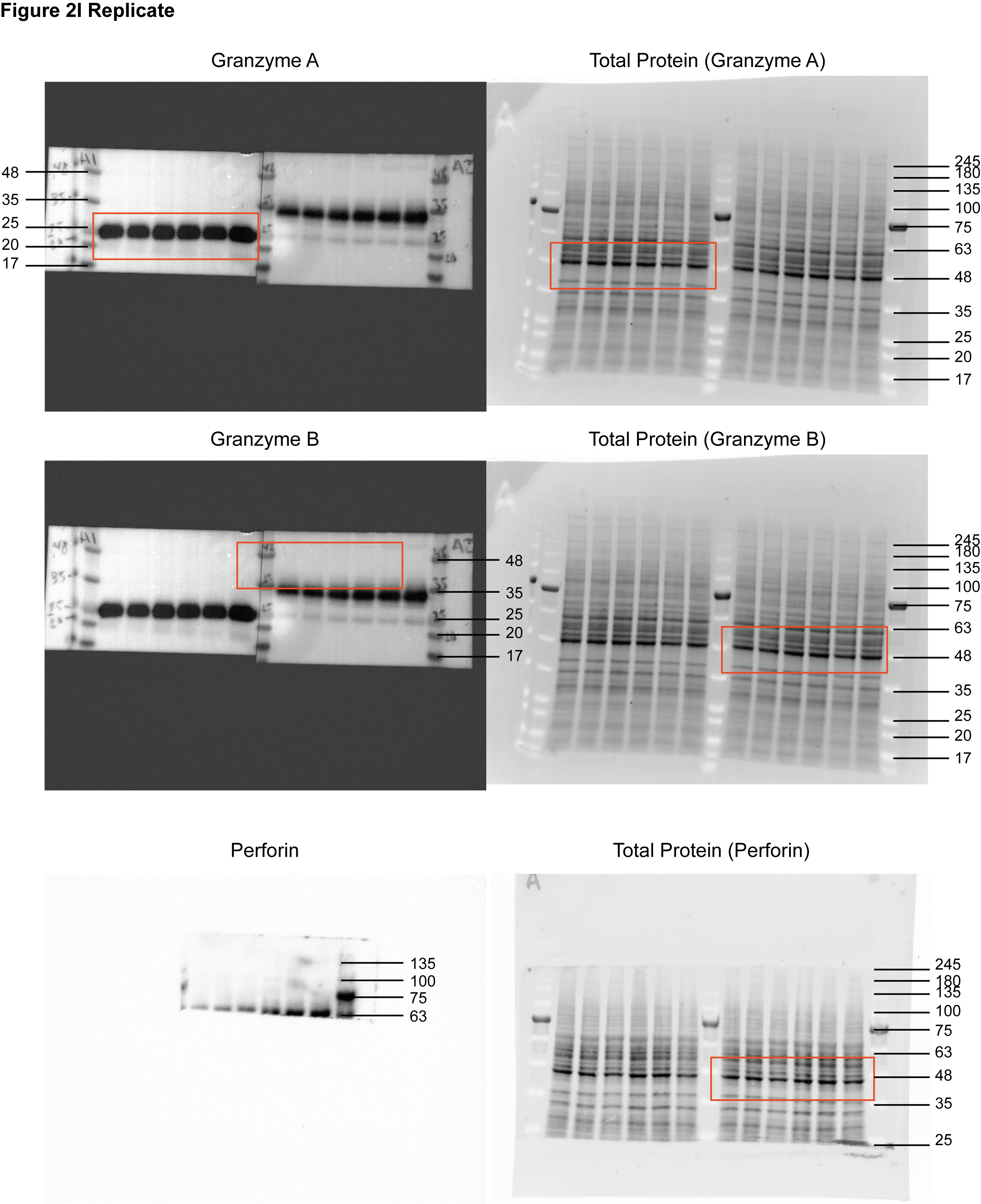

Supplement: Supplementary file 4 — Source data Fig. 2 [file 44319_2026_745_MOESM4_ESM.zip › Figure 2/2I/Replicates/2I_EXP2.tif]

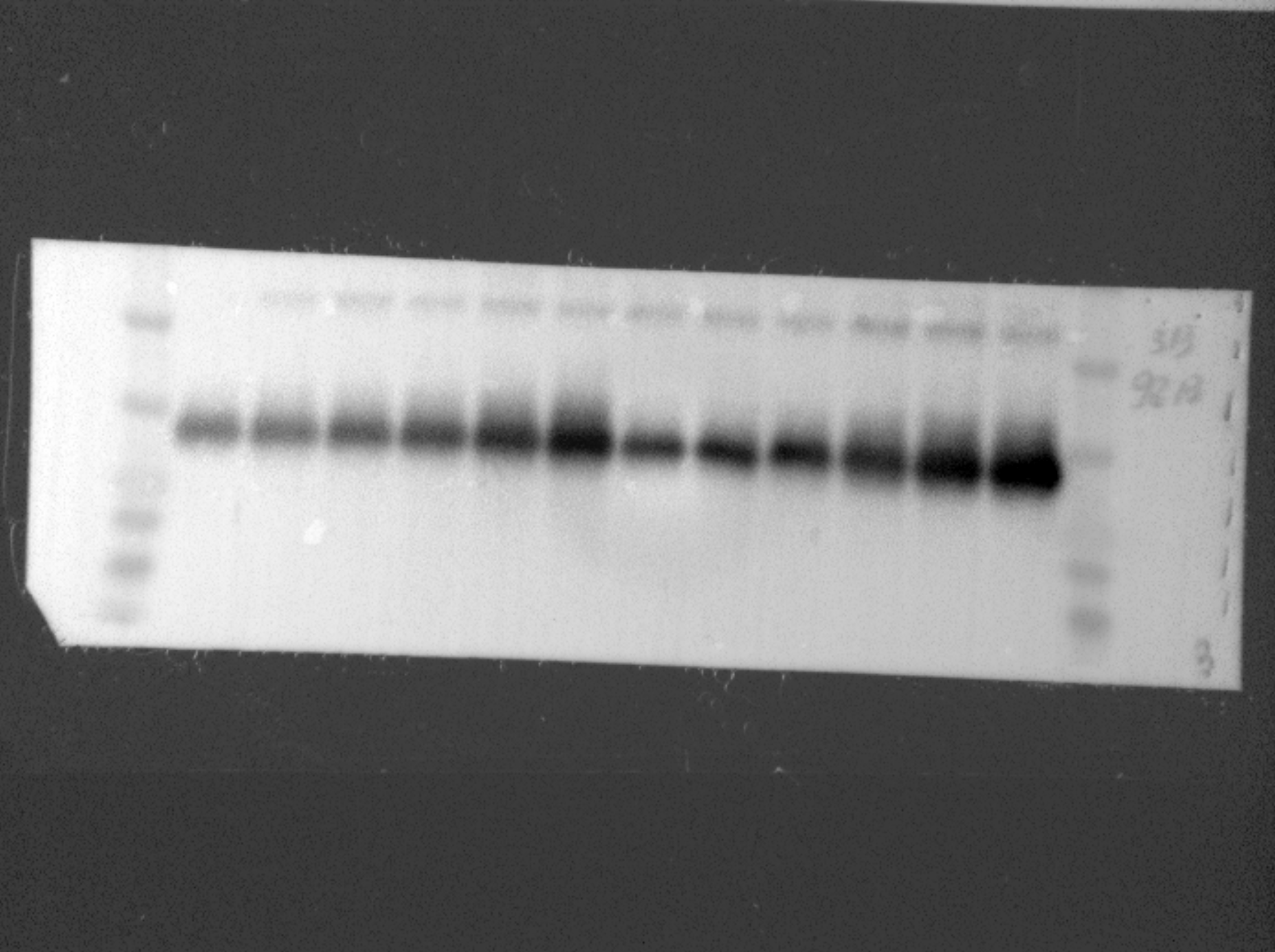

Supplement: Supplementary file 4 — Source data Fig. 2 [file 44319_2026_745_MOESM4_ESM.zip › Figure 2/2I/Replicates/Raw Data/EXP1/EXP1_5.9sec+gzmb colori.tif]

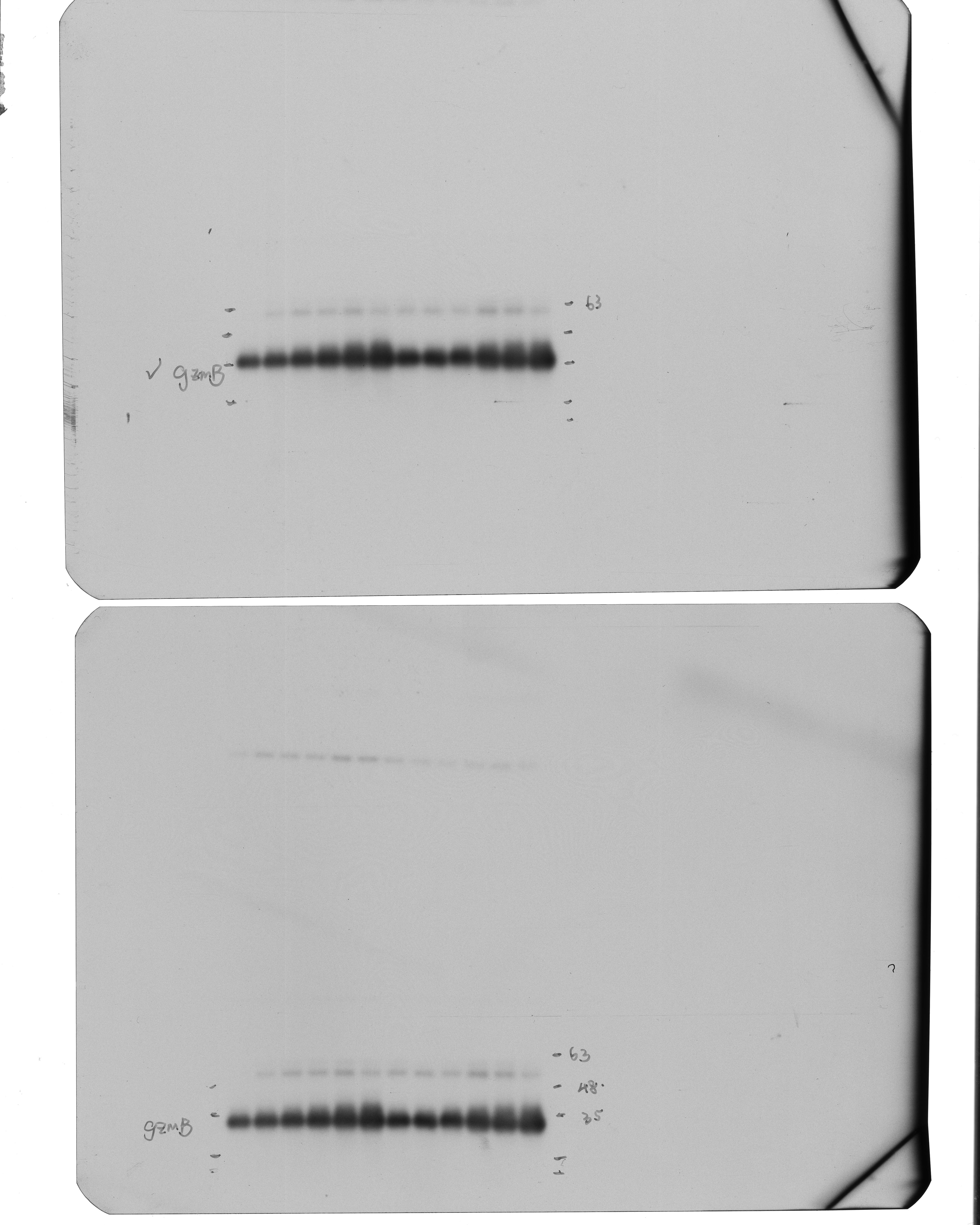

Supplement: Supplementary file 4 — Source data Fig. 2 [file 44319_2026_745_MOESM4_ESM.zip › Figure 2/2I/Replicates/Raw Data/EXP1/EXP1_20201024_gzmb010.jpg]

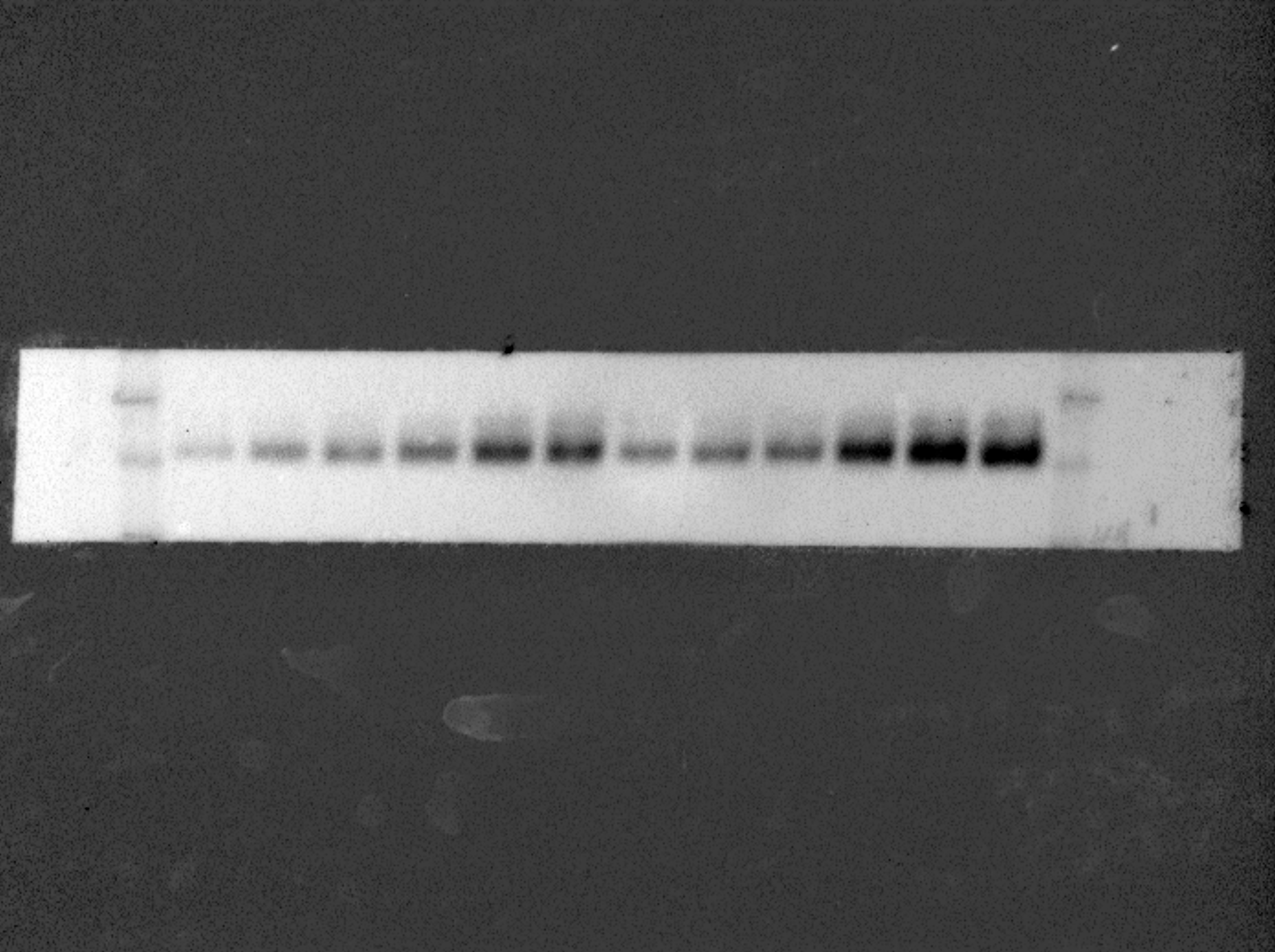

Supplement: Supplementary file 4 — Source data Fig. 2 [file 44319_2026_745_MOESM4_ESM.zip › Figure 2/2I/Replicates/Raw Data/EXP1/EXP1_PRF1_colori_59.0sec.tif]

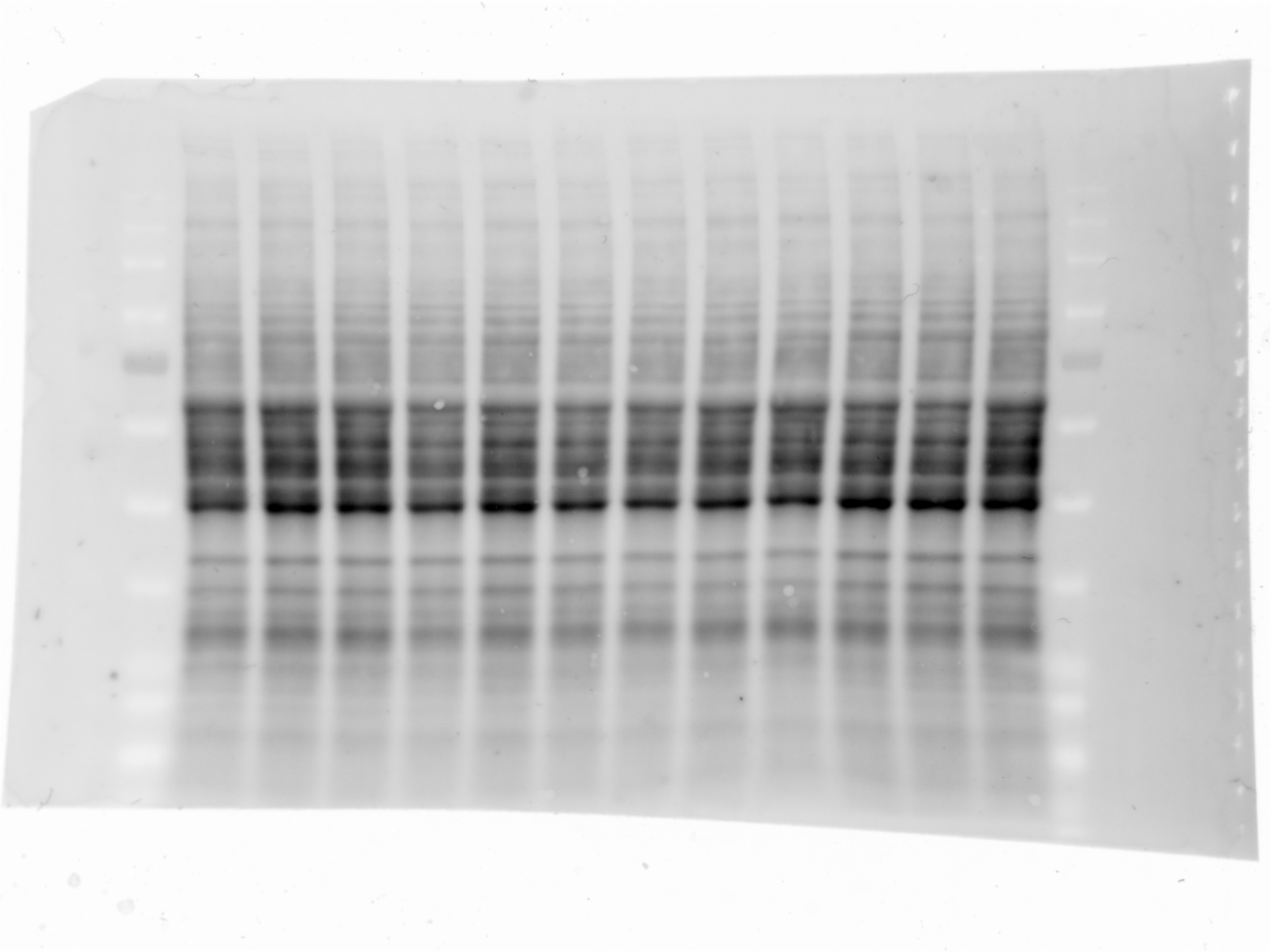

Supplement: Supplementary file 4 — Source data Fig. 2 [file 44319_2026_745_MOESM4_ESM.zip › Figure 2/2I/Replicates/Raw Data/EXP1/EXP1_total Protein_GZA PRF1_gel1 10percent.tif]

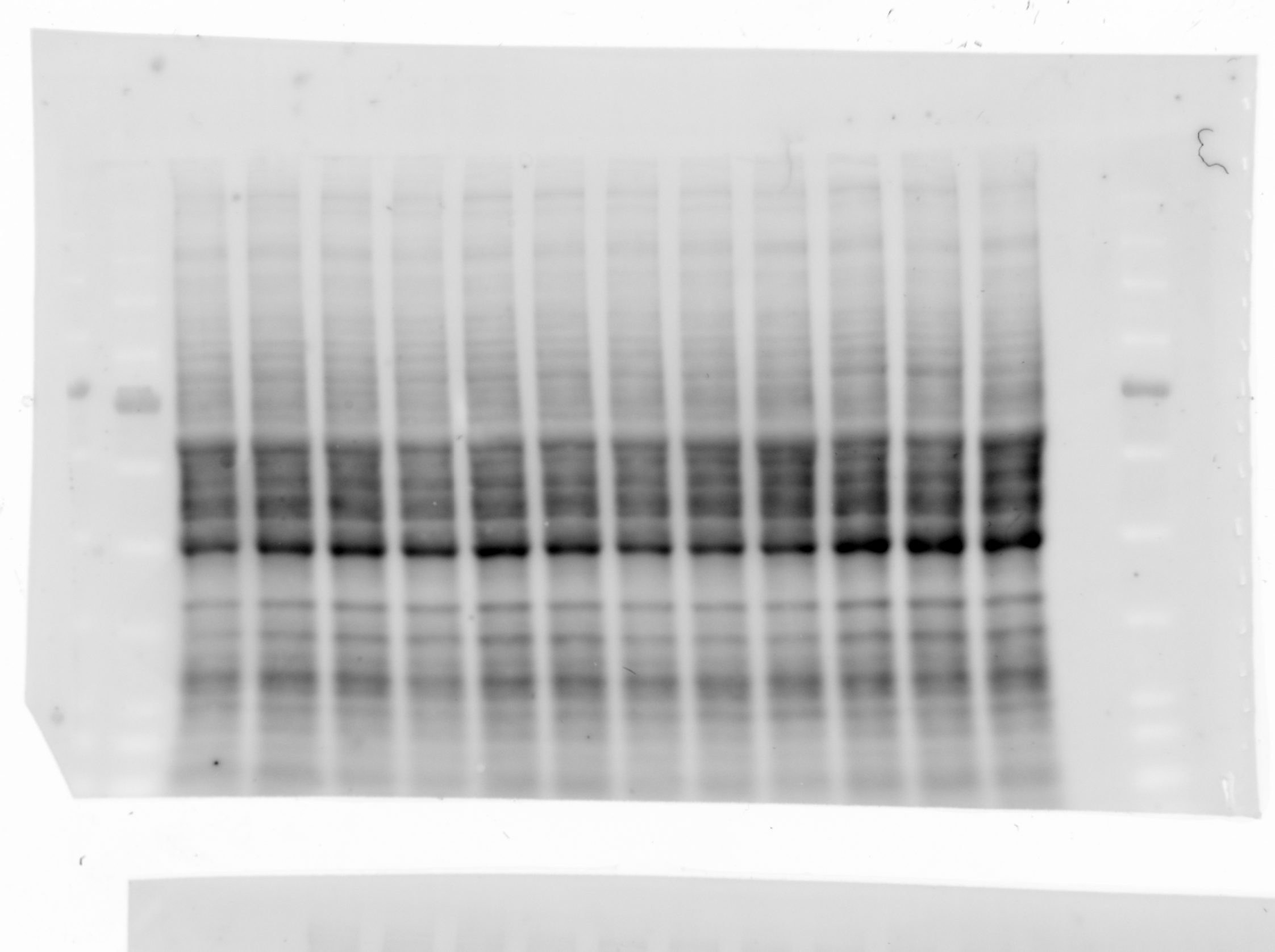

Supplement: Supplementary file 4 — Source data Fig. 2 [file 44319_2026_745_MOESM4_ESM.zip › Figure 2/2I/Replicates/Raw Data/EXP1/EXP1_Total_2020-10-24 01hr 01min g1.tif]

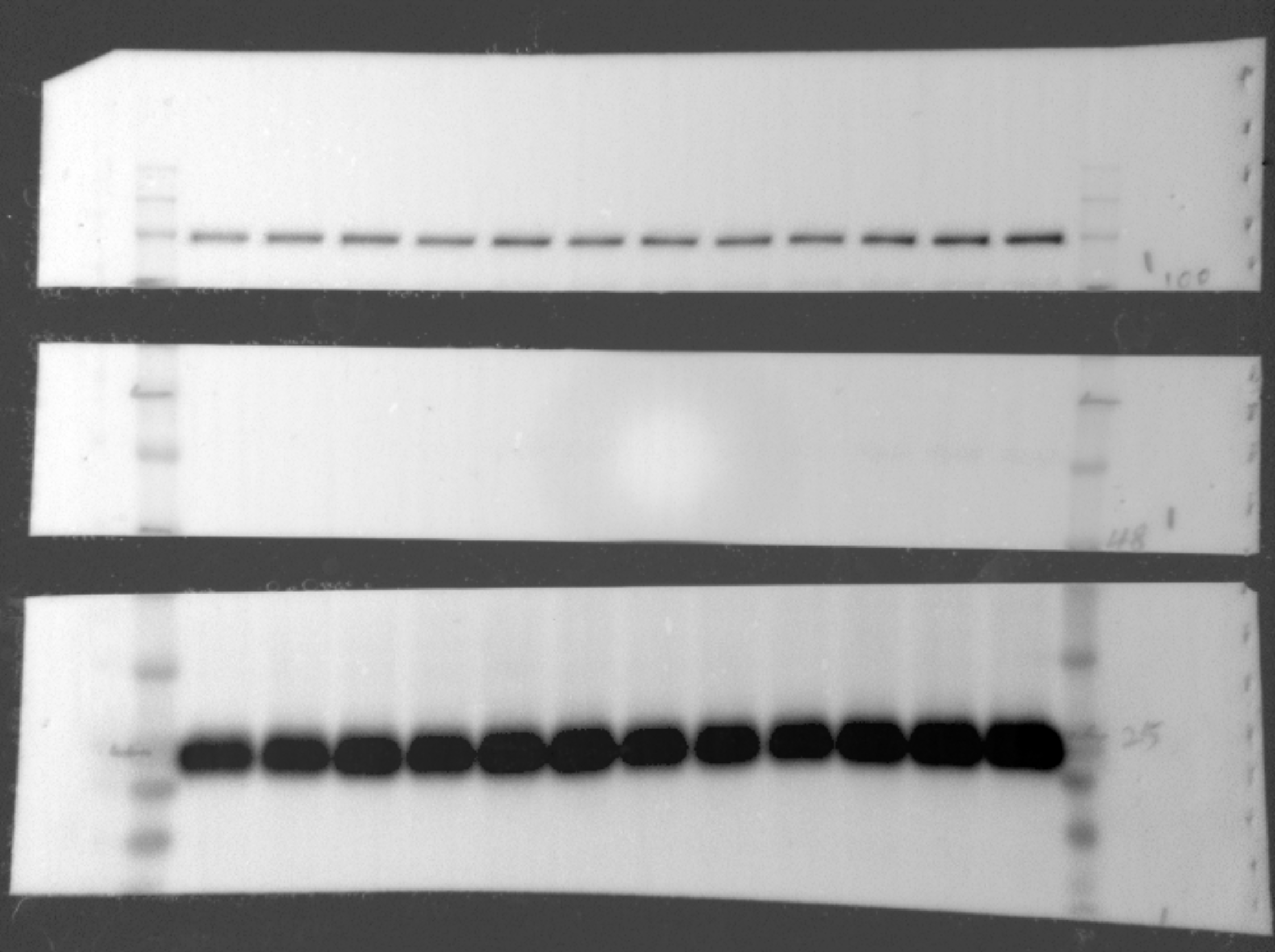

Supplement: Supplementary file 4 — Source data Fig. 2 [file 44319_2026_745_MOESM4_ESM.zip › Figure 2/2I/Replicates/Raw Data/EXP1/EXP1_Vinculin_GZA PRFcolori+v_14.3sec.tif]

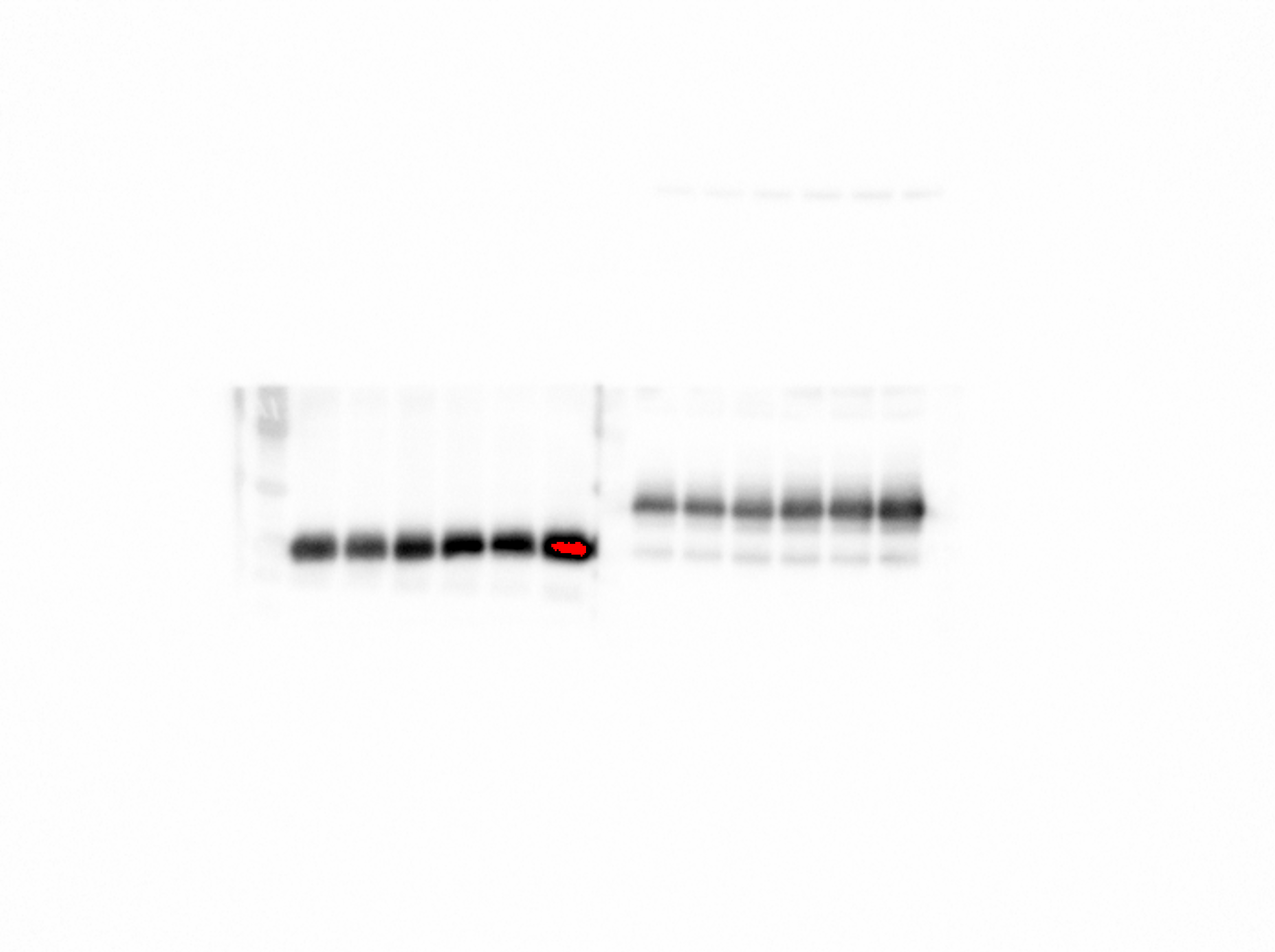

Supplement: Supplementary file 4 — Source data Fig. 2 [file 44319_2026_745_MOESM4_ESM.zip › Figure 2/2I/Replicates/Raw Data/EXP2/1.0sec.scn_gza_gzb.tif]

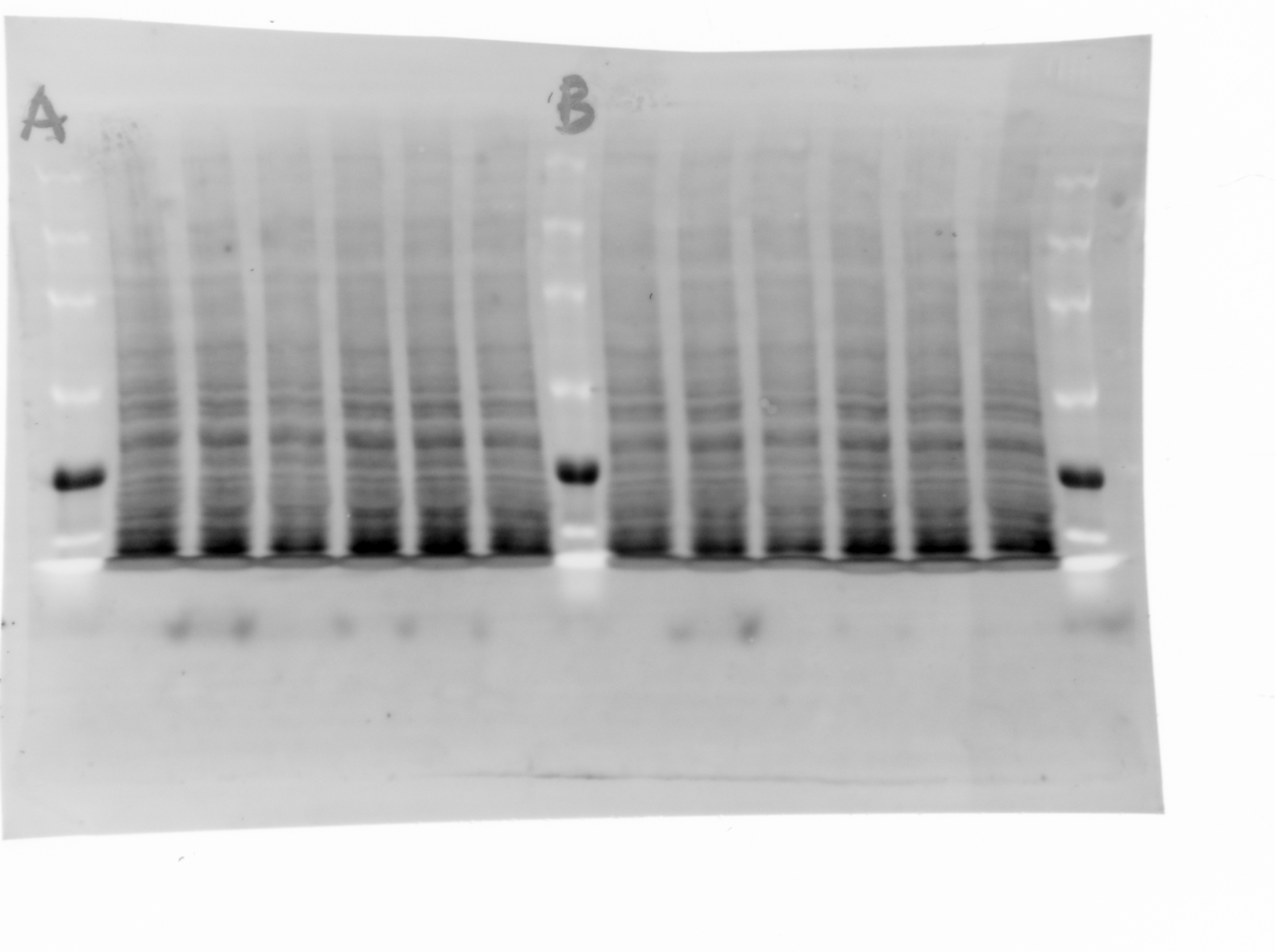

Supplement: Supplementary file 4 — Source data Fig. 2 [file 44319_2026_745_MOESM4_ESM.zip › Figure 2/2I/Replicates/Raw Data/EXP2/A+B for STAT3 Alex's.tif]

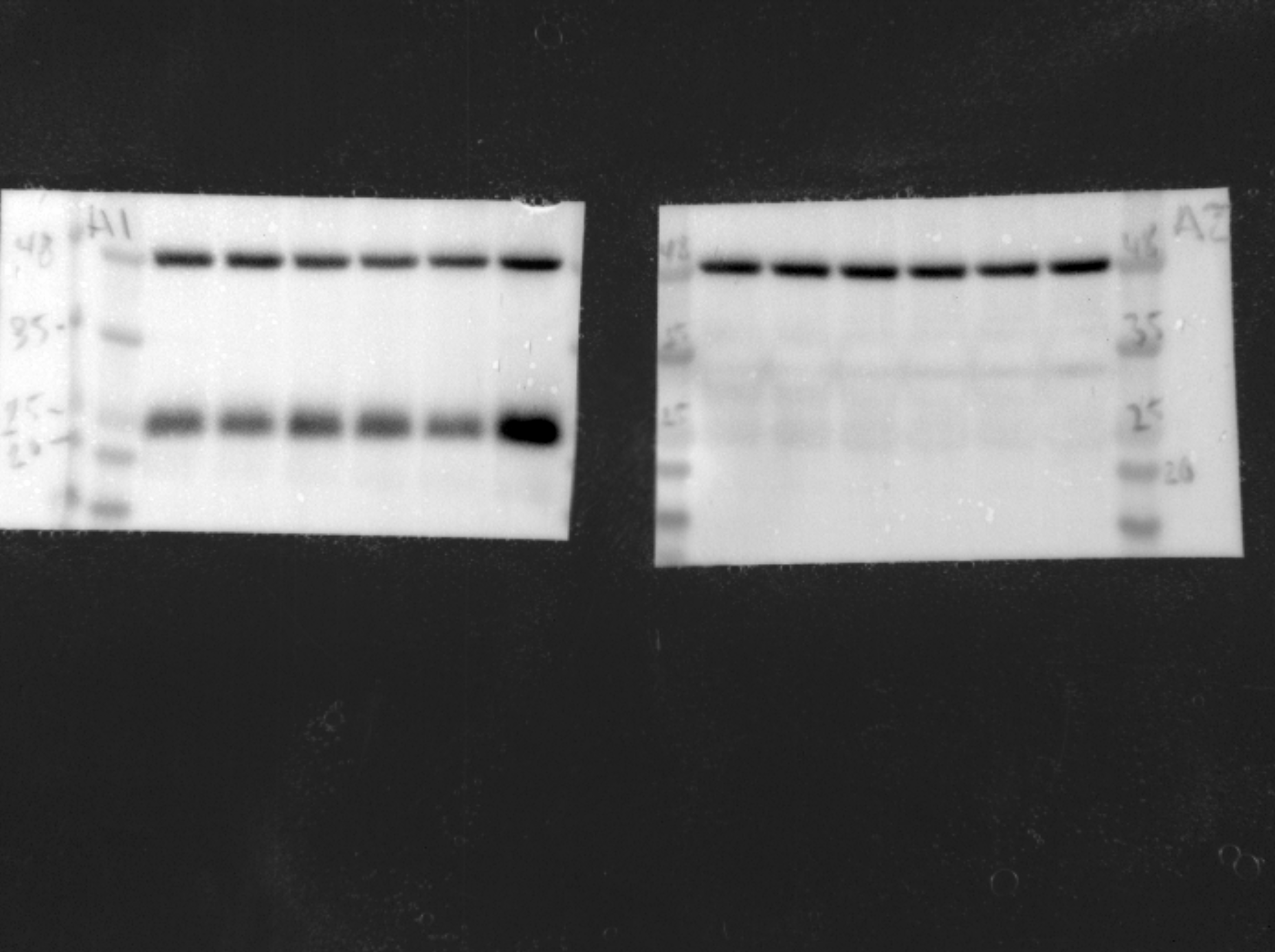

Supplement: Supplementary file 4 — Source data Fig. 2 [file 44319_2026_745_MOESM4_ESM.zip › Figure 2/2I/Replicates/Raw Data/EXP2/B-Actin.tif]

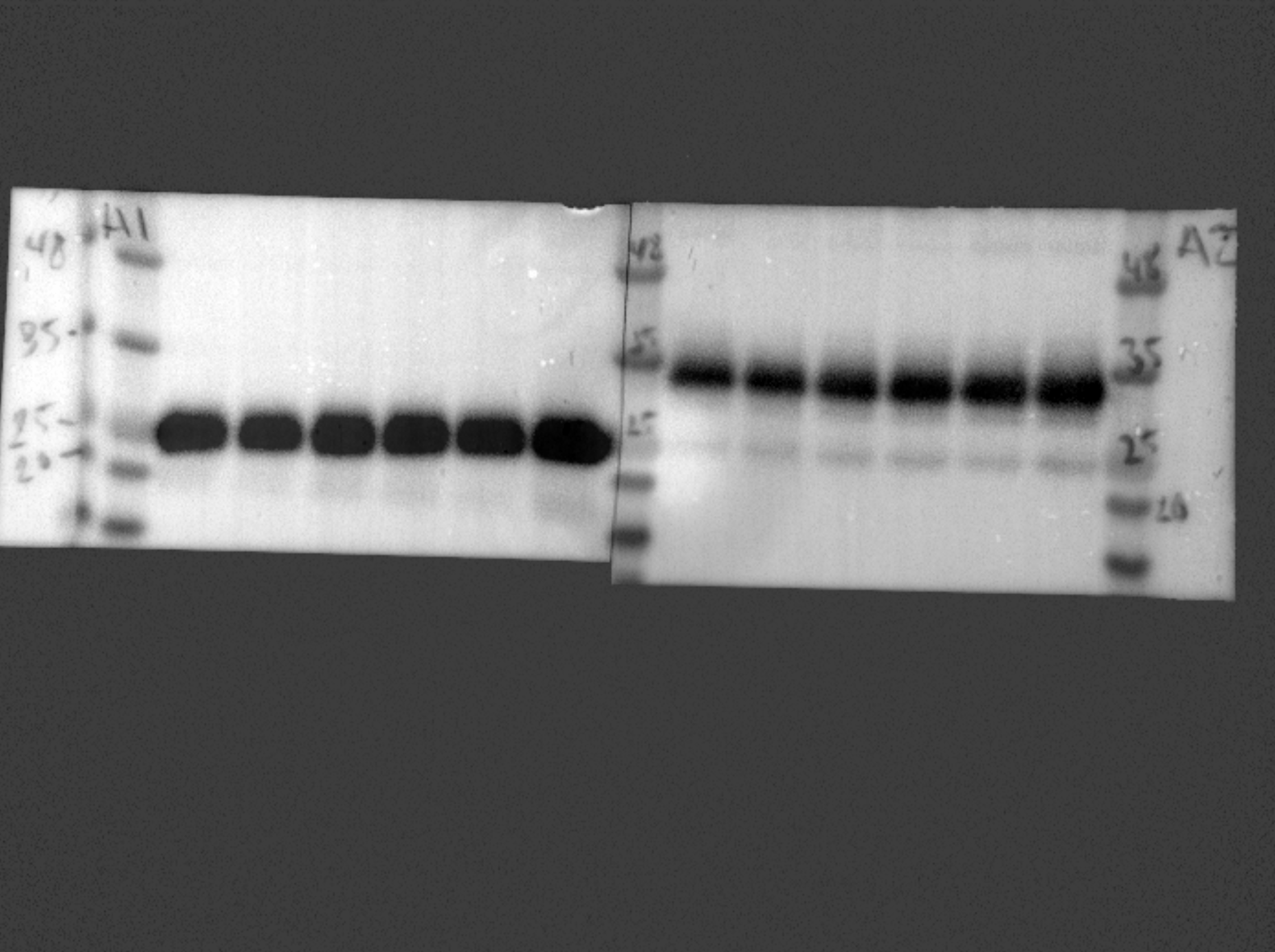

Supplement: Supplementary file 4 — Source data Fig. 2 [file 44319_2026_745_MOESM4_ESM.zip › Figure 2/2I/Replicates/Raw Data/EXP2/GZA(LEFT)_GZB(RIGHT)_0.5_SEC+COLORIMETRIC_RUN_003.tif]

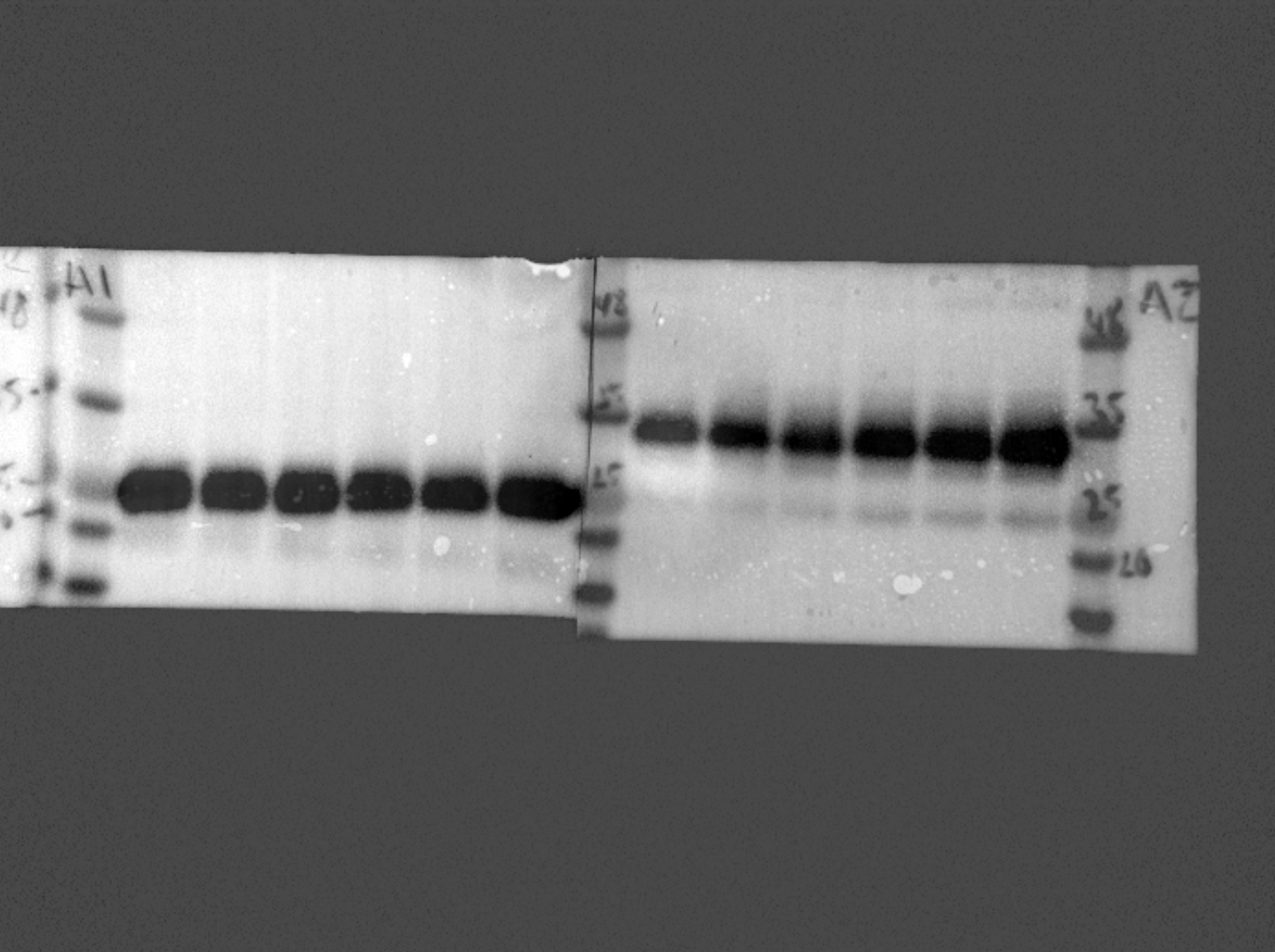

Supplement: Supplementary file 4 — Source data Fig. 2 [file 44319_2026_745_MOESM4_ESM.zip › Figure 2/2I/Replicates/Raw Data/EXP2/GZA(LEFT)_GZB(RIGHT).tif]

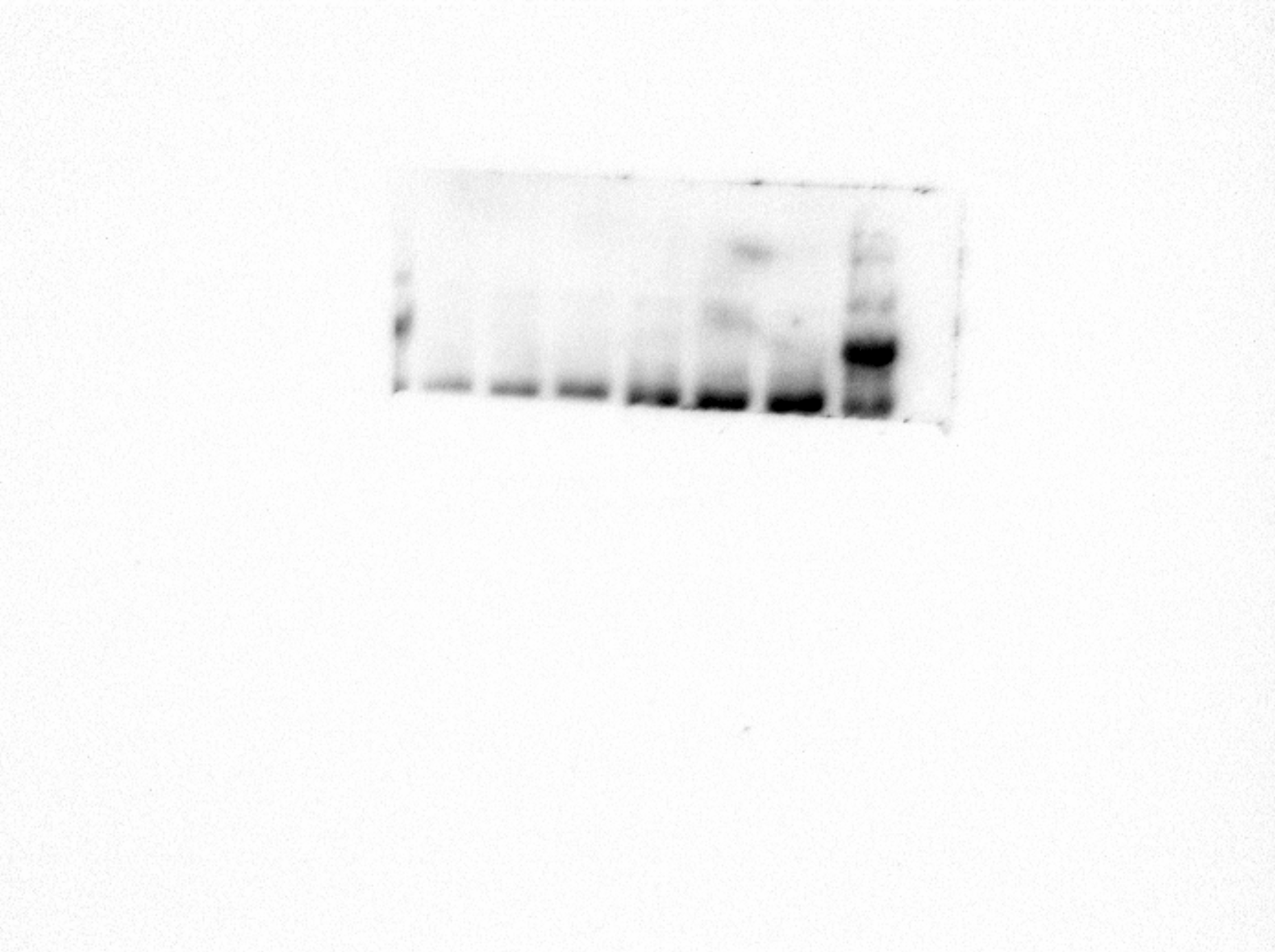

Supplement: Supplementary file 4 — Source data Fig. 2 [file 44319_2026_745_MOESM4_ESM.zip › Figure 2/2I/Replicates/Raw Data/EXP2/Perforin 2022-03-24 15hr 13min_Exposure_25.2sec.scn_prfo.tif]

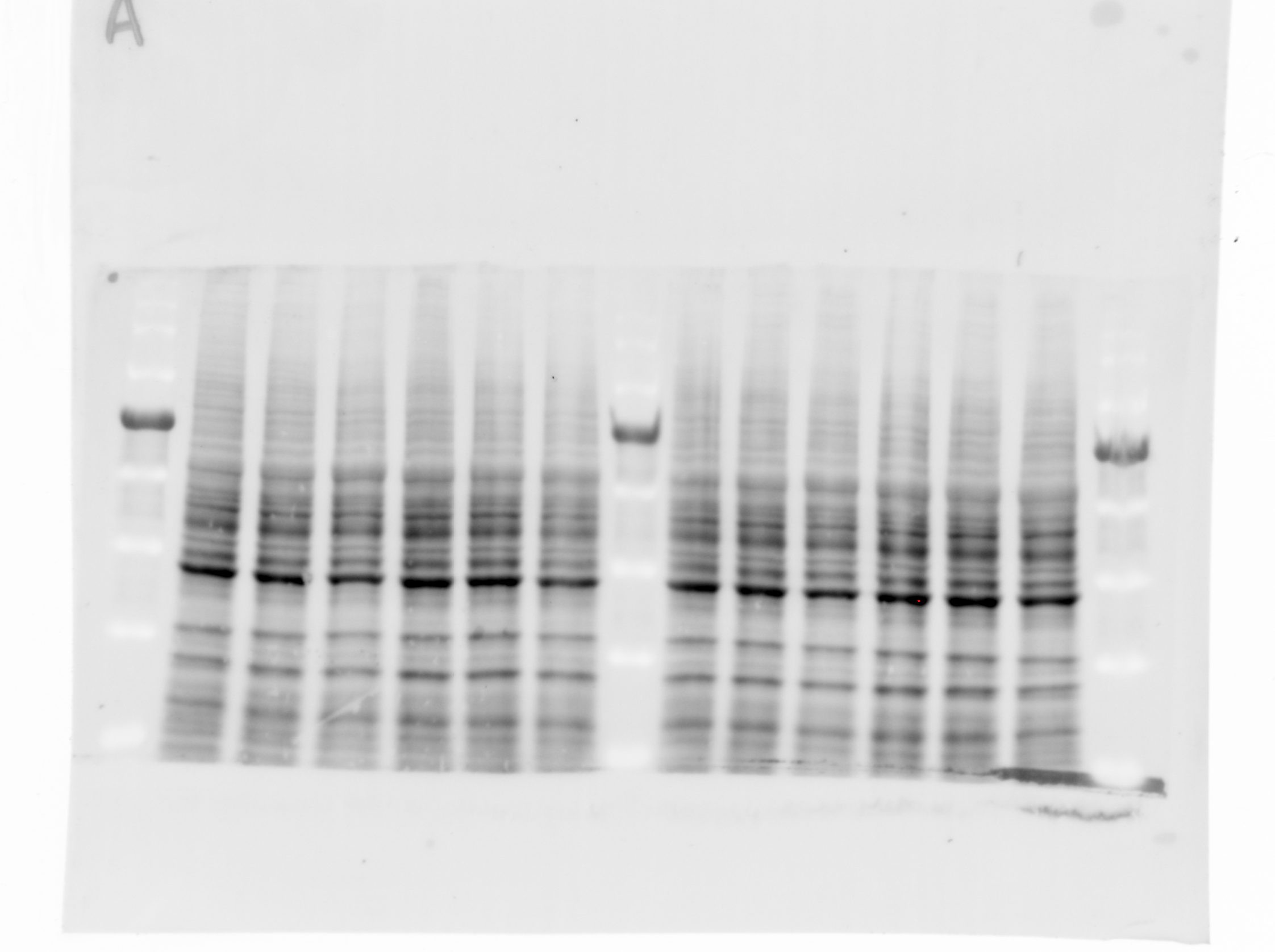

Supplement: Supplementary file 4 — Source data Fig. 2 [file 44319_2026_745_MOESM4_ESM.zip › Figure 2/2I/Replicates/Raw Data/EXP2/Perforin_TOTAL_PROTEIN.tif]

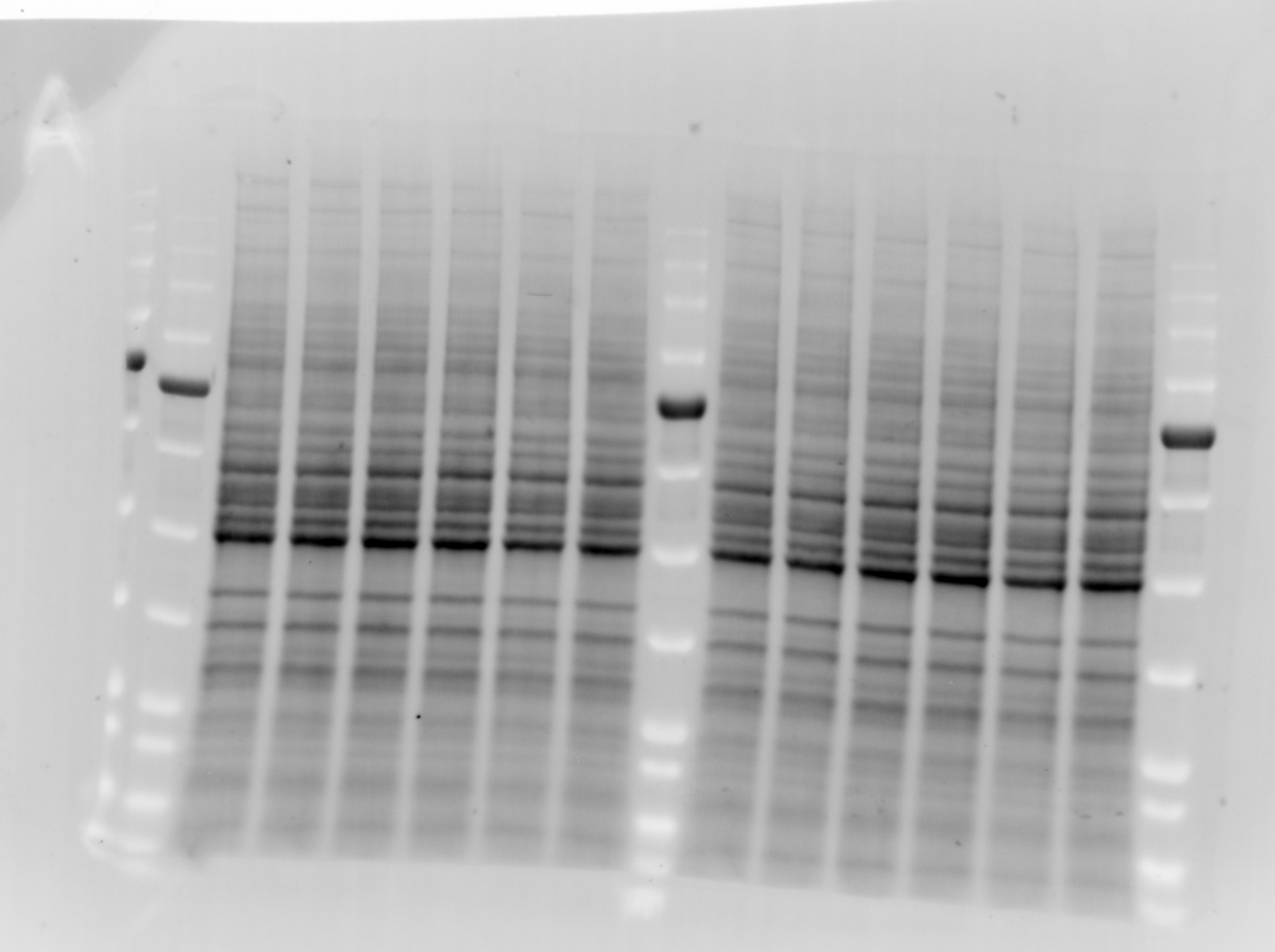

Supplement: Supplementary file 4 — Source data Fig. 2 [file 44319_2026_745_MOESM4_ESM.zip › Figure 2/2I/Replicates/Raw Data/EXP2/Total Protein STAT4 GA_LEFT_STAT5 GB_RIGHT.tif]

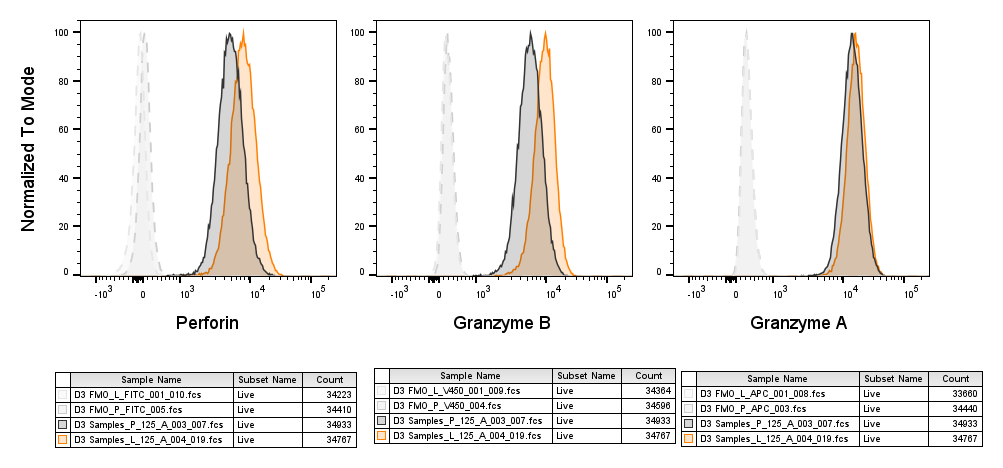

Supplement: Supplementary file 4 — Source data Fig. 2 [file 44319_2026_745_MOESM4_ESM.zip › Figure 2/2J/2J.tiff]

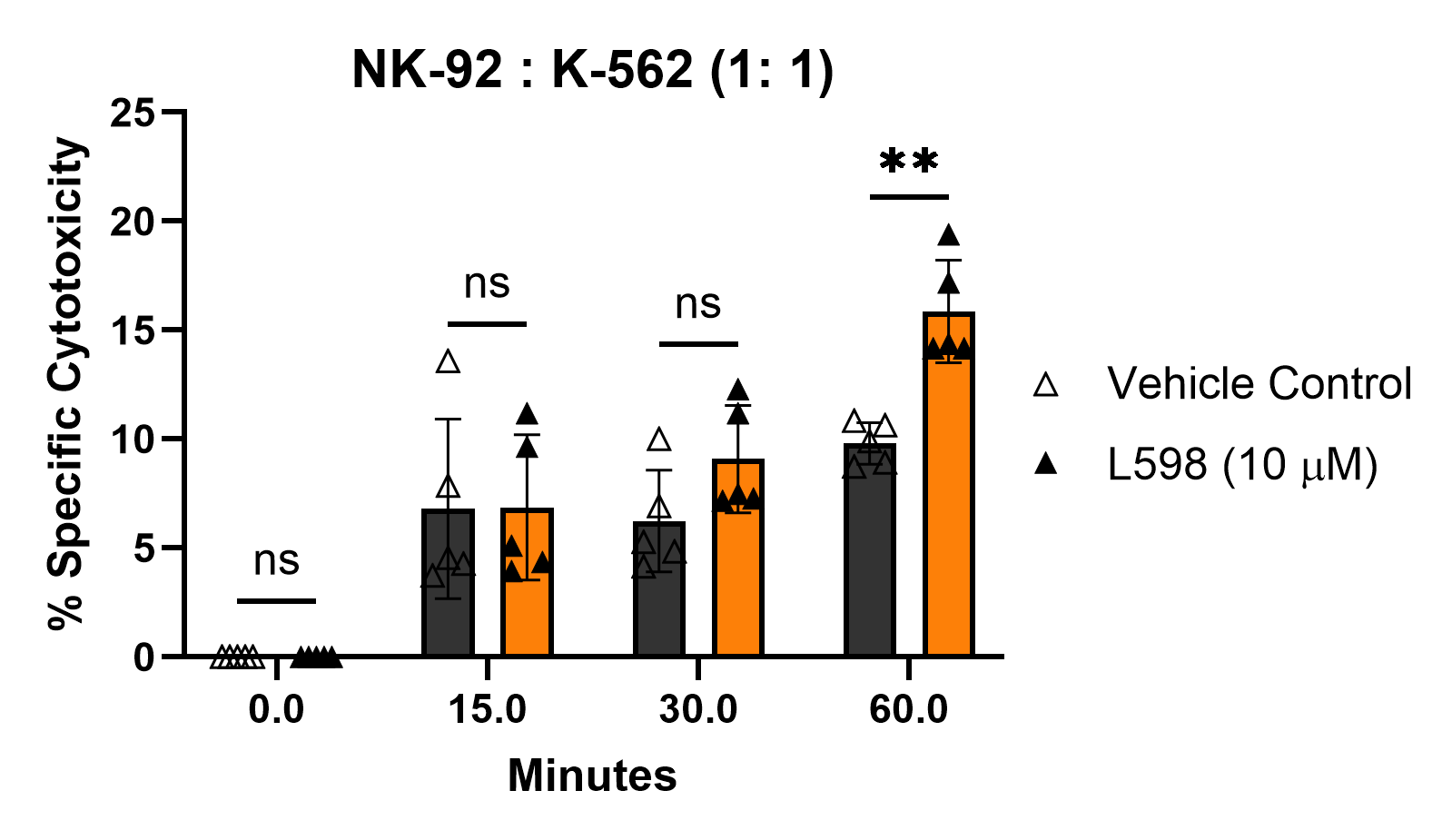

Supplement: Supplementary file 5 — Source data Fig. 3 [file 44319_2026_745_MOESM5_ESM.zip › Figure 3/3A/3A.tif]

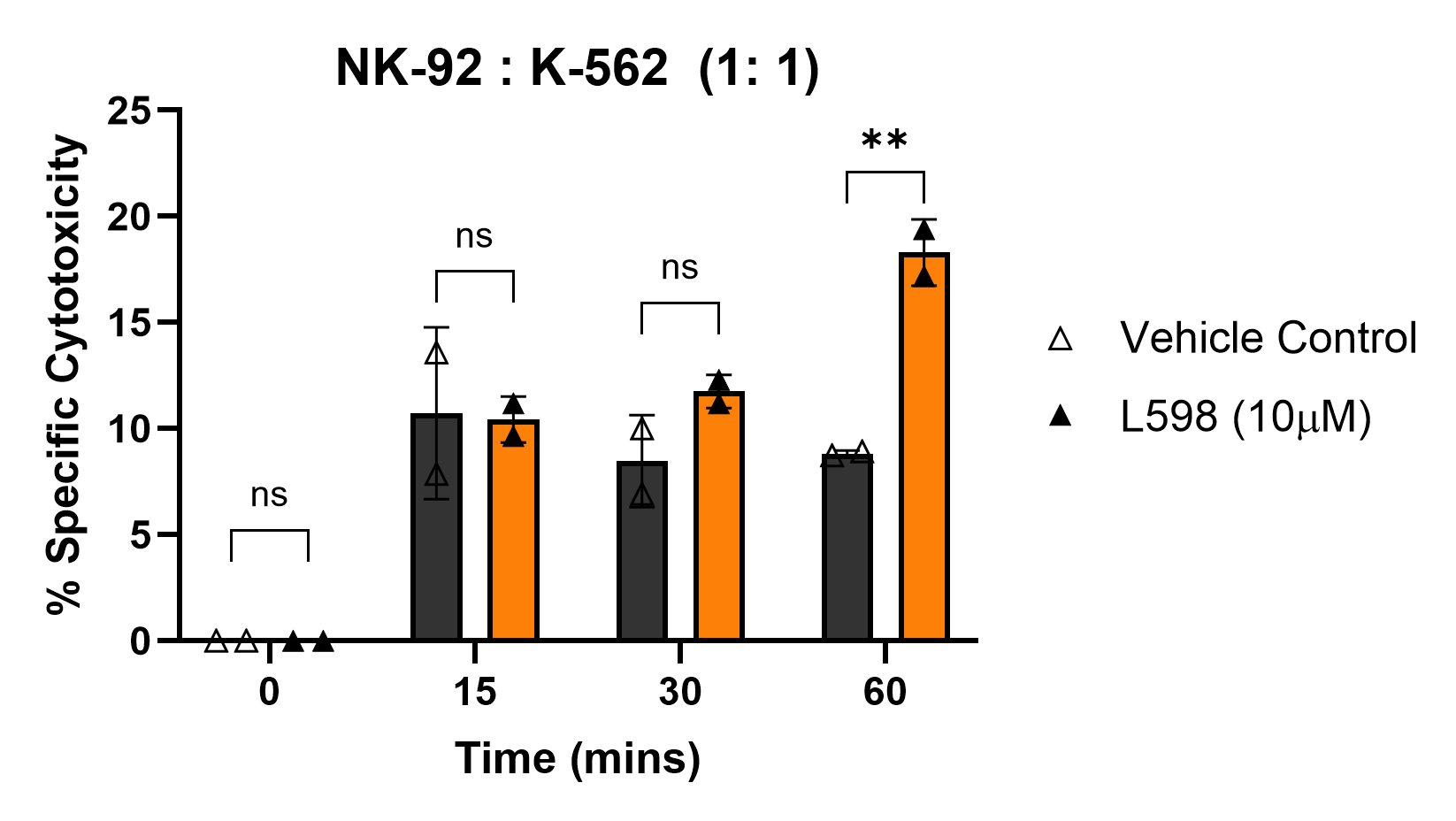

Supplement: Supplementary file 5 — Source data Fig. 3 [file 44319_2026_745_MOESM5_ESM.zip › Figure 3/3A/Replicates/3A_Exp2.tif]

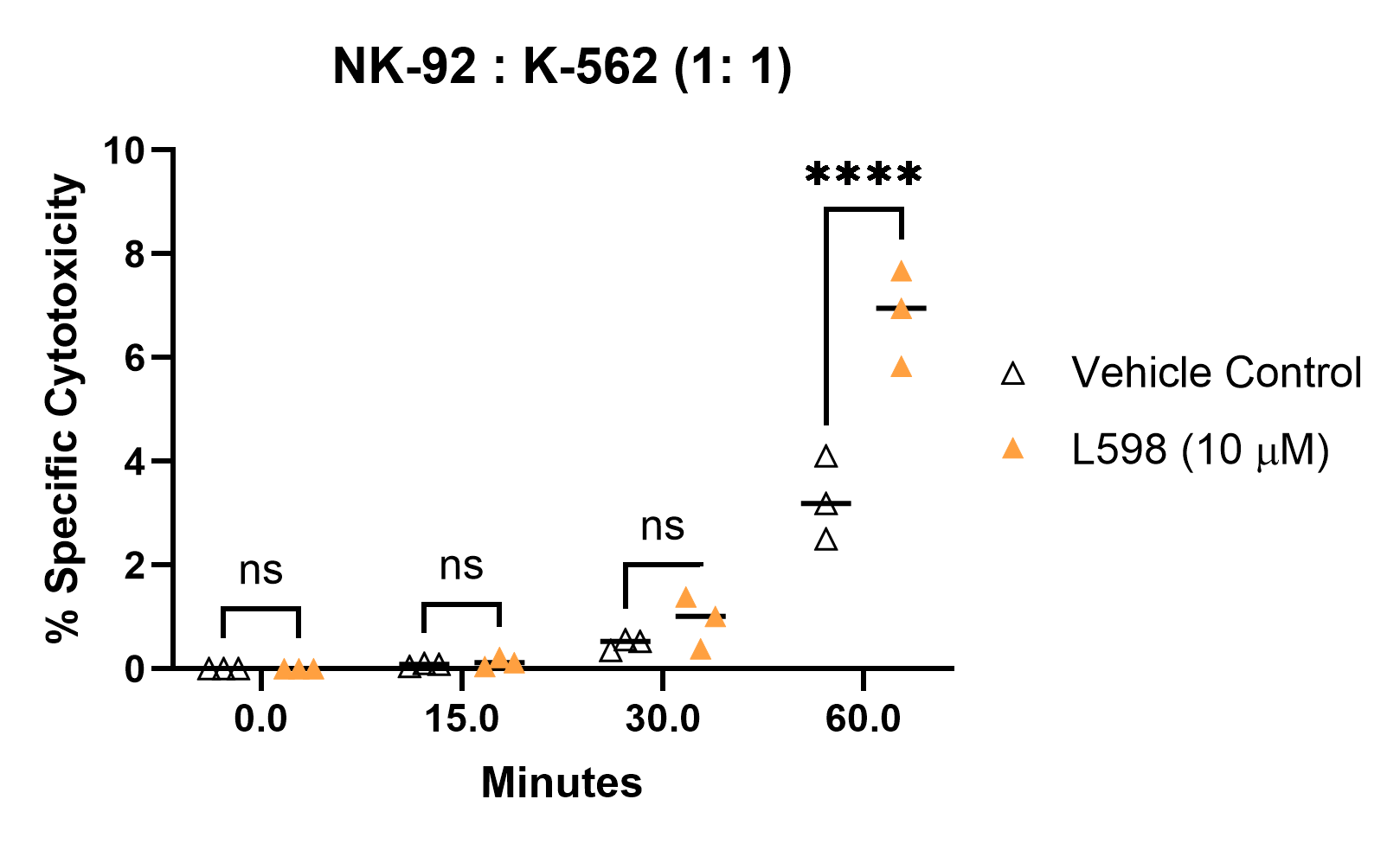

Supplement: Supplementary file 5 — Source data Fig. 3 [file 44319_2026_745_MOESM5_ESM.zip › Figure 3/3A/Replicates/3A_Exp3.tif]

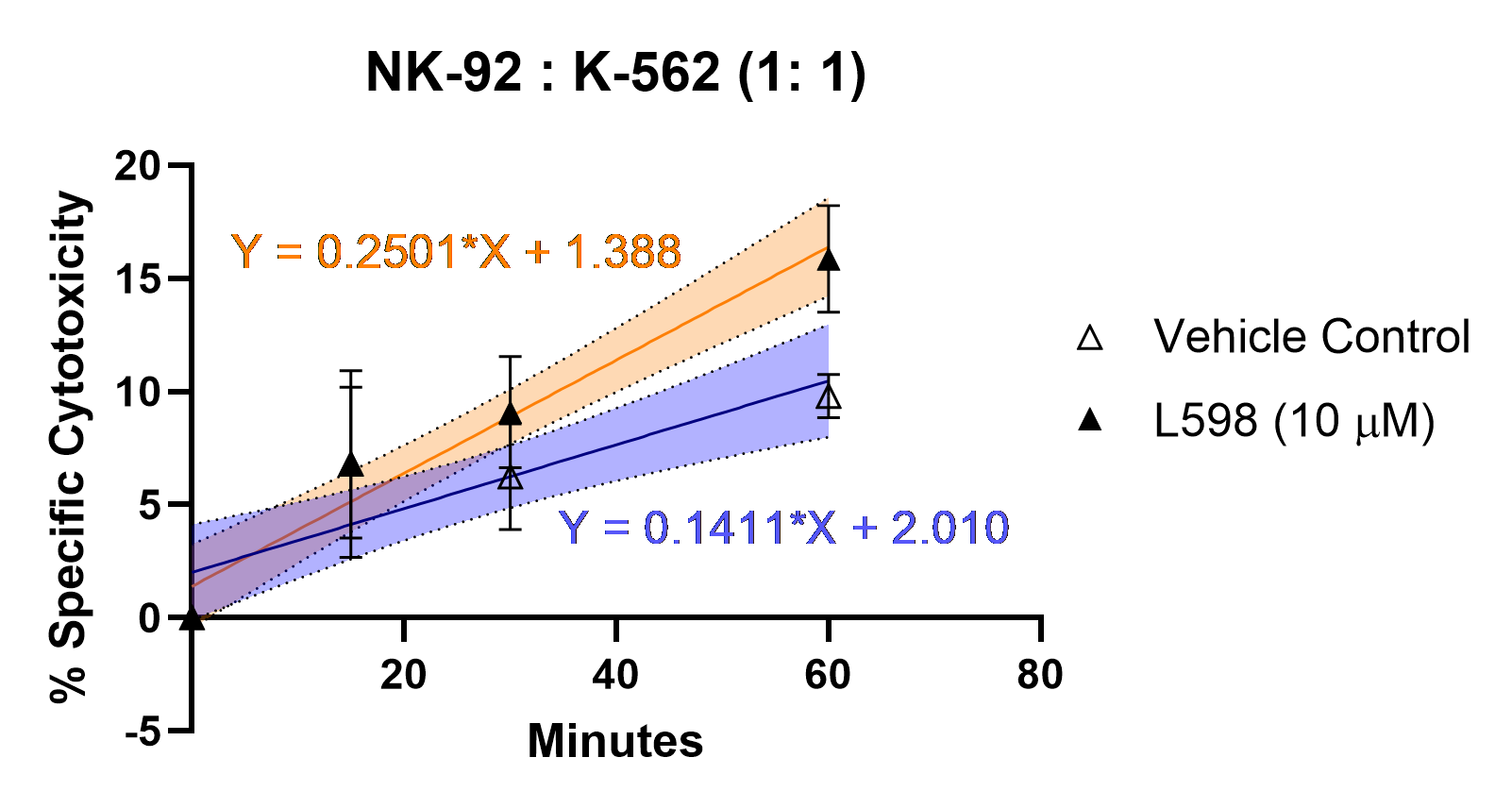

Supplement: Supplementary file 5 — Source data Fig. 3 [file 44319_2026_745_MOESM5_ESM.zip › Figure 3/3B/3B_Combined 2 experiments K562 specific killing kinetics.tif]

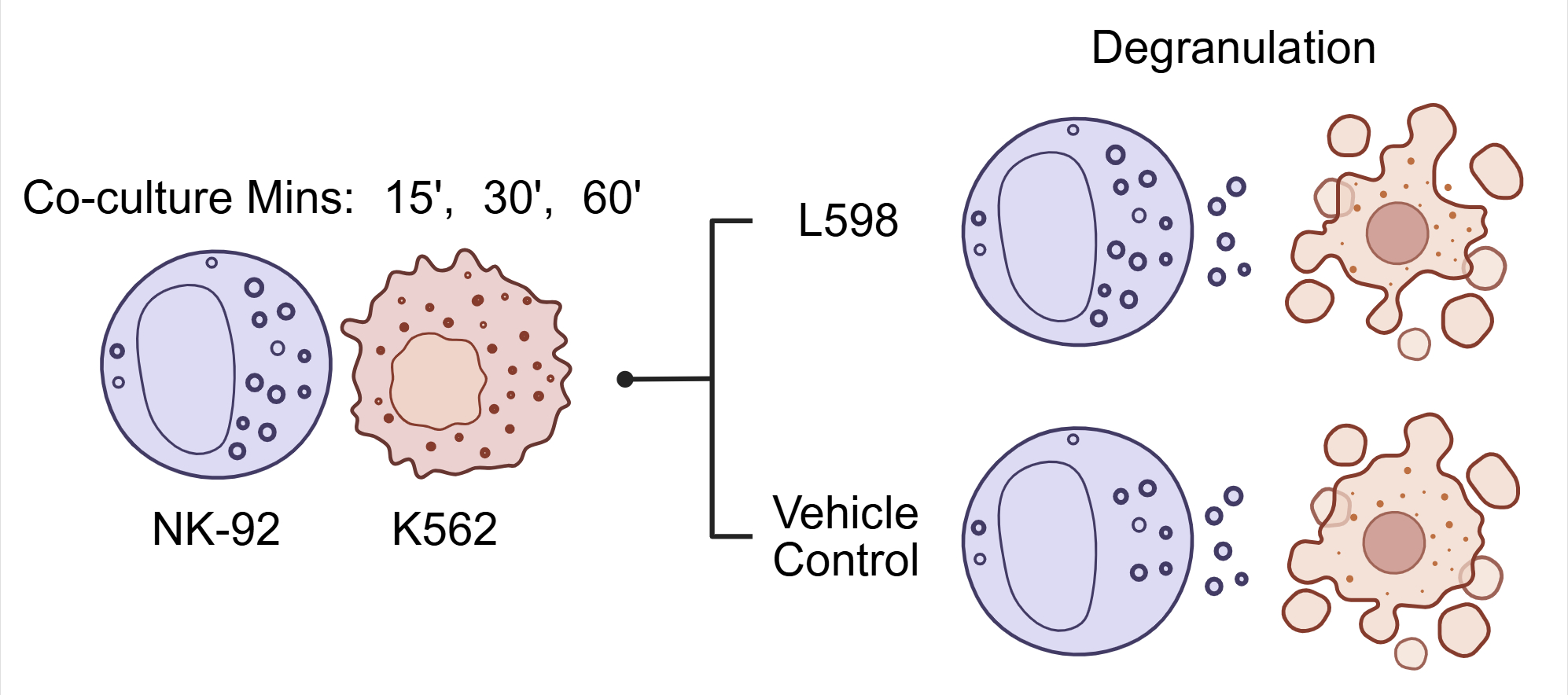

Supplement: Supplementary file 5 — Source data Fig. 3 [file 44319_2026_745_MOESM5_ESM.zip › Figure 3/3C/3C.jpeg]

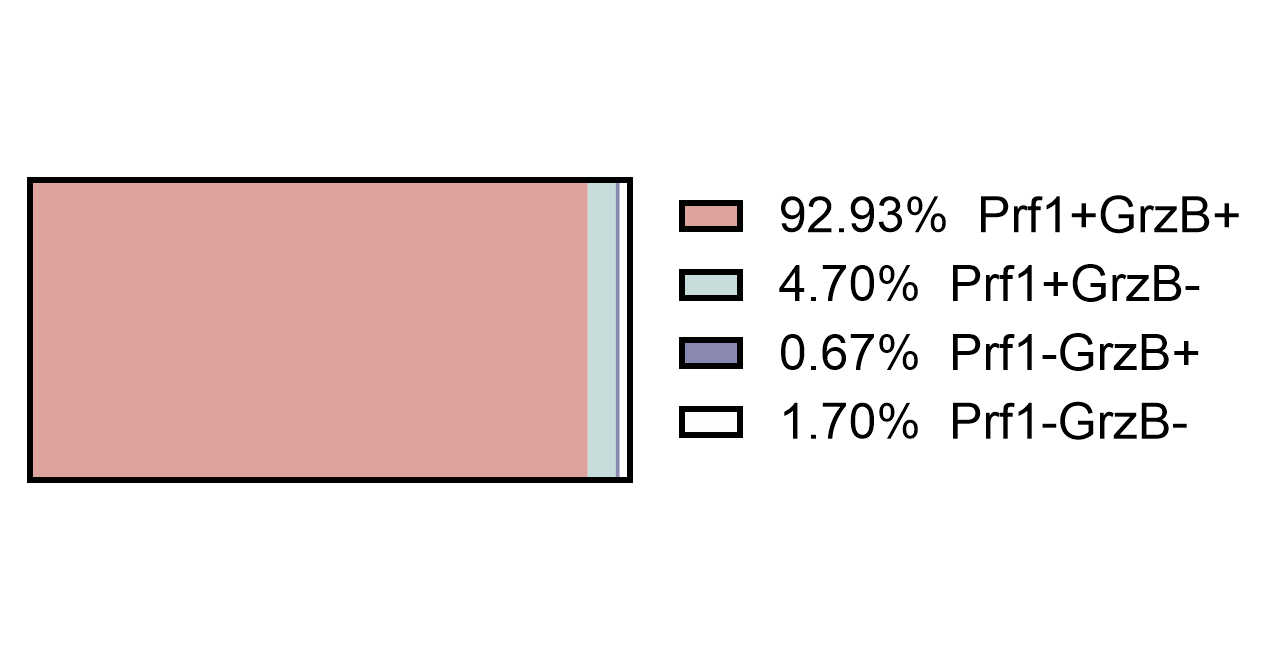

Supplement: Supplementary file 5 — Source data Fig. 3 [file 44319_2026_745_MOESM5_ESM.zip › Figure 3/3D/3D_Combined 3 Exp PBS_15'(K562).tif]

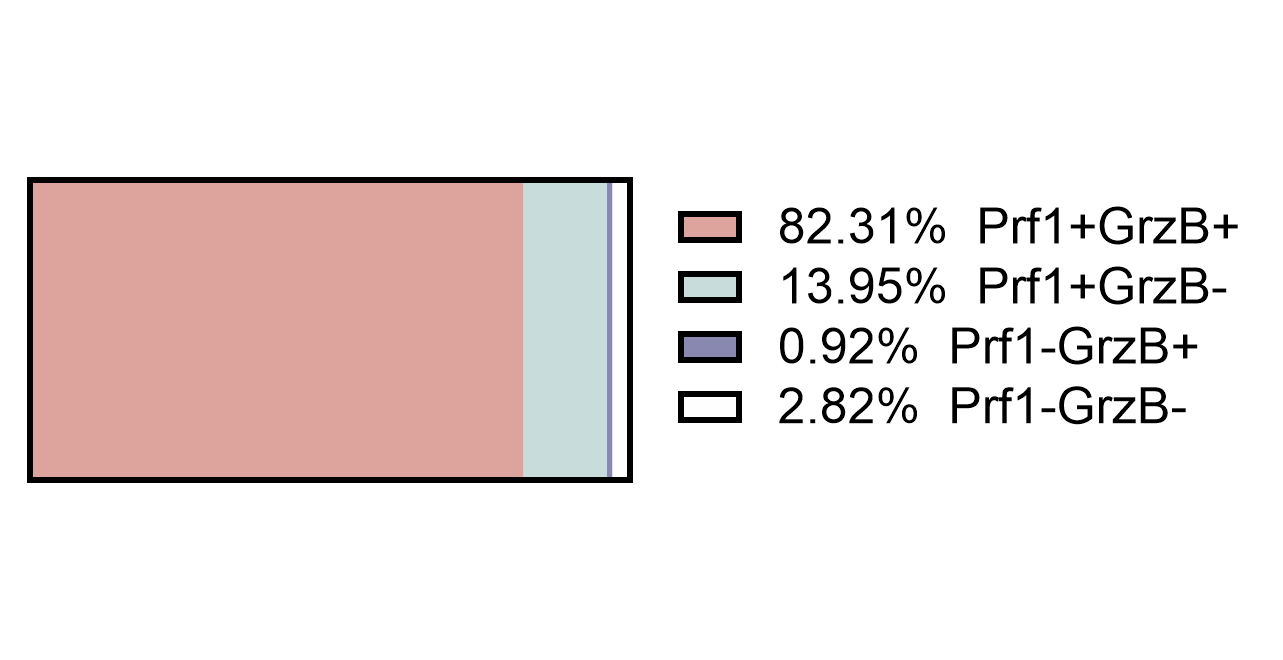

Supplement: Supplementary file 5 — Source data Fig. 3 [file 44319_2026_745_MOESM5_ESM.zip › Figure 3/3D/3D_Combined 3 Exp PP_PBS_60' (K562).tif]

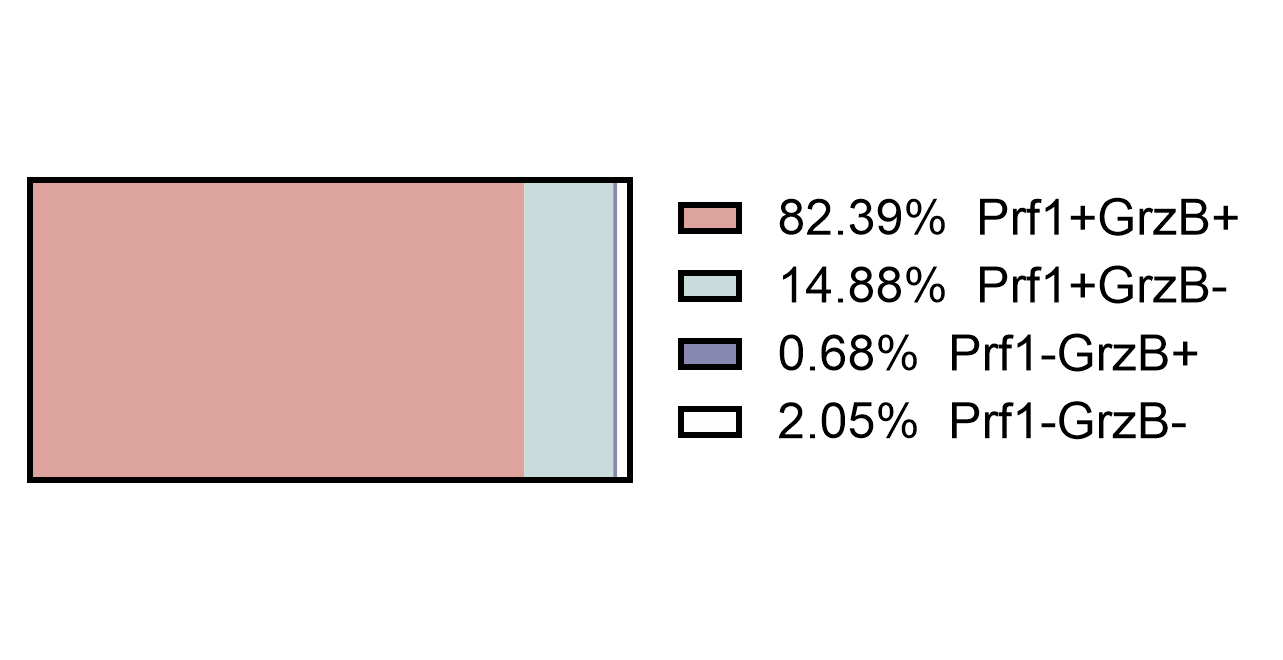

Supplement: Supplementary file 5 — Source data Fig. 3 [file 44319_2026_745_MOESM5_ESM.zip › Figure 3/3D/3D_Combined 3 Exp_PP_PBS_30'(K562).tif]

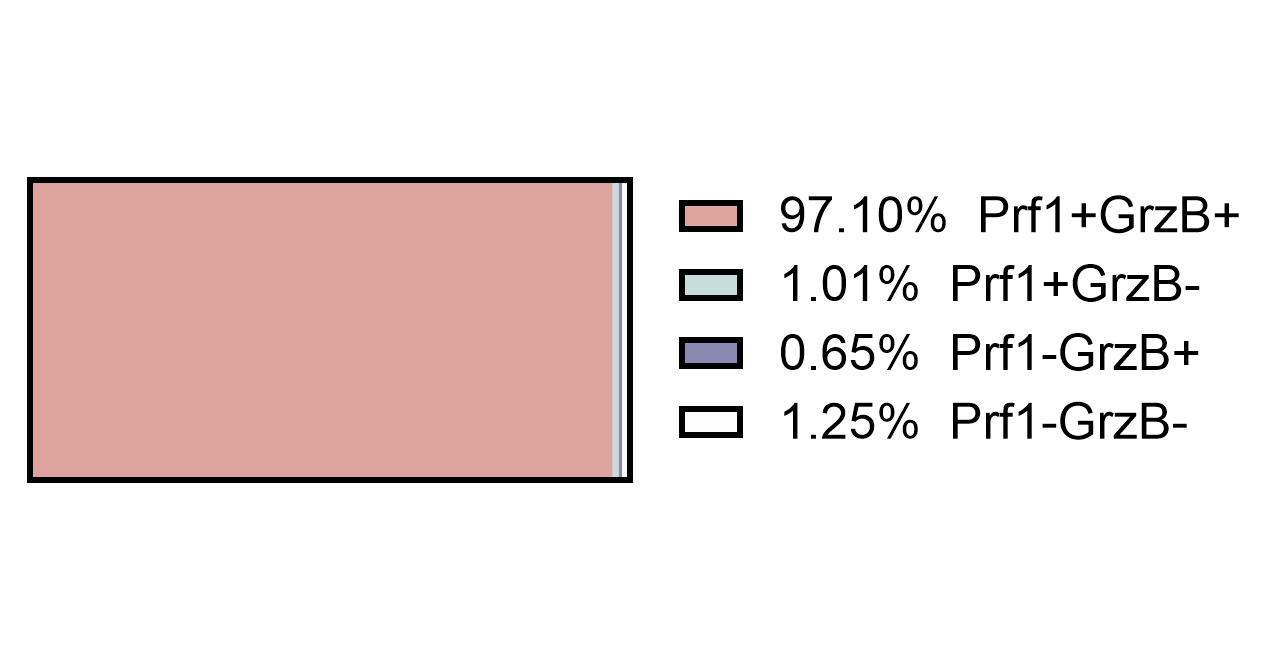

Supplement: Supplementary file 5 — Source data Fig. 3 [file 44319_2026_745_MOESM5_ESM.zip › Figure 3/3D/3D_Combined 3 Exp PP_L598_15'(K562).tif]

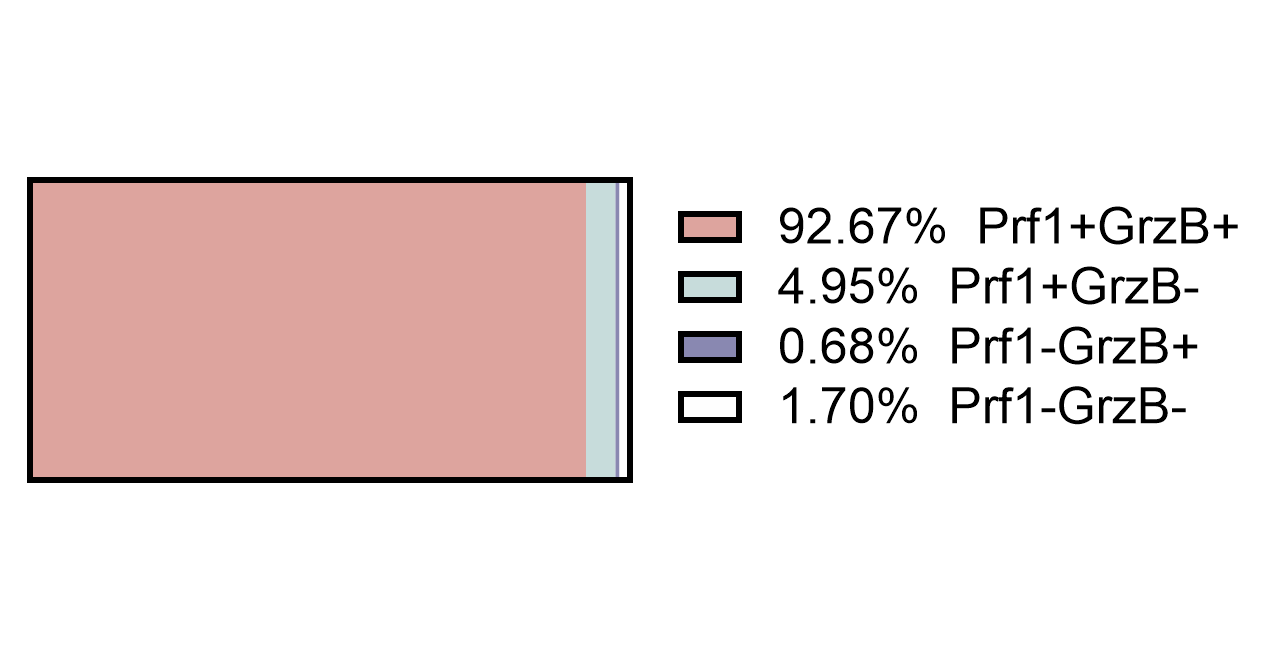

Supplement: Supplementary file 5 — Source data Fig. 3 [file 44319_2026_745_MOESM5_ESM.zip › Figure 3/3D/3D_Combined 3 Exp PP_L598_60'(K562).tif]

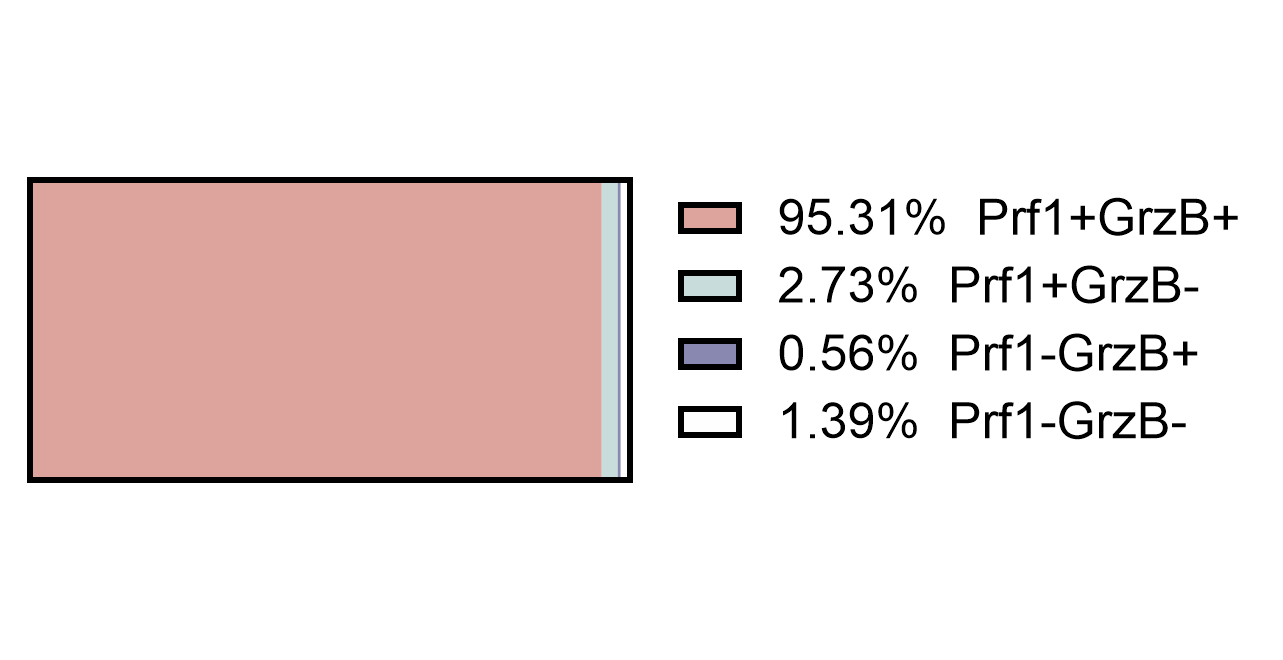

Supplement: Supplementary file 5 — Source data Fig. 3 [file 44319_2026_745_MOESM5_ESM.zip › Figure 3/3D/3D_Combined 3 Exp PP_L598_30'(K562).tif]

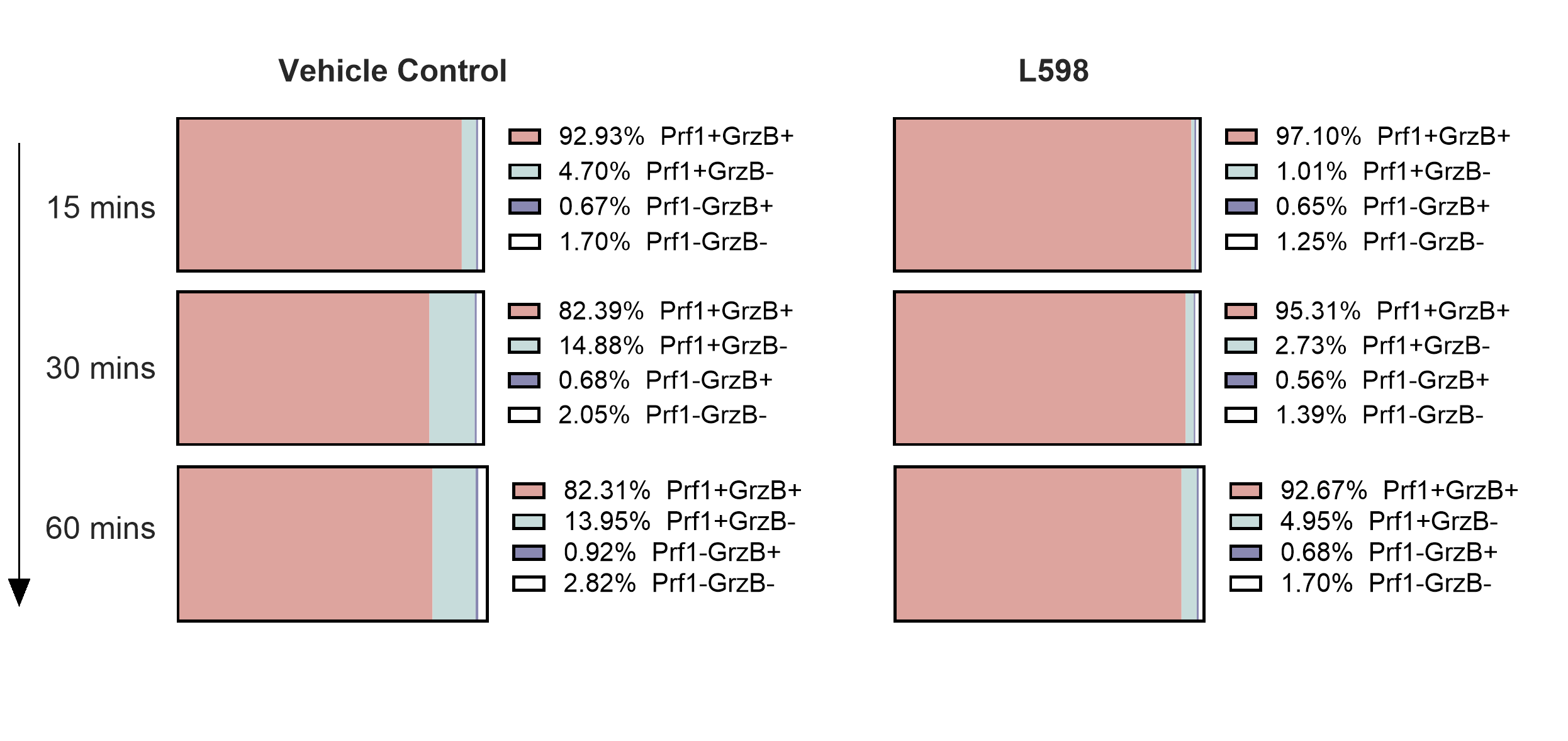

Supplement: Supplementary file 5 — Source data Fig. 3 [file 44319_2026_745_MOESM5_ESM.zip › Figure 3/3D/3D.tif]

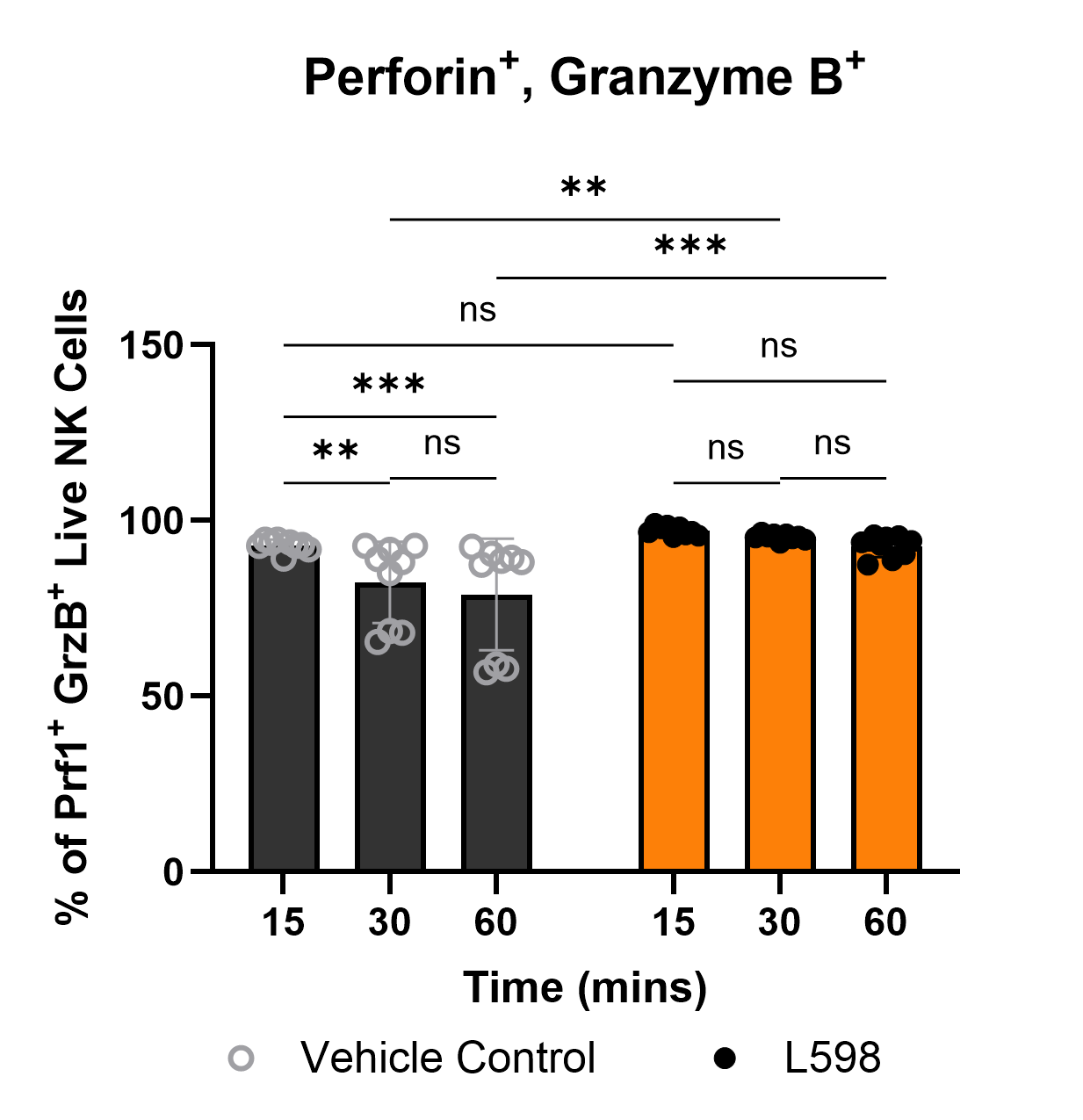

Supplement: Supplementary file 5 — Source data Fig. 3 [file 44319_2026_745_MOESM5_ESM.zip › Figure 3/3E/3E Combined 3 Experiments.tif]

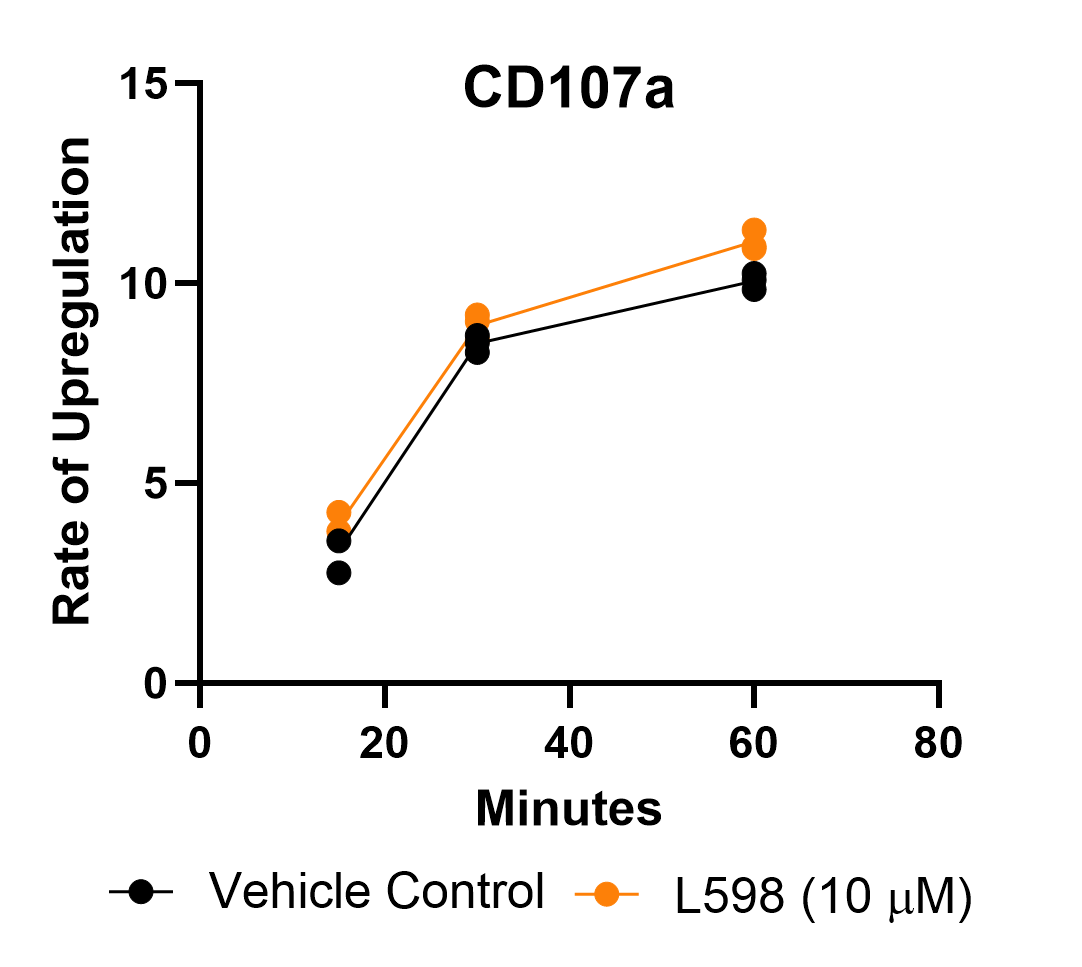

Supplement: Supplementary file 5 — Source data Fig. 3 [file 44319_2026_745_MOESM5_ESM.zip › Figure 3/3F/3F.tif]

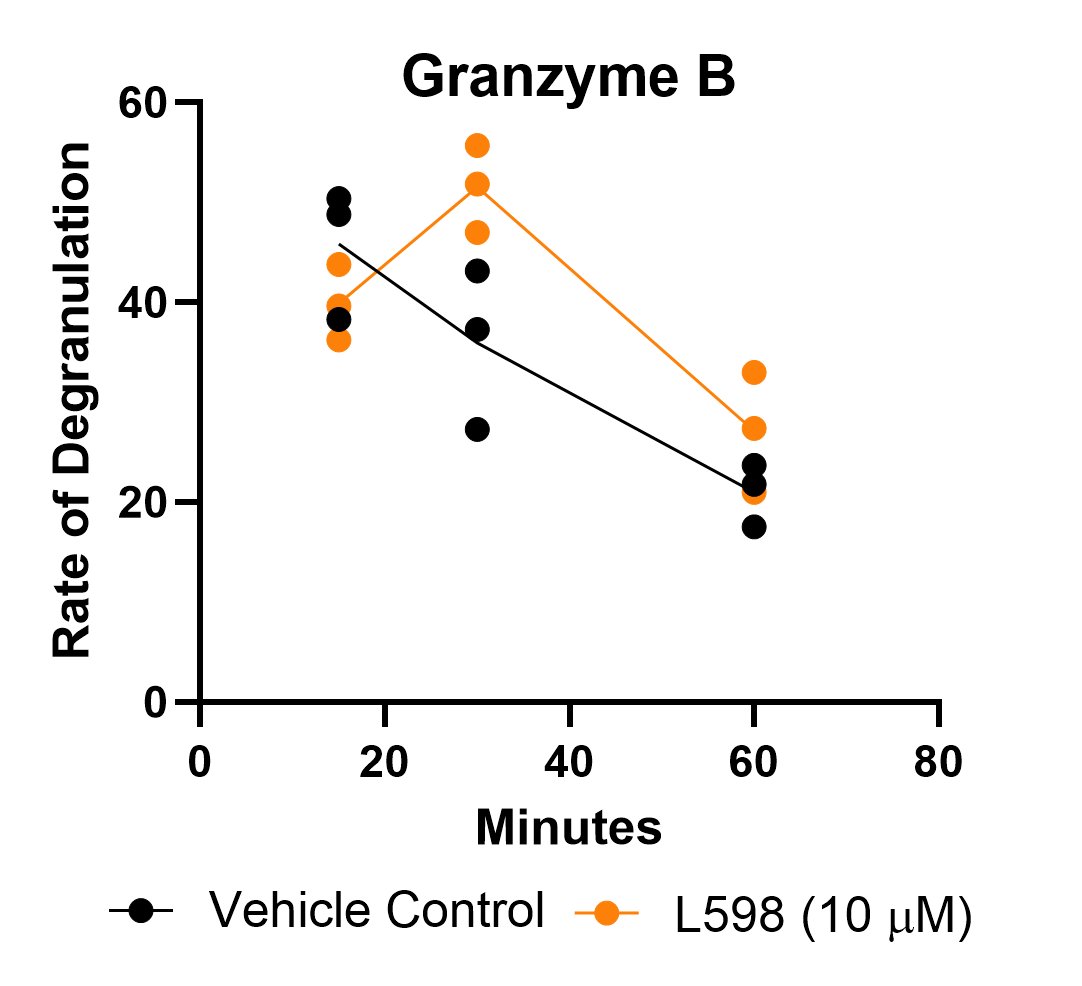

Supplement: Supplementary file 5 — Source data Fig. 3 [file 44319_2026_745_MOESM5_ESM.zip › Figure 3/3G/3G.tif]

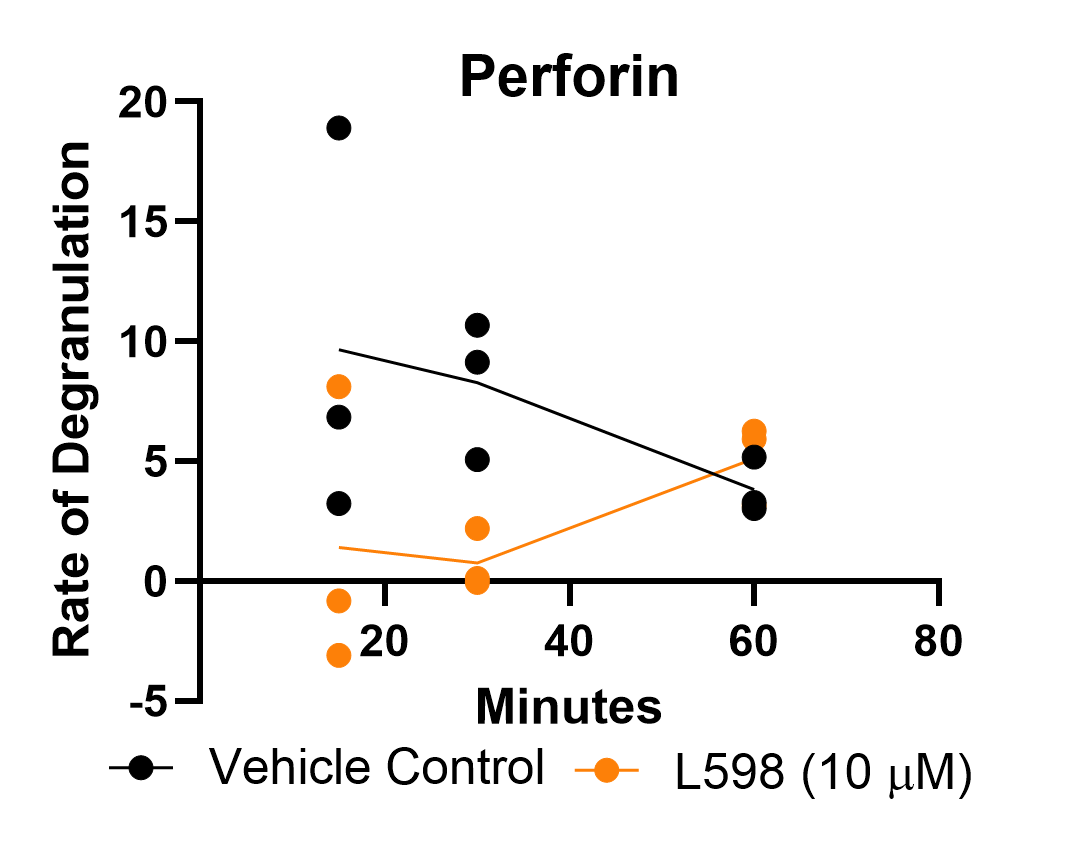

Supplement: Supplementary file 5 — Source data Fig. 3 [file 44319_2026_745_MOESM5_ESM.zip › Figure 3/3H/3H.tif]

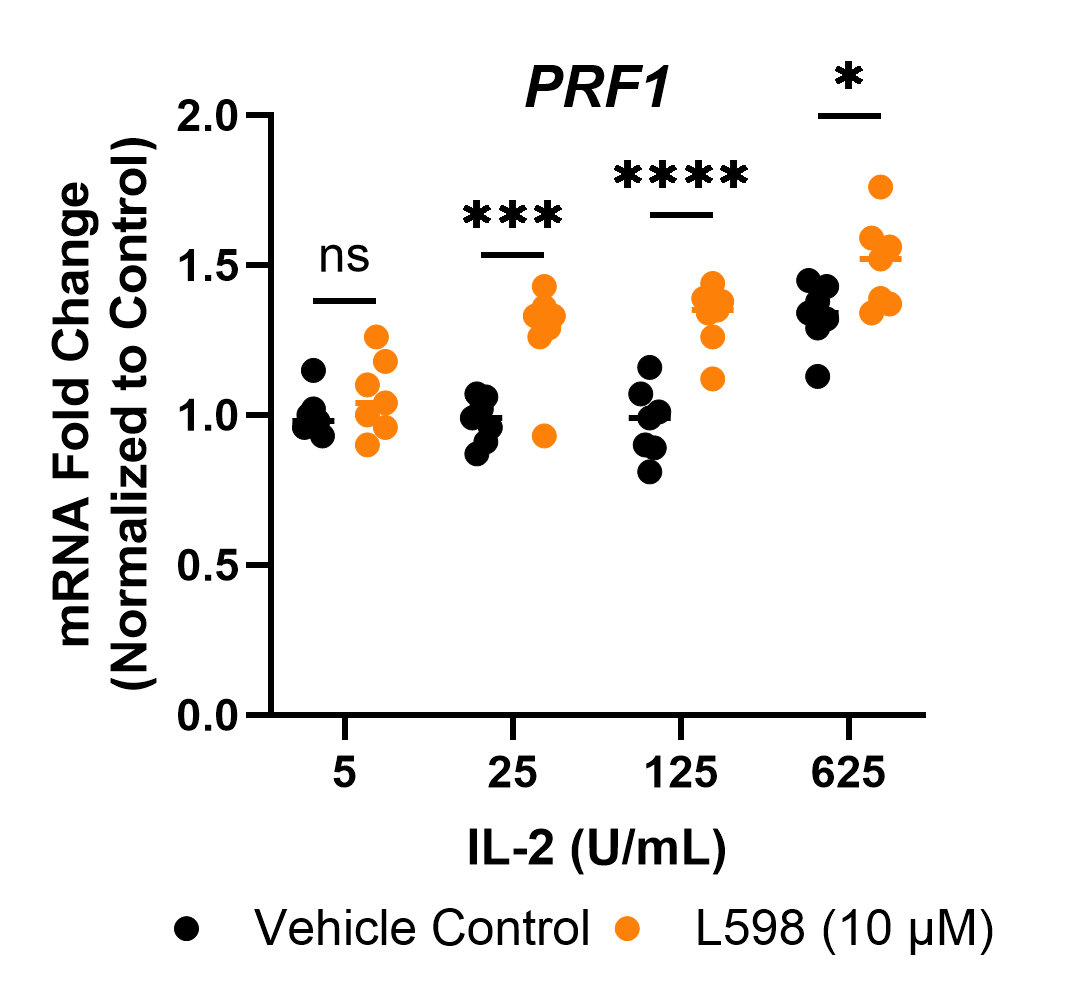

Supplement: Supplementary file 6 — Source data Fig. 4 [file 44319_2026_745_MOESM6_ESM.zip › Figure 4/4D/4D_Scatter of PRF1_Combined 2 Experiments_XY.tif]

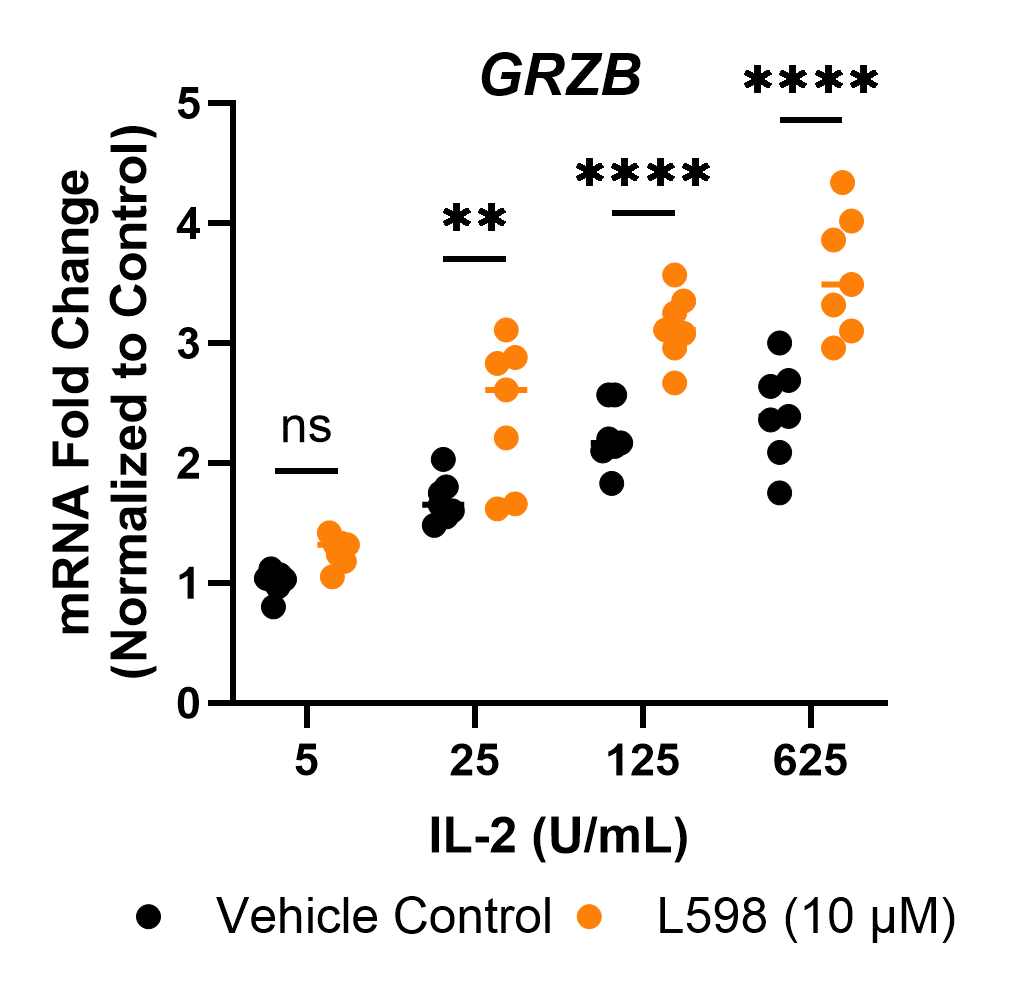

Supplement: Supplementary file 6 — Source data Fig. 4 [file 44319_2026_745_MOESM6_ESM.zip › Figure 4/4C/4C_Scatter of GRZB_Combined 2 Experiments_XY.tif]

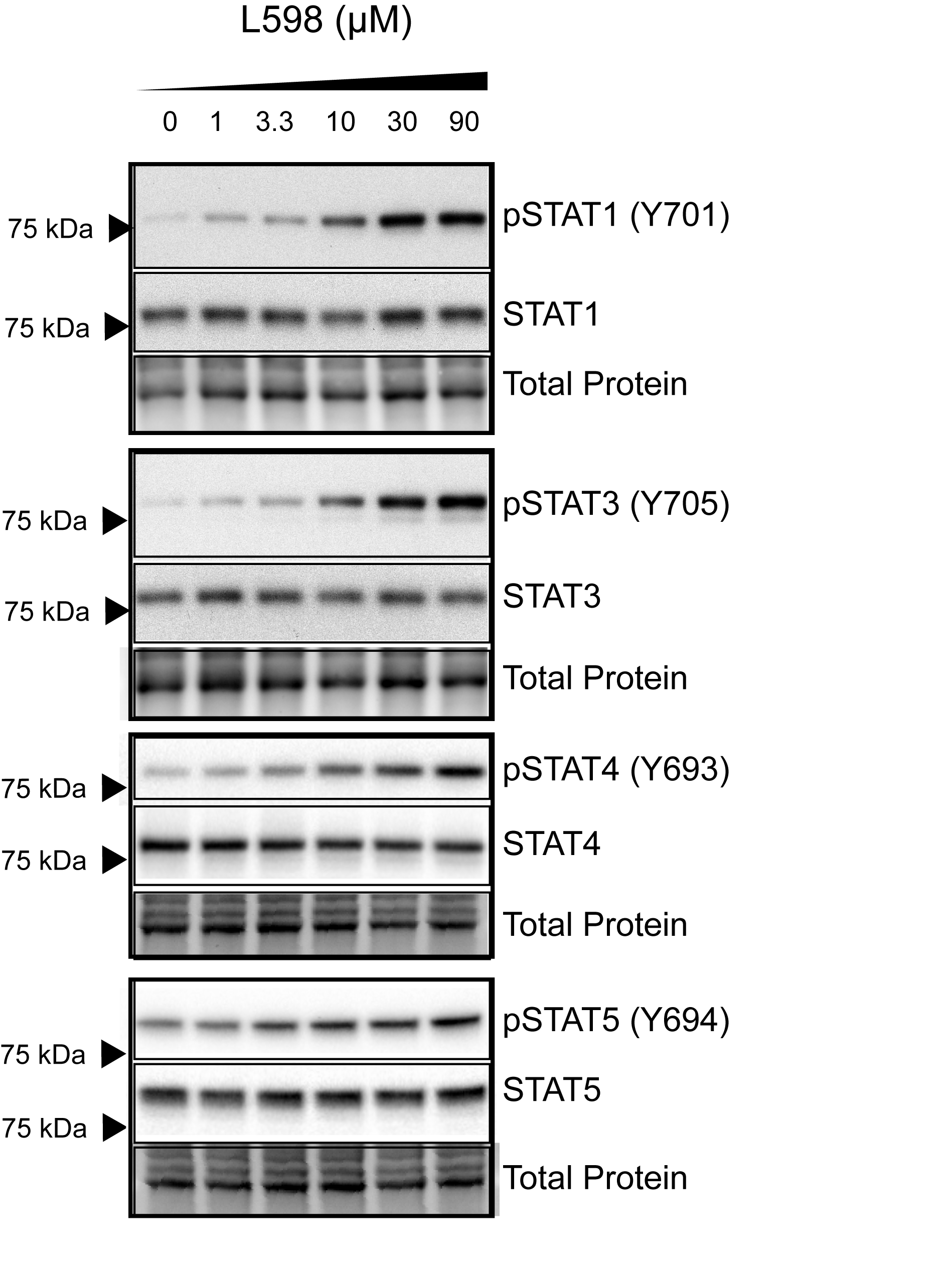

Supplement: Supplementary file 6 — Source data Fig. 4 [file 44319_2026_745_MOESM6_ESM.zip › Figure 4/4A/4A.tif]

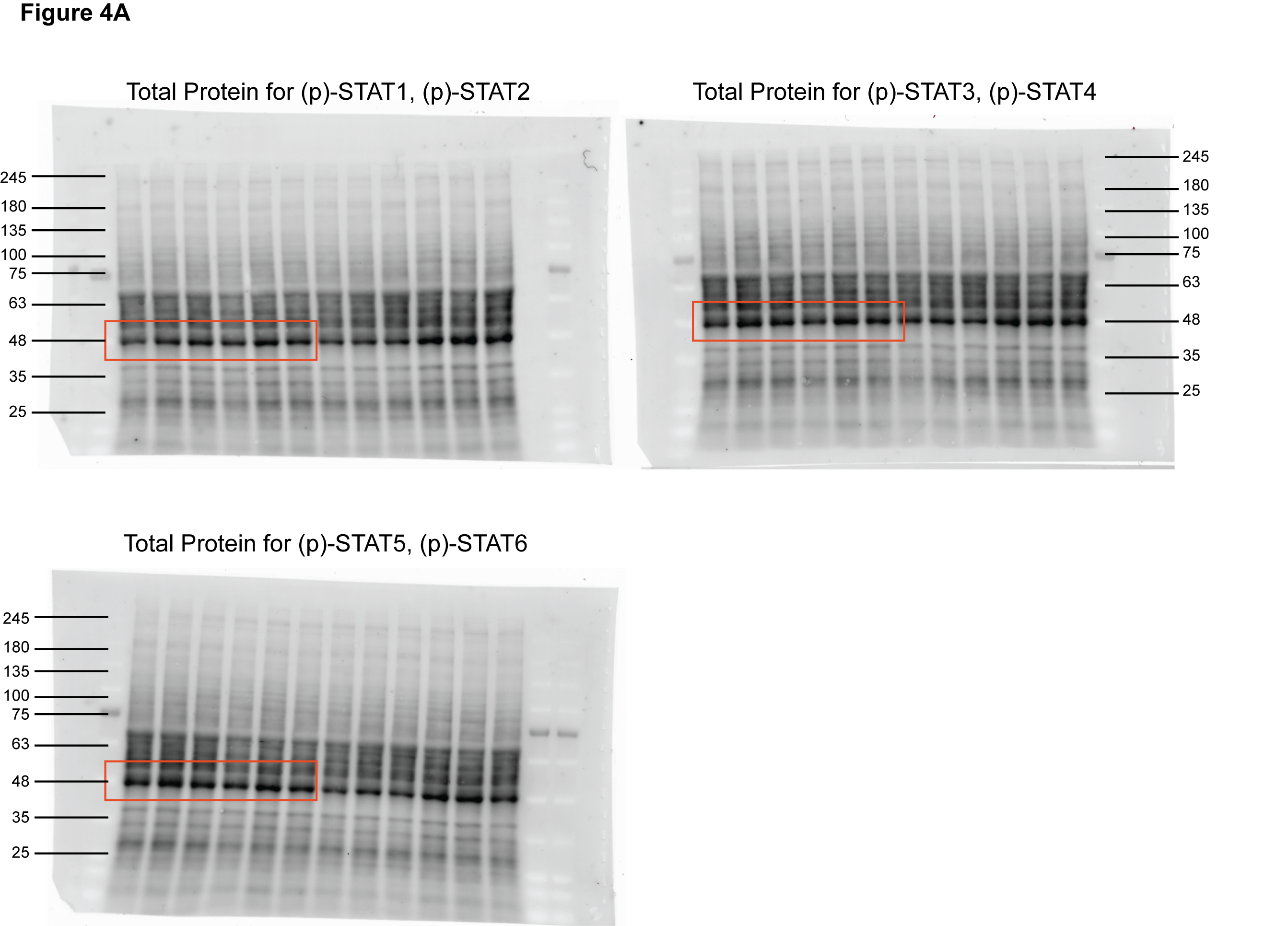

Supplement: Supplementary file 6 — Source data Fig. 4 [file 44319_2026_745_MOESM6_ESM.zip › Figure 4/4A/Replicates/4A_EXP1_Total Protein Loading Control.tif]

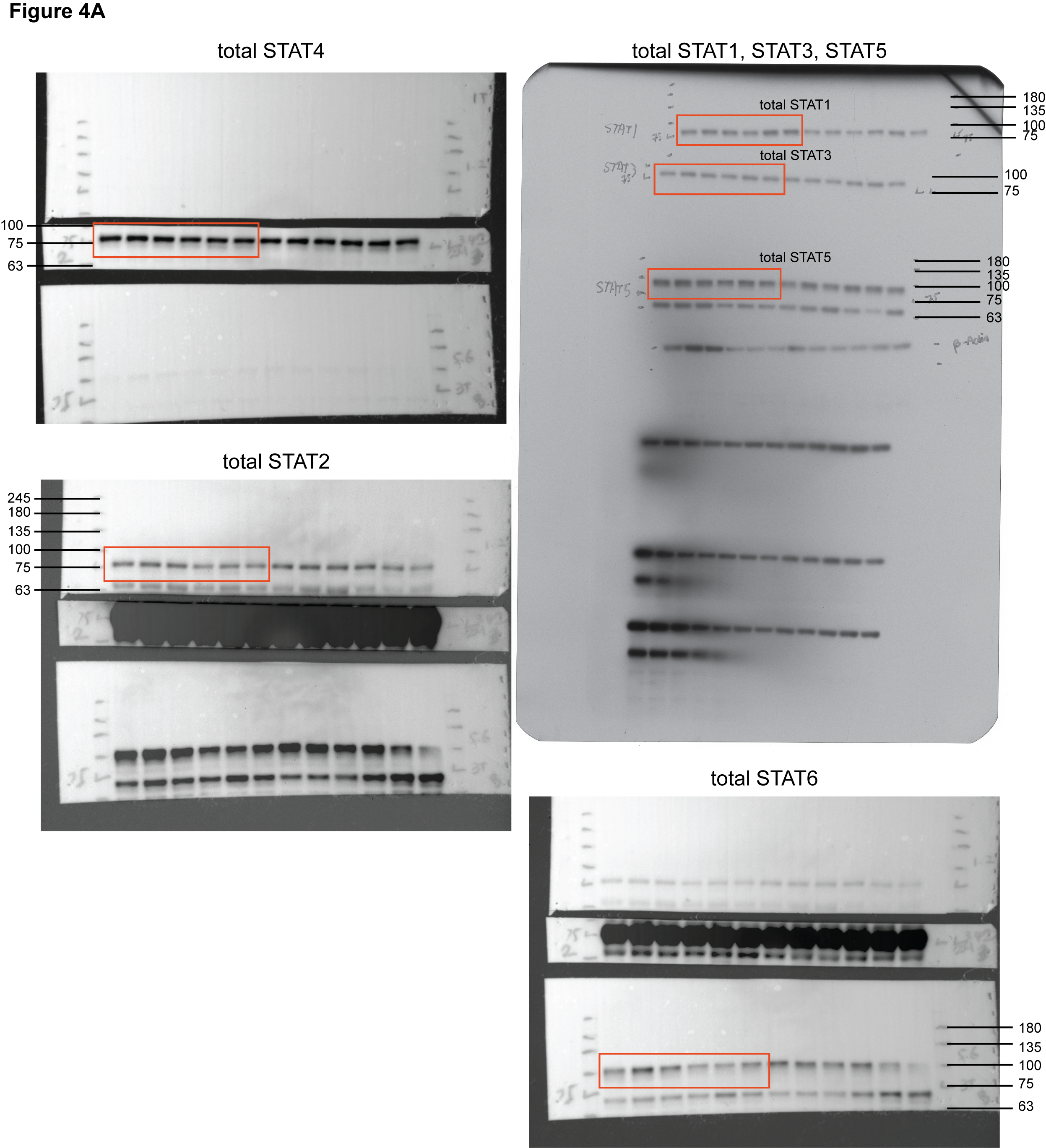

Supplement: Supplementary file 6 — Source data Fig. 4 [file 44319_2026_745_MOESM6_ESM.zip › Figure 4/4A/Replicates/4A_EXP1_Total STAT1_2_3_4_5_6.tif]

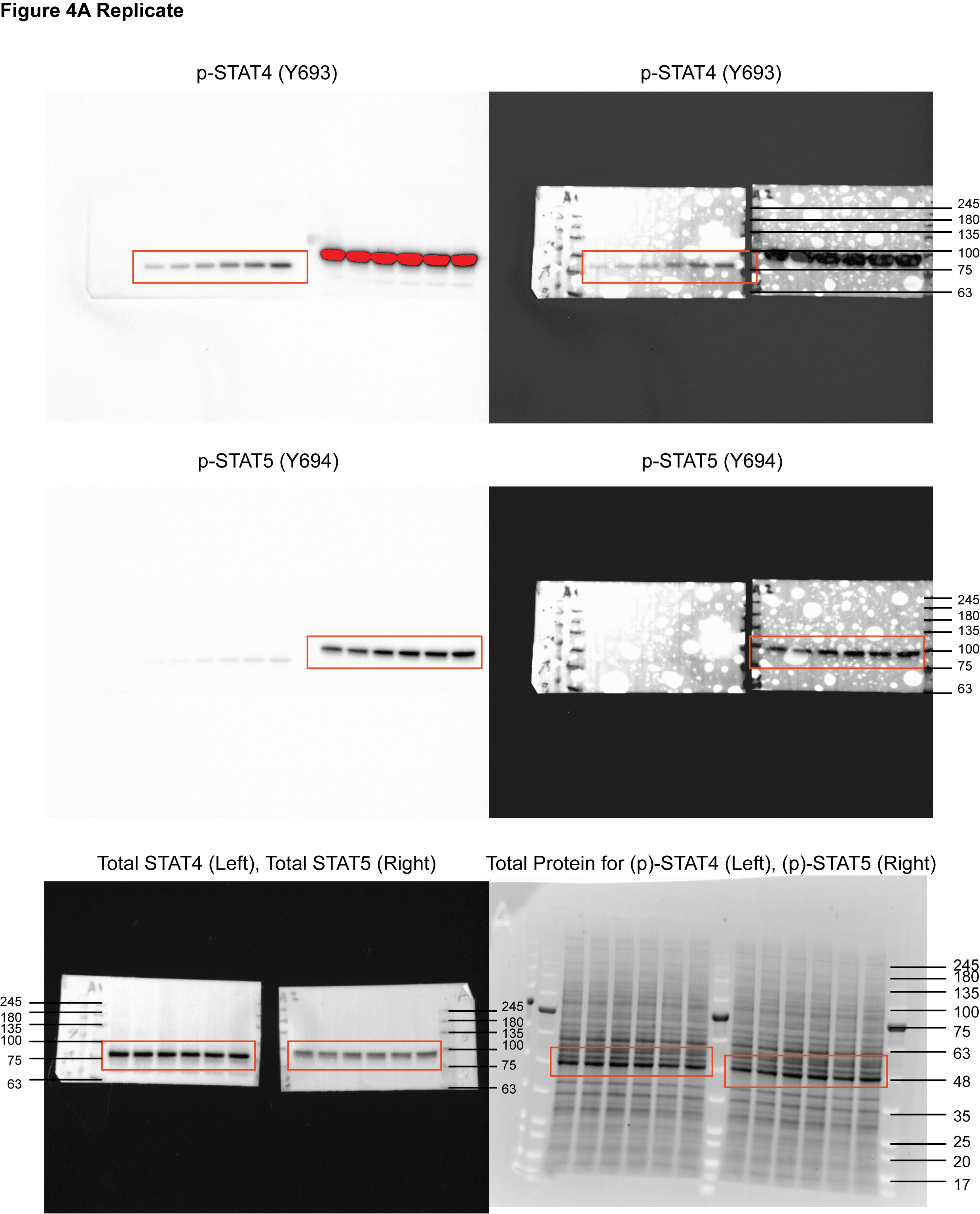

Supplement: Supplementary file 6 — Source data Fig. 4 [file 44319_2026_745_MOESM6_ESM.zip › Figure 4/4A/Replicates/4A_EXP2_PSTAT4_STAT5_Total Protein Loading Control.tif]

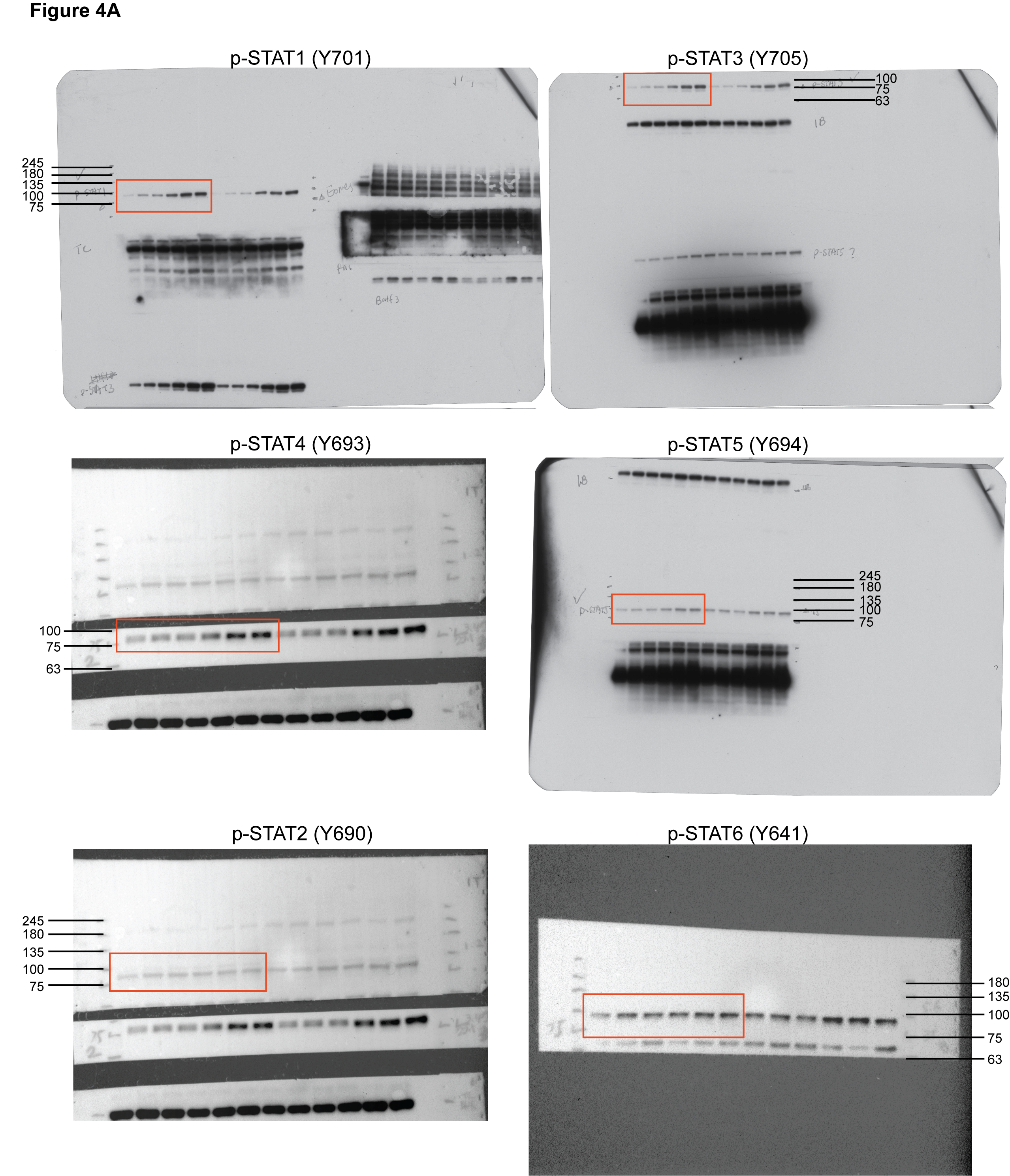

Supplement: Supplementary file 6 — Source data Fig. 4 [file 44319_2026_745_MOESM6_ESM.zip › Figure 4/4A/Replicates/4A_EXP1_Phosphor-STAT1_2_3_4_5_6.tif]

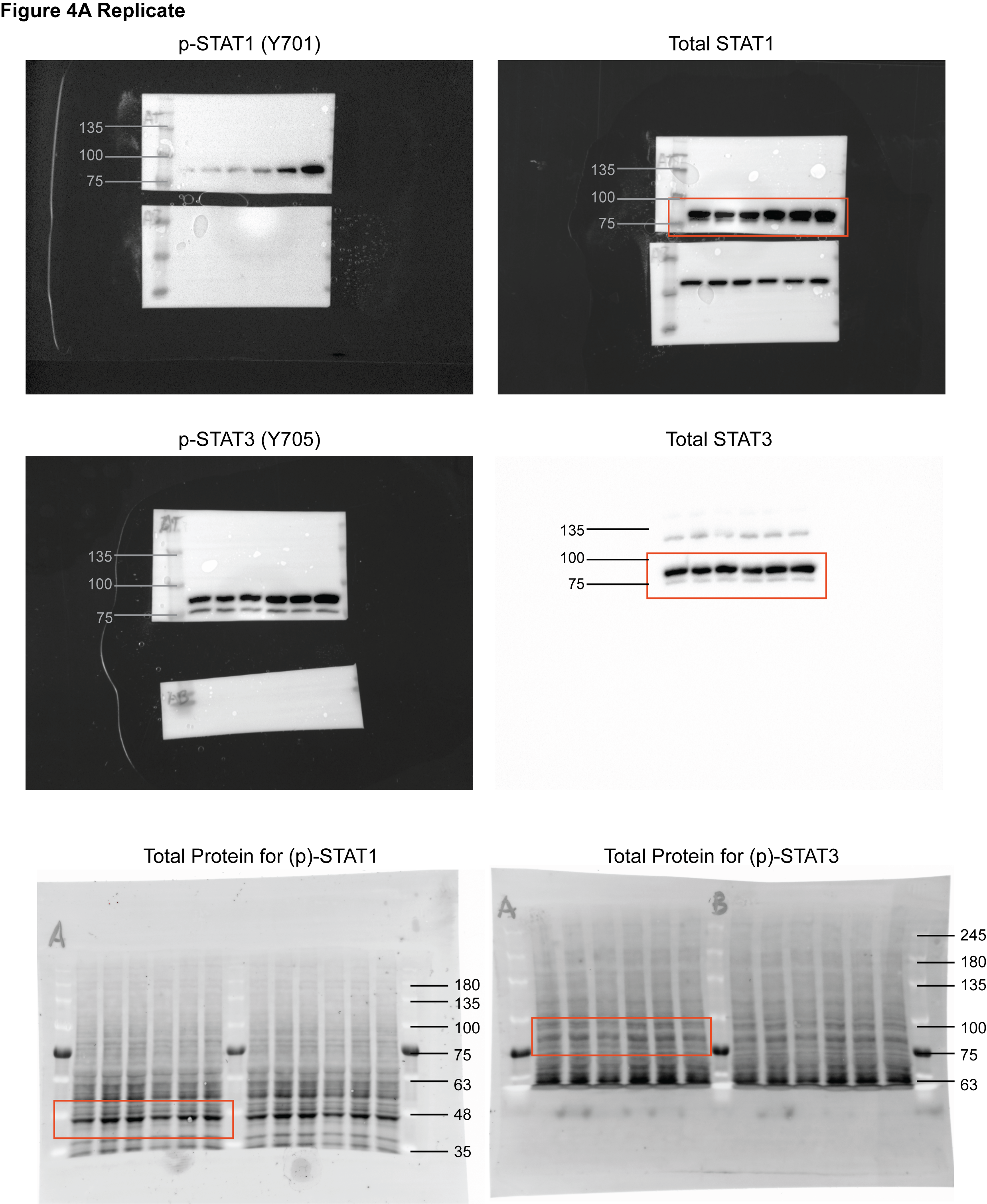

Supplement: Supplementary file 6 — Source data Fig. 4 [file 44319_2026_745_MOESM6_ESM.zip › Figure 4/4A/Replicates/4A_EXP2_PSTAT1_STAT3_and Total Protein.tif]

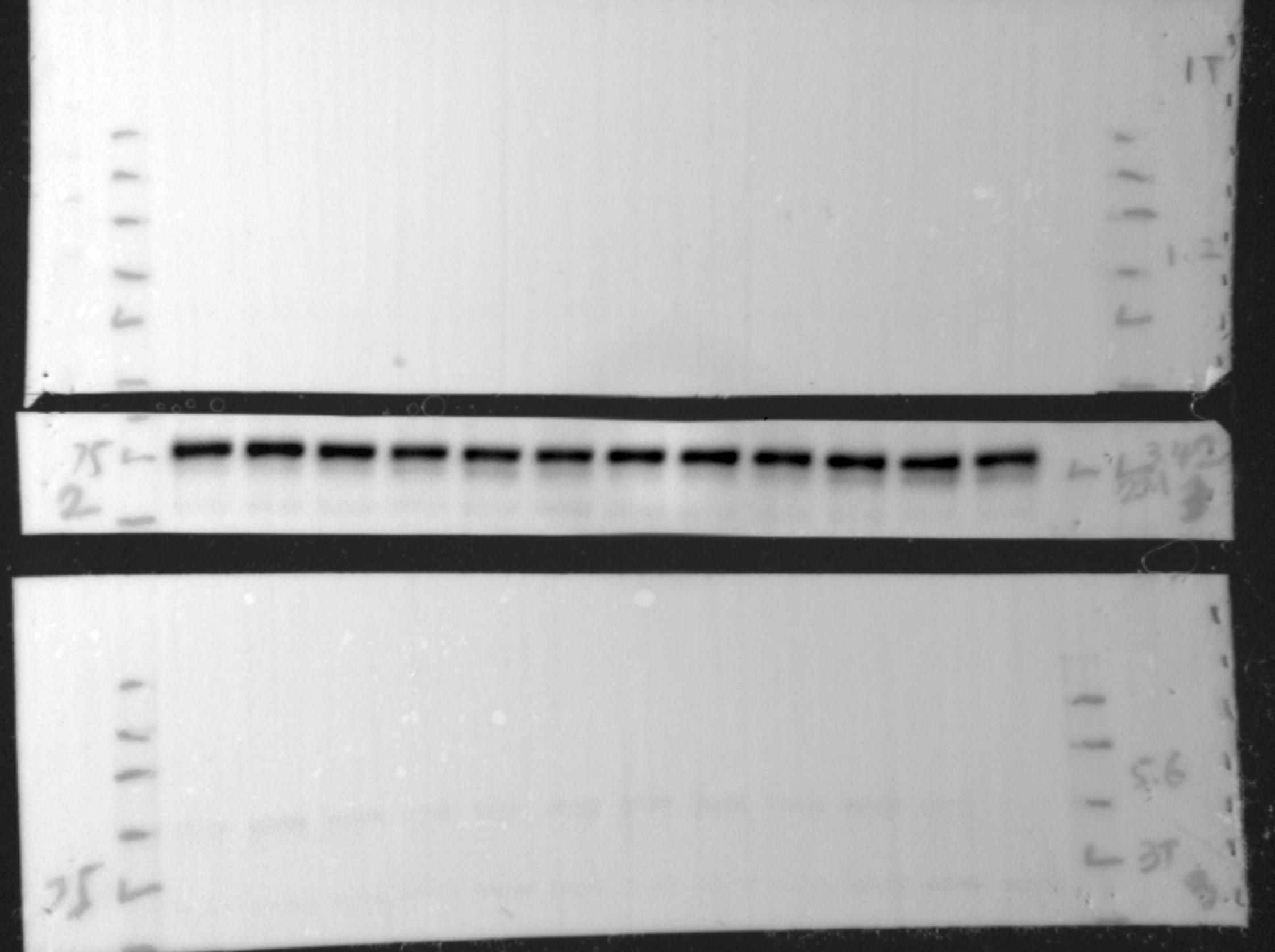

Supplement: Supplementary file 6 — Source data Fig. 4 [file 44319_2026_745_MOESM6_ESM.zip › Figure 4/4A/Raw Data/4A_EXP1/EXP1_3.6sec+colori STAT4.tif]

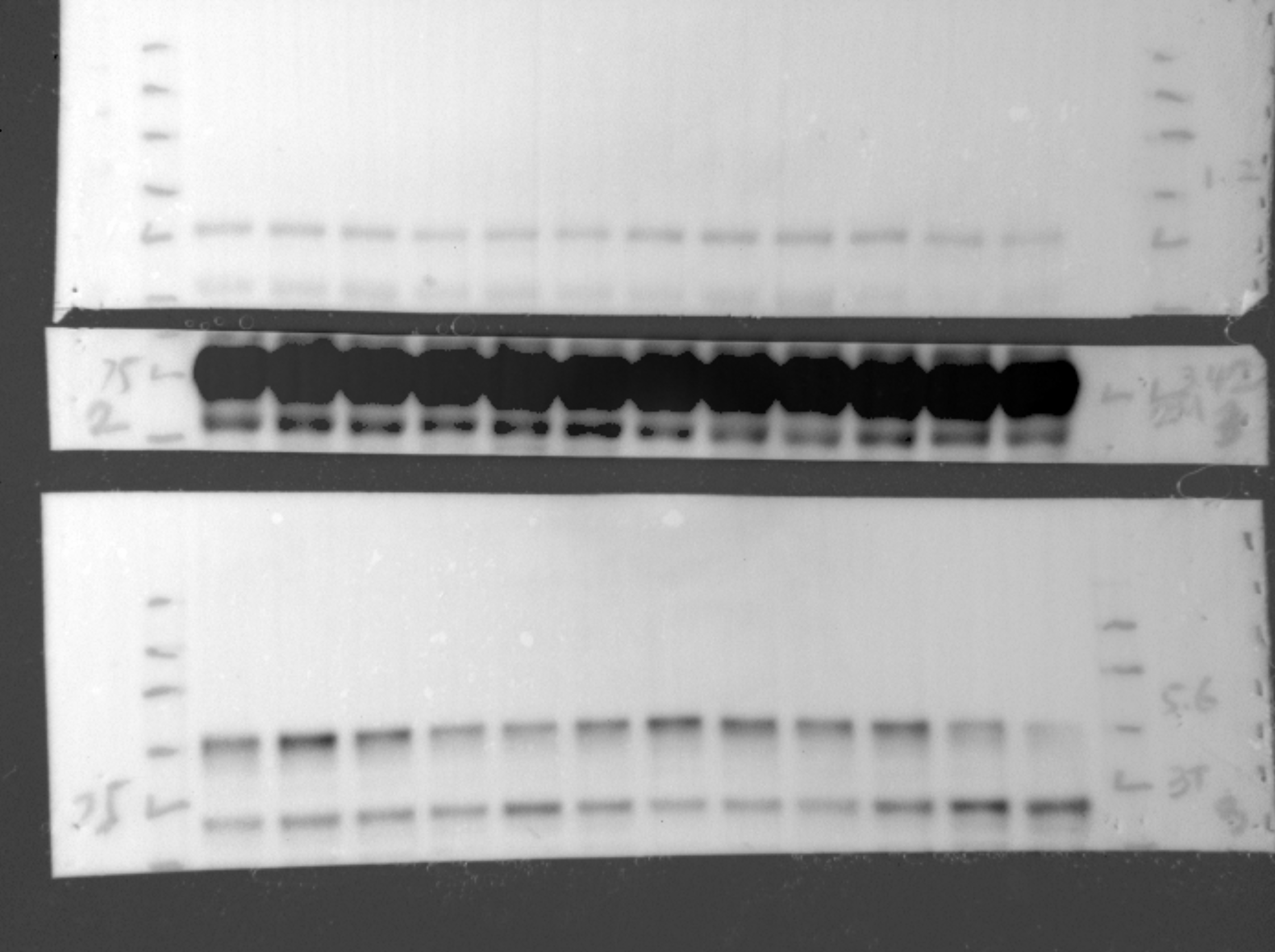

Supplement: Supplementary file 6 — Source data Fig. 4 [file 44319_2026_745_MOESM6_ESM.zip › Figure 4/4A/Raw Data/4A_EXP1/EXP1_73.6sec+colorib2_tSTAT2 tSTAT6.tif]

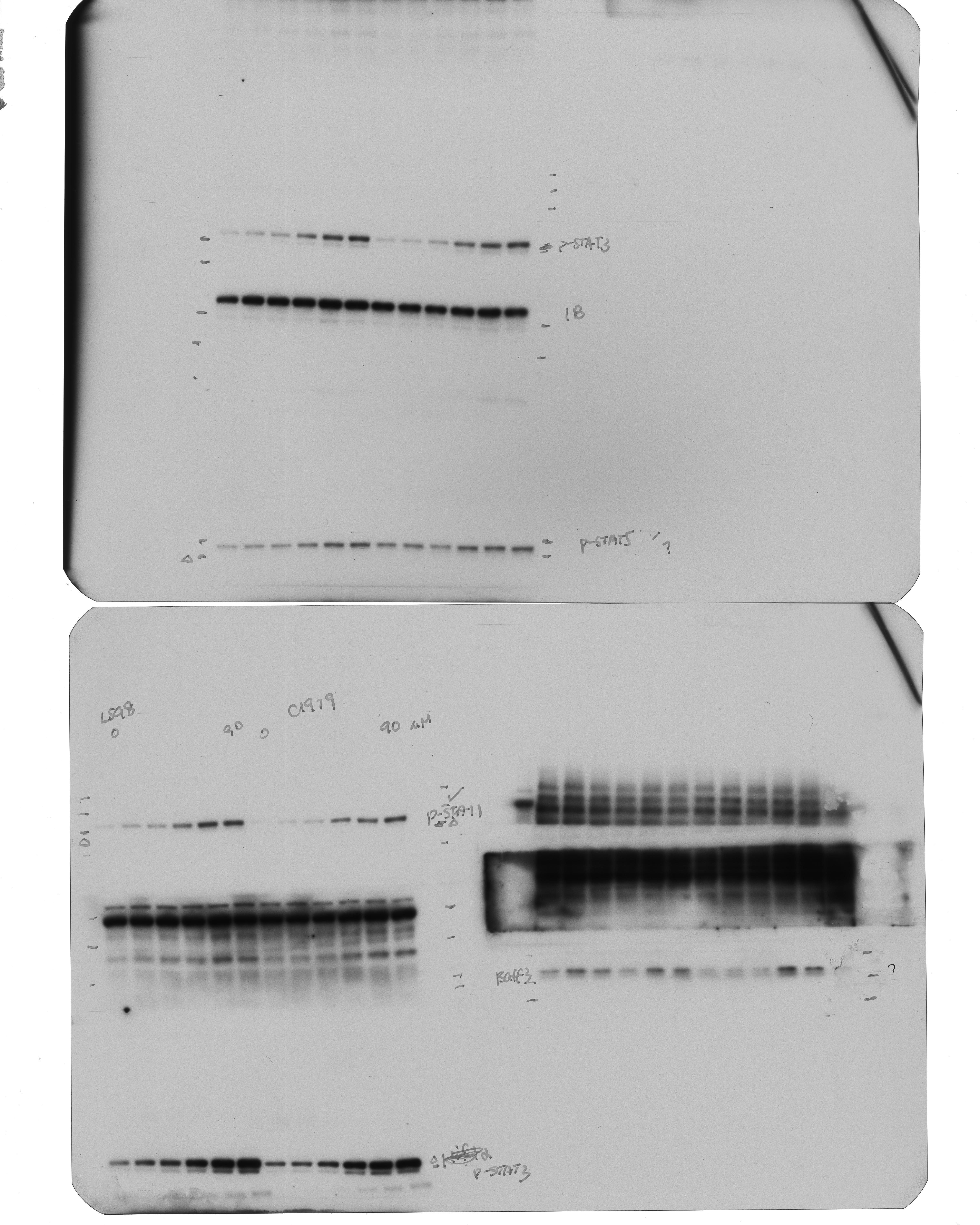

Supplement: Supplementary file 6 — Source data Fig. 4 [file 44319_2026_745_MOESM6_ESM.zip › Figure 4/4A/Raw Data/4A_EXP1/EXP1_20201024_pSTAT1_3_batf3007.jpg]

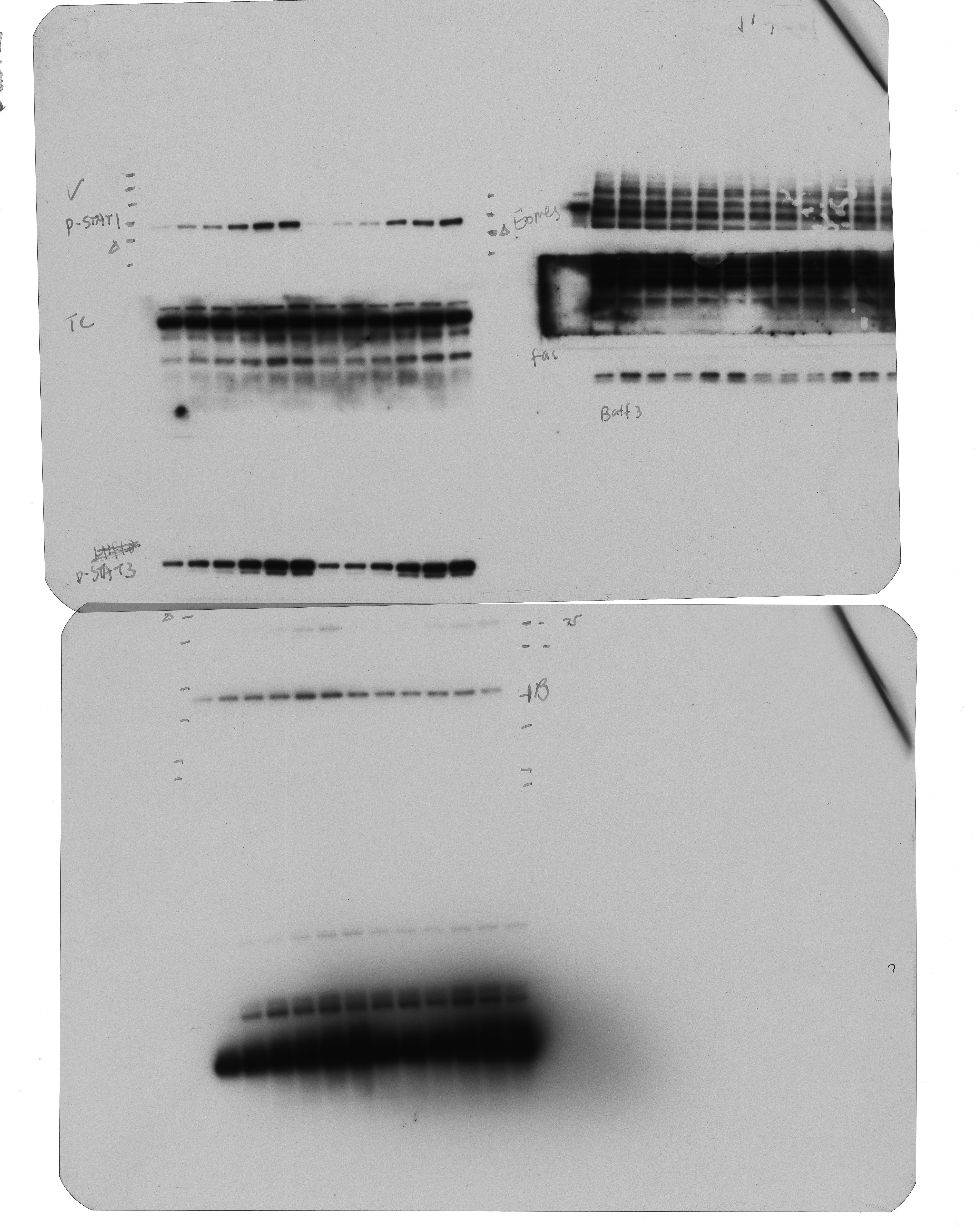

Supplement: Supplementary file 6 — Source data Fig. 4 [file 44319_2026_745_MOESM6_ESM.zip › Figure 4/4A/Raw Data/4A_EXP1/EXP1_20201024_pSTAT1_ptp1b_batf3008.jpg]

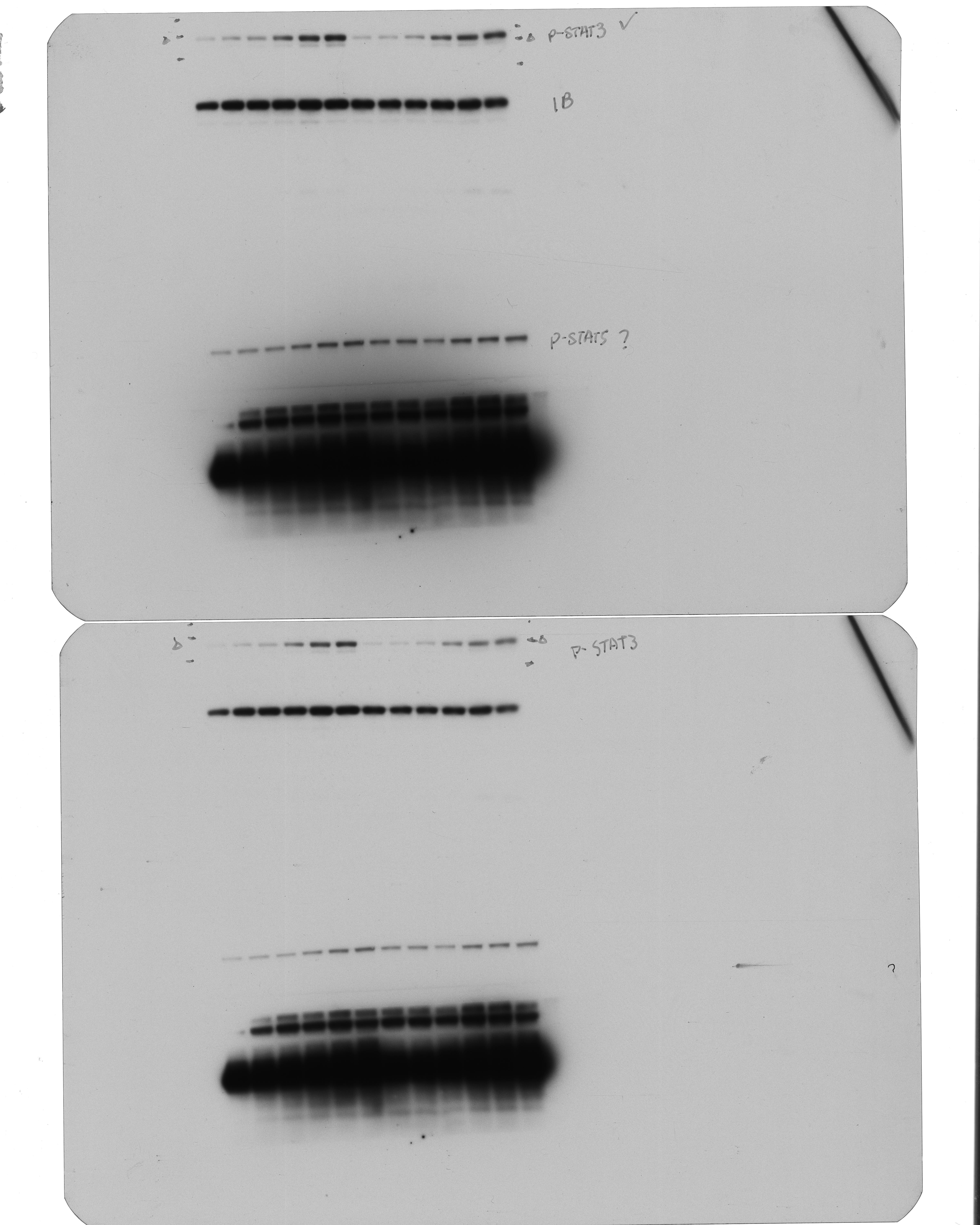

Supplement: Supplementary file 6 — Source data Fig. 4 [file 44319_2026_745_MOESM6_ESM.zip › Figure 4/4A/Raw Data/4A_EXP1/EXP1_20201024_pSTAT3_pSTAT5004.jpg]

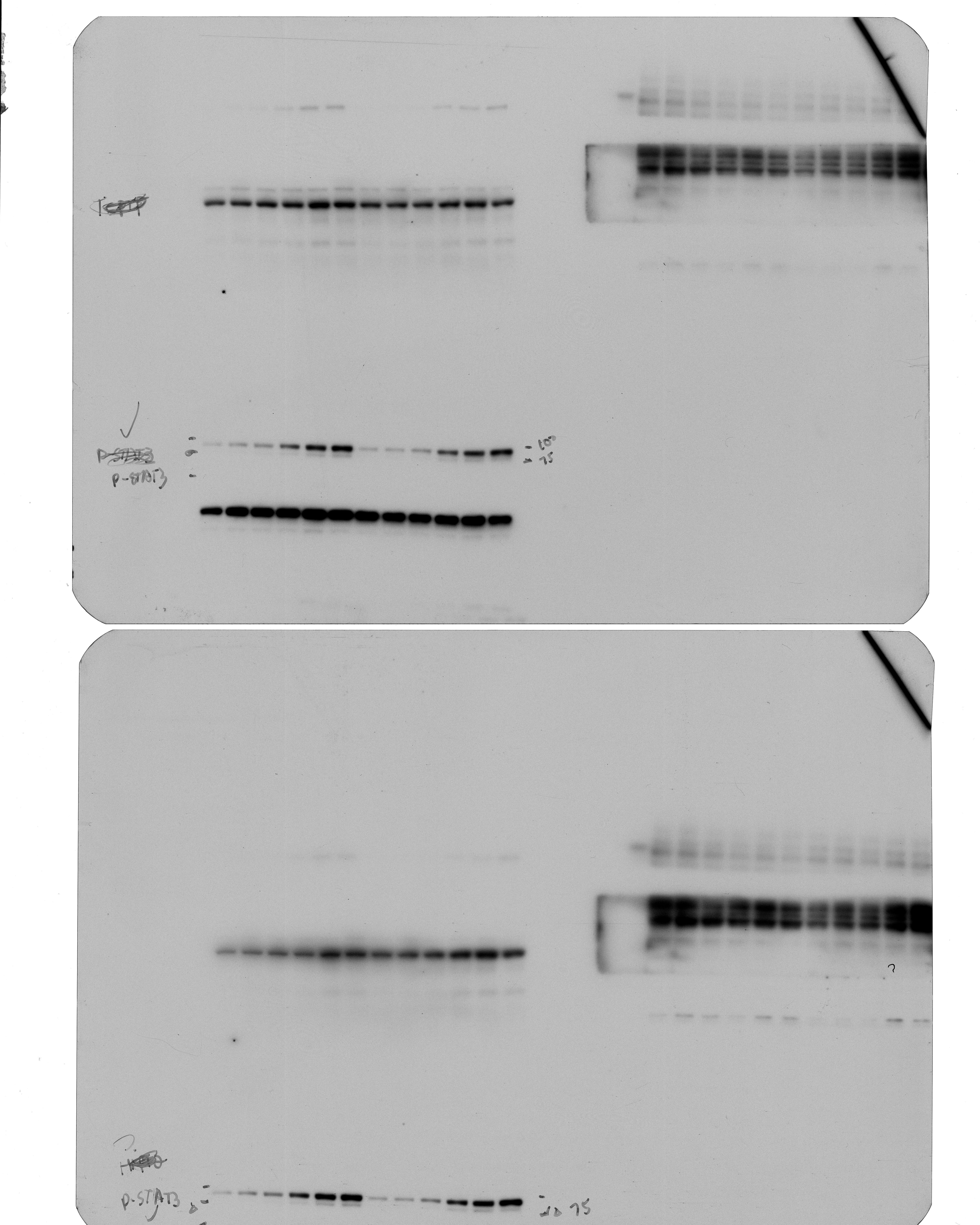

Supplement: Supplementary file 6 — Source data Fig. 4 [file 44319_2026_745_MOESM6_ESM.zip › Figure 4/4A/Raw Data/4A_EXP1/EXP1_20201024_pSTAT3_TCPTP005.jpg]

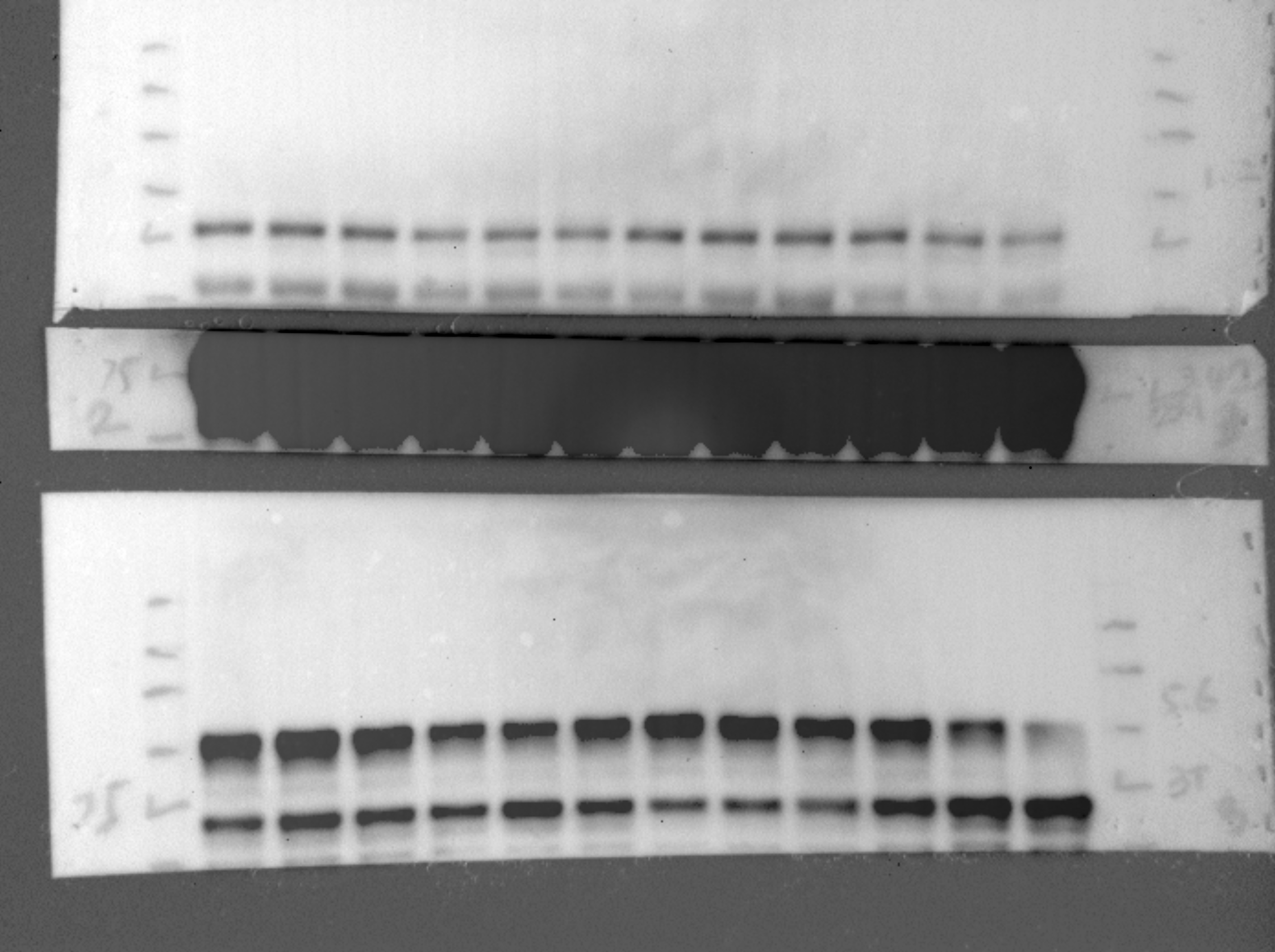

Supplement: Supplementary file 6 — Source data Fig. 4 [file 44319_2026_745_MOESM6_ESM.zip › Figure 4/4A/Raw Data/4A_EXP1/EXP1_colori+279.3sec STAT2.tif]

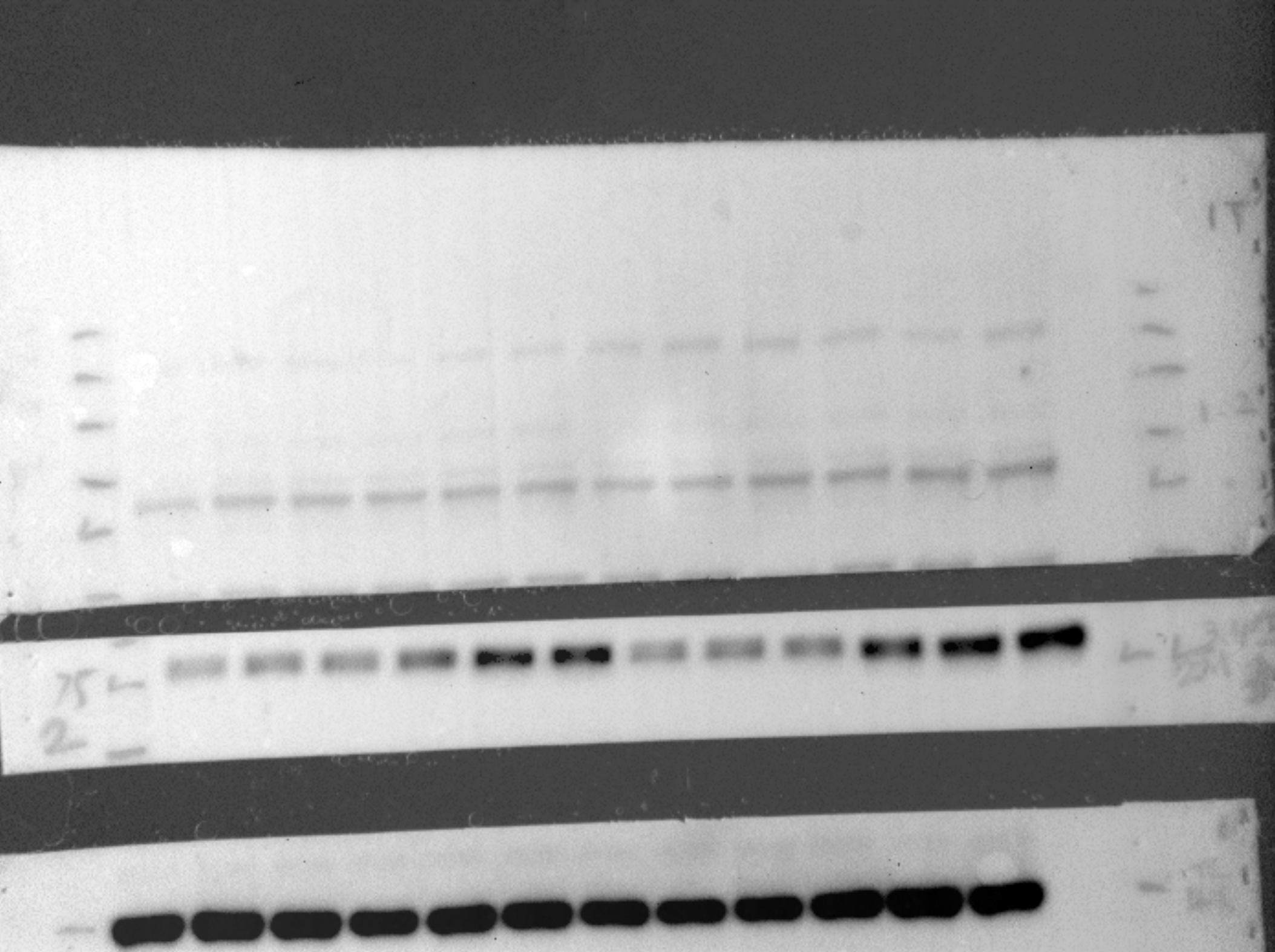

Supplement: Supplementary file 6 — Source data Fig. 4 [file 44319_2026_745_MOESM6_ESM.zip › Figure 4/4A/Raw Data/4A_EXP1/EXP1_pSTAT2, pSTAT4_352.3sec+colori.tif]

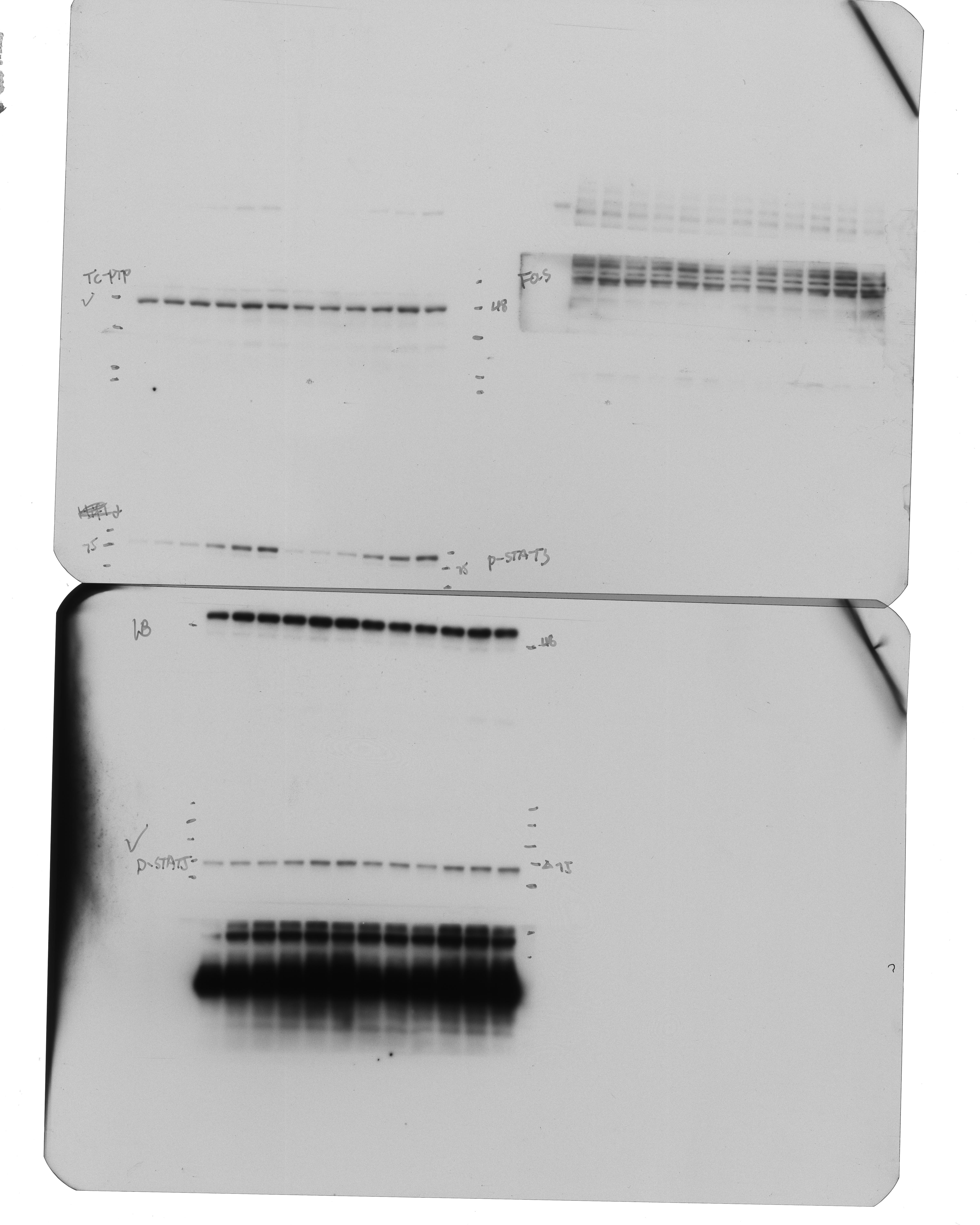

Supplement: Supplementary file 6 — Source data Fig. 4 [file 44319_2026_745_MOESM6_ESM.zip › Figure 4/4A/Raw Data/4A_EXP1/EXP1_pSTAT5_20201024_pSTAT3_5_TCPTP006.jpg]

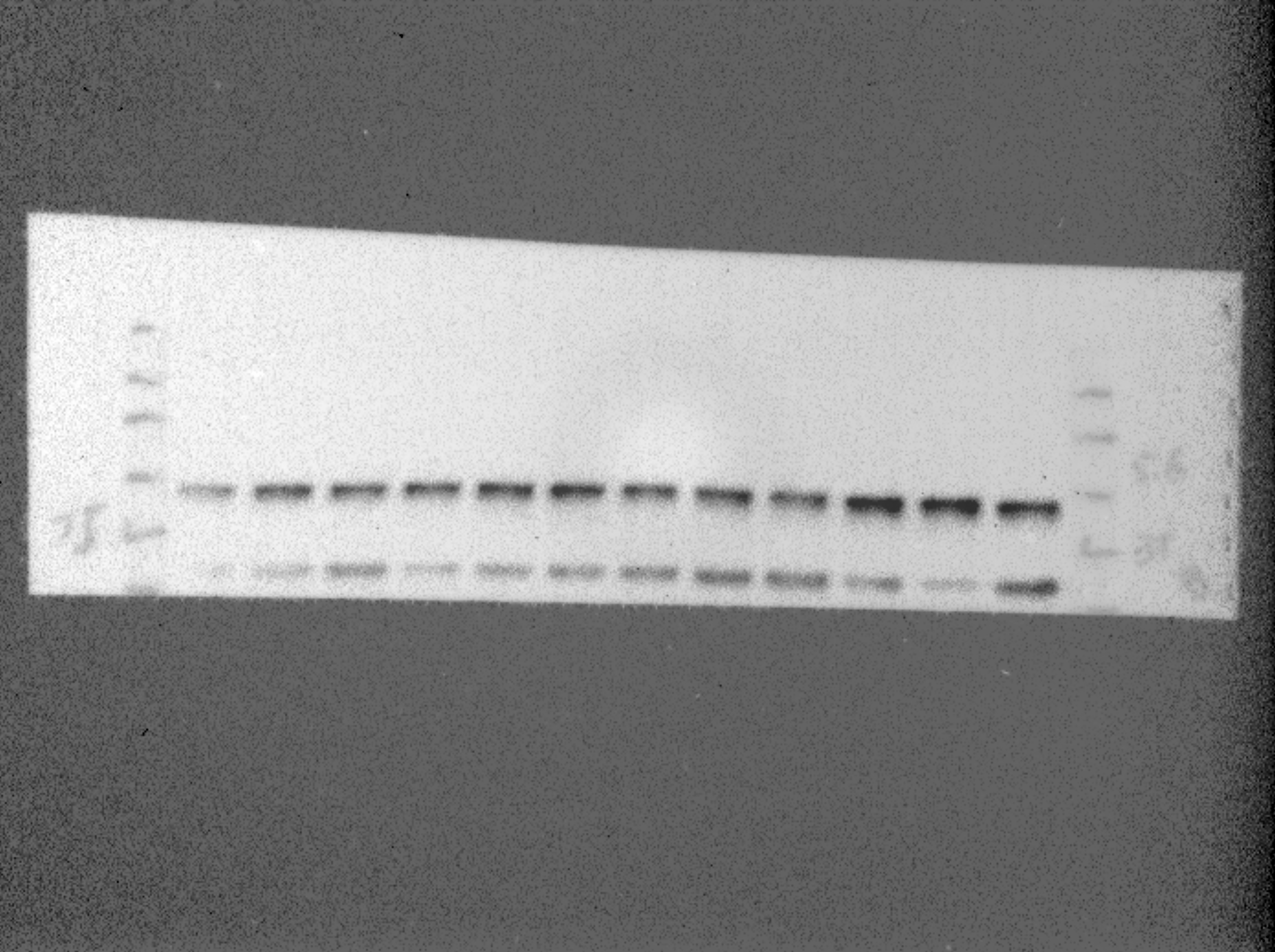

Supplement: Supplementary file 6 — Source data Fig. 4 [file 44319_2026_745_MOESM6_ESM.zip › Figure 4/4A/Raw Data/4A_EXP1/EXP1_pSTAT6_999.9sec+colori.tif]

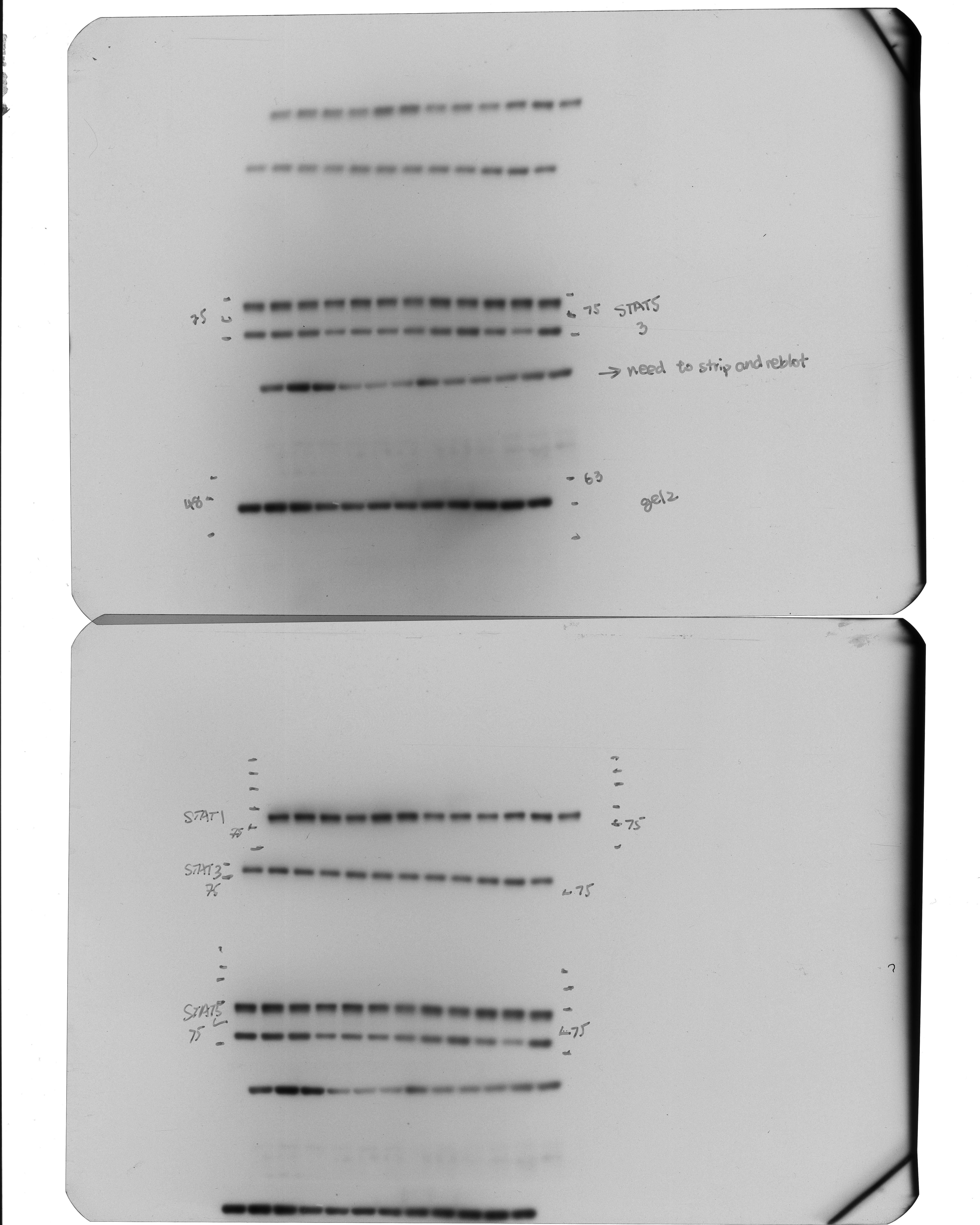

Supplement: Supplementary file 6 — Source data Fig. 4 [file 44319_2026_745_MOESM6_ESM.zip › Figure 4/4A/Raw Data/4A_EXP1/EXP1_STAT1_3_5001.jpg]

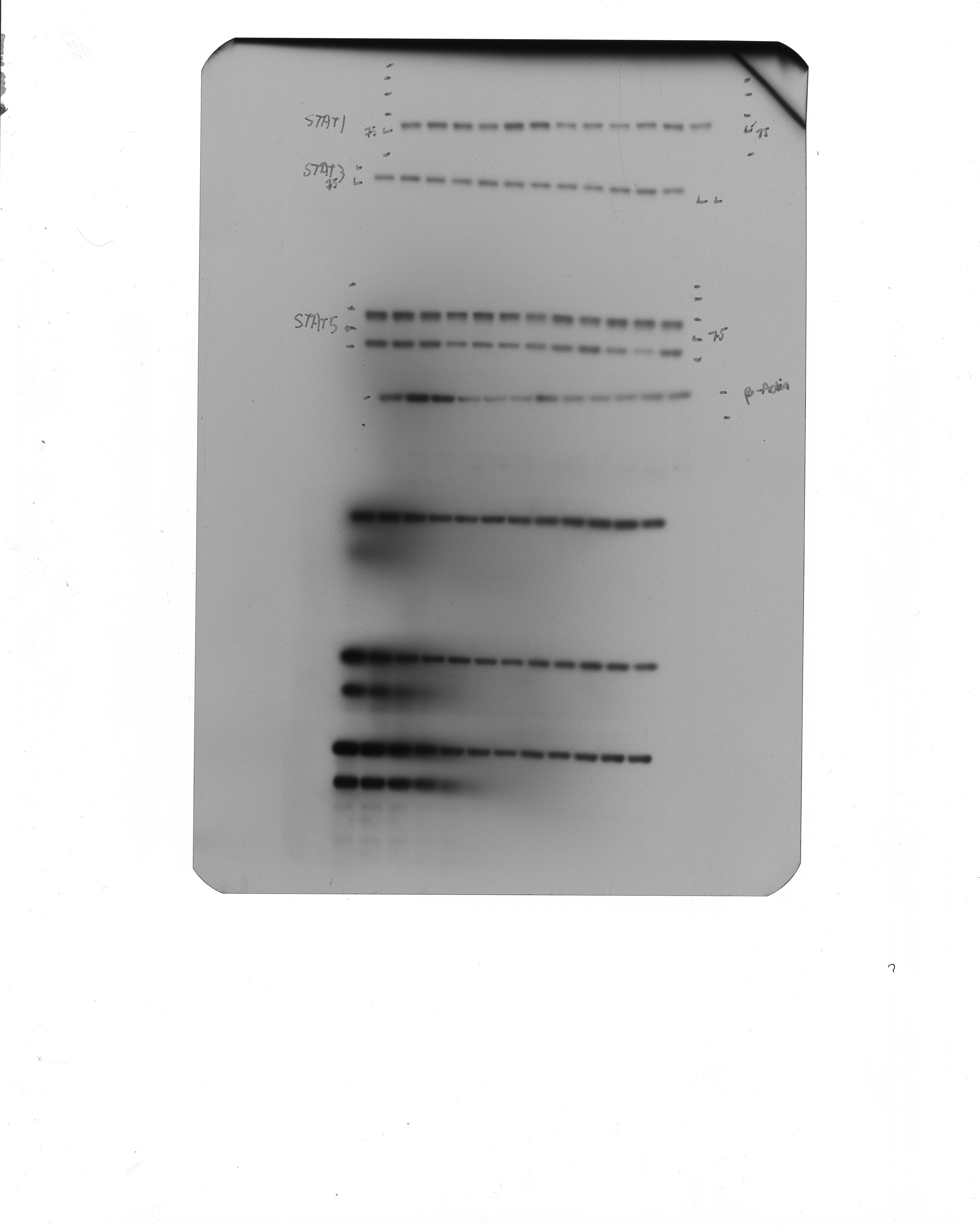

Supplement: Supplementary file 6 — Source data Fig. 4 [file 44319_2026_745_MOESM6_ESM.zip › Figure 4/4A/Raw Data/4A_EXP1/EXP1_STAT1_3_5002.jpg]

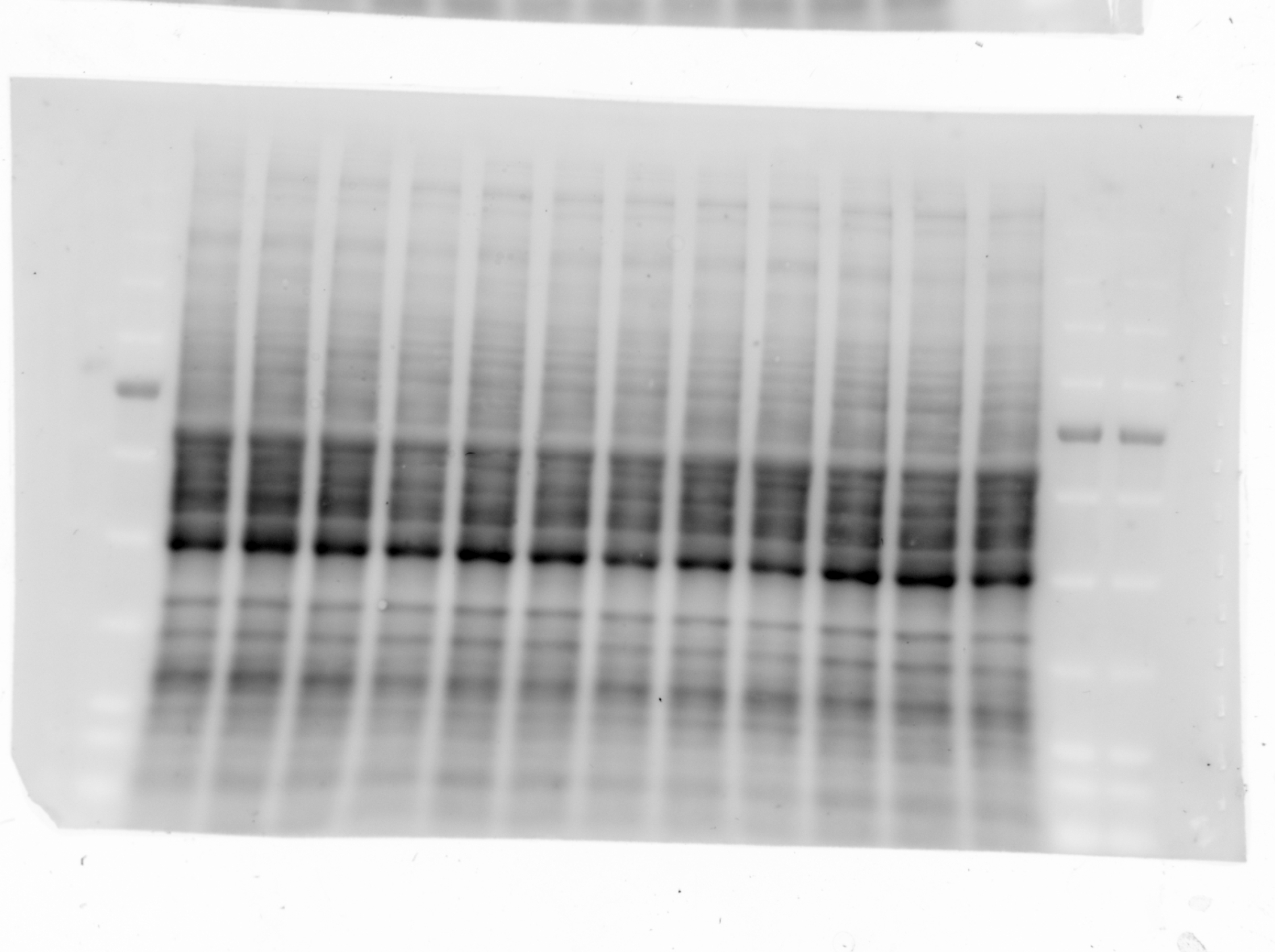

Supplement: Supplementary file 6 — Source data Fig. 4 [file 44319_2026_745_MOESM6_ESM.zip › Figure 4/4A/Raw Data/4A_EXP1/EXP1_TOTAL for total STAT5 STAT6_2020-10-24 01hr 02min g2.tif]

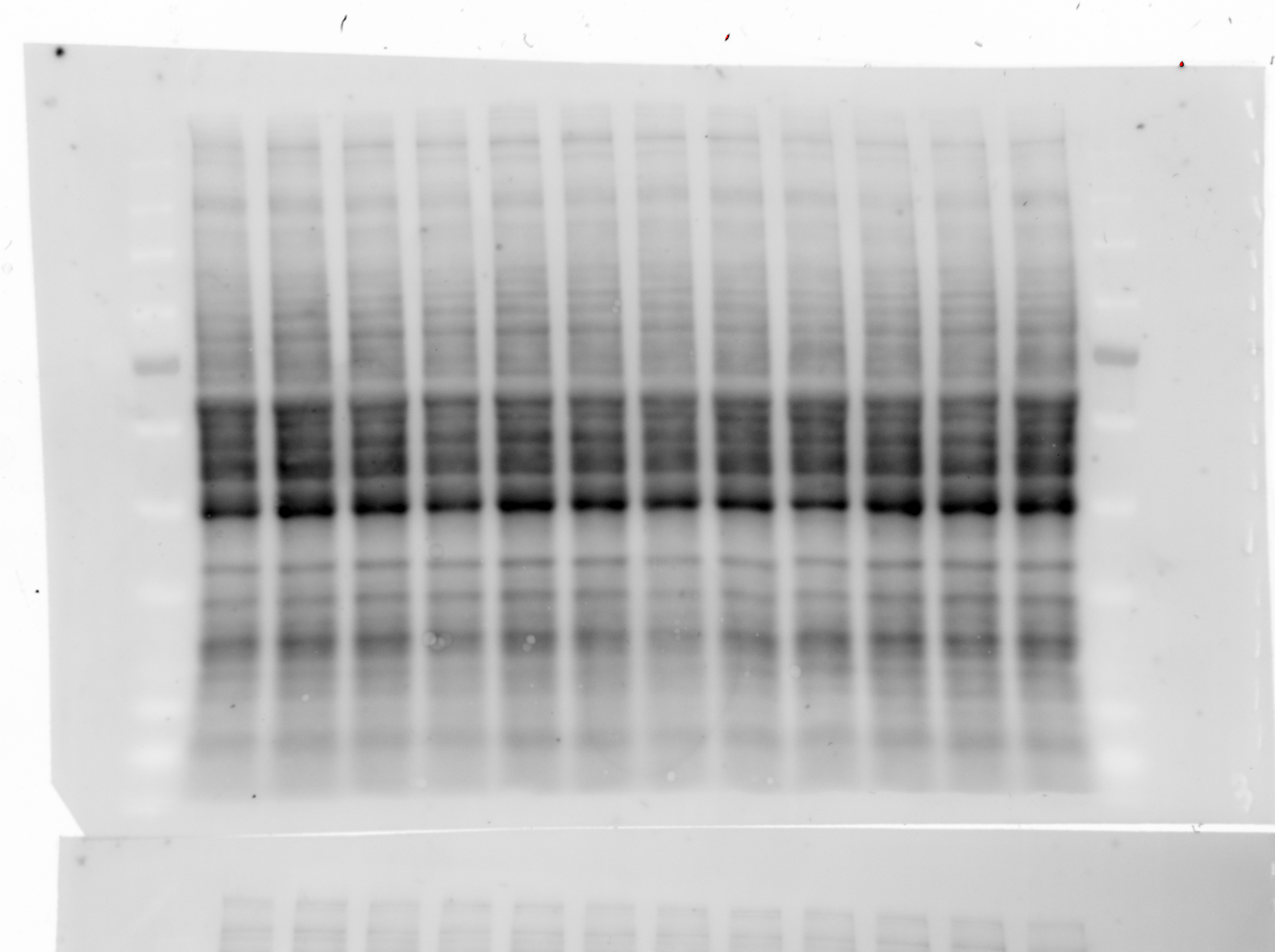

Supplement: Supplementary file 6 — Source data Fig. 4 [file 44319_2026_745_MOESM6_ESM.zip › Figure 4/4A/Raw Data/4A_EXP1/EXP1_total for totalSTAT3, STAT4_2020-10-24 01hr 07min g3.tif]
