# Supplementary figures and images for: PTPN1/PTPN2 inhibition improves NK cancer therapy by enhancing IL-2 and mitigating TGFβ1 responses (part 2 of 3)
Source: EMBO Rep. 2026 Apr 15;27(10):2581–613. doi: 10.1038/s44319-026-00745-0 (PMC13219468; doi:10.1038/s44319-026-00745-0)

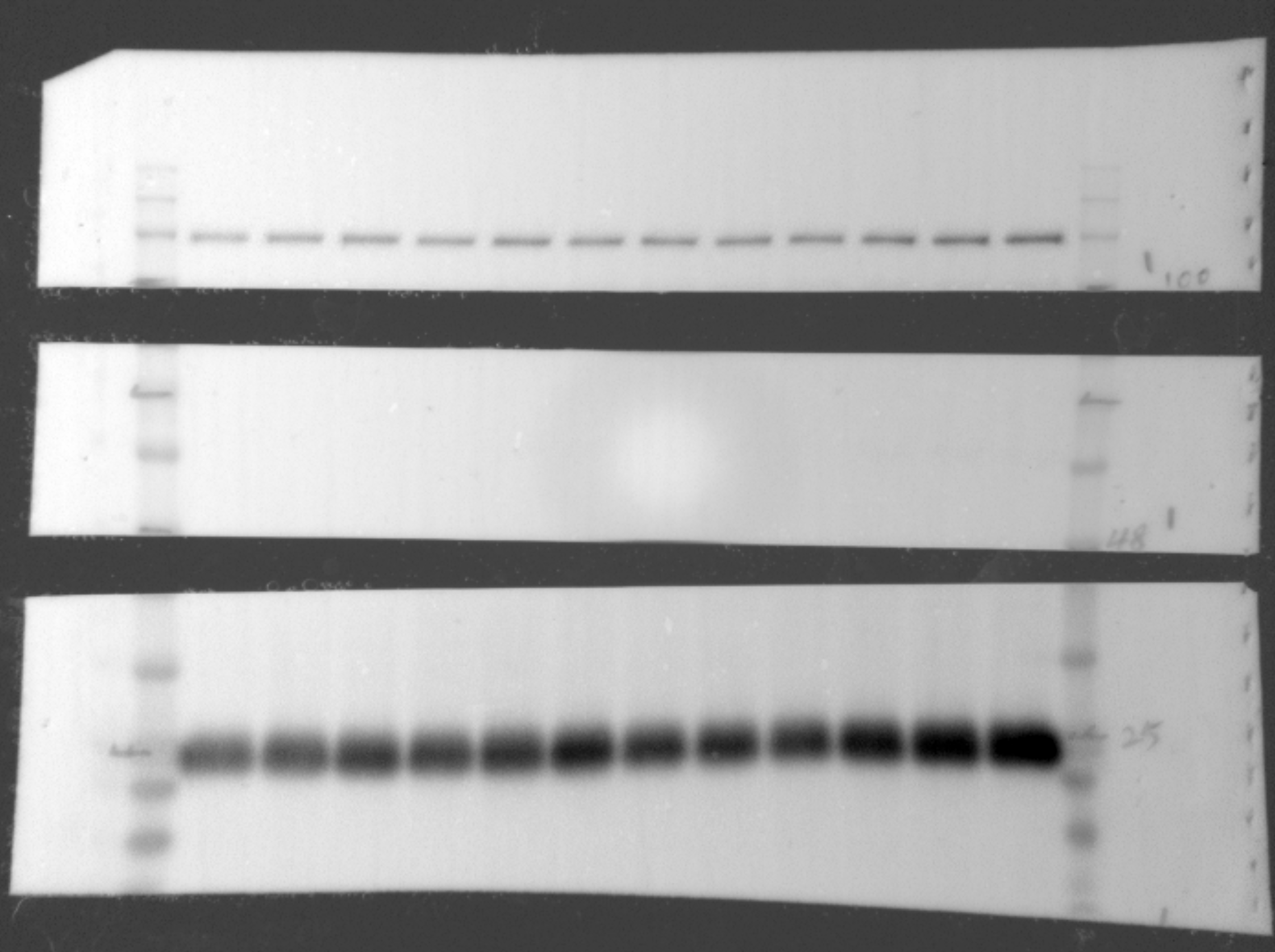

Supplement: Supplementary file 6 — Source data Fig. 4 [file 44319_2026_745_MOESM6_ESM.zip › Figure 4/4A/Raw Data/4A_EXP1/EXP1_Vinculin_ga_6.4sec+colori.tif]

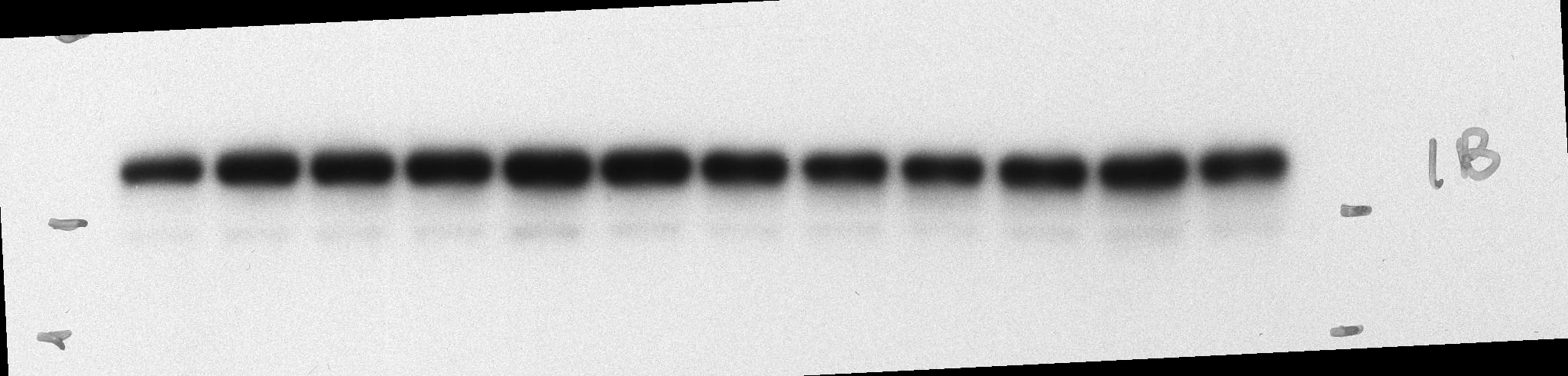

Supplement: Supplementary file 6 — Source data Fig. 4 [file 44319_2026_745_MOESM6_ESM.zip › Figure 4/4A/Raw Data/4A_EXP1/EXP1_ptp1b.tif]

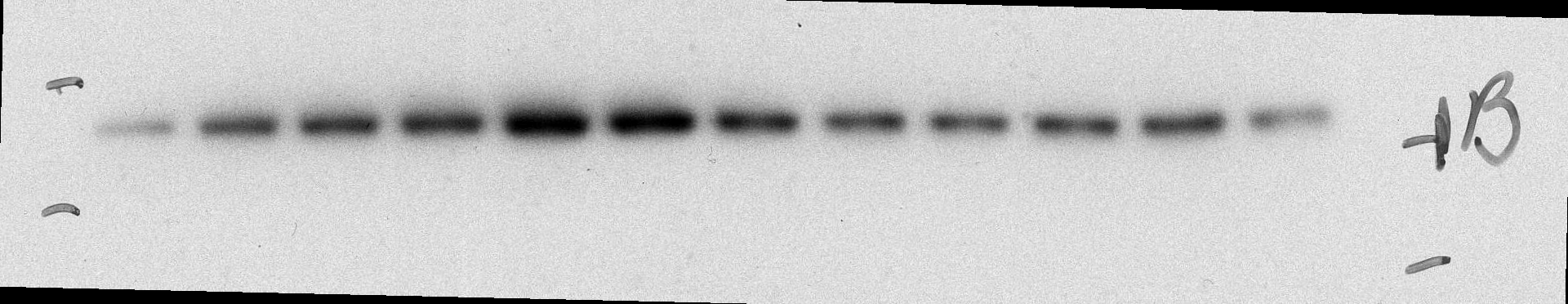

Supplement: Supplementary file 6 — Source data Fig. 4 [file 44319_2026_745_MOESM6_ESM.zip › Figure 4/4A/Raw Data/4A_EXP1/EXP1_PTP1B copy.tif]

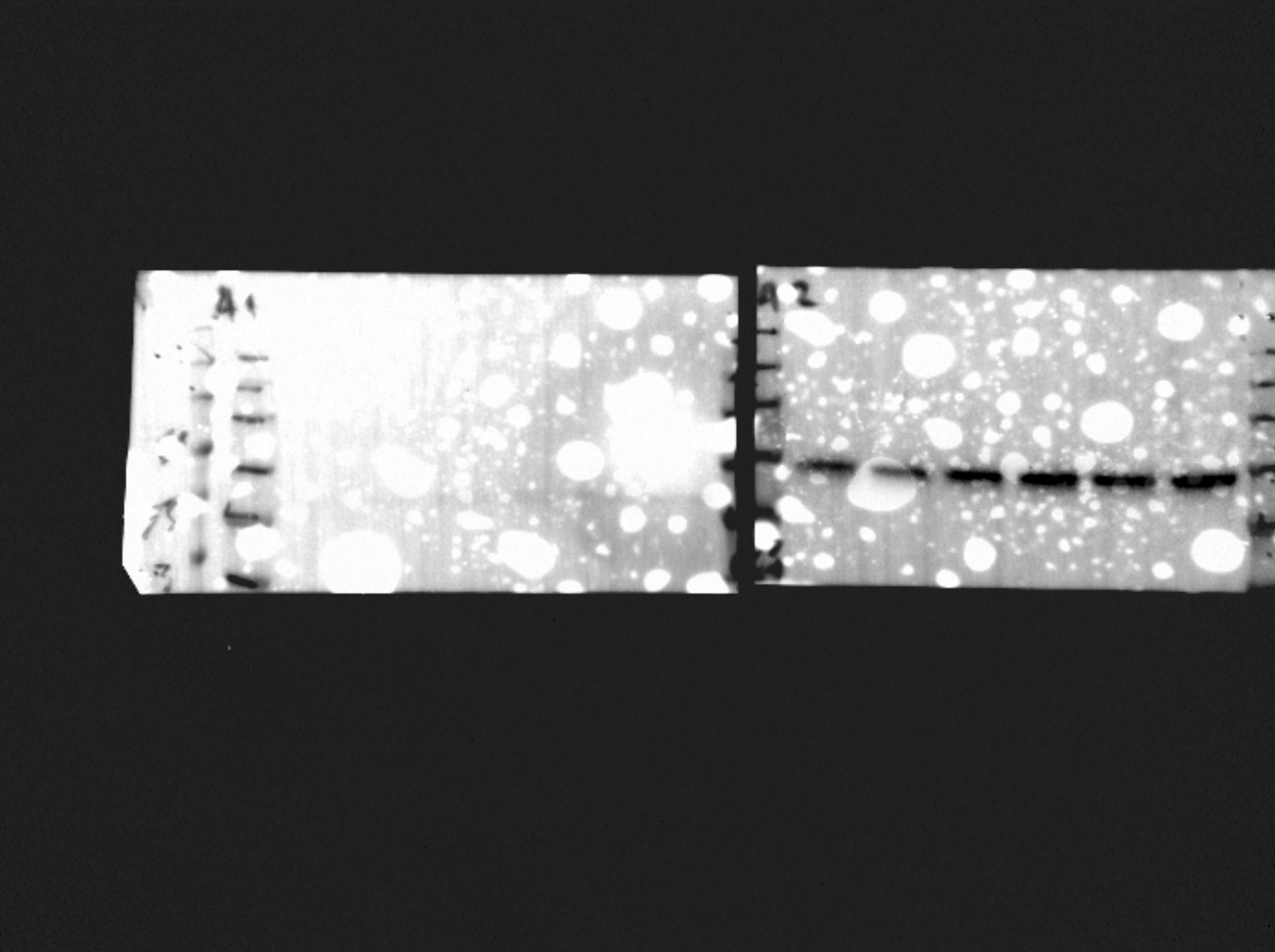

Supplement: Supplementary file 6 — Source data Fig. 4 [file 44319_2026_745_MOESM6_ESM.zip › Figure 4/4A/Raw Data/4A_EXP2/EXP2_pSTAT5 (Right)_Merge with Ladder.tif]

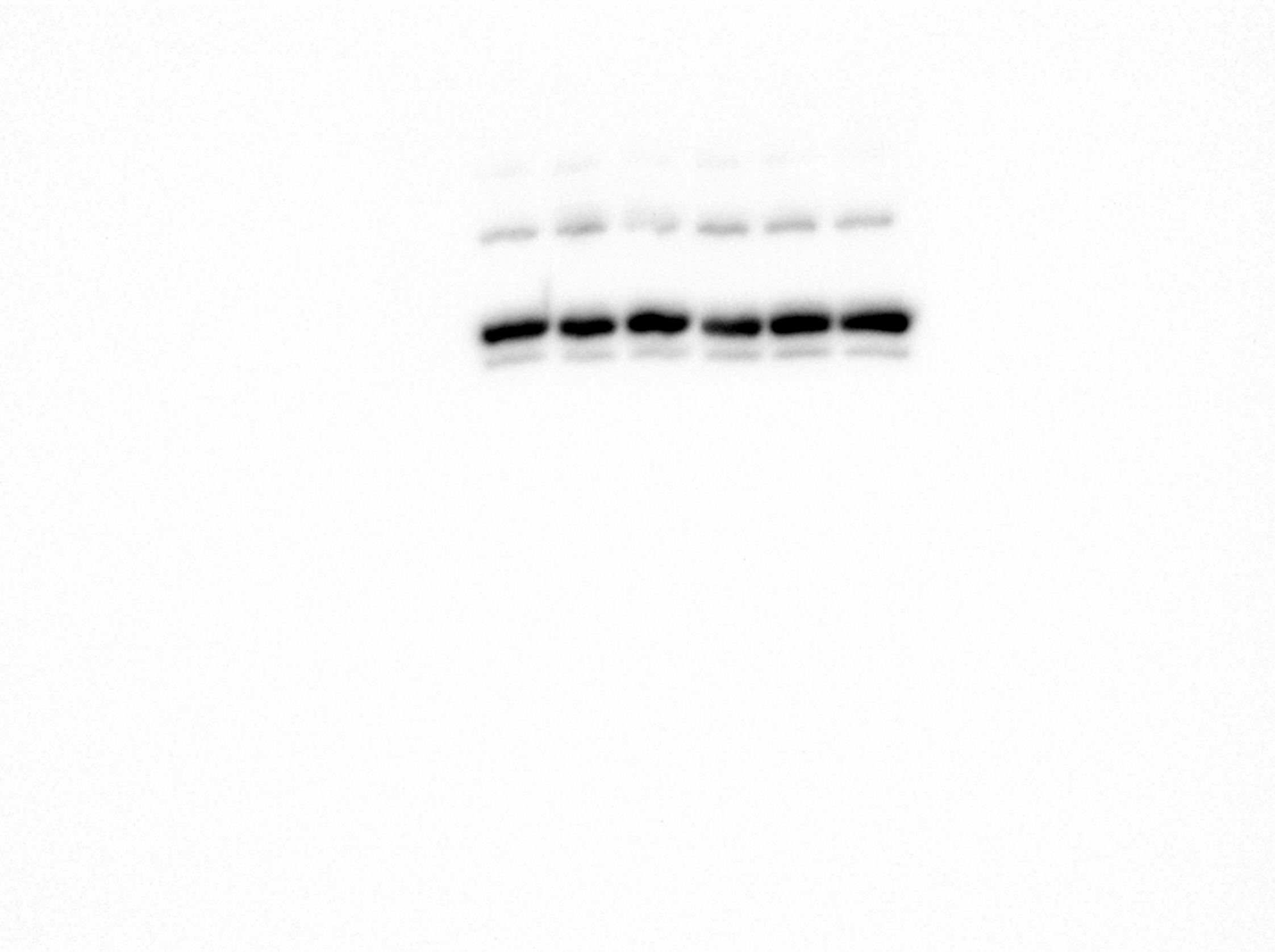

Supplement: Supplementary file 6 — Source data Fig. 4 [file 44319_2026_745_MOESM6_ESM.zip › Figure 4/4A/Raw Data/4A_EXP2/EXP2_STAT3.tif]

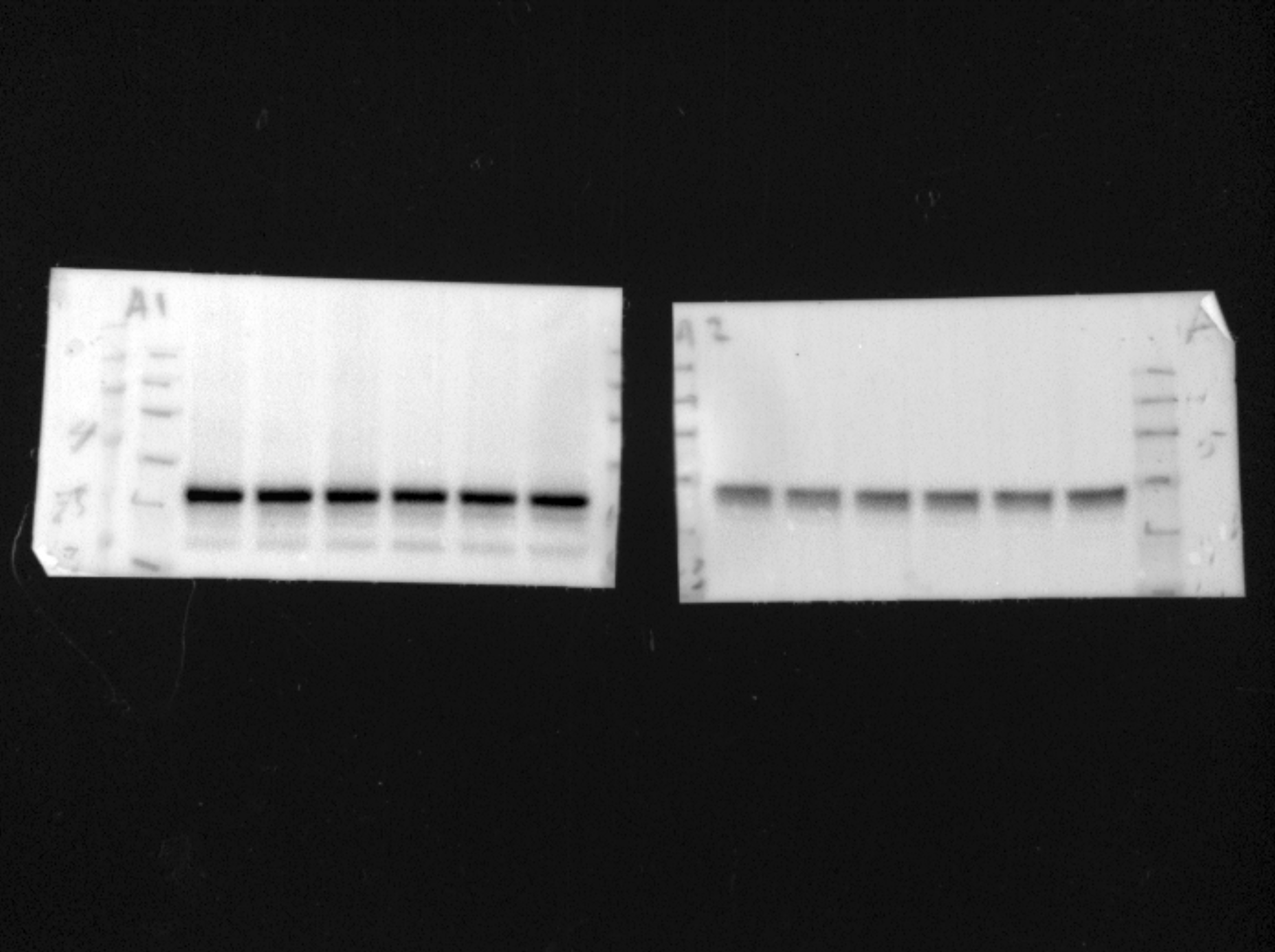

Supplement: Supplementary file 6 — Source data Fig. 4 [file 44319_2026_745_MOESM6_ESM.zip › Figure 4/4A/Raw Data/4A_EXP2/EXP2_STAT4(Left)_STAT5(Right).tif]

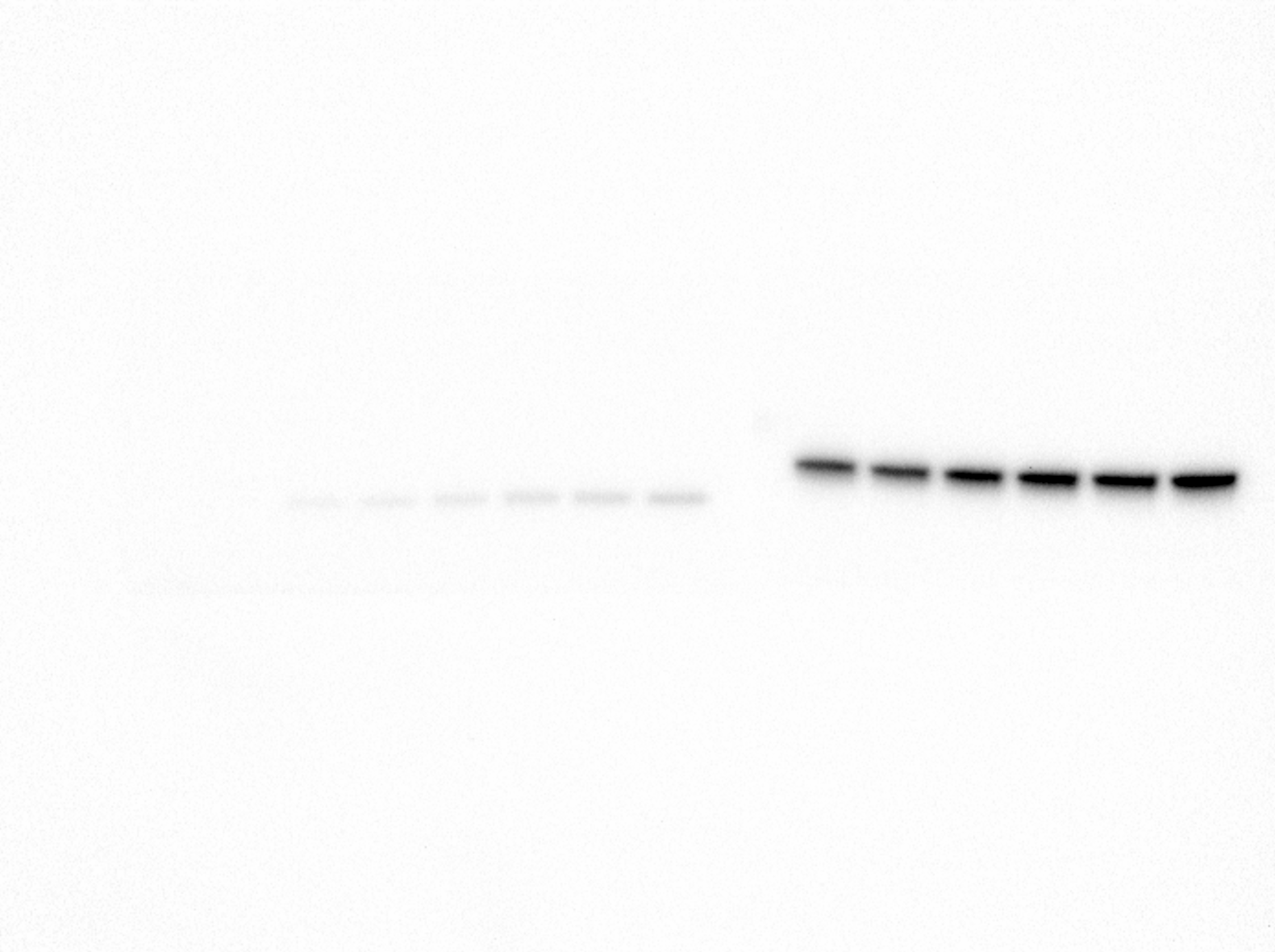

Supplement: Supplementary file 6 — Source data Fig. 4 [file 44319_2026_745_MOESM6_ESM.zip › Figure 4/4A/Raw Data/4A_EXP2/EXP2_pSTAT5 (Right).tif]

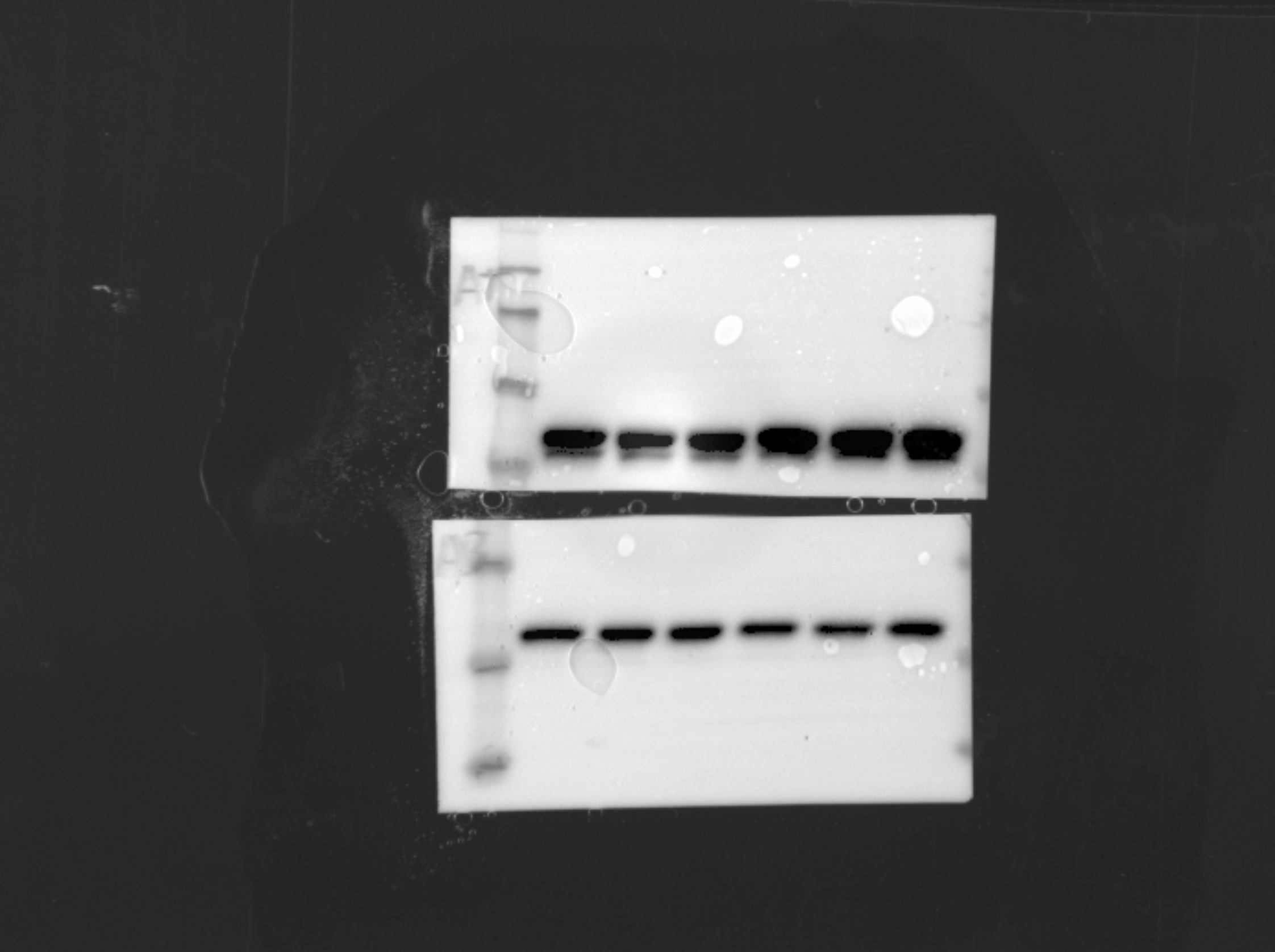

Supplement: Supplementary file 6 — Source data Fig. 4 [file 44319_2026_745_MOESM6_ESM.zip › Figure 4/4A/Raw Data/4A_EXP2/EXP2_STAT1_merge.tif]

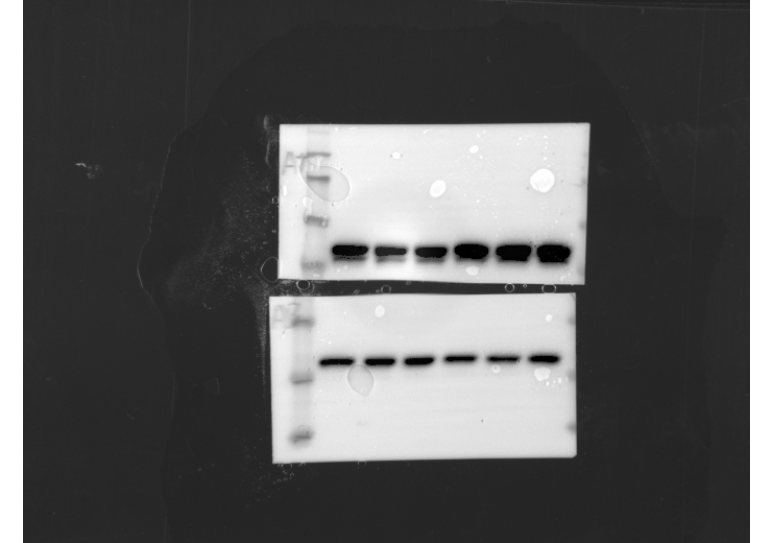

Supplement: Supplementary file 6 — Source data Fig. 4 [file 44319_2026_745_MOESM6_ESM.zip › Figure 4/4A/Raw Data/4A_EXP2/EXP2_PTPN1_Bottom.tif]

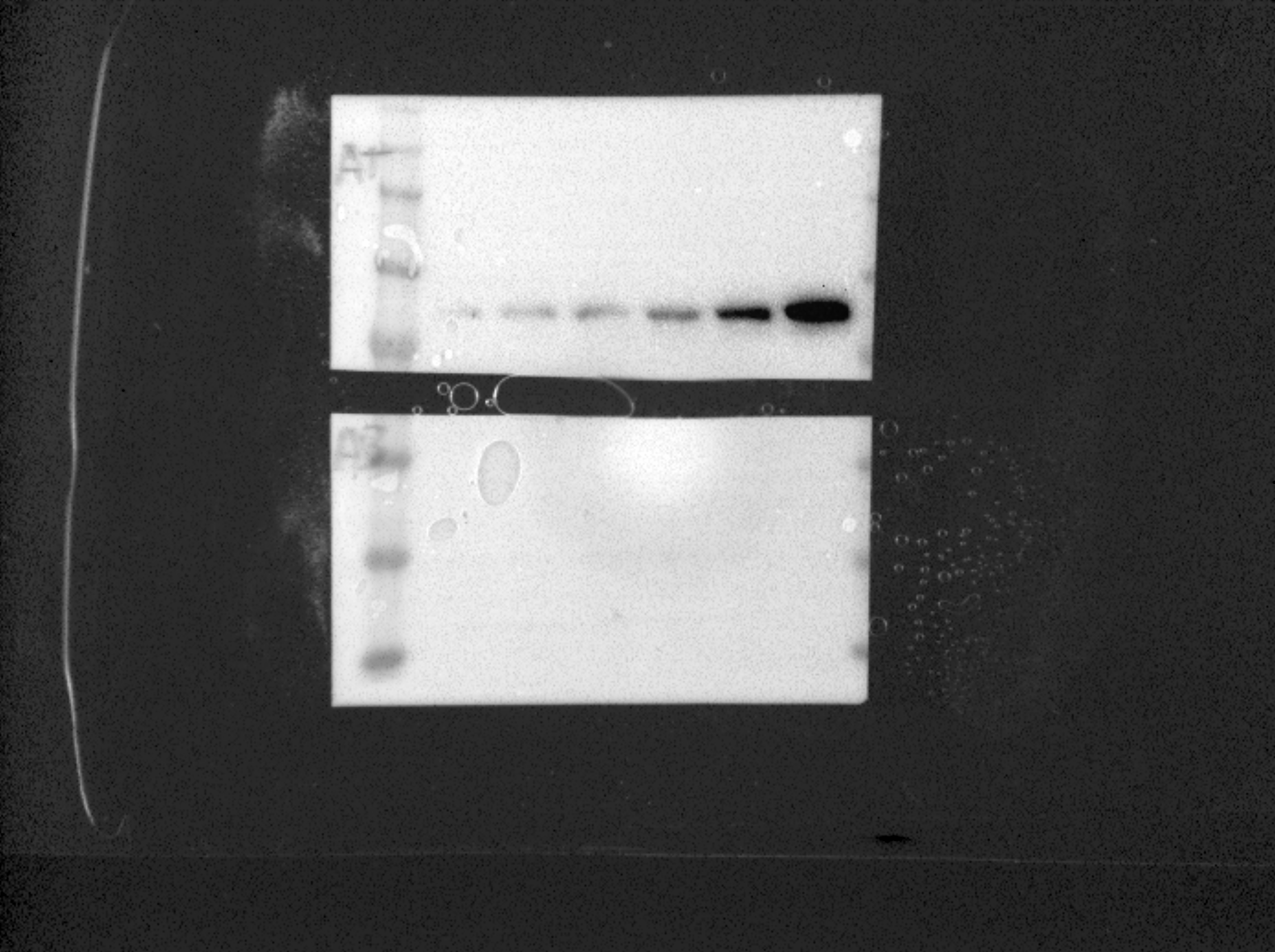

Supplement: Supplementary file 6 — Source data Fig. 4 [file 44319_2026_745_MOESM6_ESM.zip › Figure 4/4A/Raw Data/4A_EXP2/EXP2_PSTAT1.tif]

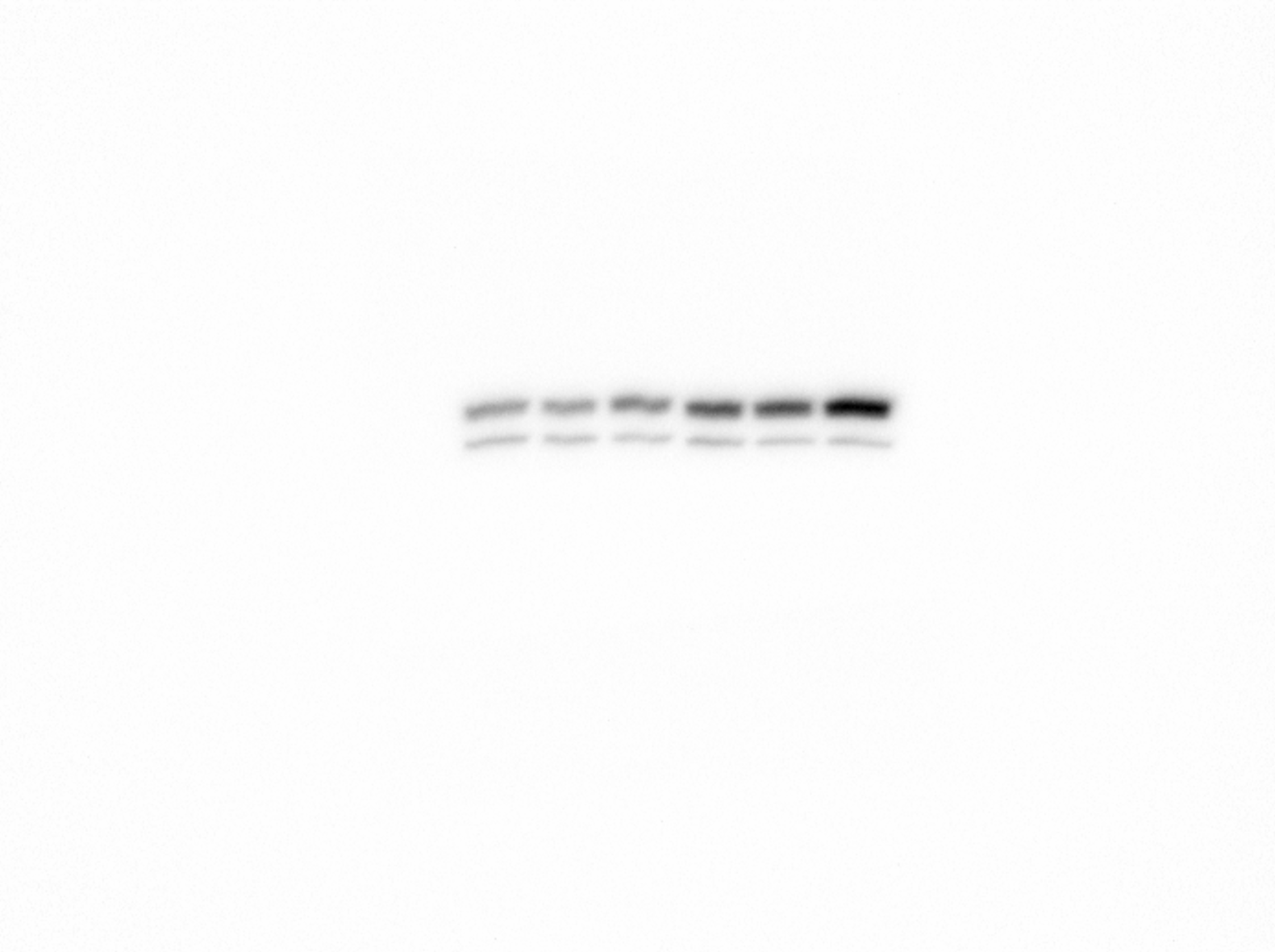

Supplement: Supplementary file 6 — Source data Fig. 4 [file 44319_2026_745_MOESM6_ESM.zip › Figure 4/4A/Raw Data/4A_EXP2/EXP2_PSTAT3.tif]

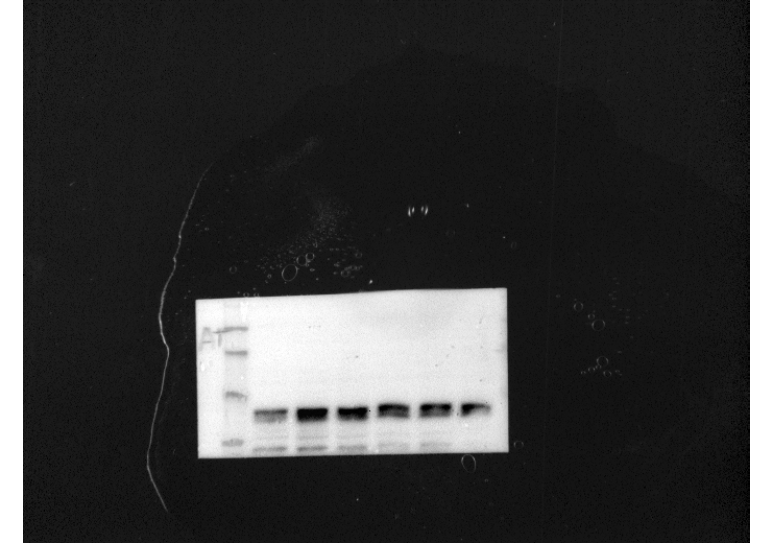

Supplement: Supplementary file 6 — Source data Fig. 4 [file 44319_2026_745_MOESM6_ESM.zip › Figure 4/4A/Raw Data/4A_EXP2/EXP2_STAT2_MERGE.tif]

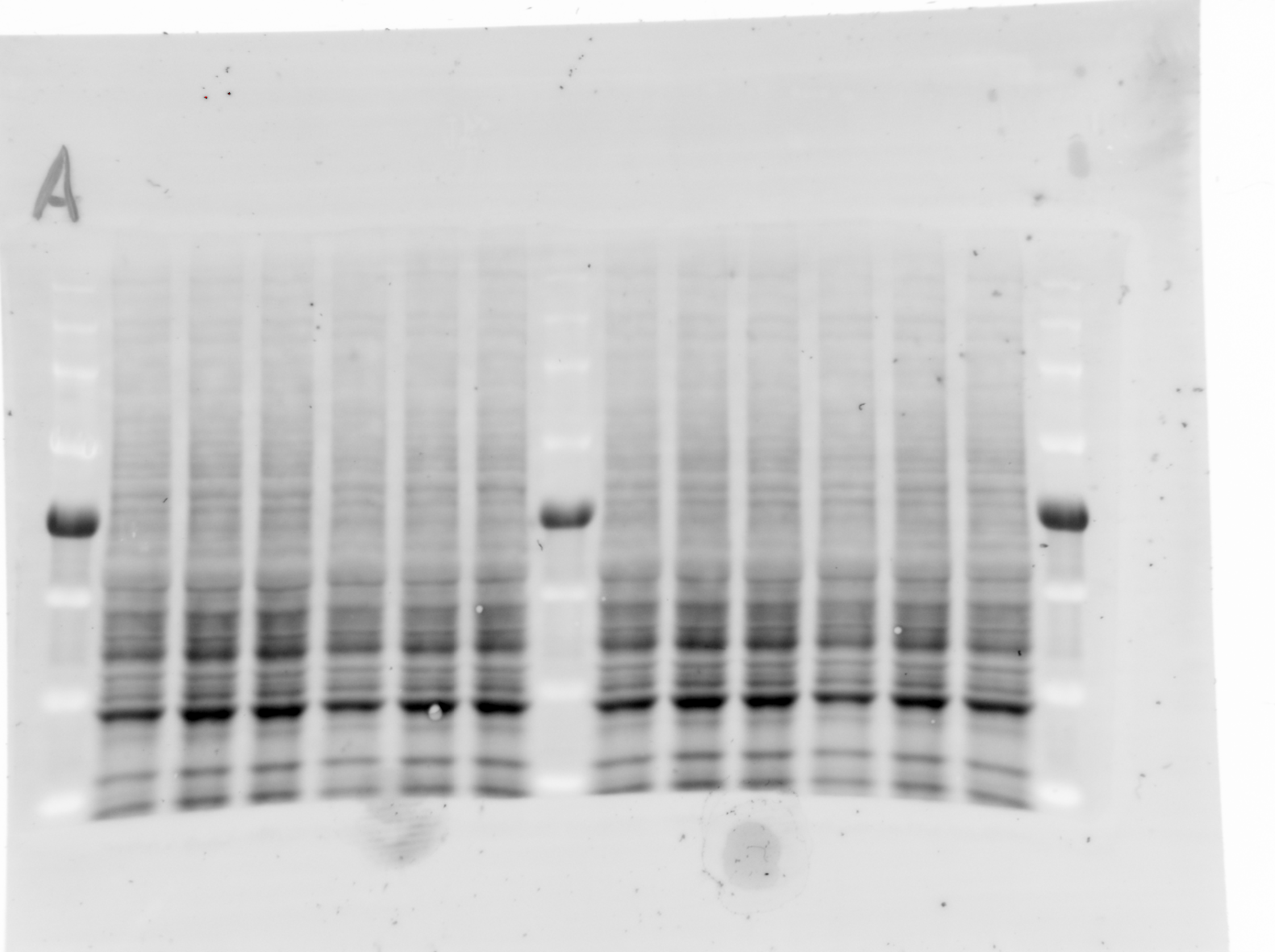

Supplement: Supplementary file 6 — Source data Fig. 4 [file 44319_2026_745_MOESM6_ESM.zip › Figure 4/4A/Raw Data/4A_EXP2/EXP2_Total Protein Loading Control for pSTAT1 (Left)_pSTAT3 (Right).tif]

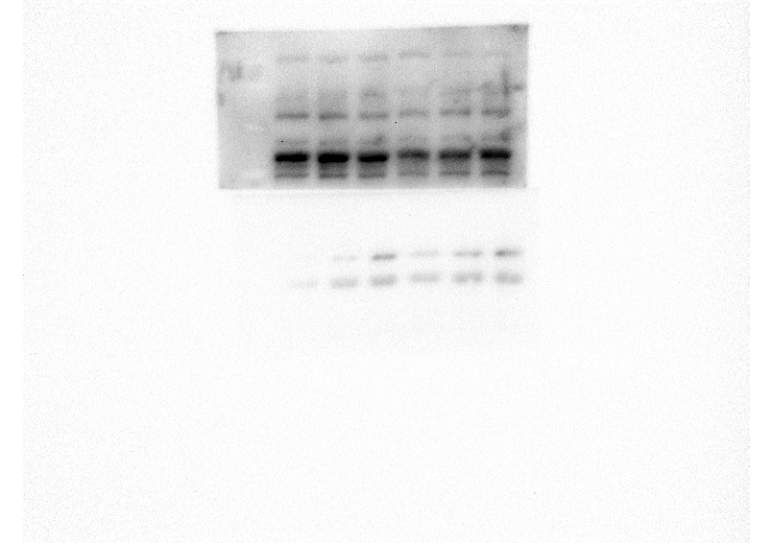

Supplement: Supplementary file 6 — Source data Fig. 4 [file 44319_2026_745_MOESM6_ESM.zip › Figure 4/4A/Raw Data/4A_EXP2/EXP2_PSTAT2_Low Exposure.tif]

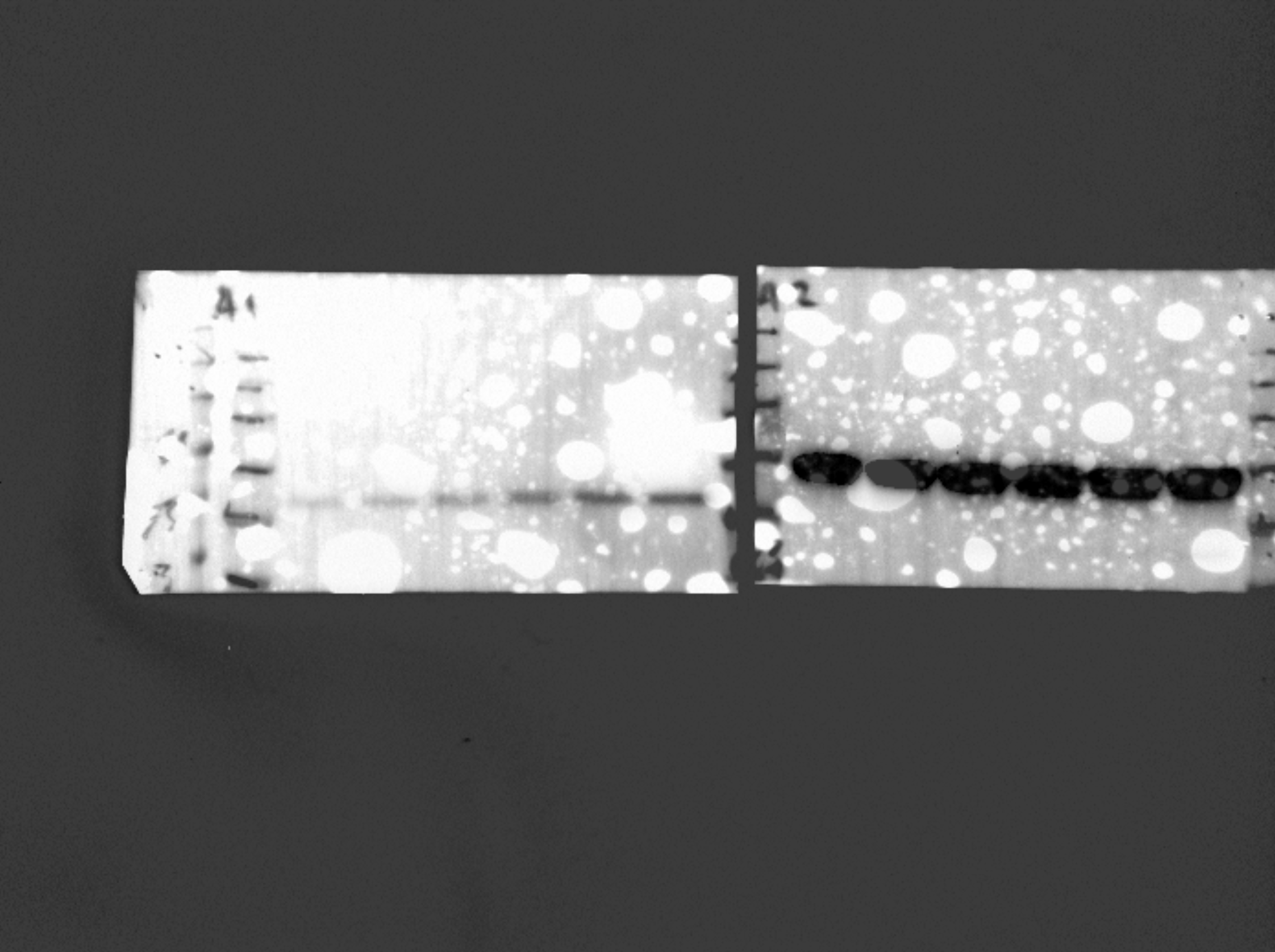

Supplement: Supplementary file 6 — Source data Fig. 4 [file 44319_2026_745_MOESM6_ESM.zip › Figure 4/4A/Raw Data/4A_EXP2/EXP2_pSTAT4(Left)_pSTAT5(Right)_Merge with Ladder.tif]

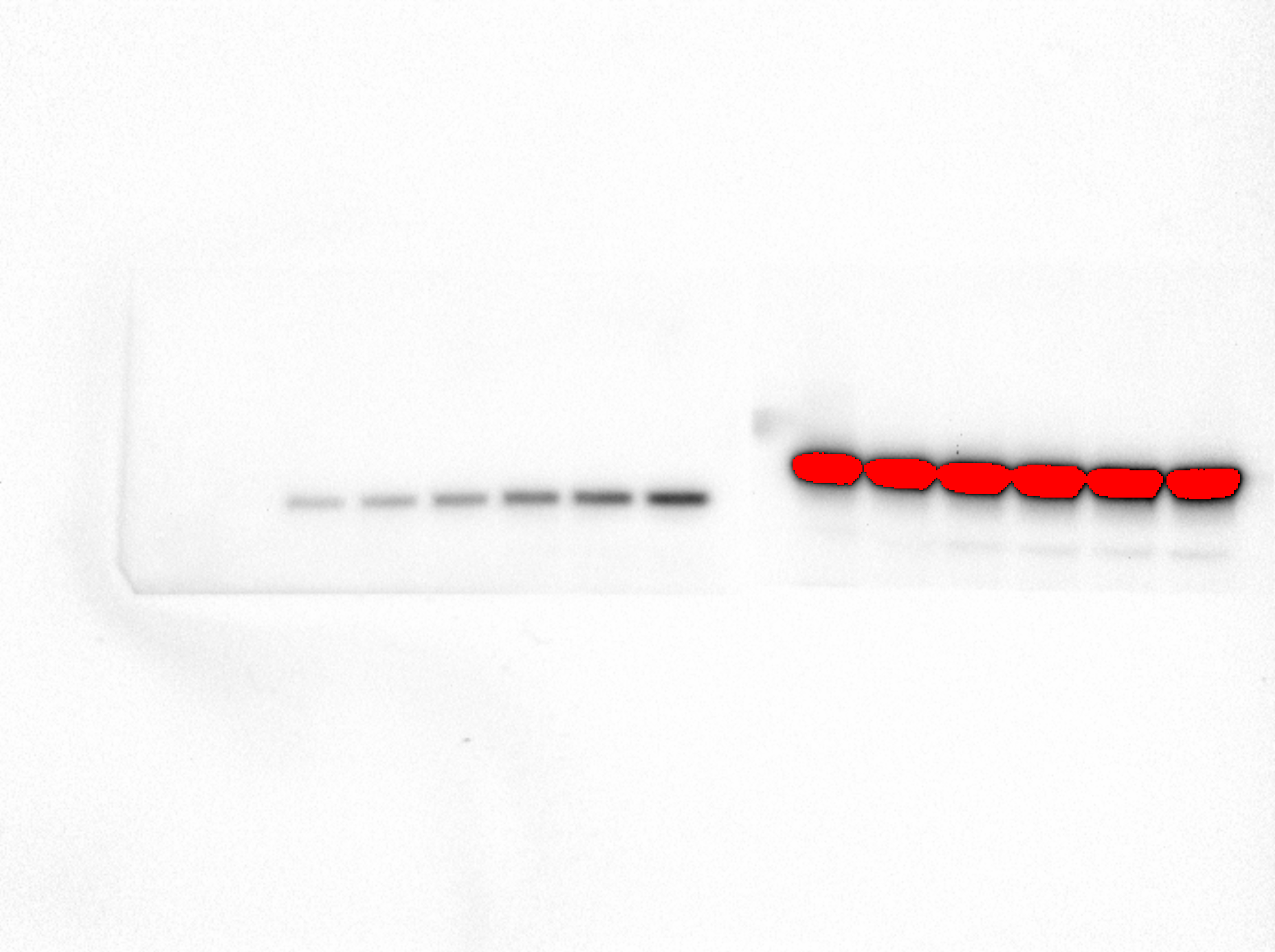

Supplement: Supplementary file 6 — Source data Fig. 4 [file 44319_2026_745_MOESM6_ESM.zip › Figure 4/4A/Raw Data/4A_EXP2/EXP2_pSTAT4(Left)_pSTAT5(Right).tif]

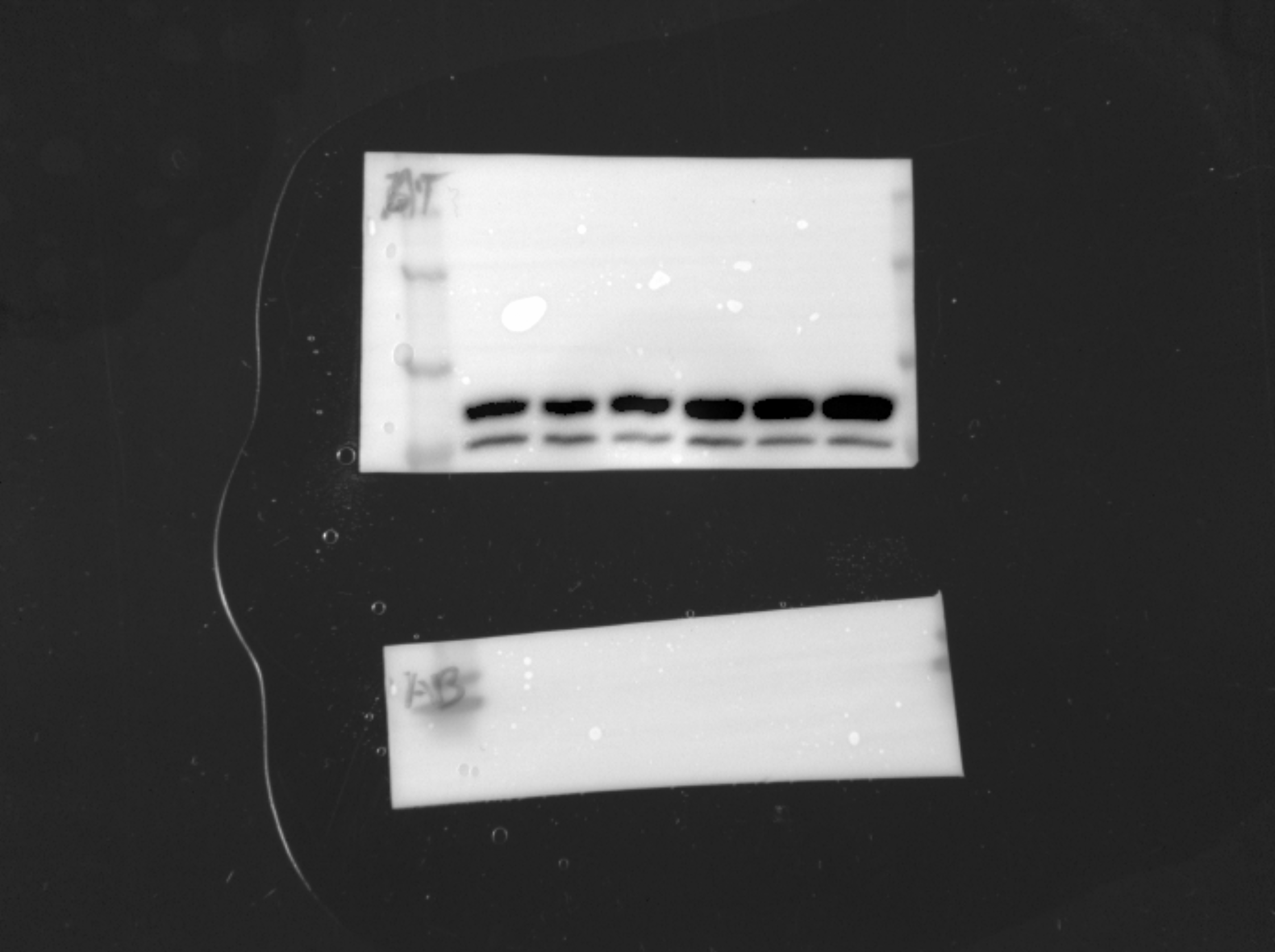

Supplement: Supplementary file 6 — Source data Fig. 4 [file 44319_2026_745_MOESM6_ESM.zip › Figure 4/4A/Raw Data/4A_EXP2/EXP2_pSTAT3_Merge with Ladder.tif]

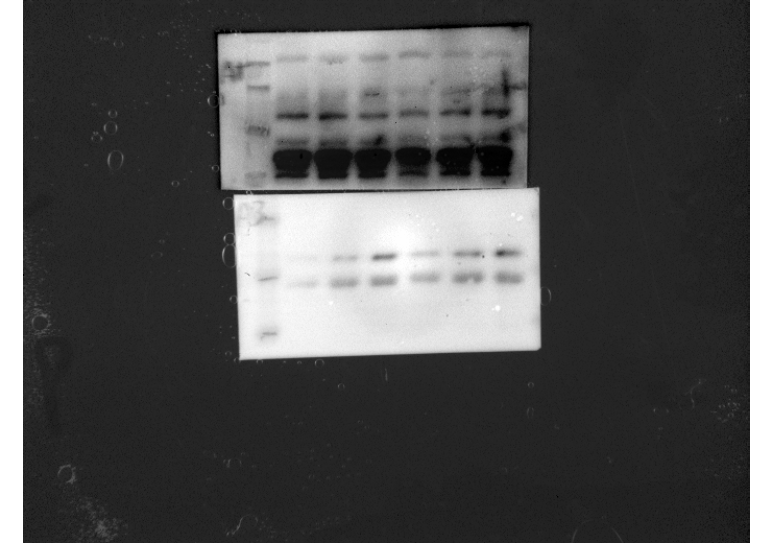

Supplement: Supplementary file 6 — Source data Fig. 4 [file 44319_2026_745_MOESM6_ESM.zip › Figure 4/4A/Raw Data/4A_EXP2/EXP2_PSTAT2(Top)_PTPN2(Bottom).tif]

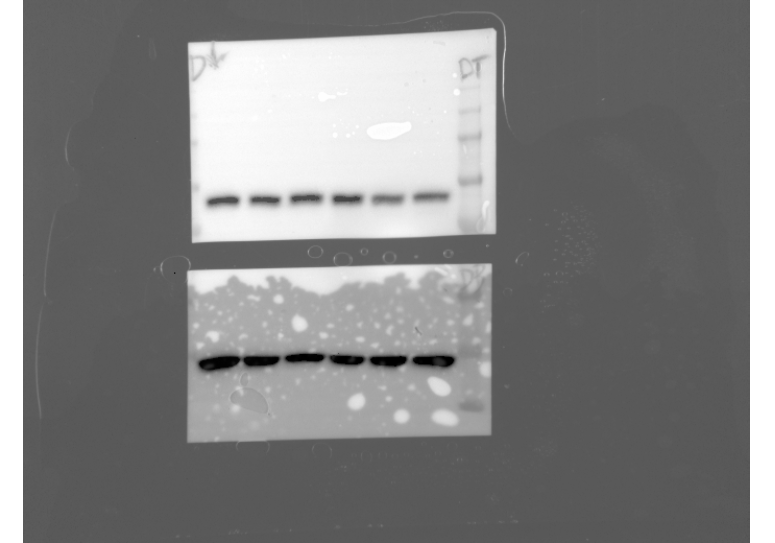

Supplement: Supplementary file 6 — Source data Fig. 4 [file 44319_2026_745_MOESM6_ESM.zip › Figure 4/4A/Raw Data/4A_EXP3/EXP3_PSTAT5(Top) Merge with Molecular Ladder.tif]

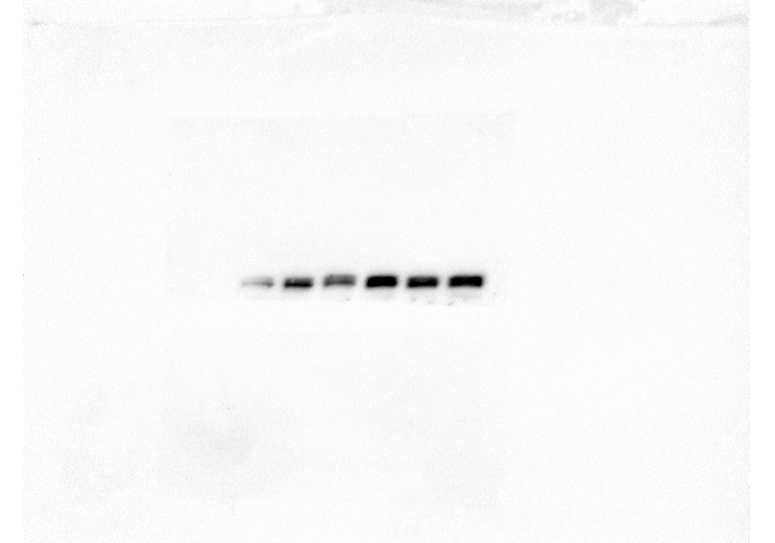

Supplement: Supplementary file 6 — Source data Fig. 4 [file 44319_2026_745_MOESM6_ESM.zip › Figure 4/4A/Raw Data/4A_EXP3/EXP3_PSTAT4.tif]

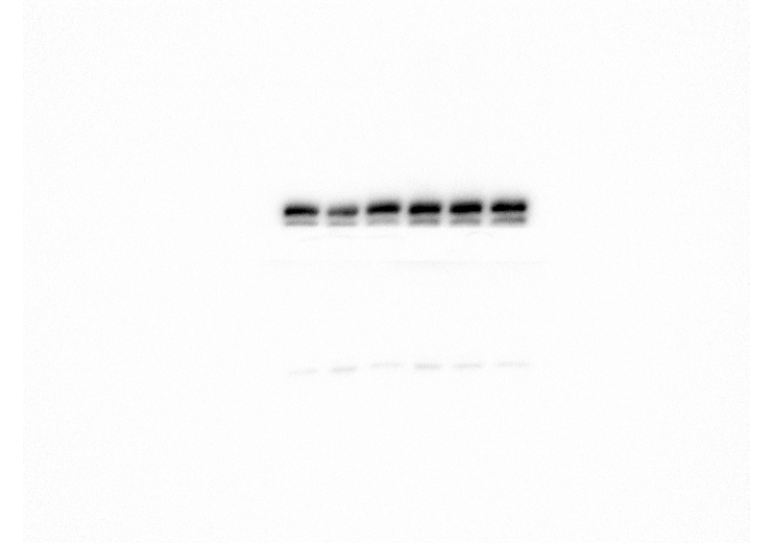

Supplement: Supplementary file 6 — Source data Fig. 4 [file 44319_2026_745_MOESM6_ESM.zip › Figure 4/4A/Raw Data/4A_EXP3/EXP3_TSTAT4.tif]

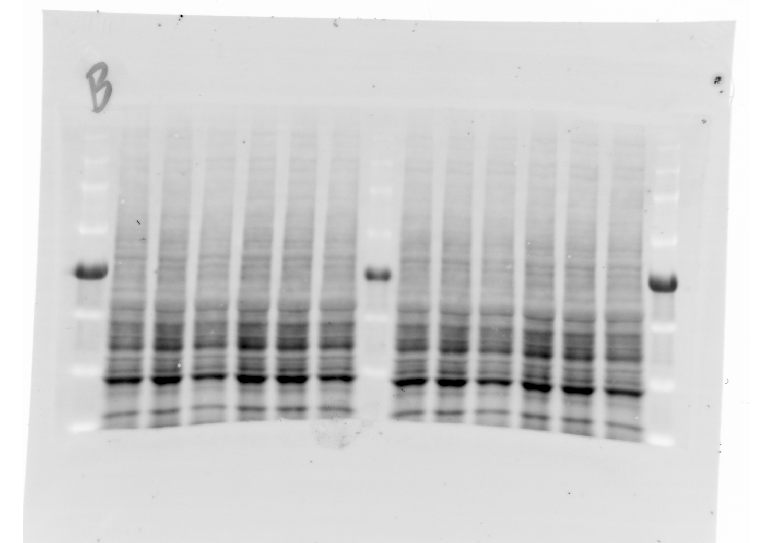

Supplement: Supplementary file 6 — Source data Fig. 4 [file 44319_2026_745_MOESM6_ESM.zip › Figure 4/4A/Raw Data/4A_EXP3/EXP3_Total Protein Loading Control_STAT4(Left)_STAT5(Right).tif]

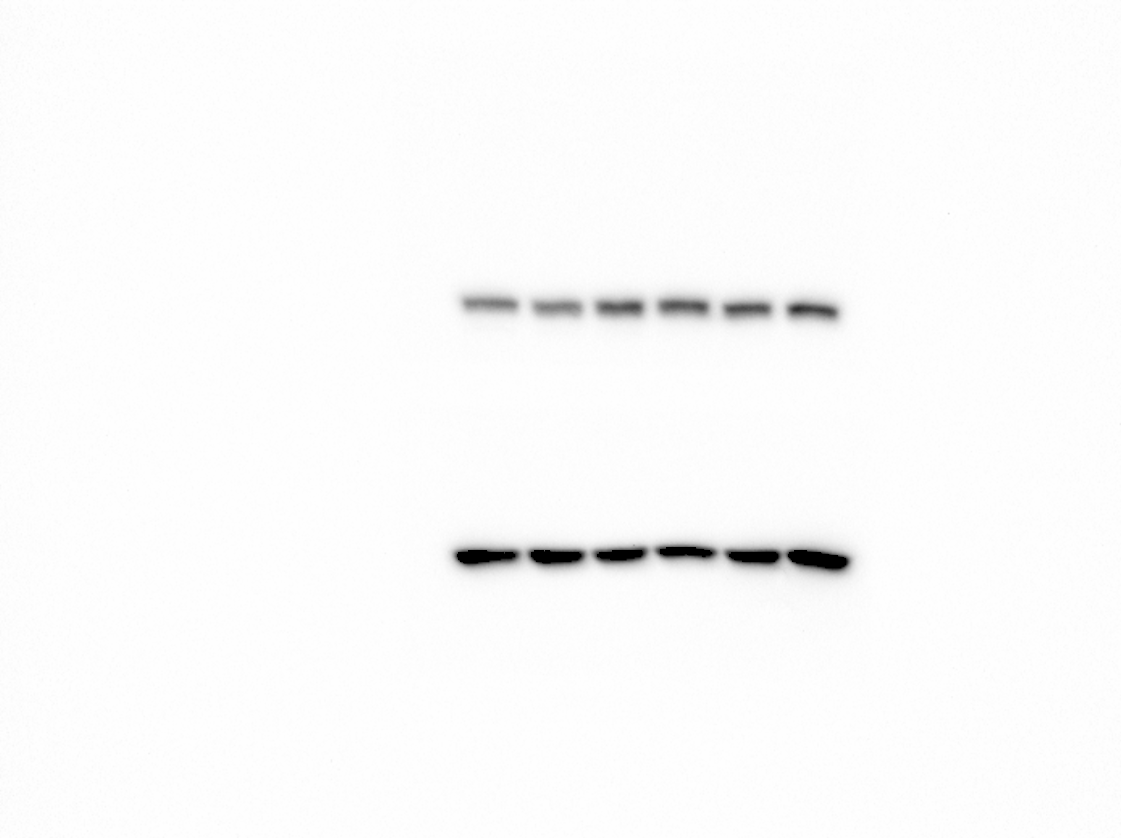

Supplement: Supplementary file 6 — Source data Fig. 4 [file 44319_2026_745_MOESM6_ESM.zip › Figure 4/4A/Raw Data/4A_EXP3/EXP3_PSTAT5(Top).tif]

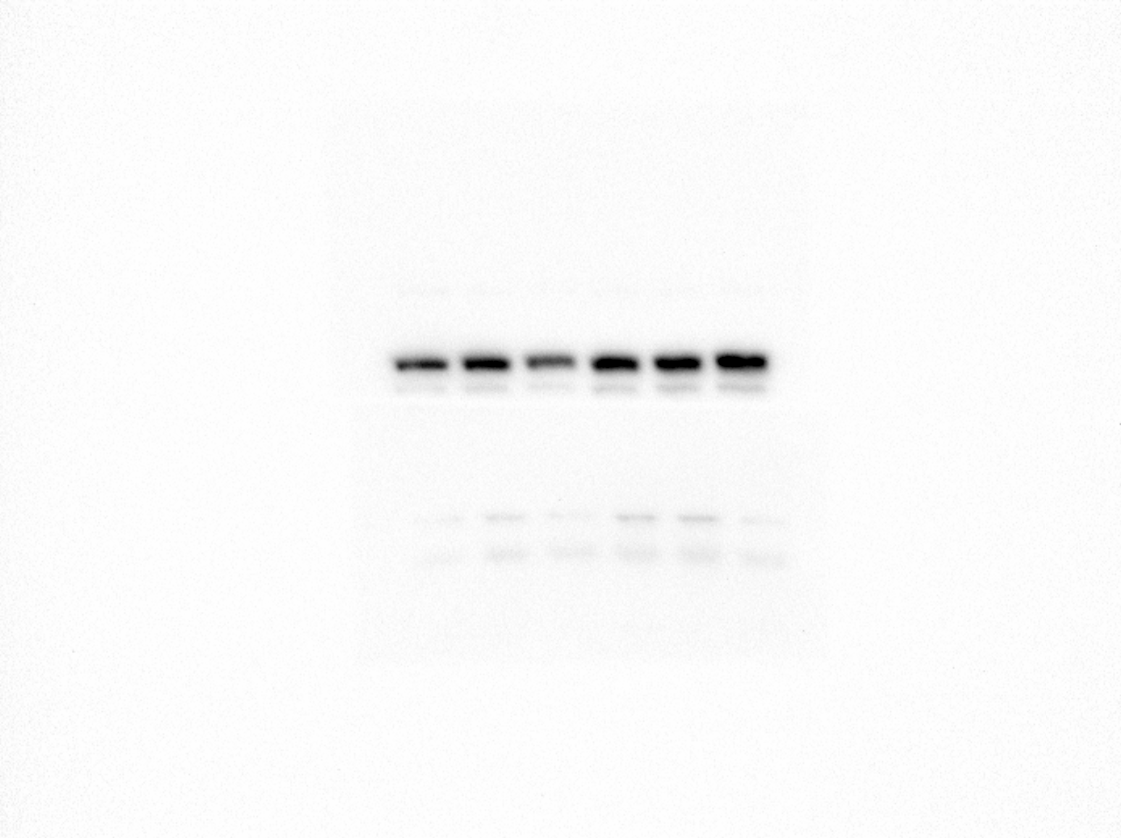

Supplement: Supplementary file 6 — Source data Fig. 4 [file 44319_2026_745_MOESM6_ESM.zip › Figure 4/4A/Raw Data/4A_EXP3/EXP3_PSTAT3.tif]

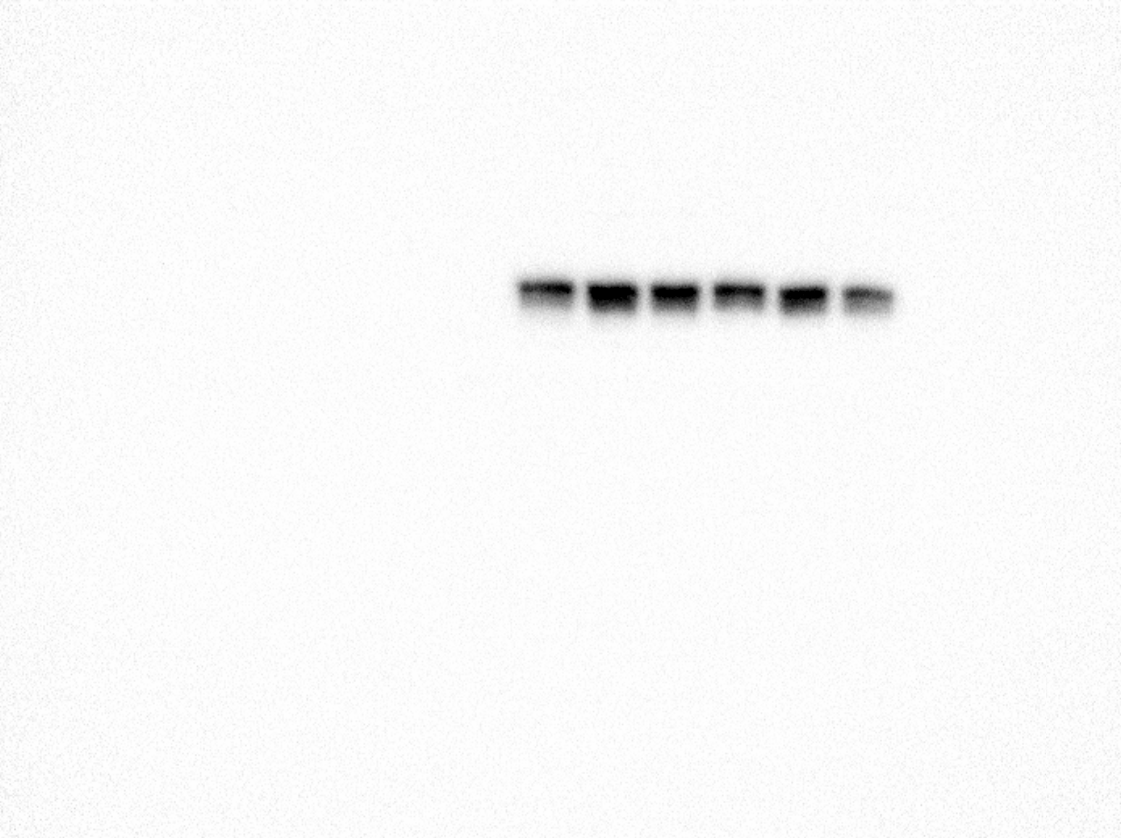

Supplement: Supplementary file 6 — Source data Fig. 4 [file 44319_2026_745_MOESM6_ESM.zip › Figure 4/4A/Raw Data/4A_EXP3/EXP3_TSTAT5.tif]

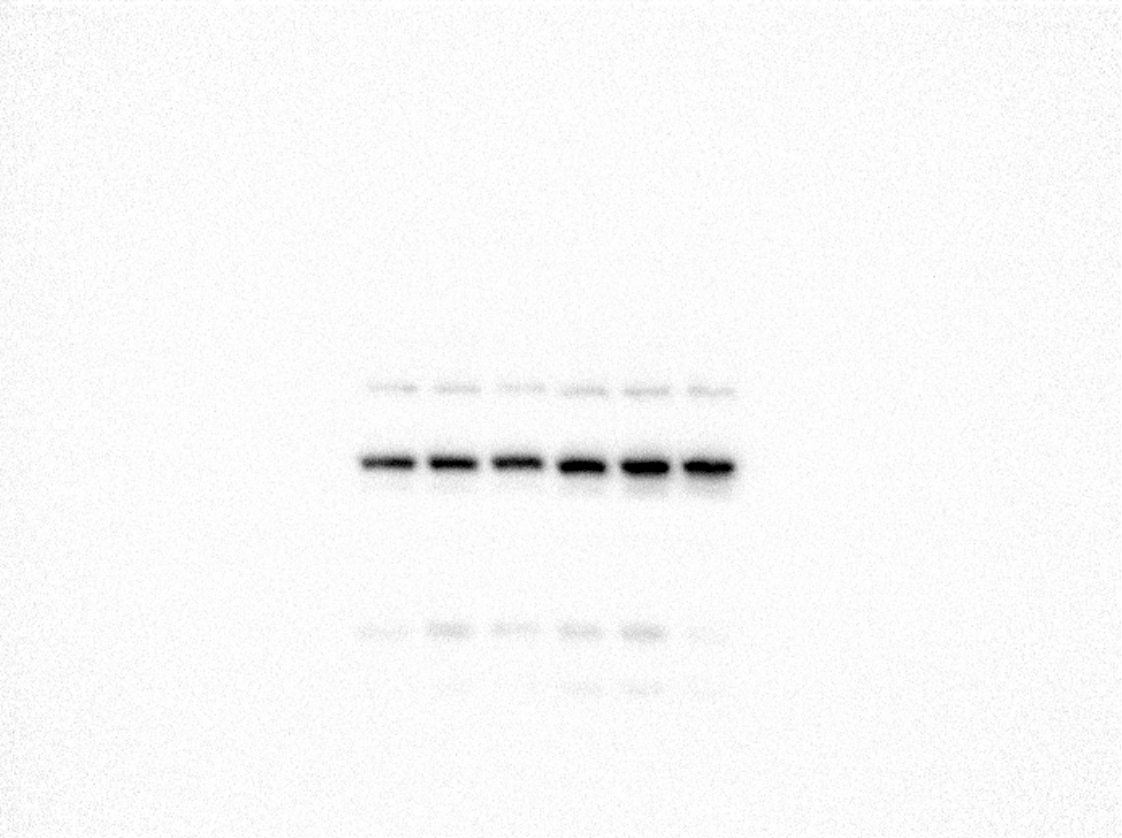

Supplement: Supplementary file 6 — Source data Fig. 4 [file 44319_2026_745_MOESM6_ESM.zip › Figure 4/4A/Raw Data/4A_EXP3/EXP3_TSTAT3.tif]

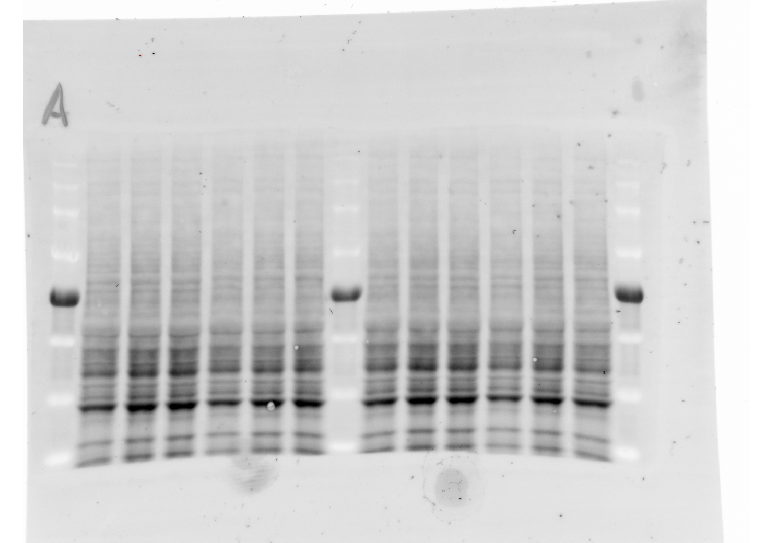

Supplement: Supplementary file 6 — Source data Fig. 4 [file 44319_2026_745_MOESM6_ESM.zip › Figure 4/4A/Raw Data/4A_EXP3/EXP3_Total Protein Loading Control_STAT1(Left)_STAT3(Right).tif]

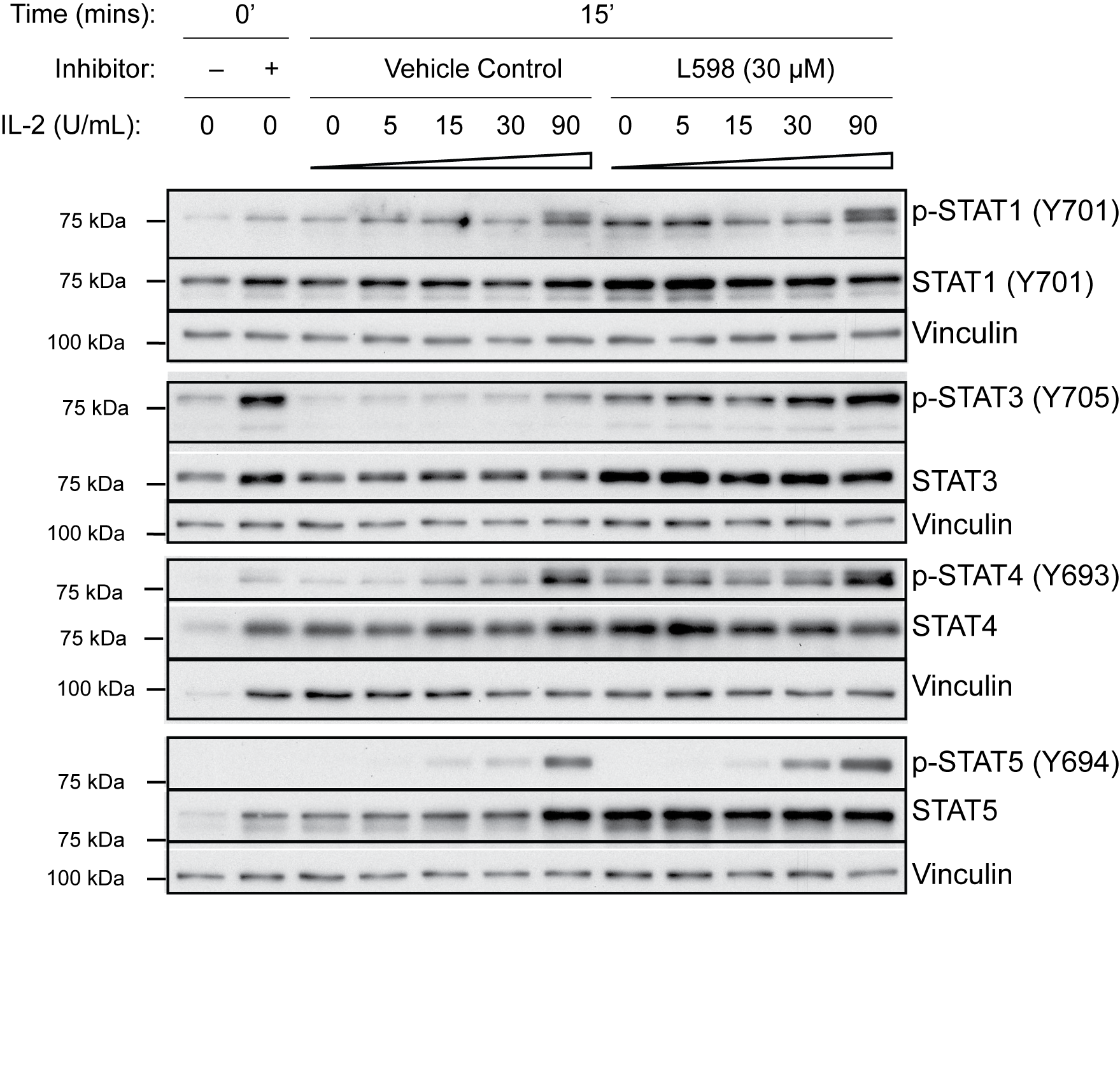

Supplement: Supplementary file 6 — Source data Fig. 4 [file 44319_2026_745_MOESM6_ESM.zip › Figure 4/4B/4B_JAK STAT.tif]

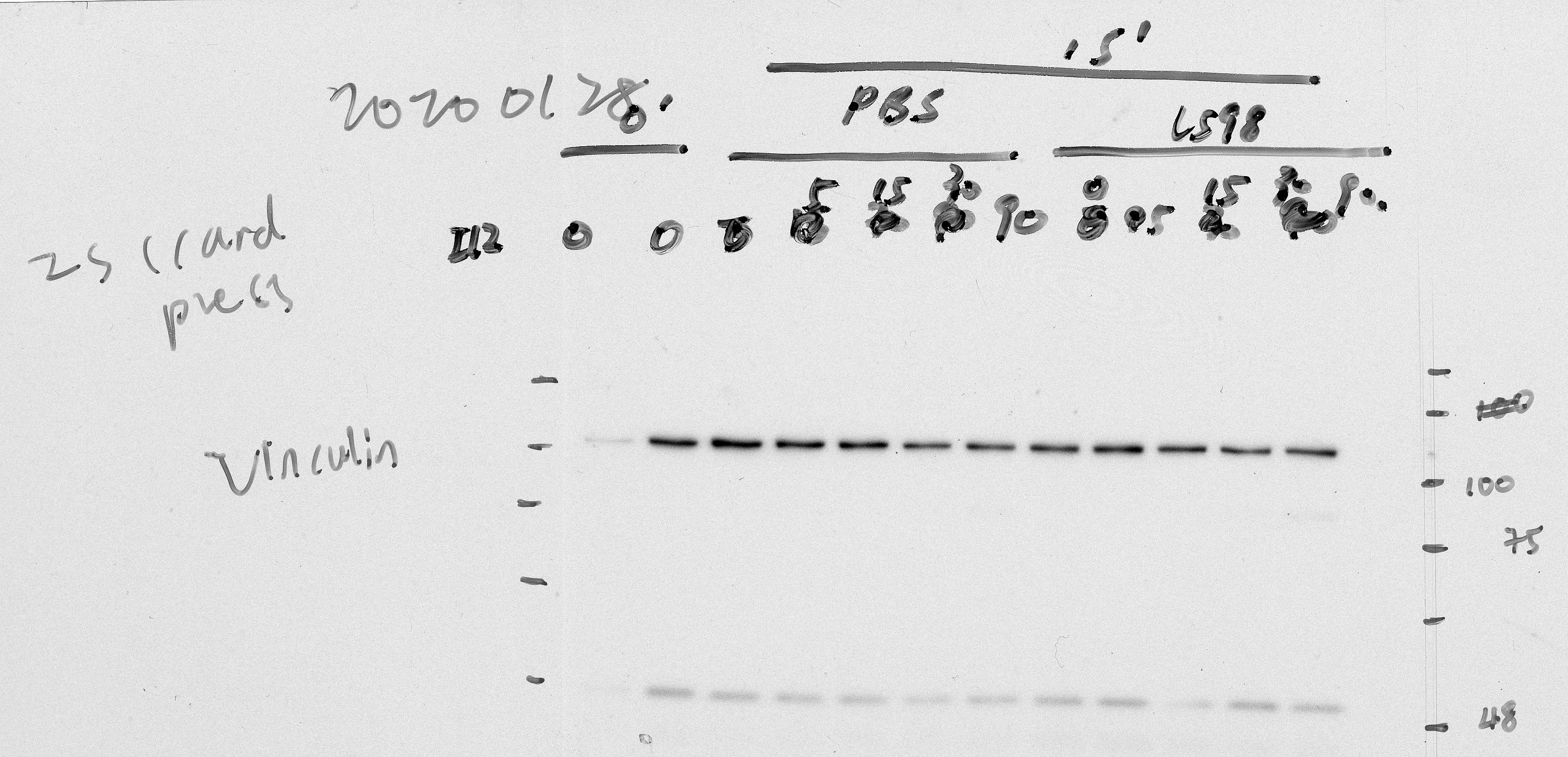

Supplement: Supplementary file 6 — Source data Fig. 4 [file 44319_2026_745_MOESM6_ESM.zip › Figure 4/4B/Raw Data/EXP1/4B_EXP1_vinculin gel1_NK92_IL2_15min010_STAT4.jpg]

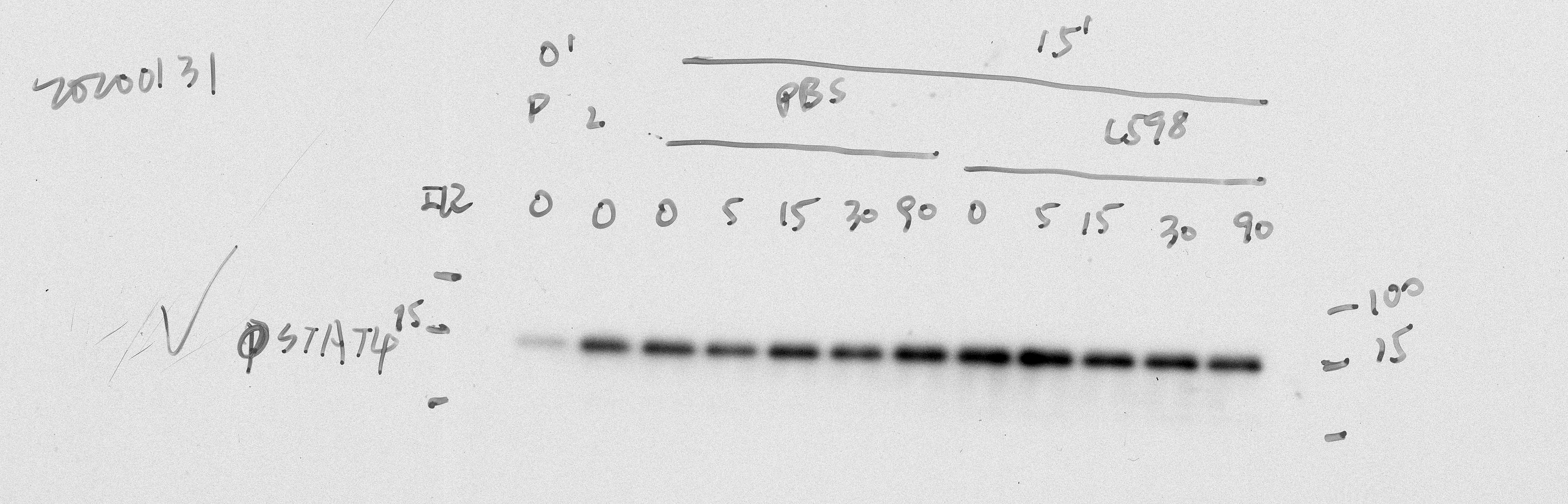

Supplement: Supplementary file 6 — Source data Fig. 4 [file 44319_2026_745_MOESM6_ESM.zip › Figure 4/4B/Raw Data/EXP1/4B_EXP1_Total STAT4_IL2_15mins_NK92002.jpg]

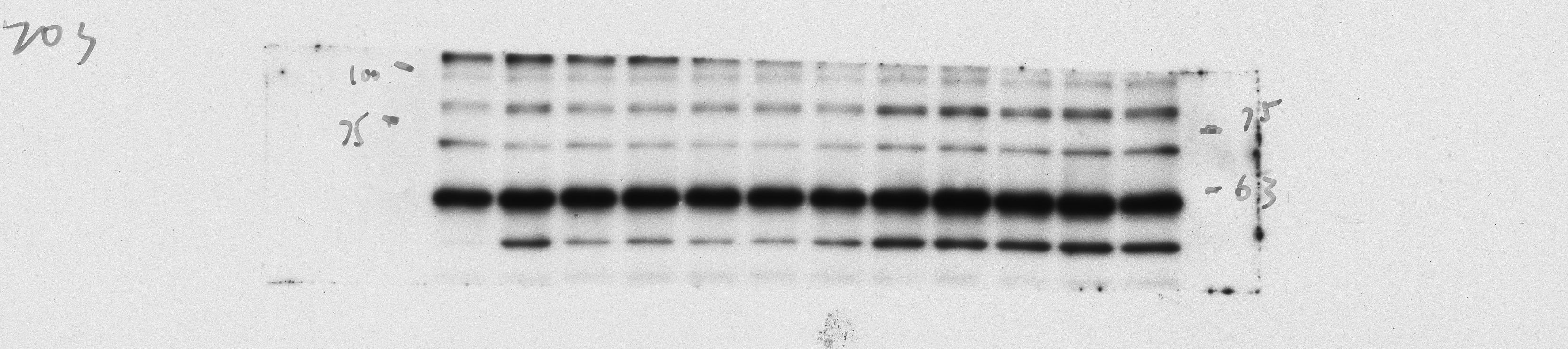

Supplement: Supplementary file 6 — Source data Fig. 4 [file 44319_2026_745_MOESM6_ESM.zip › Figure 4/4B/Raw Data/EXP1/4B_EXP1_totalSTAT6_il2_15mins_NK92006.jpg]

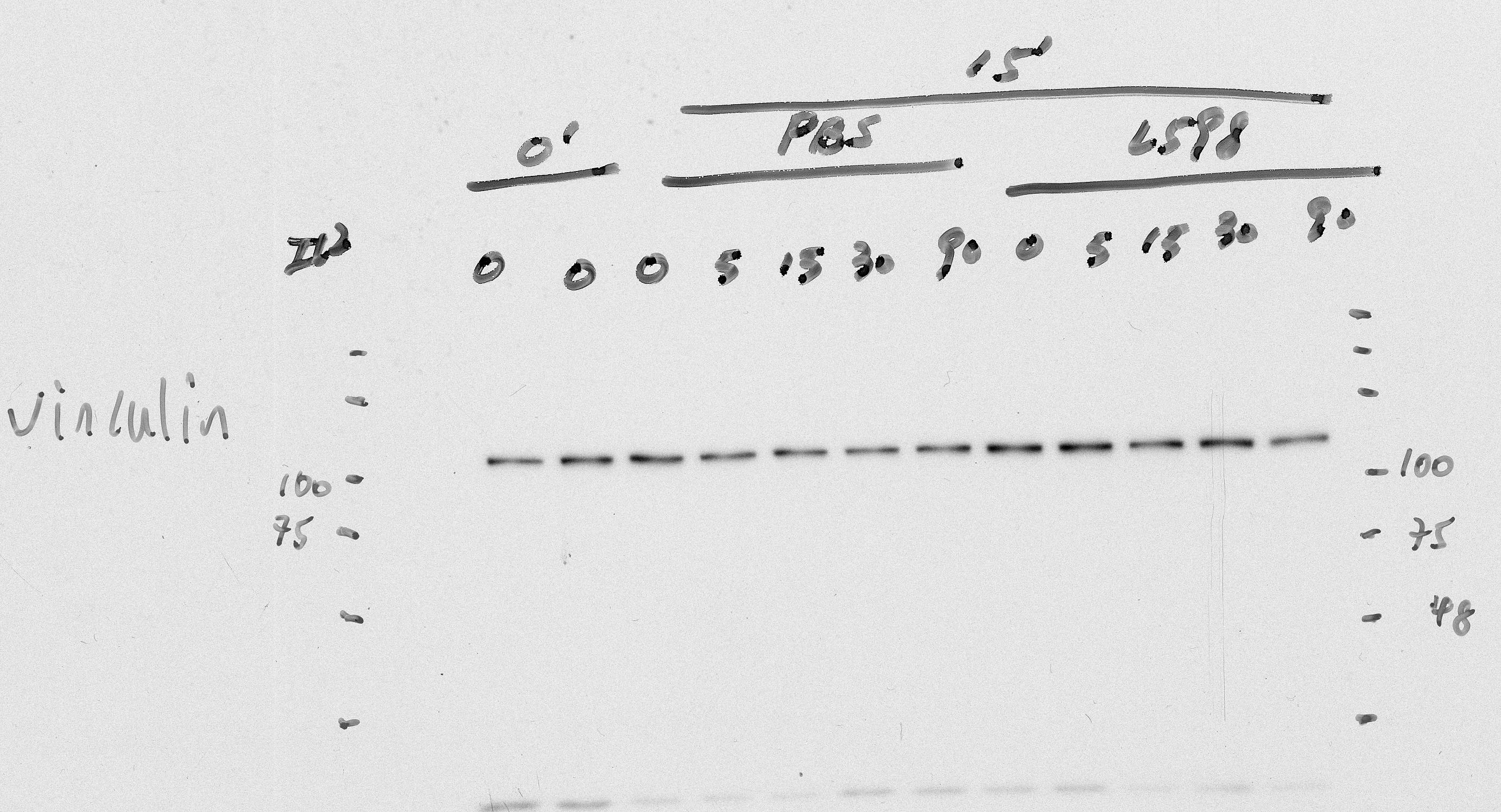

Supplement: Supplementary file 6 — Source data Fig. 4 [file 44319_2026_745_MOESM6_ESM.zip › Figure 4/4B/Raw Data/EXP1/4B_EXP1_vinculin gel3_NK92_IL2_15min012 STAT3 STAT5 AKT.jpg]

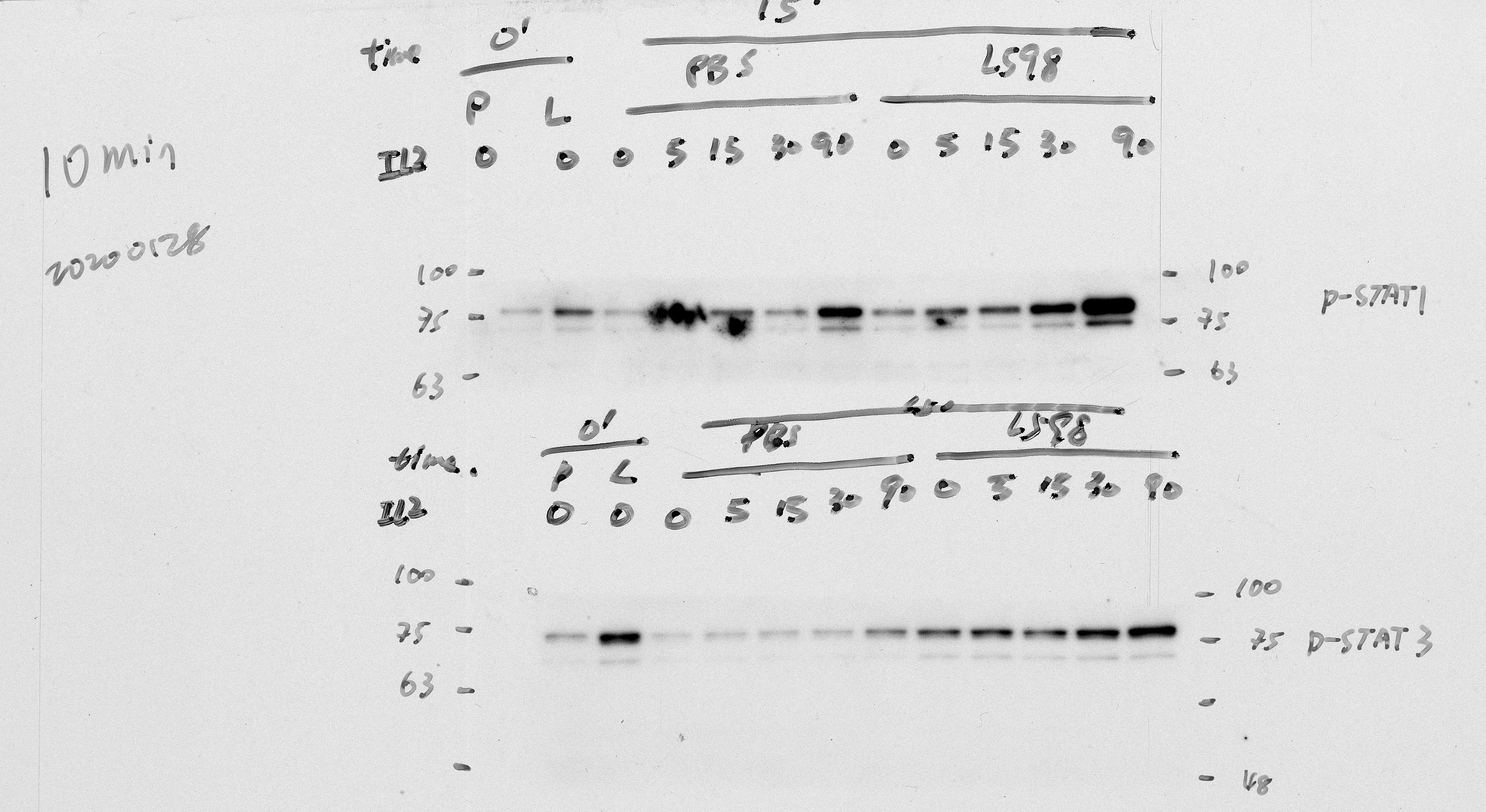

Supplement: Supplementary file 6 — Source data Fig. 4 [file 44319_2026_745_MOESM6_ESM.zip › Figure 4/4B/Raw Data/EXP1/4B_EXP1_pSTAT1_pSTAT3_NK92_IL2_15min014.jpg]

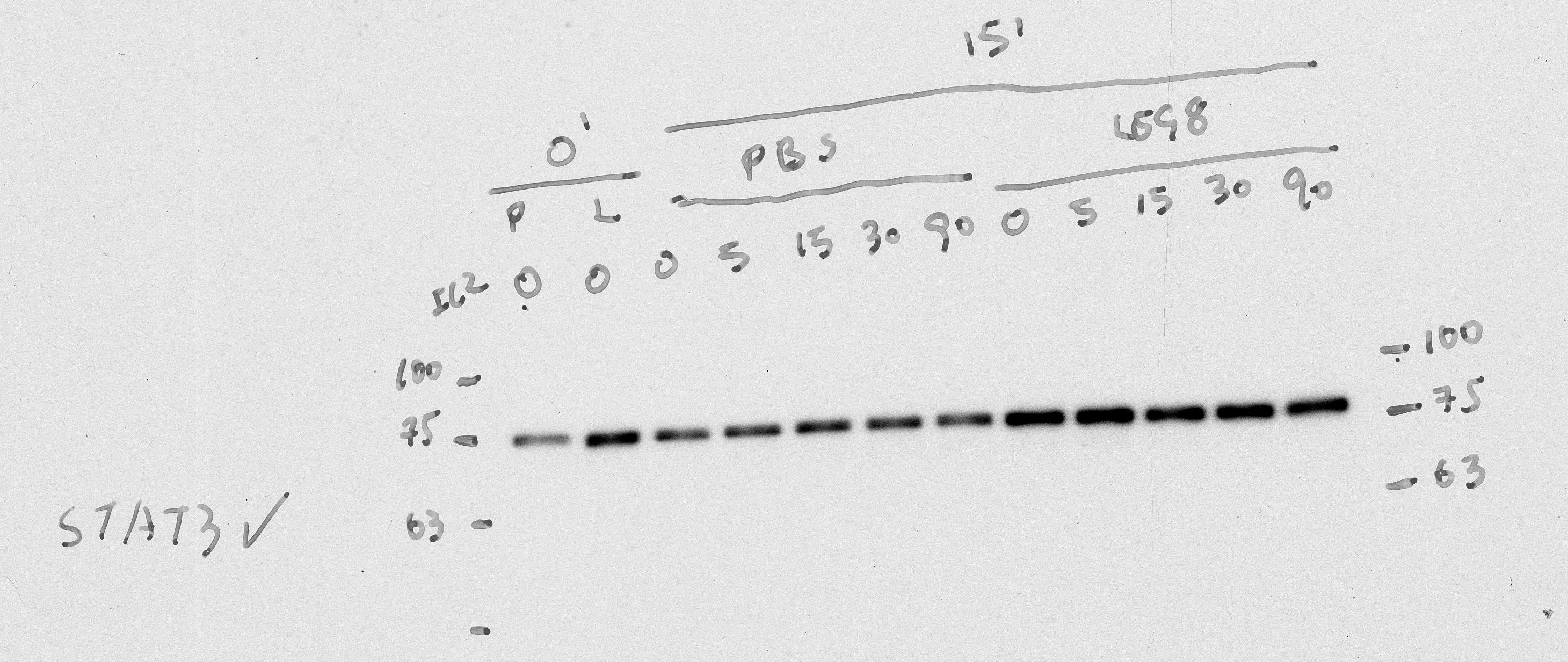

Supplement: Supplementary file 6 — Source data Fig. 4 [file 44319_2026_745_MOESM6_ESM.zip › Figure 4/4B/Raw Data/EXP1/4B_EXP1_total STAT3_NK92_IL2_15min004.jpg]

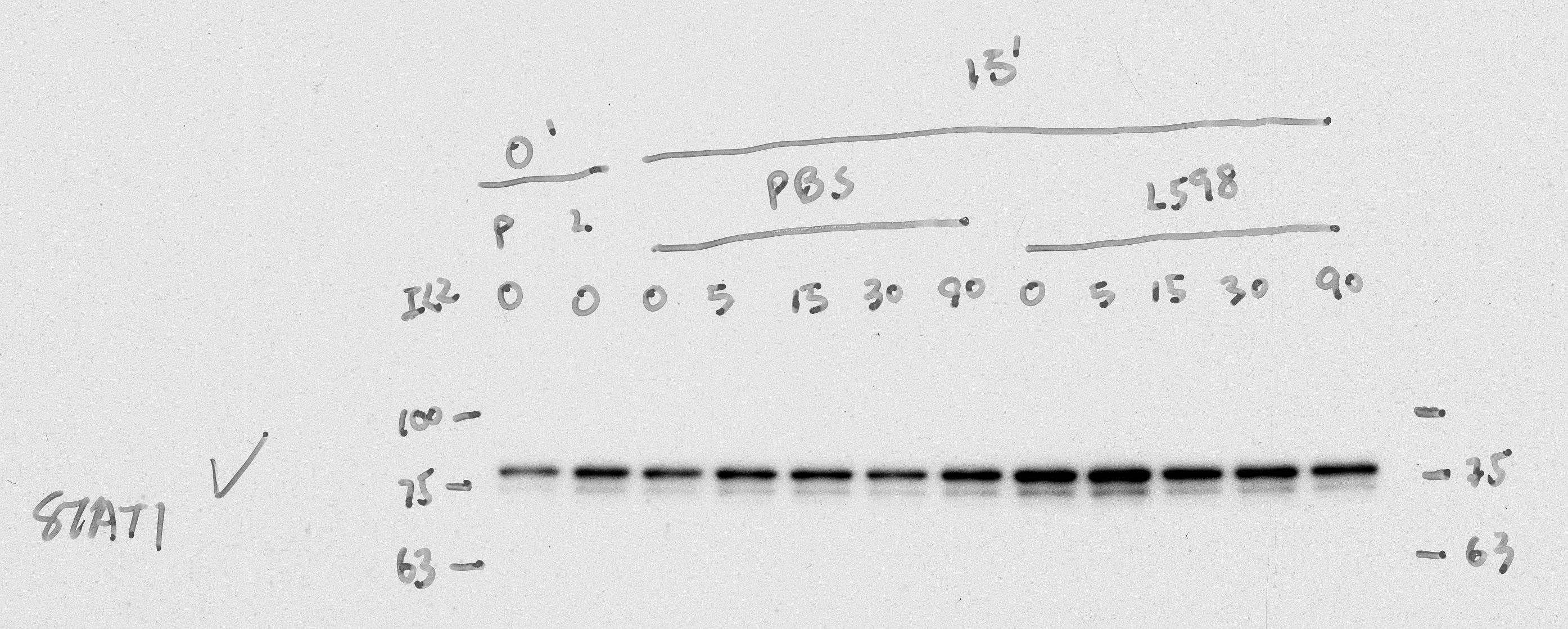

Supplement: Supplementary file 6 — Source data Fig. 4 [file 44319_2026_745_MOESM6_ESM.zip › Figure 4/4B/Raw Data/EXP1/4B_EXP1_total STAT1_NK92_IL2_15min003.jpg]

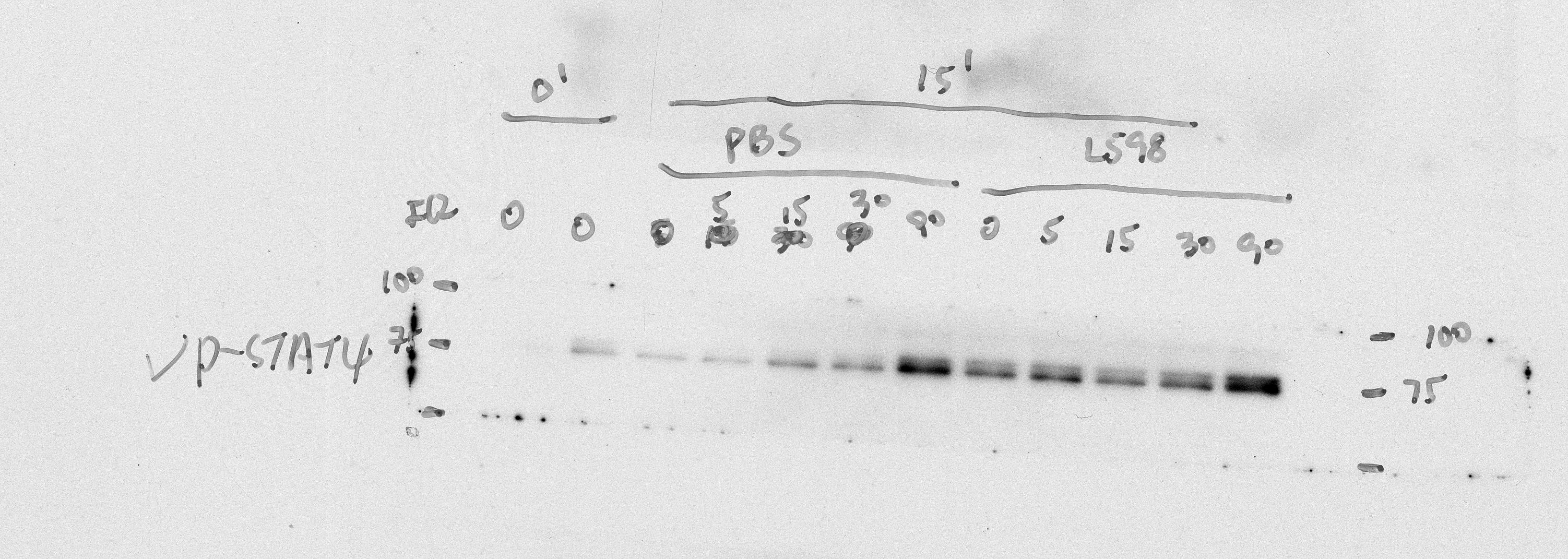

Supplement: Supplementary file 6 — Source data Fig. 4 [file 44319_2026_745_MOESM6_ESM.zip › Figure 4/4B/Raw Data/EXP1/4B_EXP1_p-STAT4_IL2_15mins_NK92011.jpg]

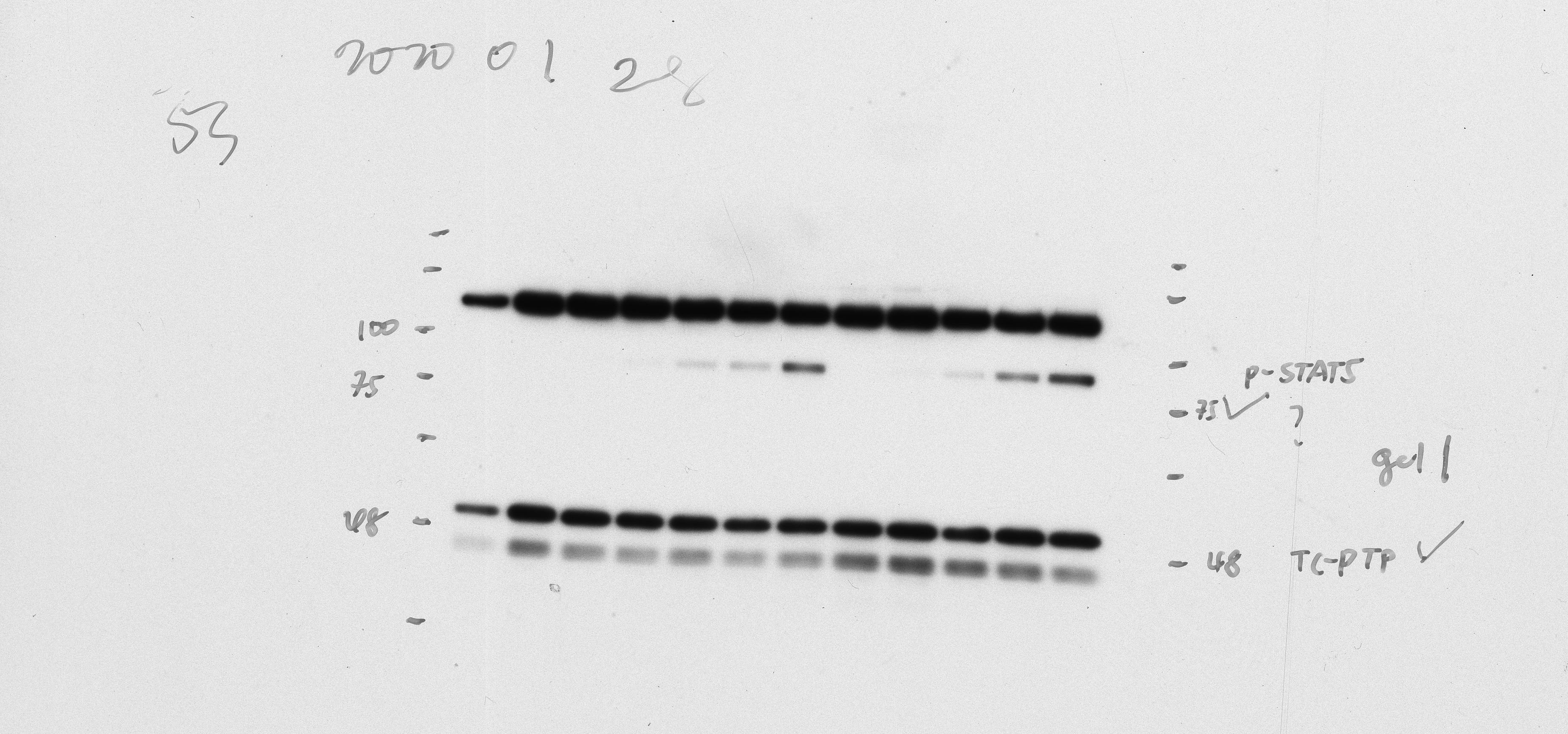

Supplement: Supplementary file 6 — Source data Fig. 4 [file 44319_2026_745_MOESM6_ESM.zip › Figure 4/4B/Raw Data/EXP1/4B_EXP1_pSTAT5_NK92_IL2_15min006.jpg]

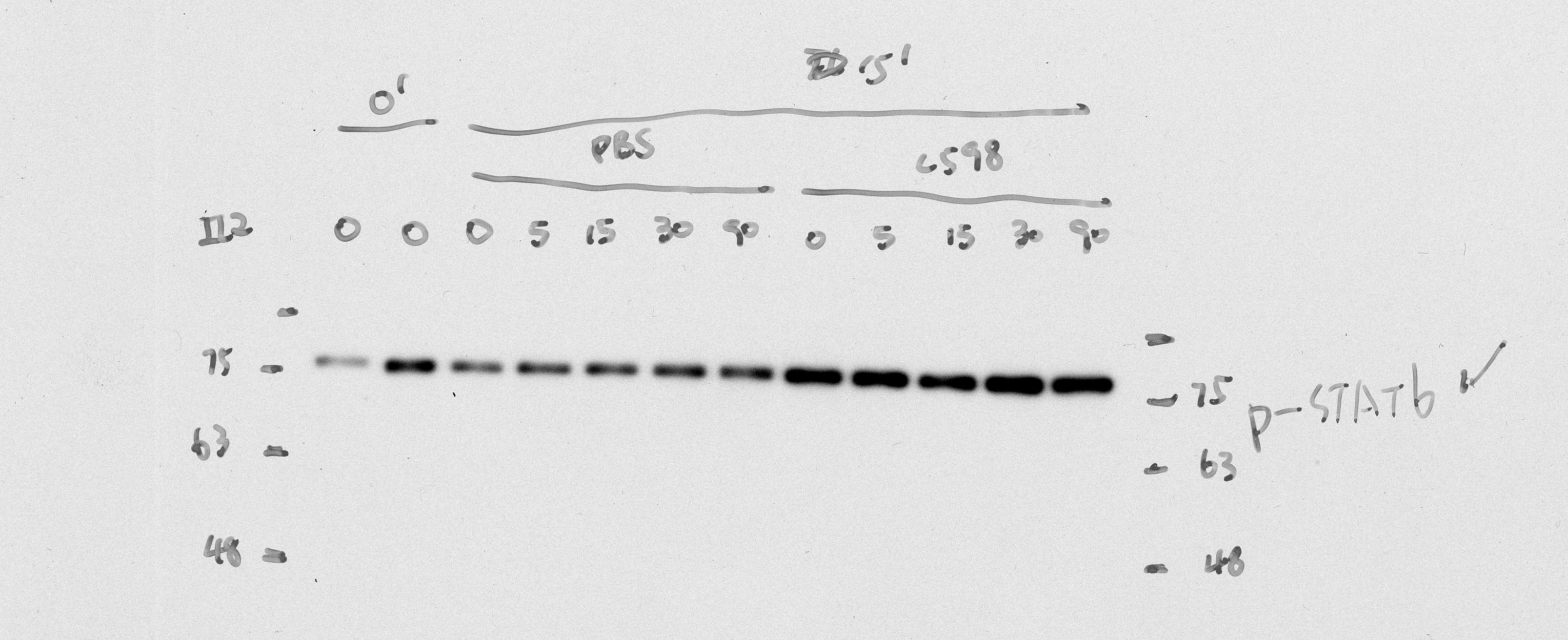

Supplement: Supplementary file 6 — Source data Fig. 4 [file 44319_2026_745_MOESM6_ESM.zip › Figure 4/4B/Raw Data/EXP1/4B_EXP1_p-STAT6_IL2_15mins_NK92007.jpg]

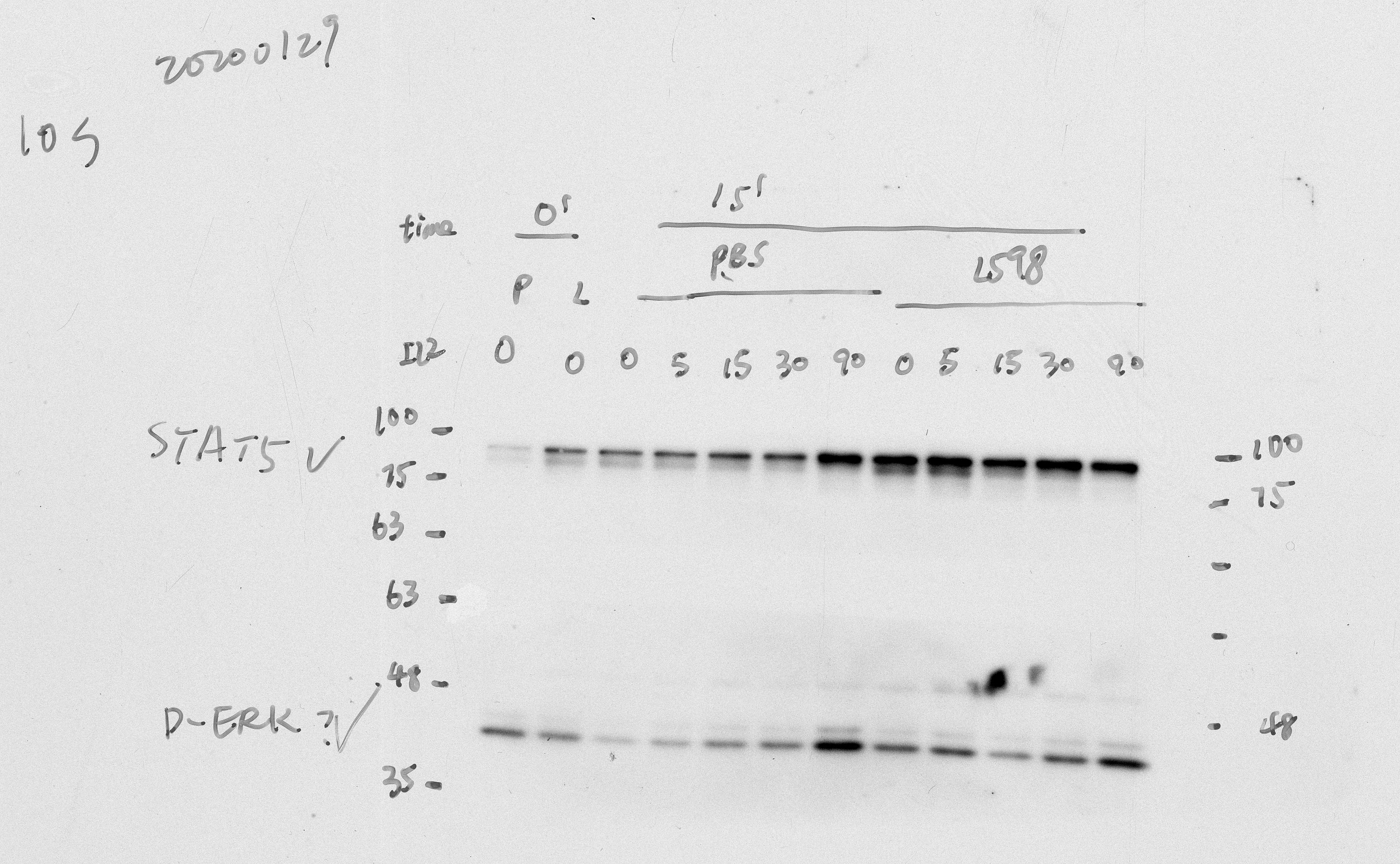

Supplement: Supplementary file 6 — Source data Fig. 4 [file 44319_2026_745_MOESM6_ESM.zip › Figure 4/4B/Raw Data/EXP1/4B_EXP1_Total STAT5_pERK1_2_NK92_IL2_15min001.jpg]

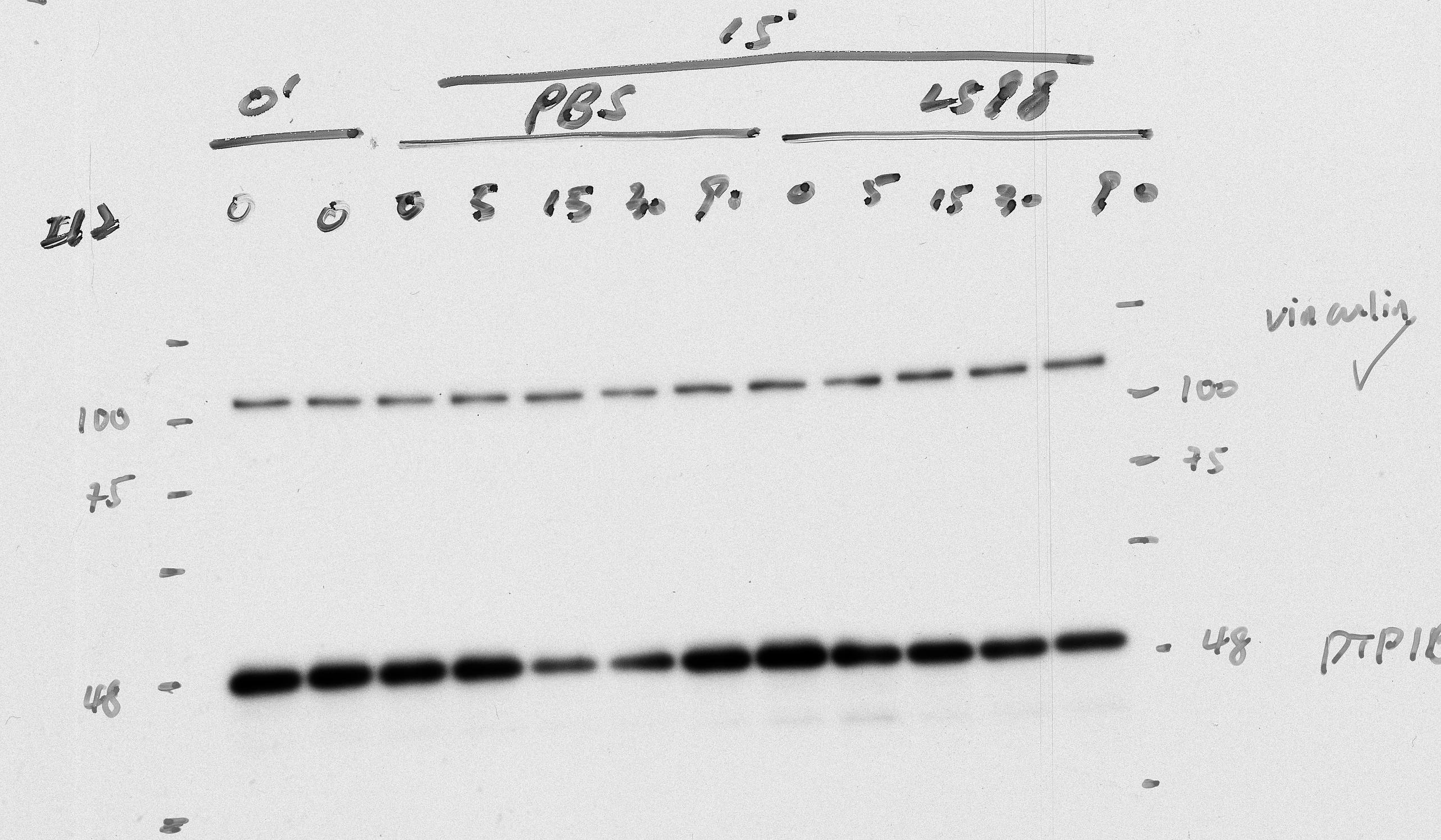

Supplement: Supplementary file 6 — Source data Fig. 4 [file 44319_2026_745_MOESM6_ESM.zip › Figure 4/4B/Raw Data/EXP1/4B_EXP1_vinculin gel2_NK92_IL2_15min011 STAT1.jpg]

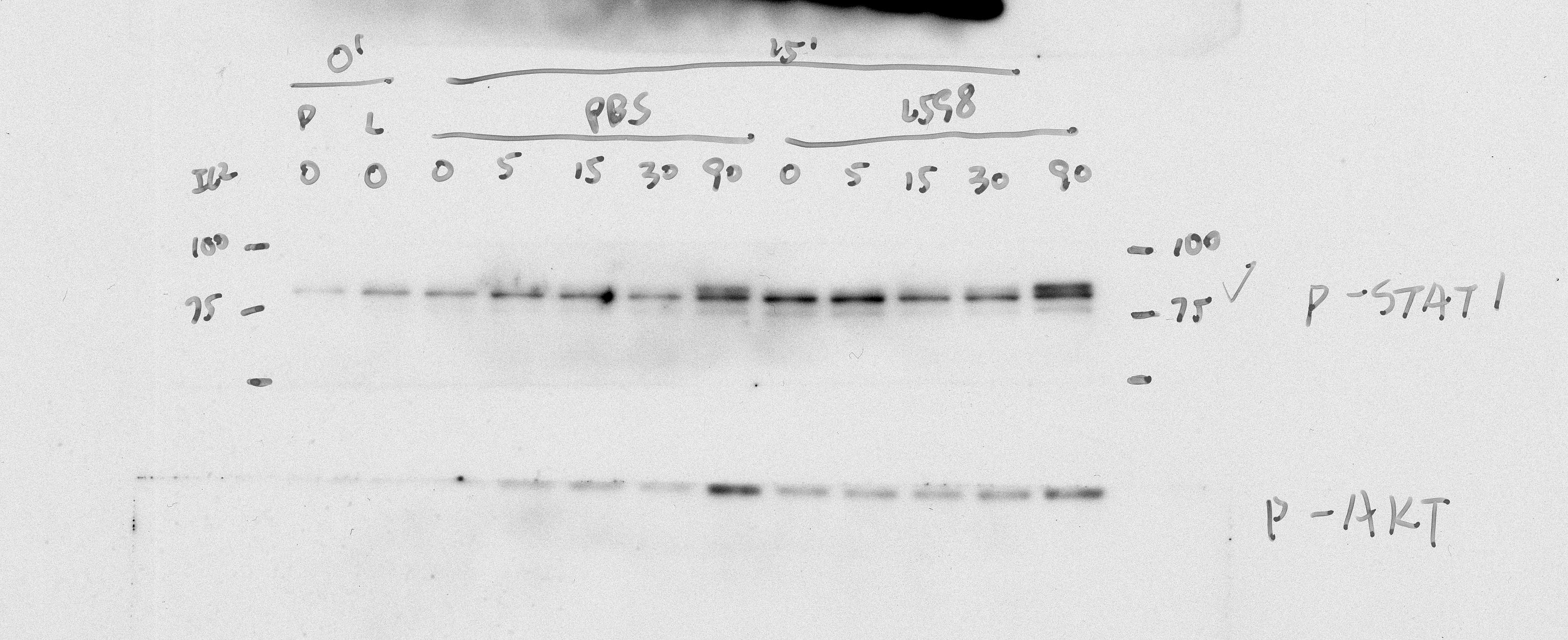

Supplement: Supplementary file 6 — Source data Fig. 4 [file 44319_2026_745_MOESM6_ESM.zip › Figure 4/4B/Raw Data/EXP1/4B_EXP1_p-STAT1_IL2_15mins_NK92008.jpg]

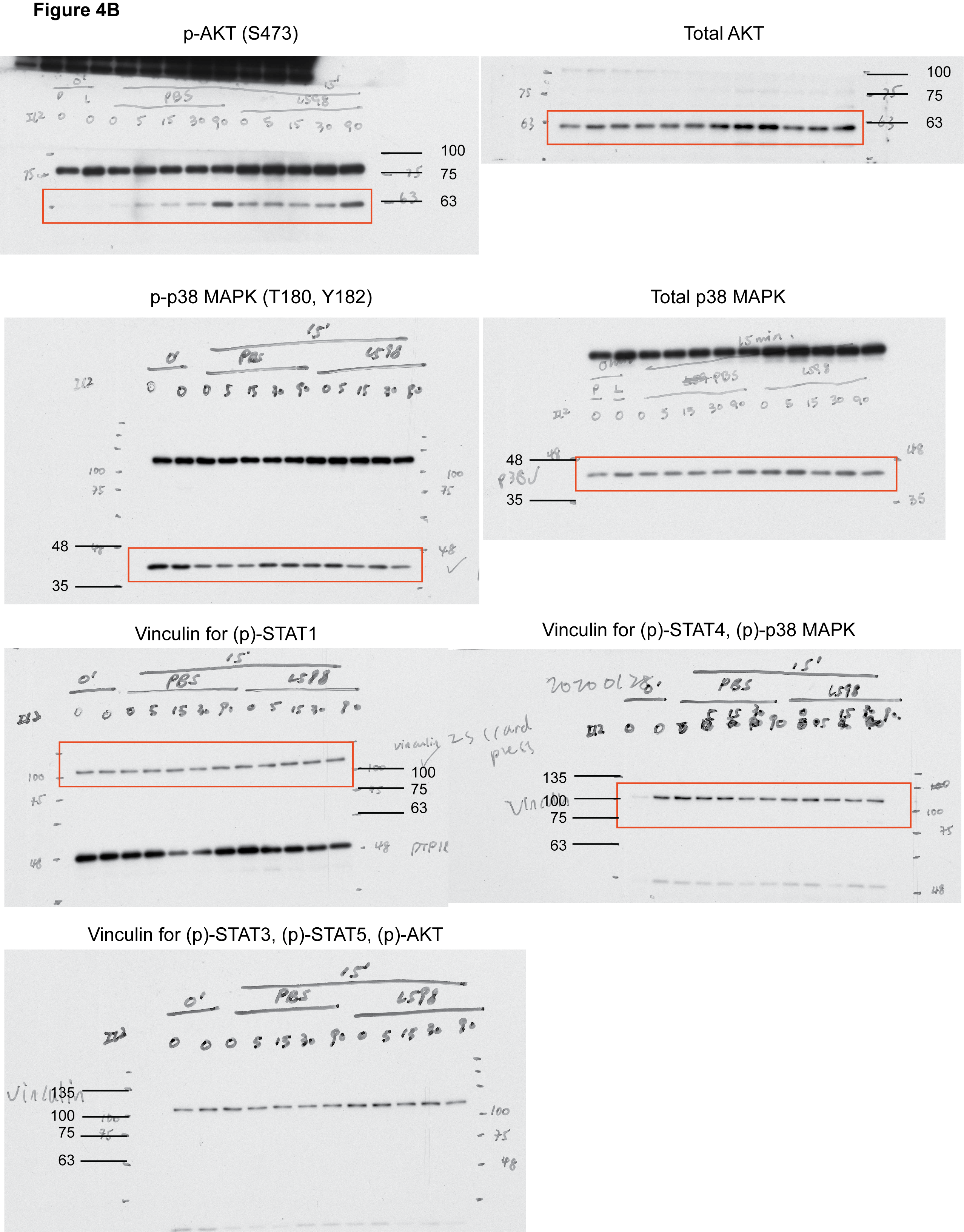

Supplement: Supplementary file 6 — Source data Fig. 4 [file 44319_2026_745_MOESM6_ESM.zip › Figure 4/4B/Raw Data/EXP1/4B_EXP1_Vinculin Loading Control_AKT_MAPK.tif]

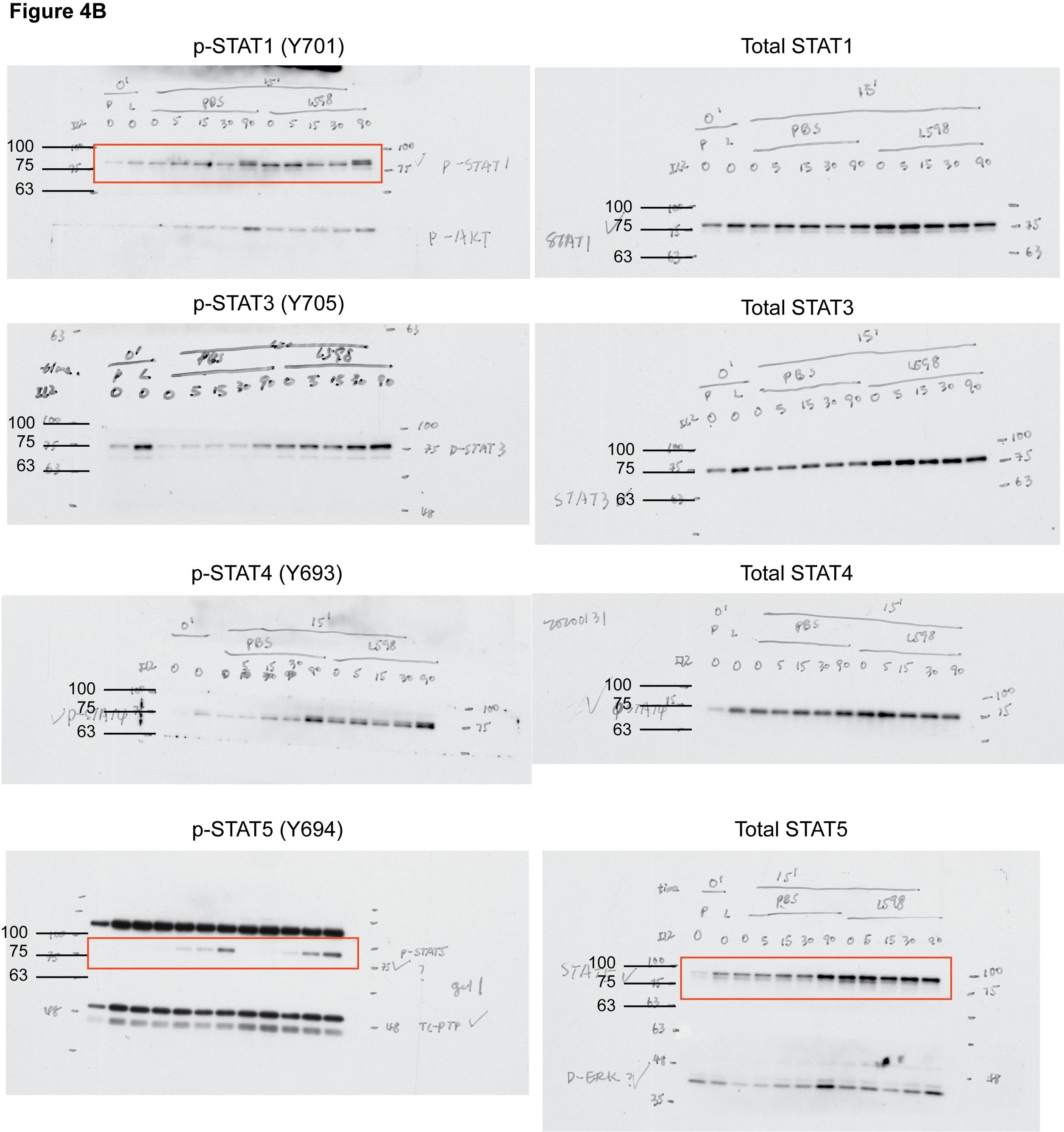

Supplement: Supplementary file 6 — Source data Fig. 4 [file 44319_2026_745_MOESM6_ESM.zip › Figure 4/4B/Raw Data/EXP1/4B_EXP1_STAT1_STAT3_STAT4_STAT5.tif]

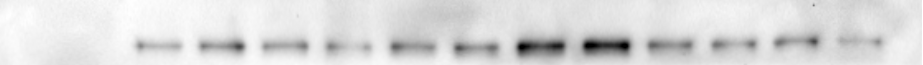

Supplement: Supplementary file 6 — Source data Fig. 4 [file 44319_2026_745_MOESM6_ESM.zip › Figure 4/4B/Raw Data/EXP2/4B_Vinculin STAT5_31.5sec.tif]

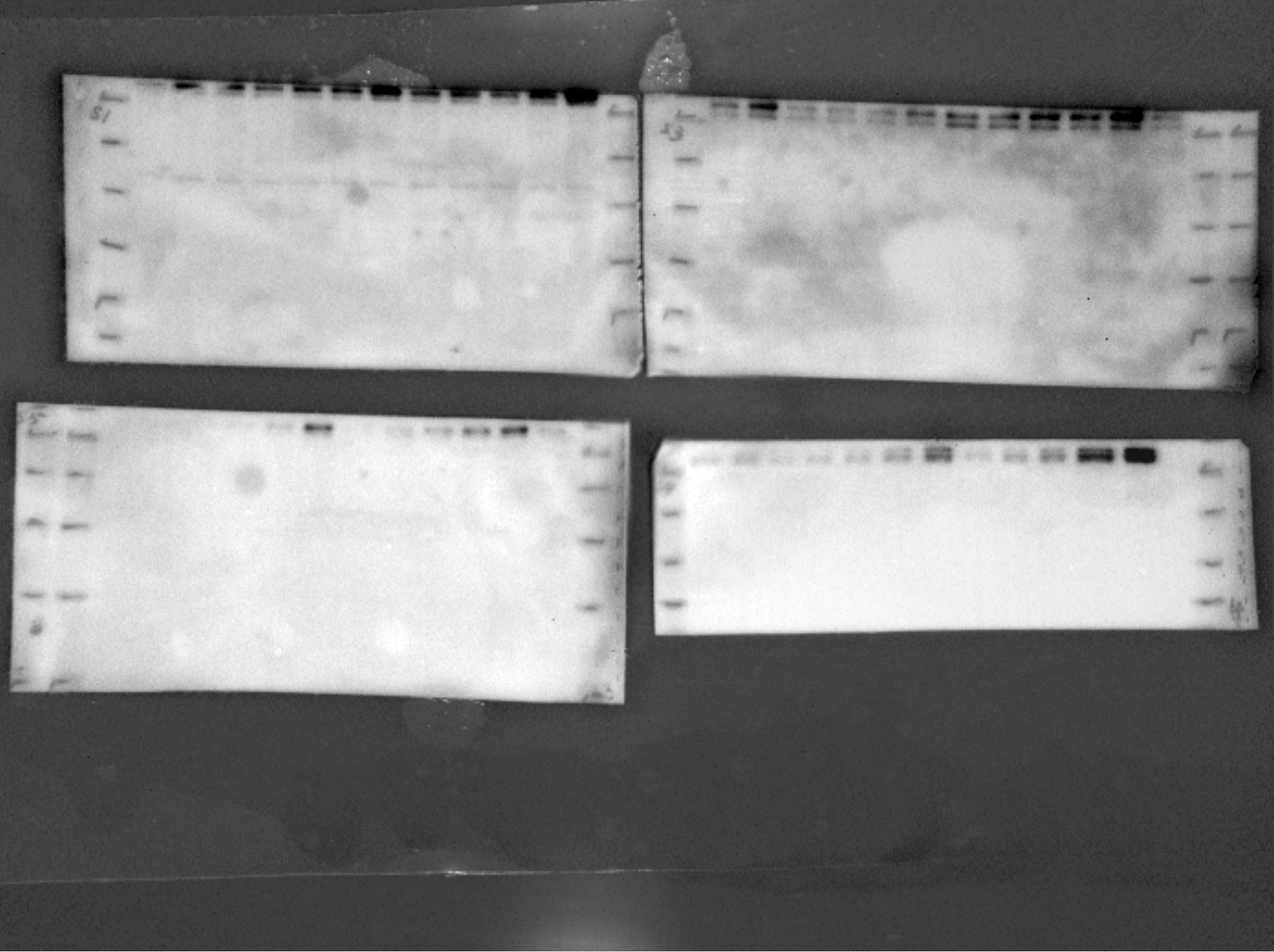

Supplement: Supplementary file 6 — Source data Fig. 4 [file 44319_2026_745_MOESM6_ESM.zip › Figure 4/4B/Raw Data/EXP2/4B_pstat_25.4sec+colori pstats.tif]

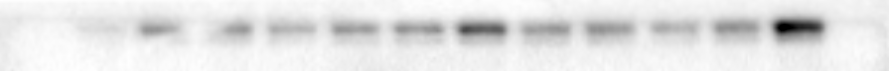

Supplement: Supplementary file 6 — Source data Fig. 4 [file 44319_2026_745_MOESM6_ESM.zip › Figure 4/4B/Raw Data/EXP2/4B_pstat1_13.2sec.tif]

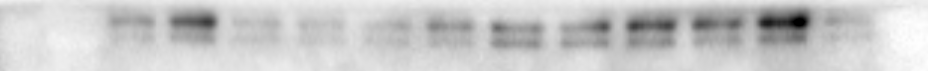

Supplement: Supplementary file 6 — Source data Fig. 4 [file 44319_2026_745_MOESM6_ESM.zip › Figure 4/4B/Raw Data/EXP2/4B_pstat3_25.4sec.tif]

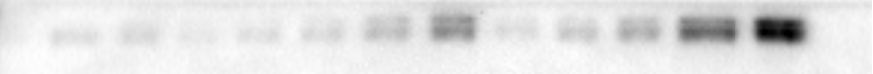

Supplement: Supplementary file 6 — Source data Fig. 4 [file 44319_2026_745_MOESM6_ESM.zip › Figure 4/4B/Raw Data/EXP2/4B_pstat4_31.5sec.tif]

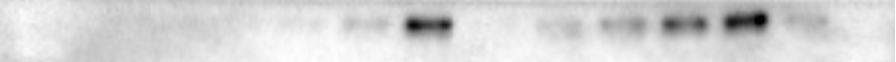

Supplement: Supplementary file 6 — Source data Fig. 4 [file 44319_2026_745_MOESM6_ESM.zip › Figure 4/4B/Raw Data/EXP2/4B_pstat5_31.5sec.tif]

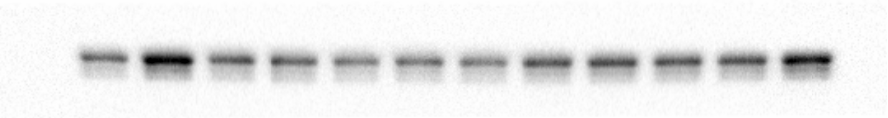

Supplement: Supplementary file 6 — Source data Fig. 4 [file 44319_2026_745_MOESM6_ESM.zip › Figure 4/4B/Raw Data/EXP2/4B_stat1_27.2sec.tif]

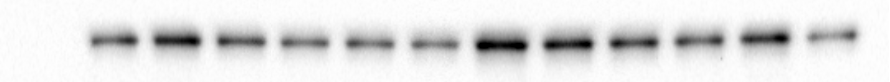

Supplement: Supplementary file 6 — Source data Fig. 4 [file 44319_2026_745_MOESM6_ESM.zip › Figure 4/4B/Raw Data/EXP2/4B_stat3_29.2sec.tif]

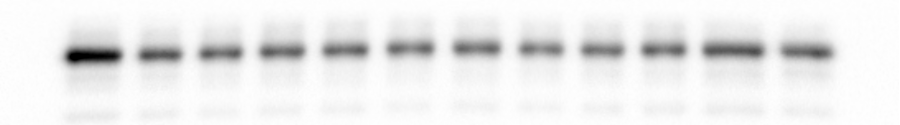

Supplement: Supplementary file 6 — Source data Fig. 4 [file 44319_2026_745_MOESM6_ESM.zip › Figure 4/4B/Raw Data/EXP2/4B_stat4_5.0sec.tif]

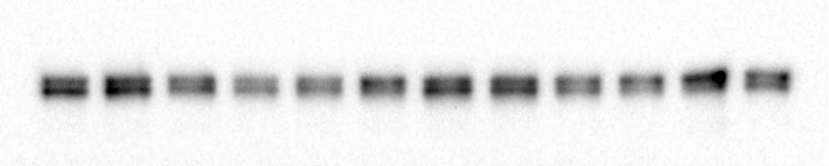

Supplement: Supplementary file 6 — Source data Fig. 4 [file 44319_2026_745_MOESM6_ESM.zip › Figure 4/4B/Raw Data/EXP2/4B_stat5_29.2sec.tif]

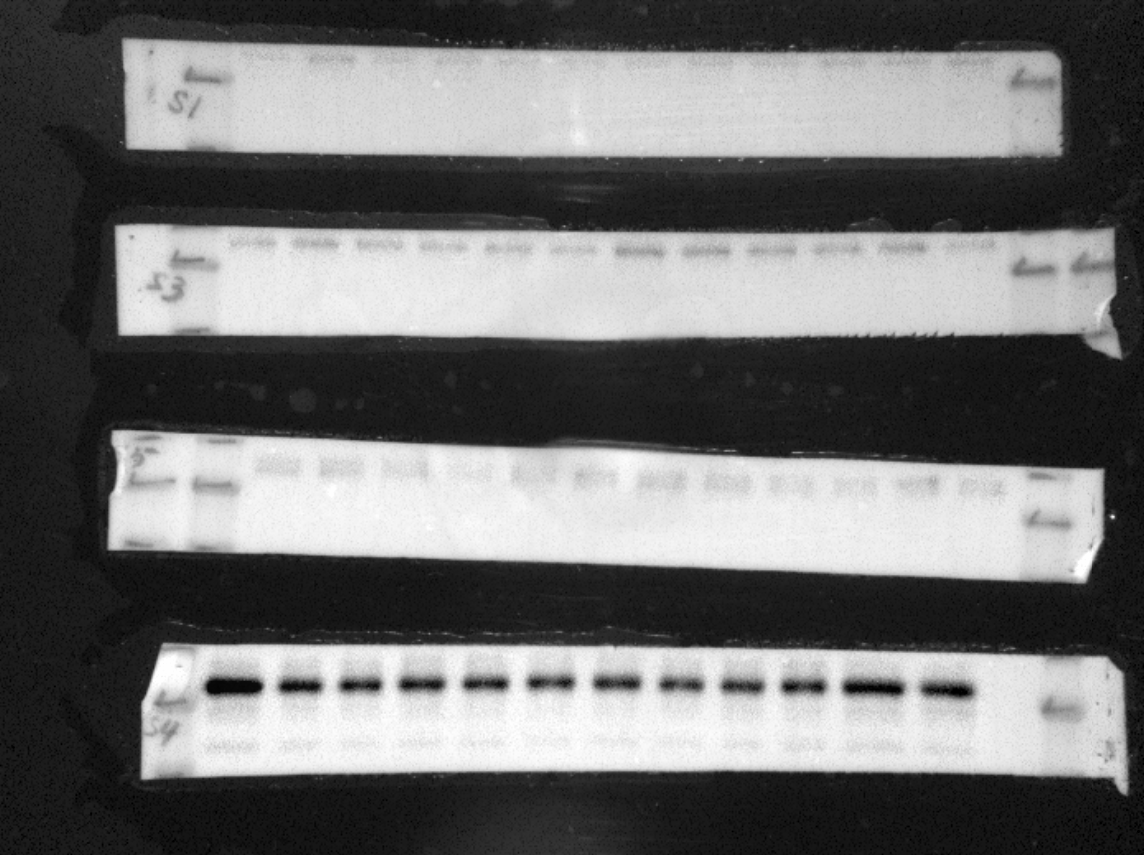

Supplement: Supplementary file 6 — Source data Fig. 4 [file 44319_2026_745_MOESM6_ESM.zip › Figure 4/4B/Raw Data/EXP2/4B_stats_1.0sec+day 2 colori stats.tif]

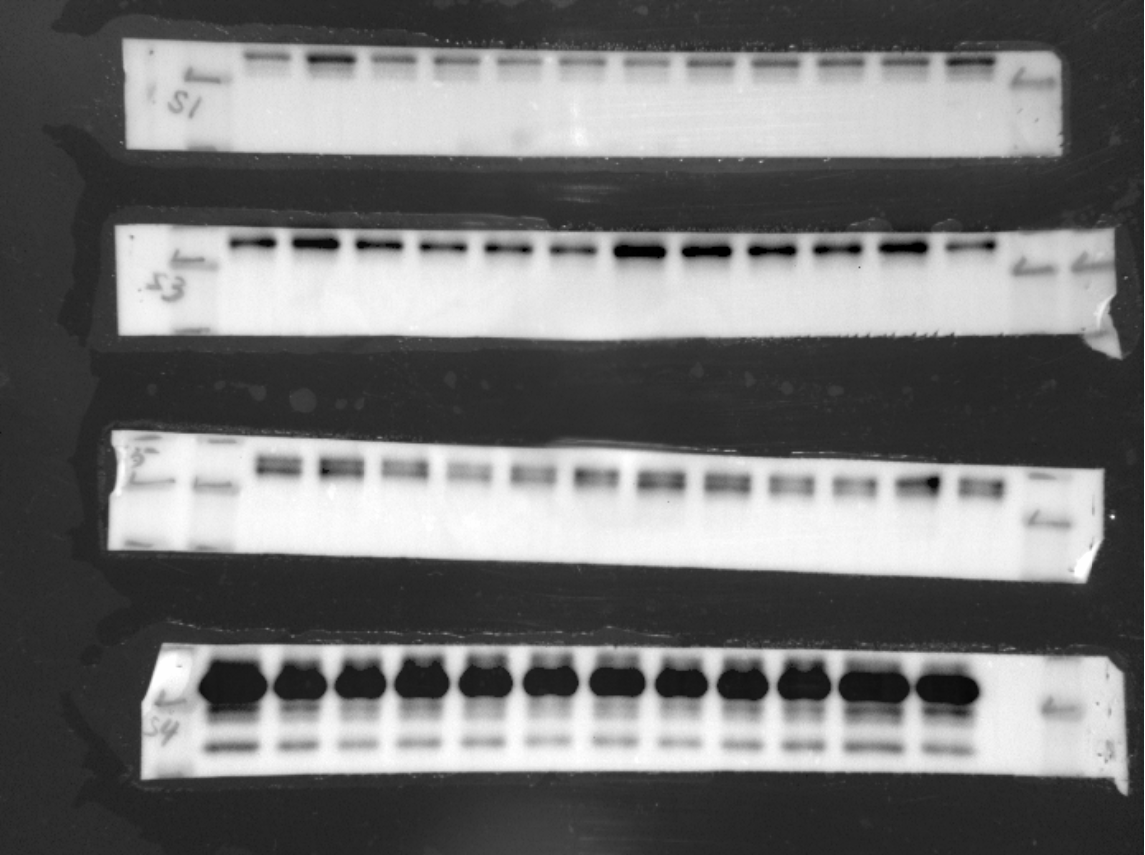

Supplement: Supplementary file 6 — Source data Fig. 4 [file 44319_2026_745_MOESM6_ESM.zip › Figure 4/4B/Raw Data/EXP2/4B_stats_60.0sec+day 2 colori stats.tif]

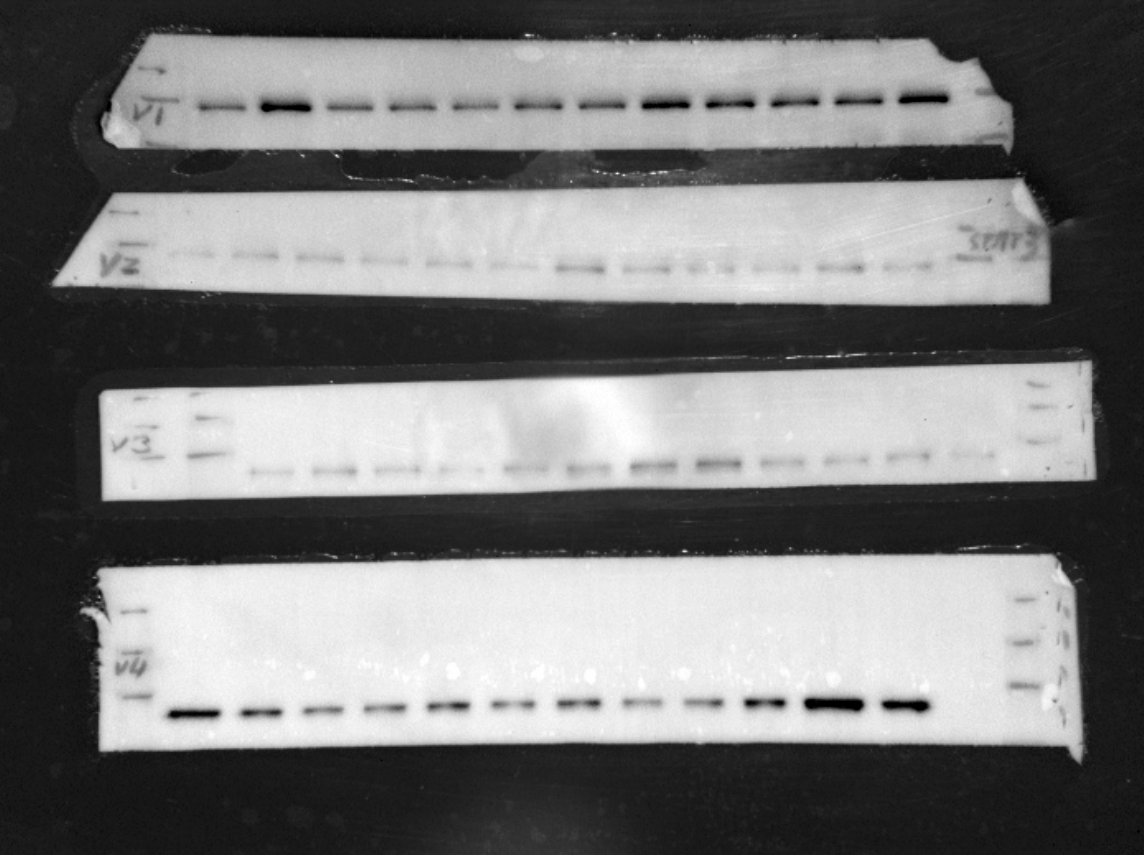

Supplement: Supplementary file 6 — Source data Fig. 4 [file 44319_2026_745_MOESM6_ESM.zip › Figure 4/4B/Raw Data/EXP2/4B_v2_7.1sec+v2 colori.tif]

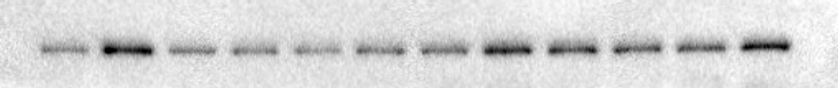

Supplement: Supplementary file 6 — Source data Fig. 4 [file 44319_2026_745_MOESM6_ESM.zip › Figure 4/4B/Raw Data/EXP2/4B_Vinculin STAT1_1.0sec.tif]

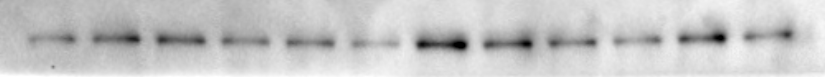

Supplement: Supplementary file 6 — Source data Fig. 4 [file 44319_2026_745_MOESM6_ESM.zip › Figure 4/4B/Raw Data/EXP2/4B_Vinculin STAT3_19.3sec.tif]

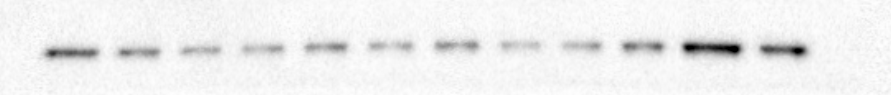

Supplement: Supplementary file 6 — Source data Fig. 4 [file 44319_2026_745_MOESM6_ESM.zip › Figure 4/4B/Raw Data/EXP2/4B_Vinculin STAT4_1.0sec.tif]

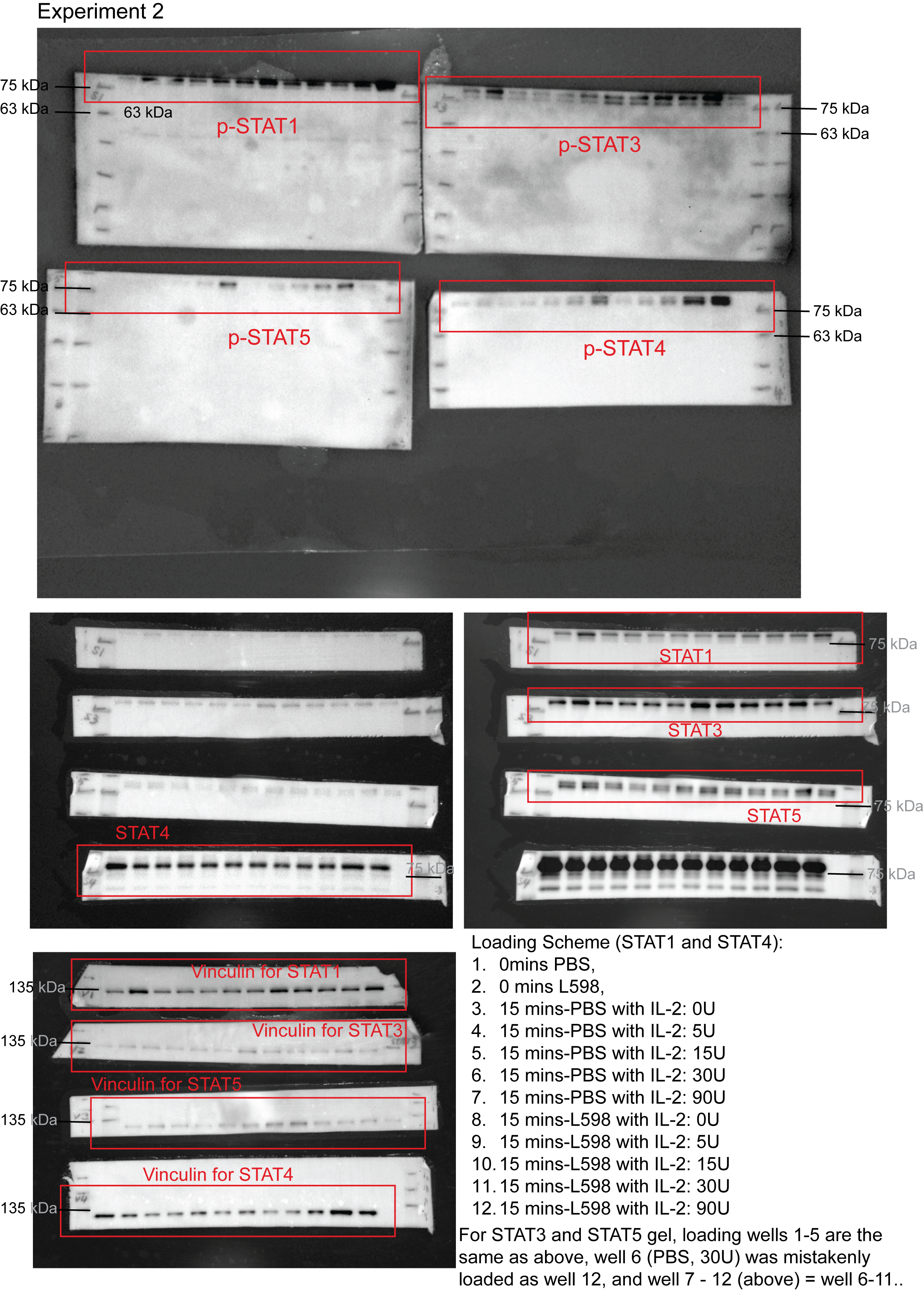

Supplement: Supplementary file 6 — Source data Fig. 4 [file 44319_2026_745_MOESM6_ESM.zip › Figure 4/4B/Raw Data/EXP2/Exp2_IL2 Titration .tif]

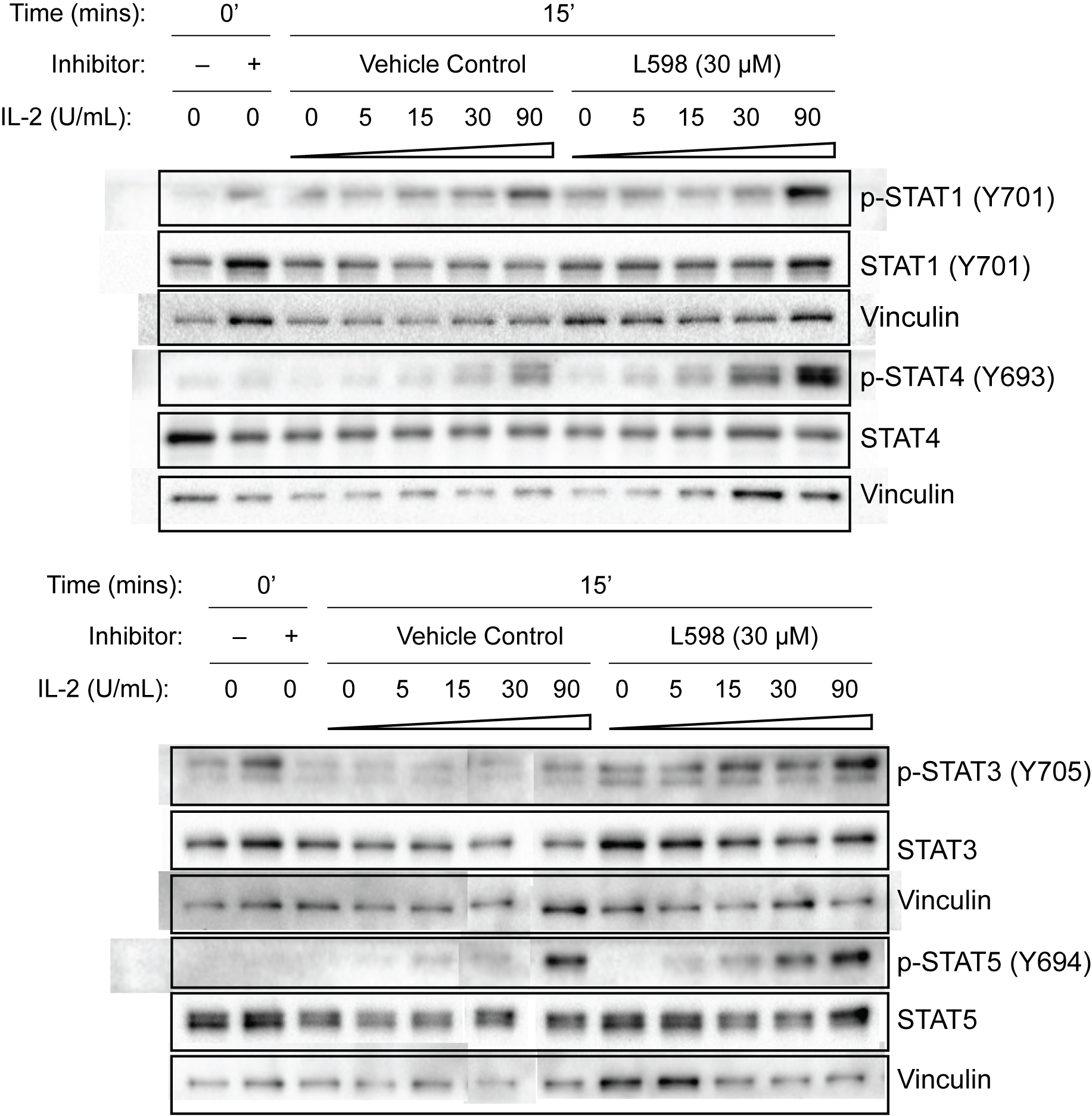

Supplement: Supplementary file 6 — Source data Fig. 4 [file 44319_2026_745_MOESM6_ESM.zip › Figure 4/4B/Raw Data/EXP2/Exp2_IL2 Titration Aligned.tif]

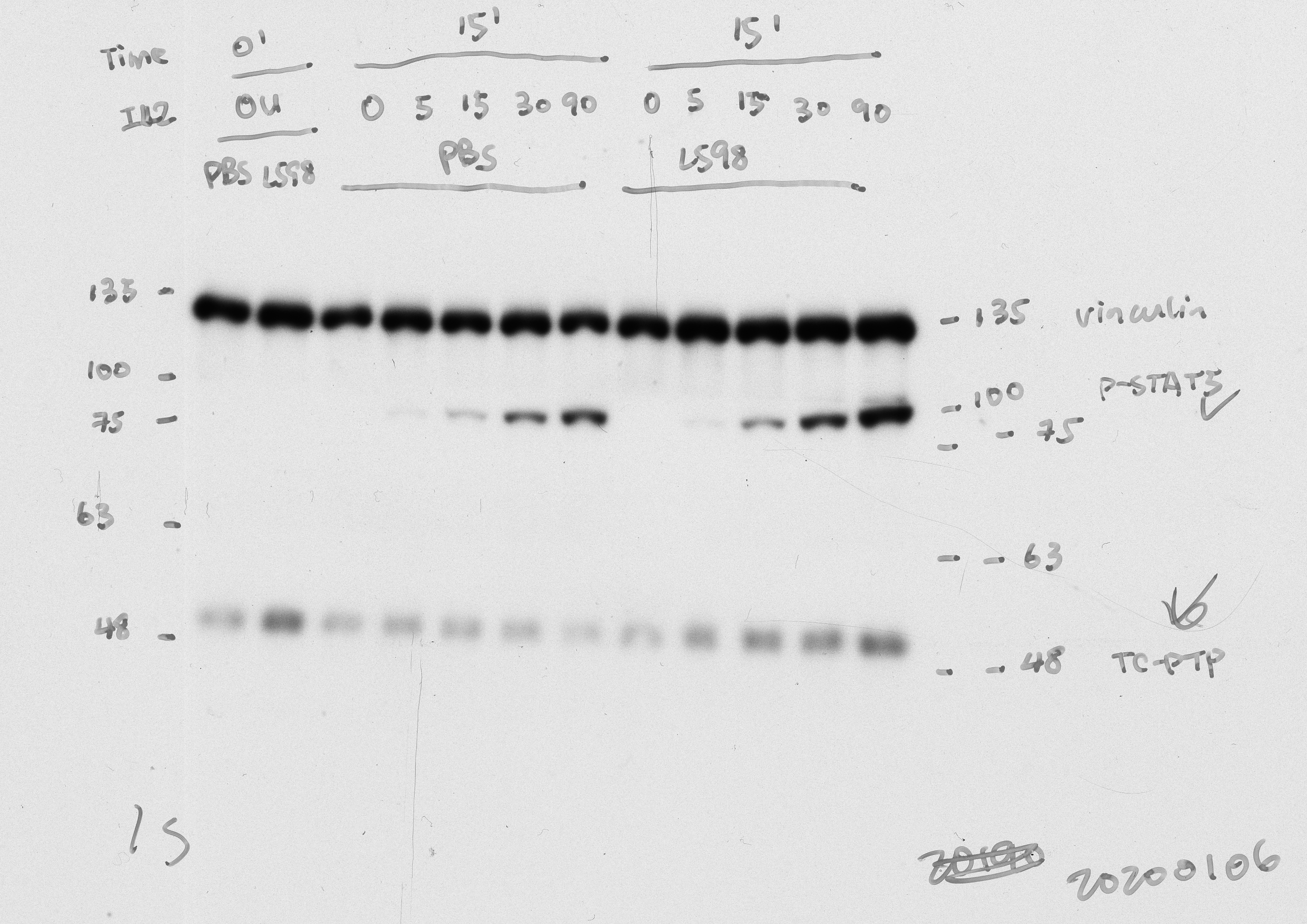

Supplement: Supplementary file 6 — Source data Fig. 4 [file 44319_2026_745_MOESM6_ESM.zip › Figure 4/4B/Raw Data/Other Replicates/p-STAT5003.tif]

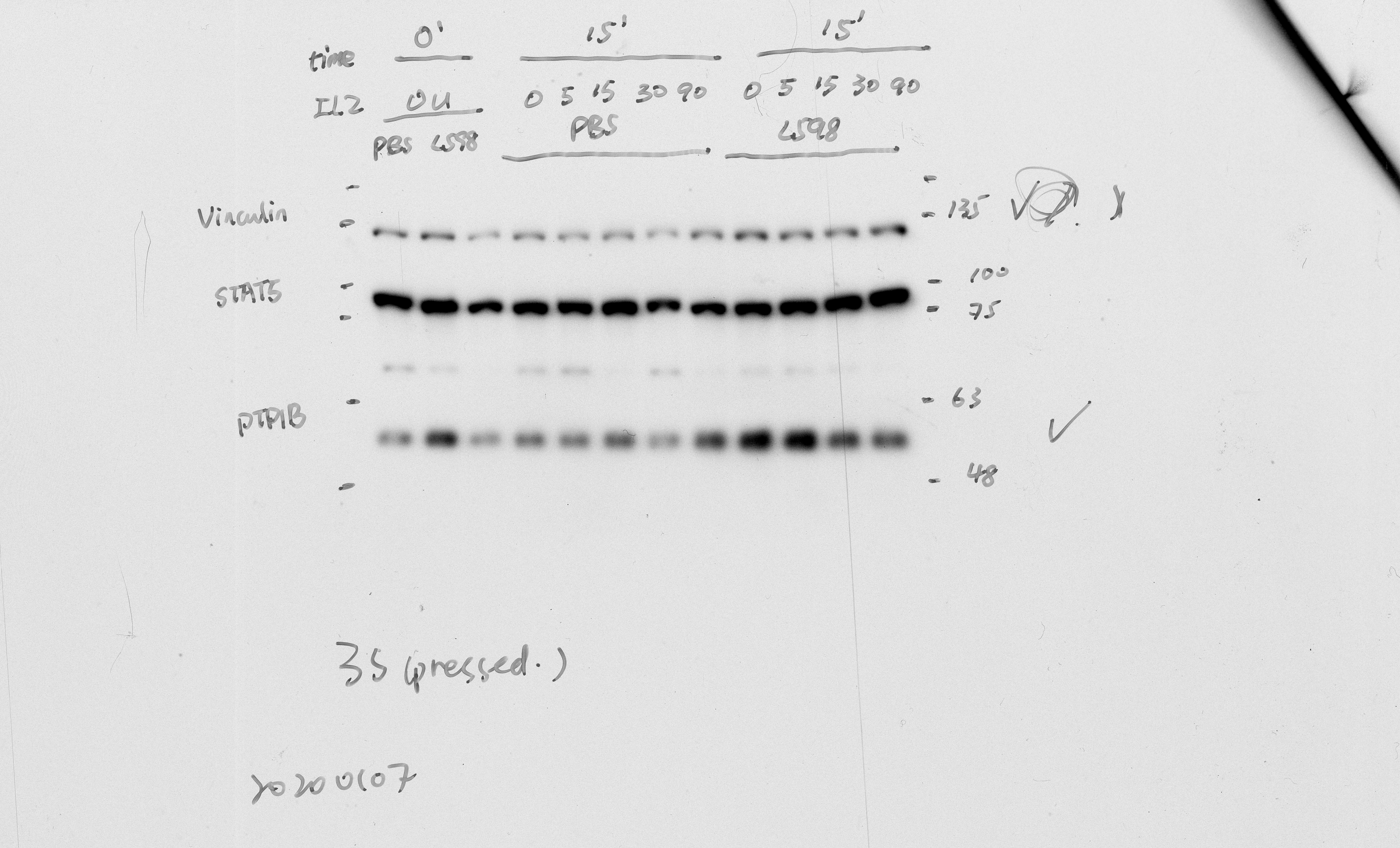

Supplement: Supplementary file 6 — Source data Fig. 4 [file 44319_2026_745_MOESM6_ESM.zip › Figure 4/4B/Raw Data/Other Replicates/PTP1B006.tif]

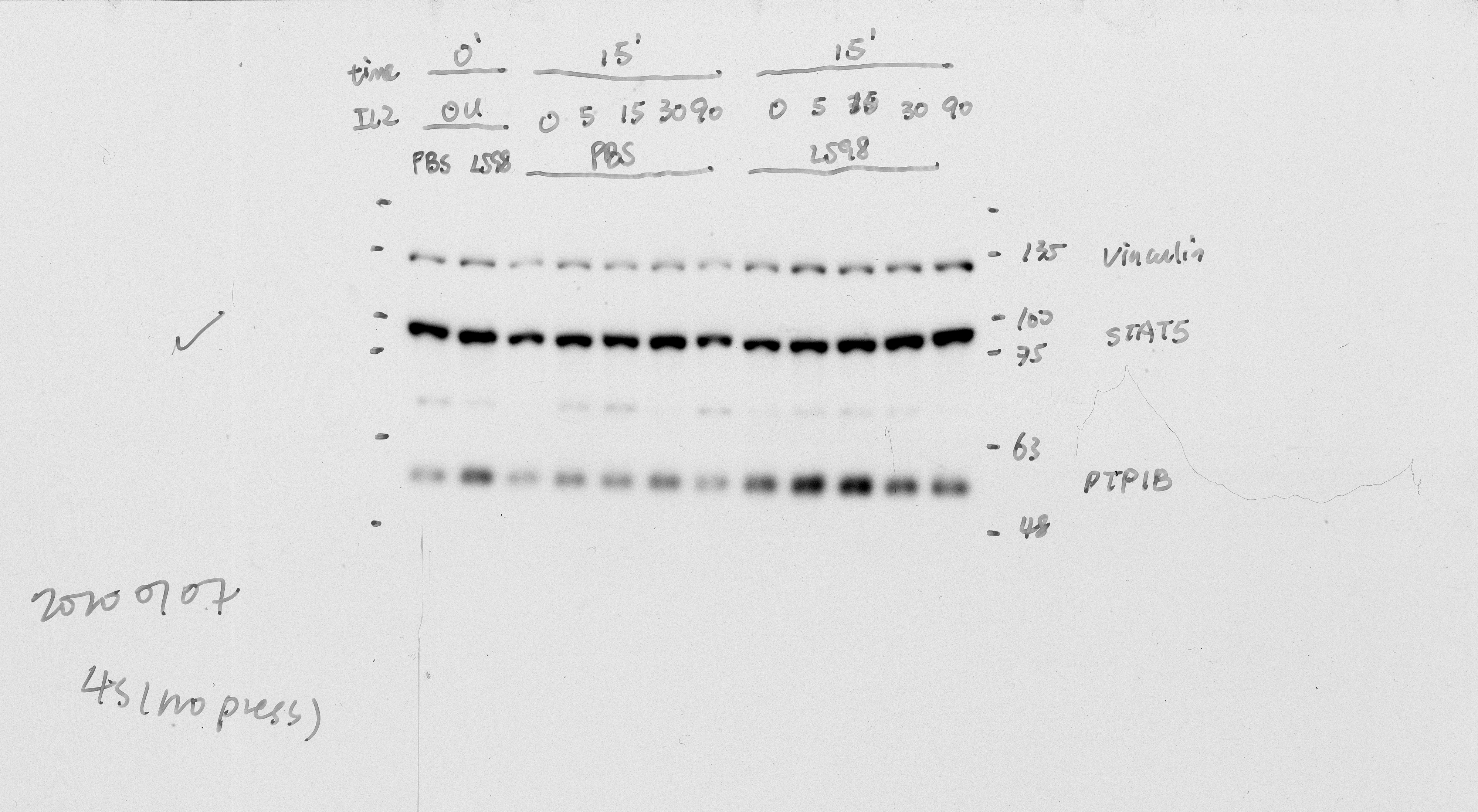

Supplement: Supplementary file 6 — Source data Fig. 4 [file 44319_2026_745_MOESM6_ESM.zip › Figure 4/4B/Raw Data/Other Replicates/STAT5005.tif]

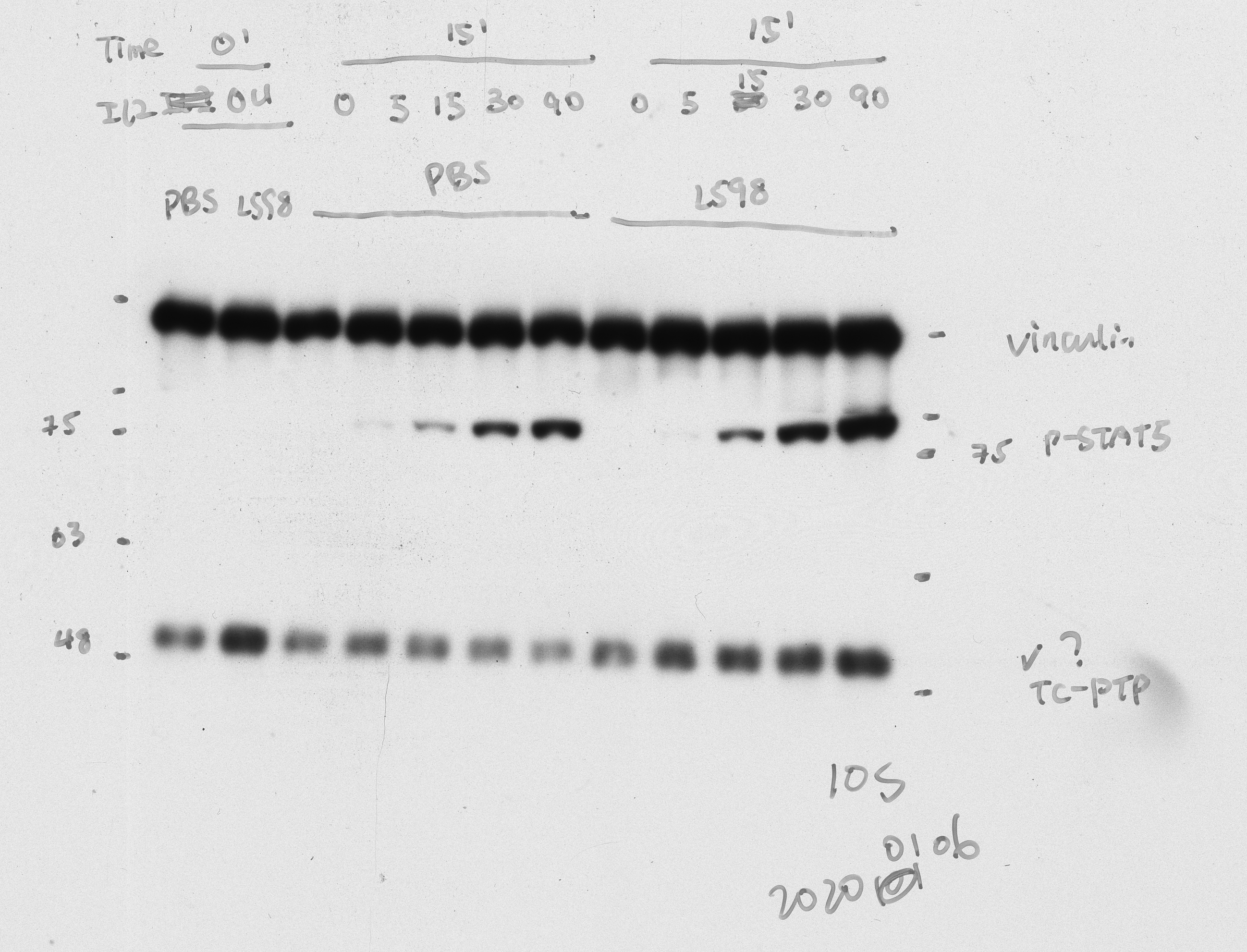

Supplement: Supplementary file 6 — Source data Fig. 4 [file 44319_2026_745_MOESM6_ESM.zip › Figure 4/4B/Raw Data/Other Replicates/TC-PTP001.tif]

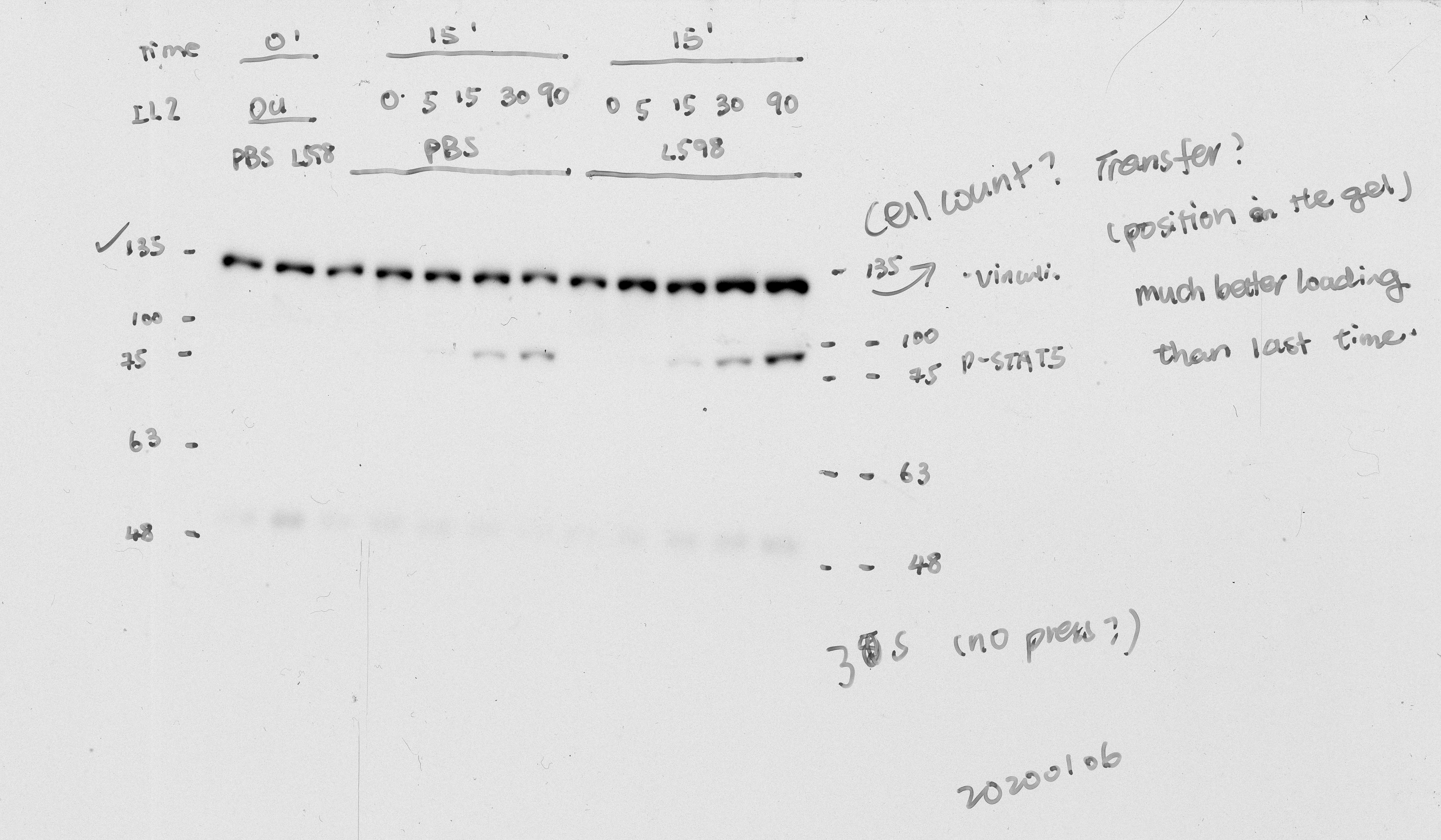

Supplement: Supplementary file 6 — Source data Fig. 4 [file 44319_2026_745_MOESM6_ESM.zip › Figure 4/4B/Raw Data/Other Replicates/vinculin004.tif]

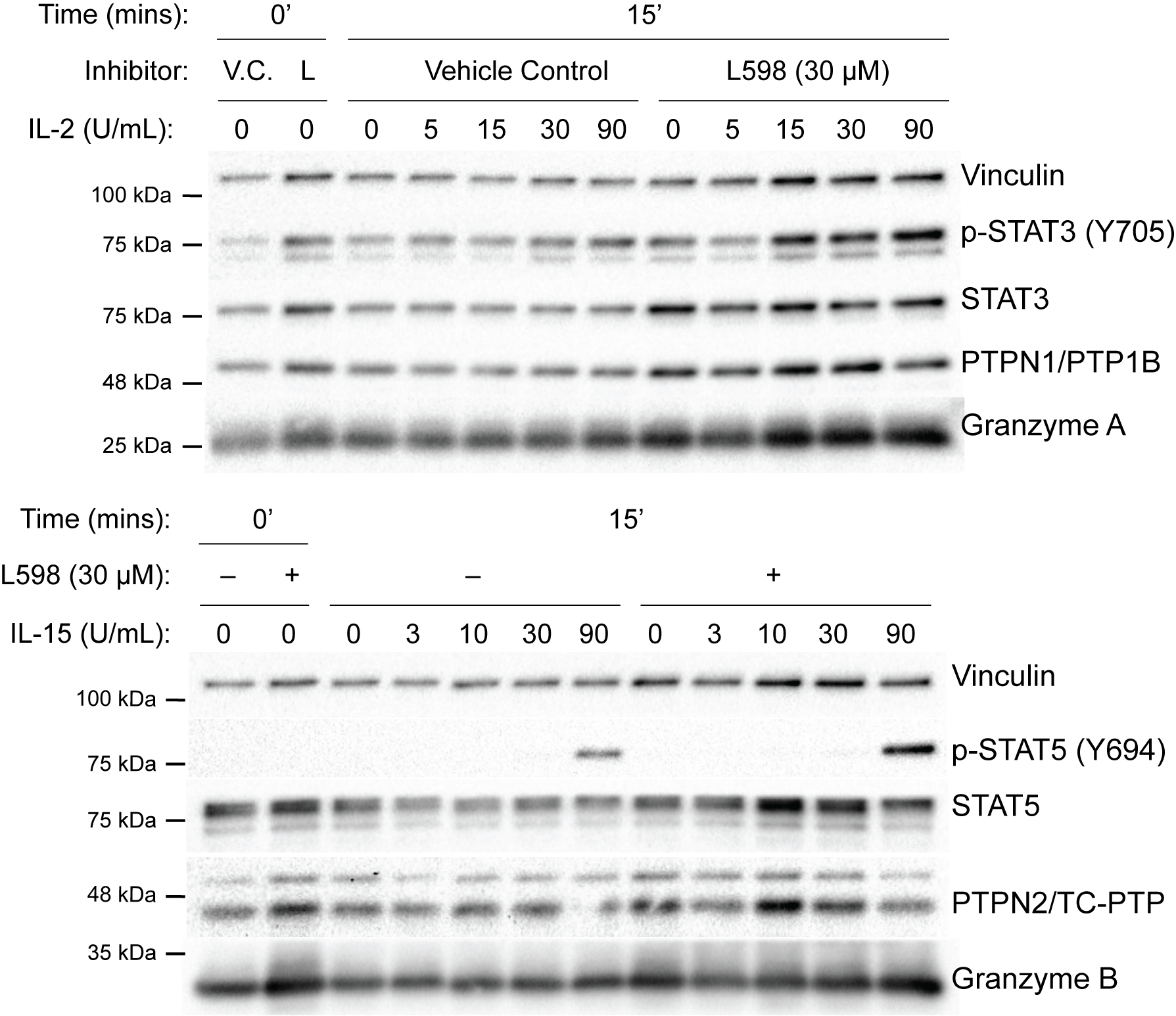

Supplement: Supplementary file 6 — Source data Fig. 4 [file 44319_2026_745_MOESM6_ESM.zip › Figure 4/4B/Raw Data/Other Replicates/4B_EXP2_IL-2_pSTAT3_pSTAT5.tif]

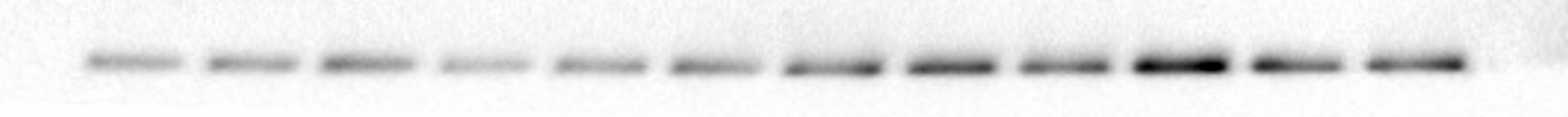

Supplement: Supplementary file 6 — Source data Fig. 4 [file 44319_2026_745_MOESM6_ESM.zip › Figure 4/4B/Raw Data/Other Replicates/4B_EXP2_204.1sec total stat5 15 mins il2 titration.jpg]

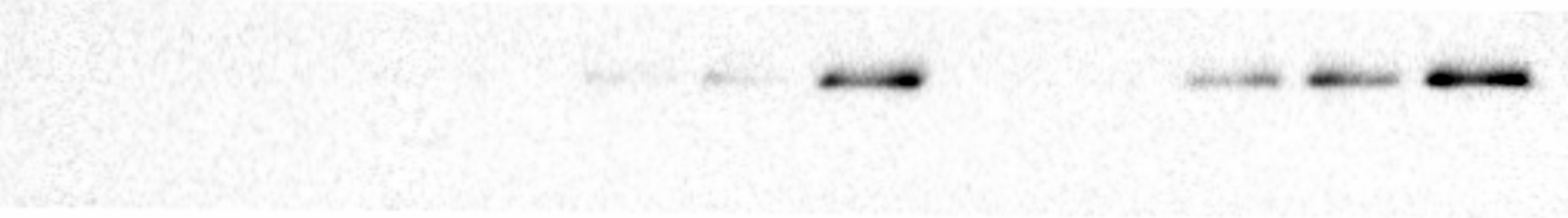

Supplement: Supplementary file 6 — Source data Fig. 4 [file 44319_2026_745_MOESM6_ESM.zip › Figure 4/4B/Raw Data/Other Replicates/4B_EXP2_899.9sec pSTAT5 IL2 stimulation.jpg]

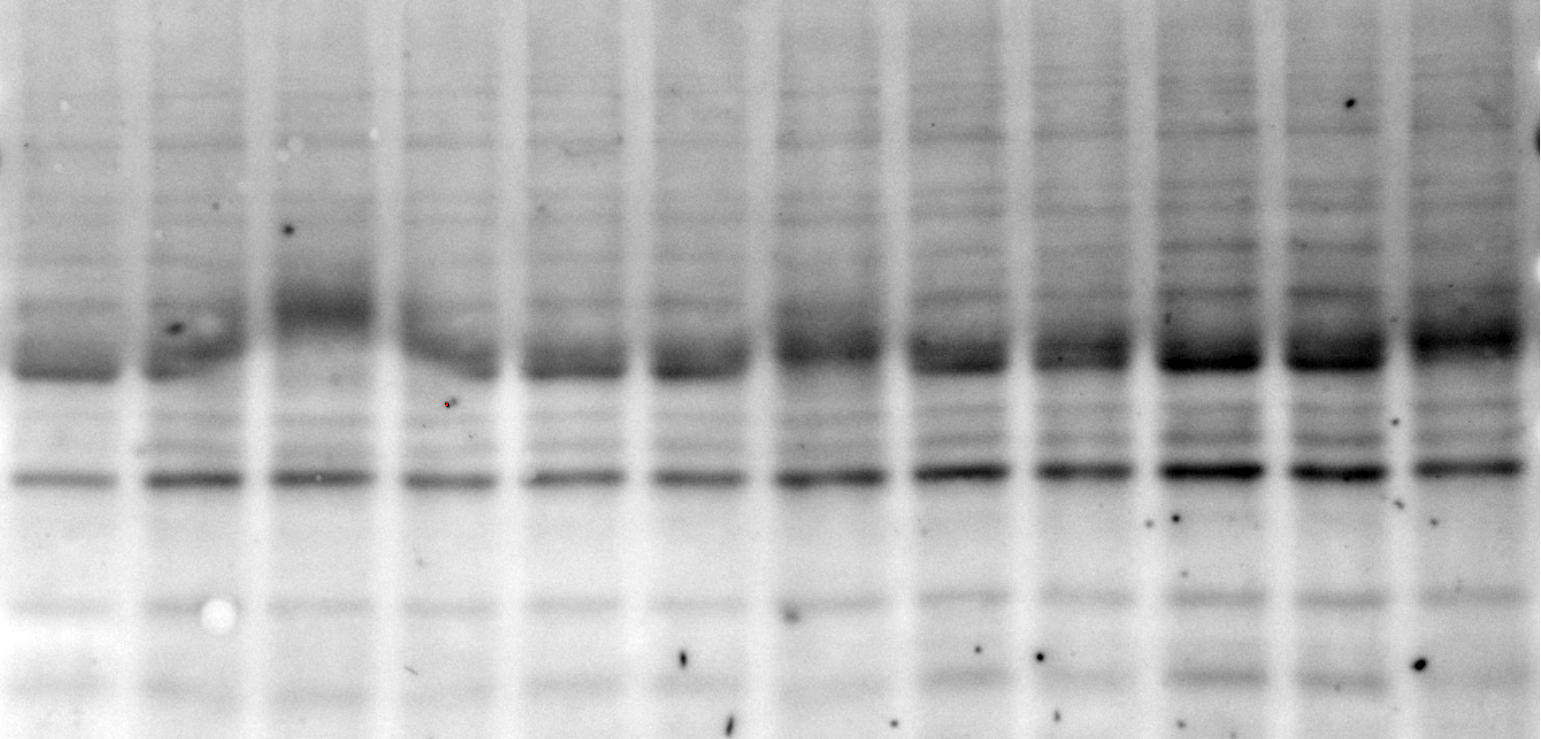

Supplement: Supplementary file 6 — Source data Fig. 4 [file 44319_2026_745_MOESM6_ESM.zip › Figure 4/4B/Raw Data/Other Replicates/4B_EXP2_G2 H total iamge lab AKT and STAT5.tif]

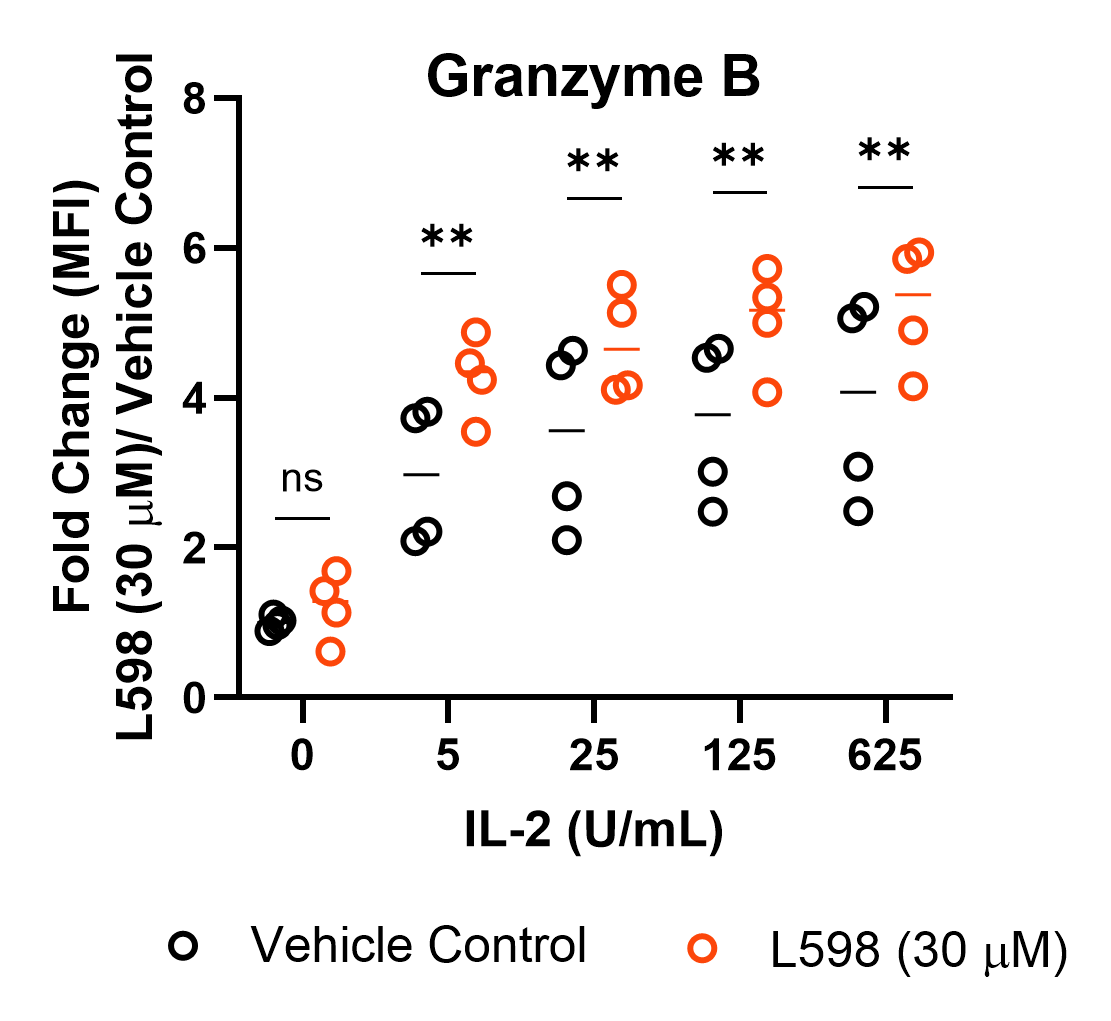

Supplement: Supplementary file 6 — Source data Fig. 4 [file 44319_2026_745_MOESM6_ESM.zip › Figure 4/4E/4E_Scatter of Fold Change Day 3 GranzymeB (Live) Combined 2 Exps.tif]

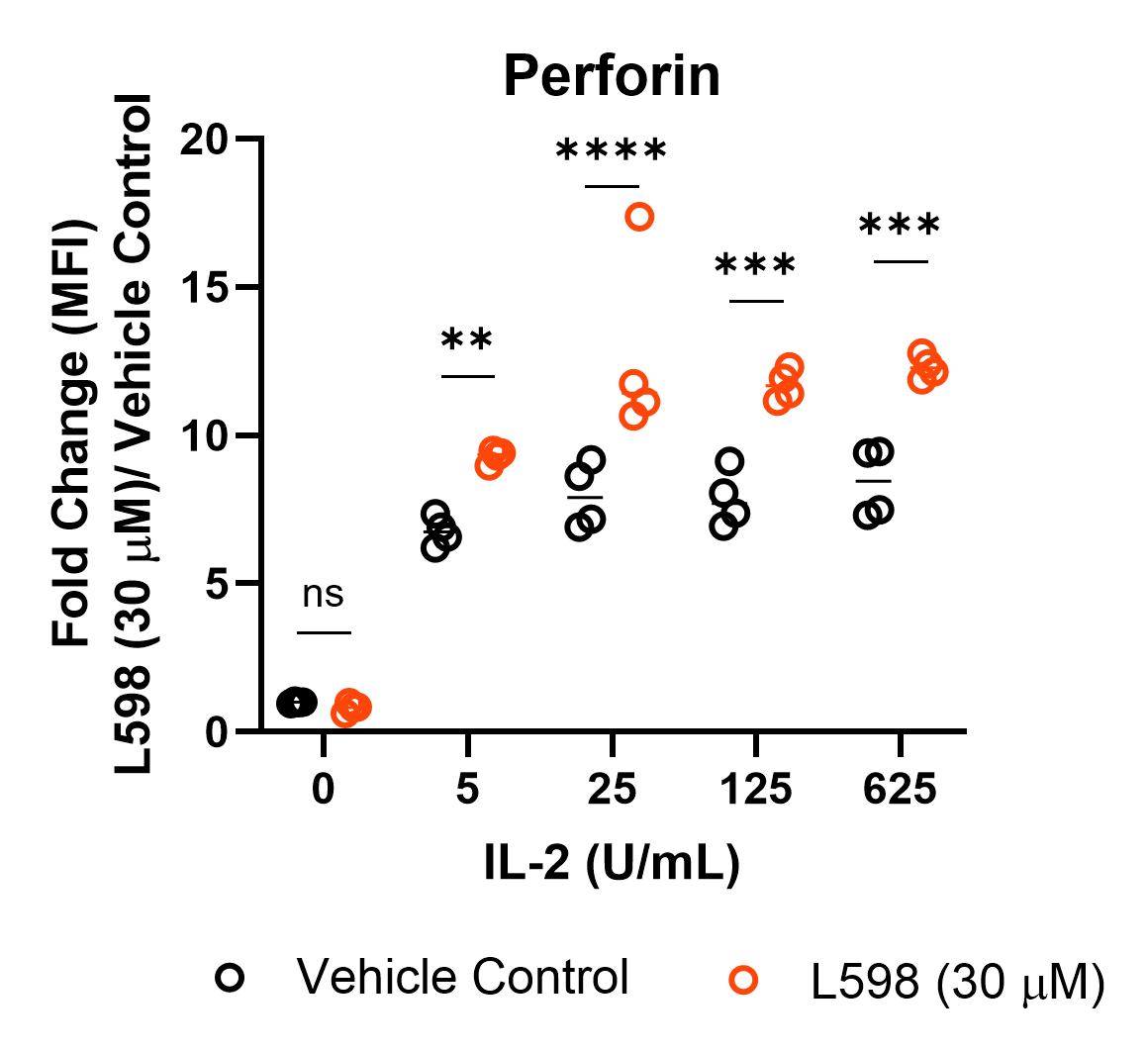

Supplement: Supplementary file 6 — Source data Fig. 4 [file 44319_2026_745_MOESM6_ESM.zip › Figure 4/4F/4F.tif]

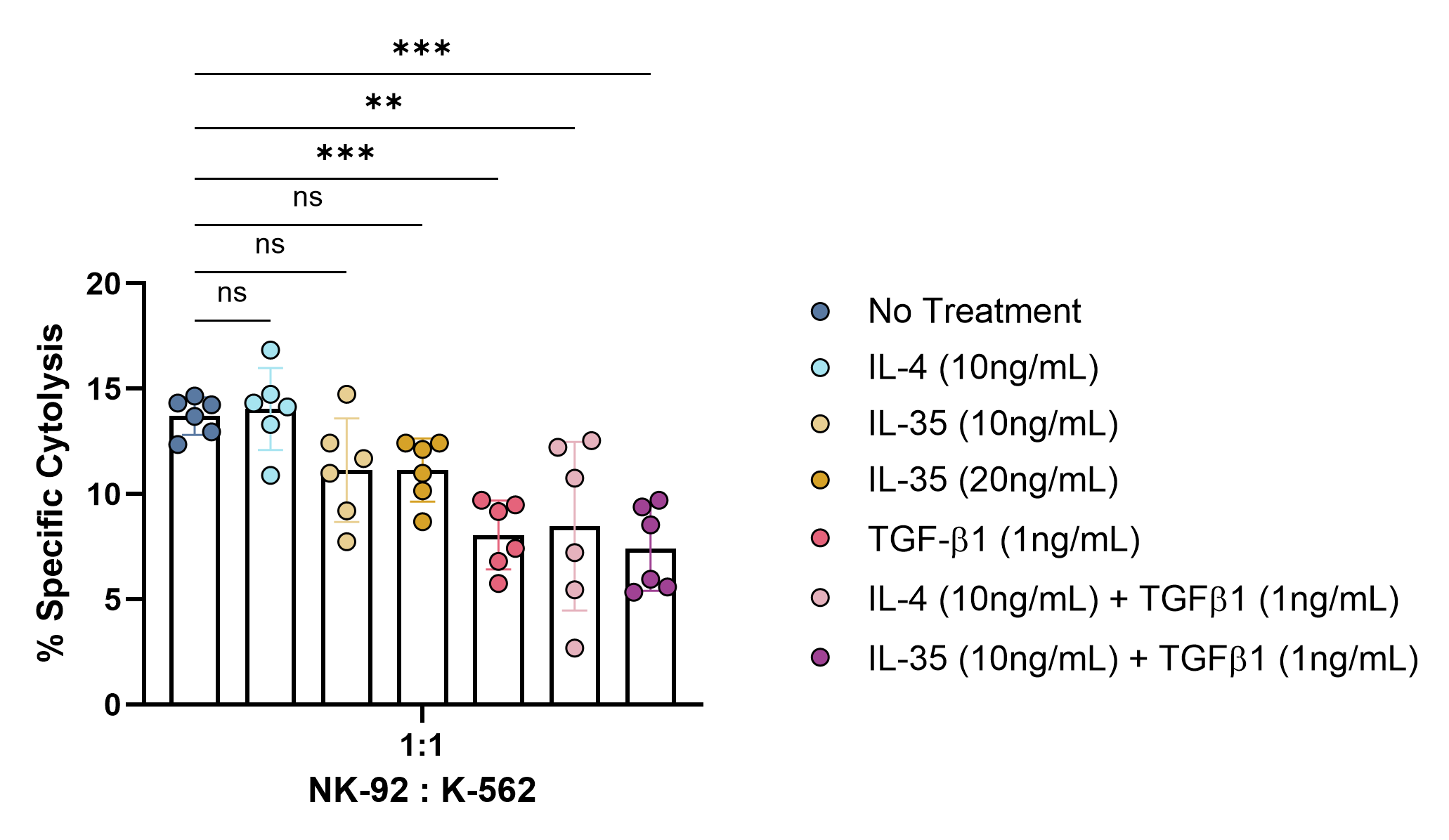

Supplement: Supplementary file 7 — Source data Fig. 5 [file 44319_2026_745_MOESM7_ESM.zip › Figure 5/5A/5A.tif]

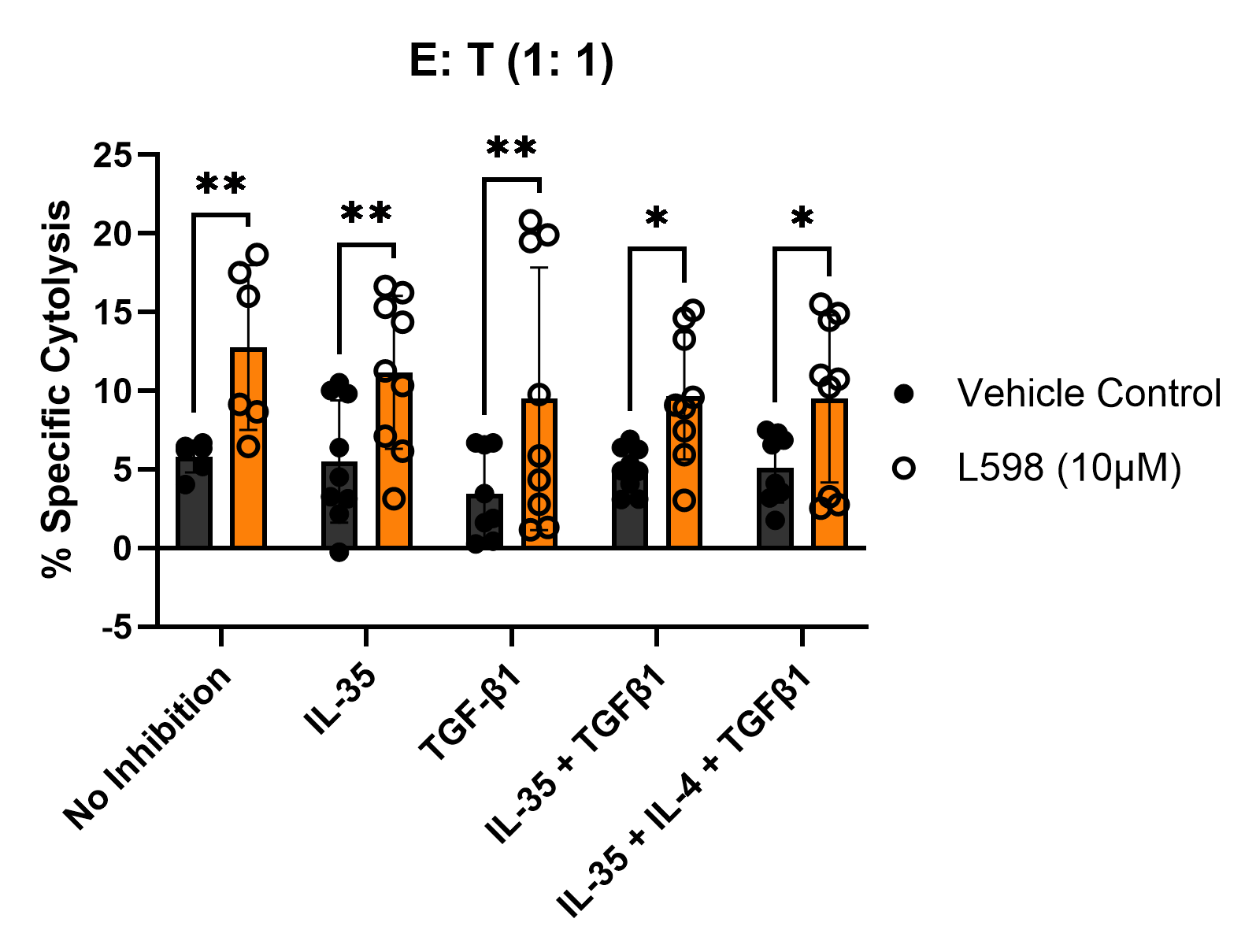

Supplement: Supplementary file 7 — Source data Fig. 5 [file 44319_2026_745_MOESM7_ESM.zip › Figure 5/5B/5B.tif]

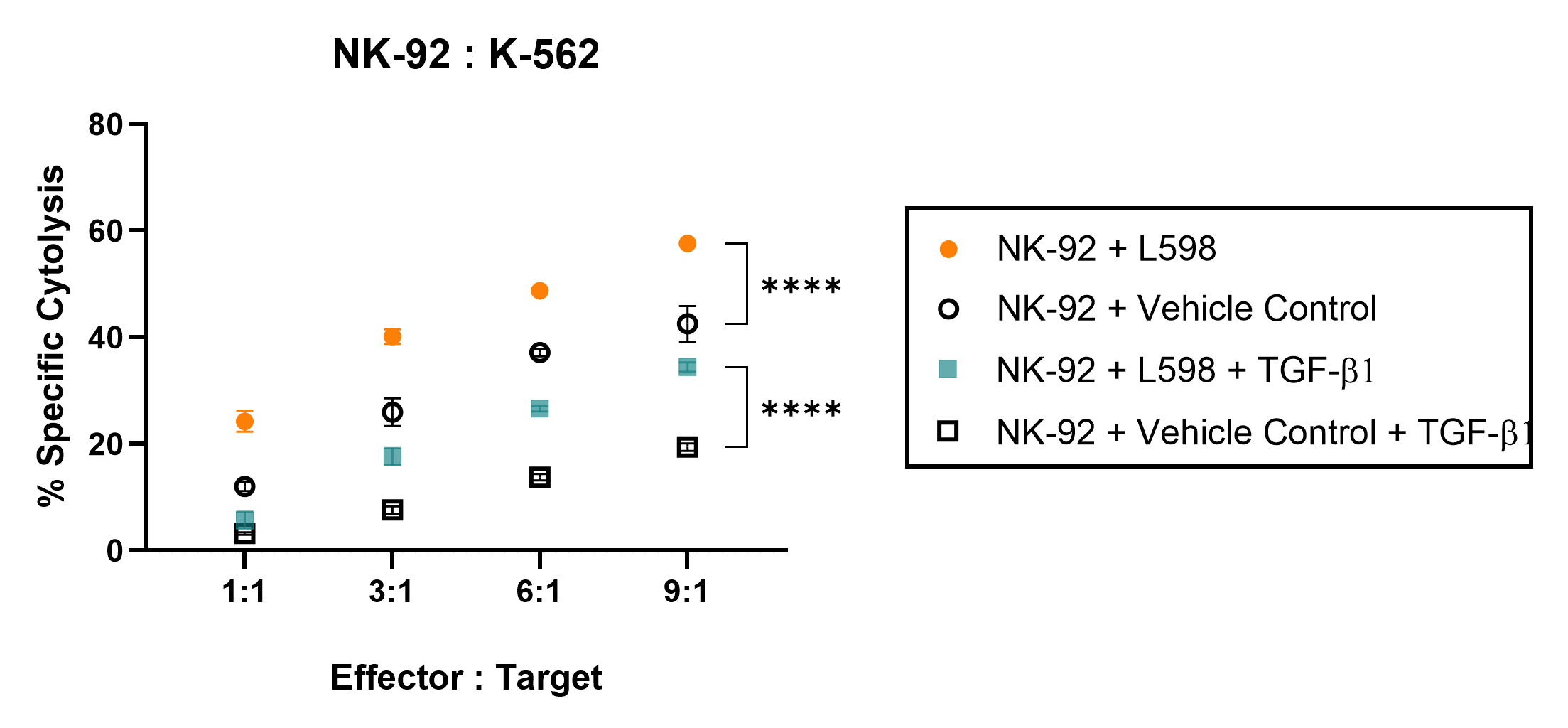

Supplement: Supplementary file 7 — Source data Fig. 5 [file 44319_2026_745_MOESM7_ESM.zip › Figure 5/5C/Repeats/5C_EXP2.tif]

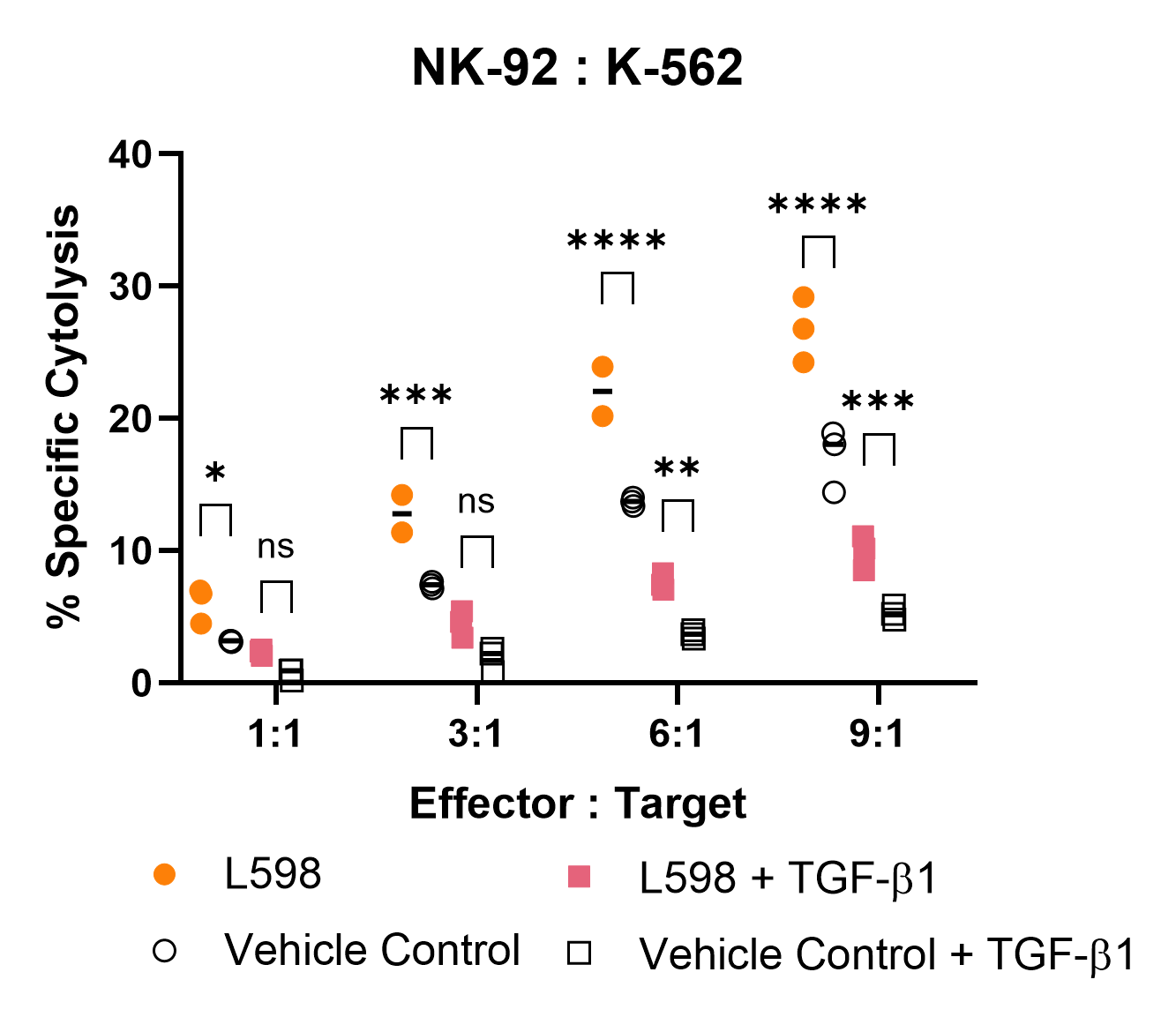

Supplement: Supplementary file 7 — Source data Fig. 5 [file 44319_2026_745_MOESM7_ESM.zip › Figure 5/5C/5C.tif]

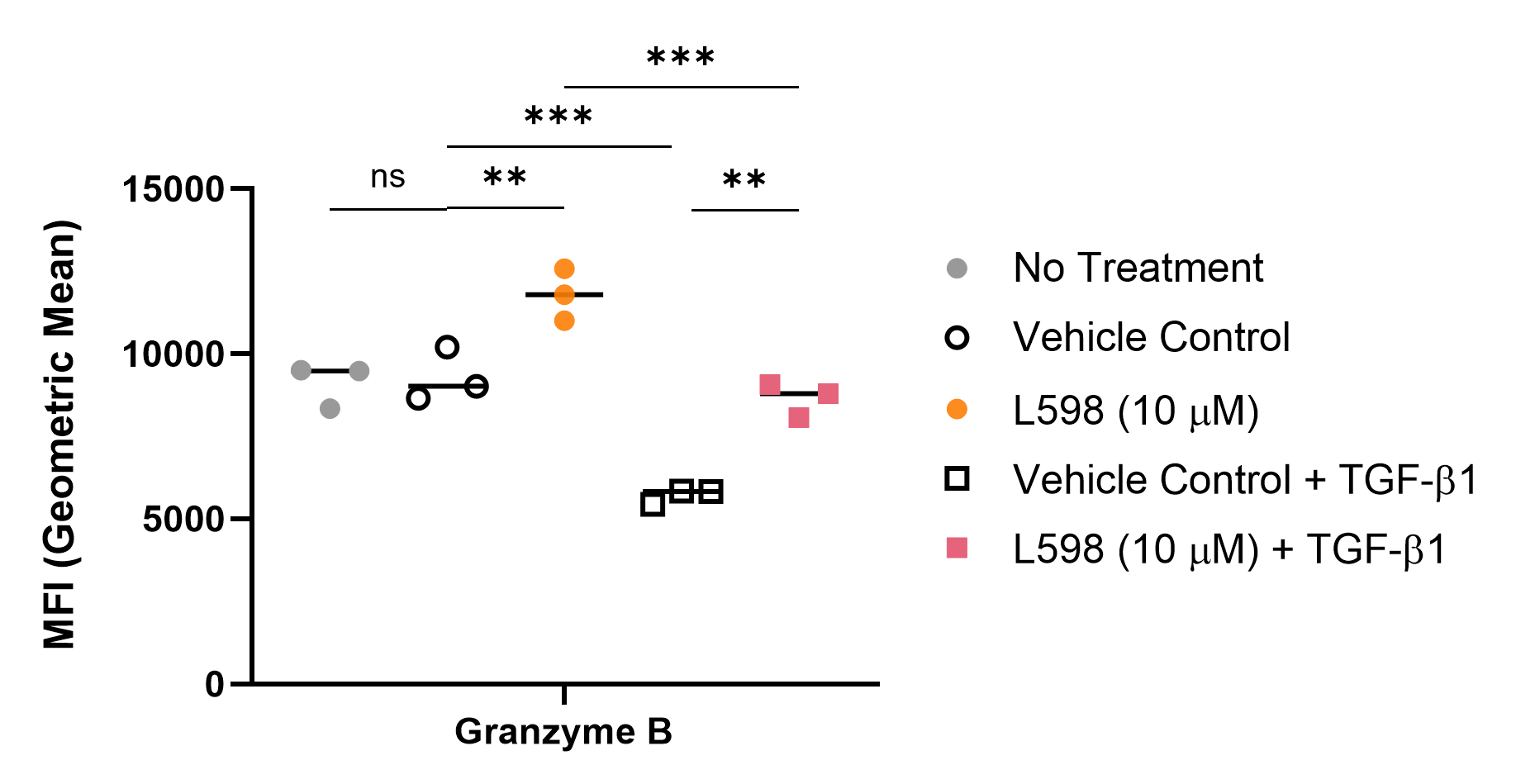

Supplement: Supplementary file 7 — Source data Fig. 5 [file 44319_2026_745_MOESM7_ESM.zip › Figure 5/5D/5D.tif]

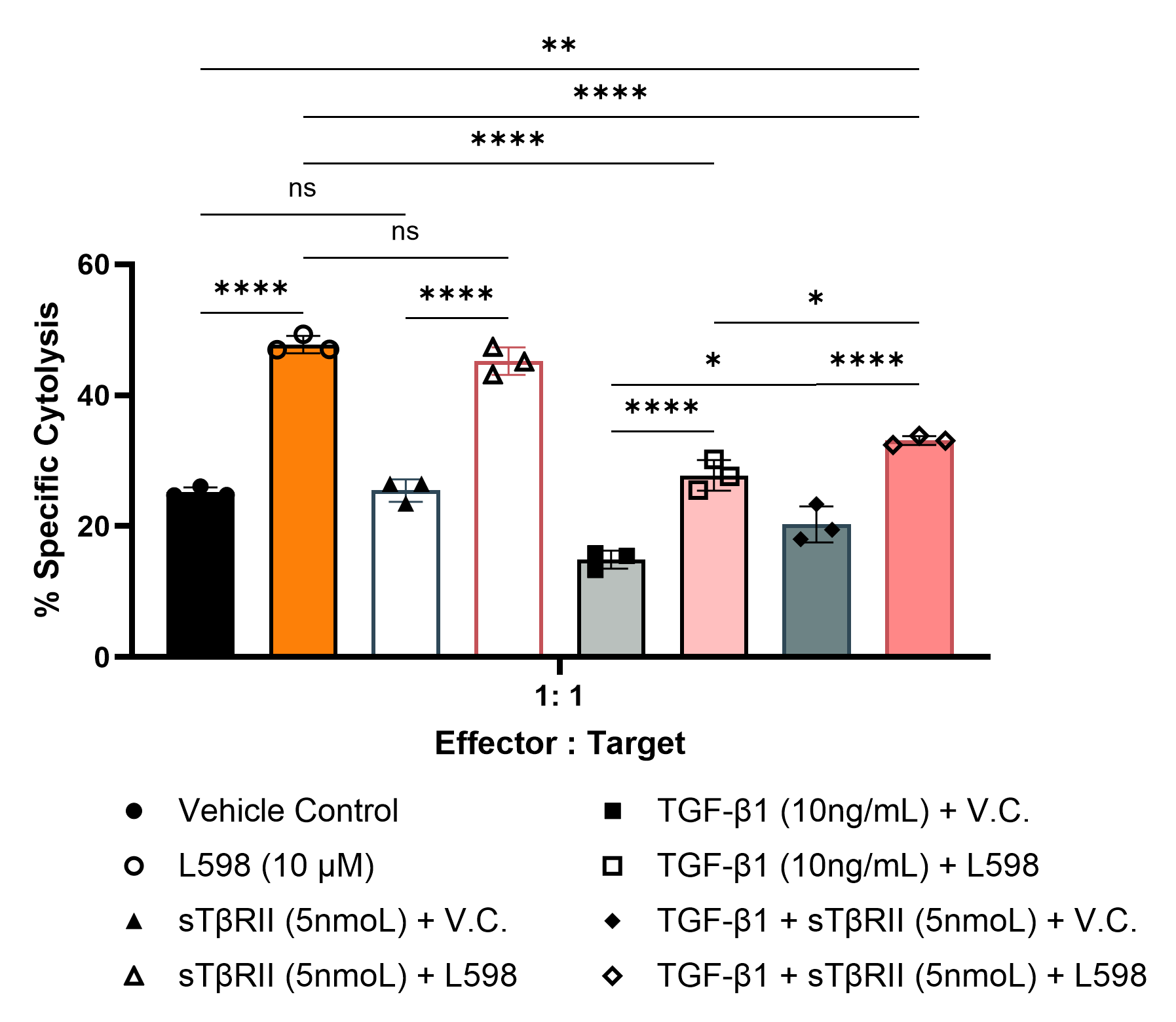

Supplement: Supplementary file 7 — Source data Fig. 5 [file 44319_2026_745_MOESM7_ESM.zip › Figure 5/5E/5E.tif]

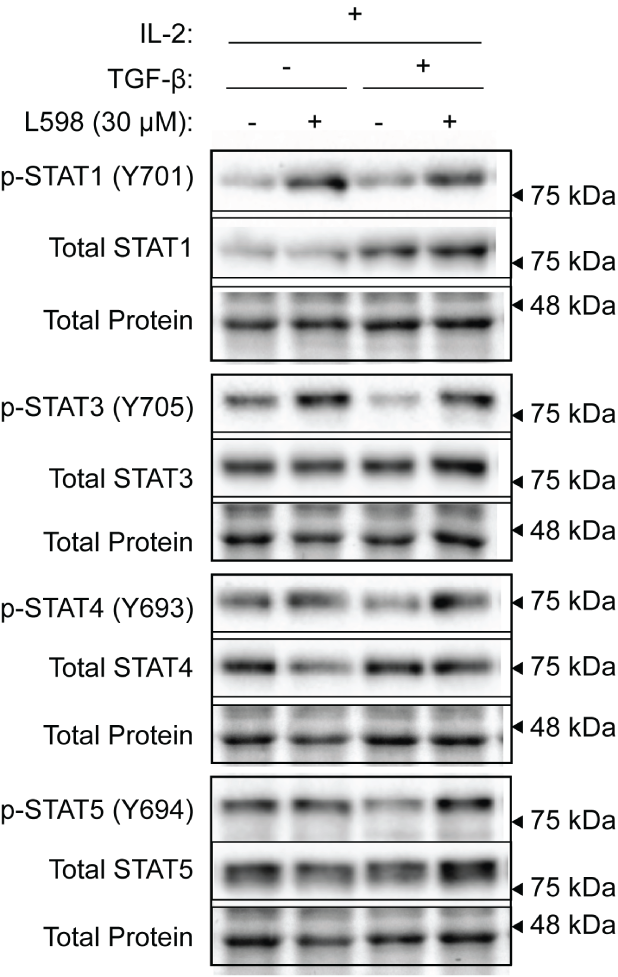

Supplement: Supplementary file 7 — Source data Fig. 5 [file 44319_2026_745_MOESM7_ESM.zip › Figure 5/5F/5F.tif]

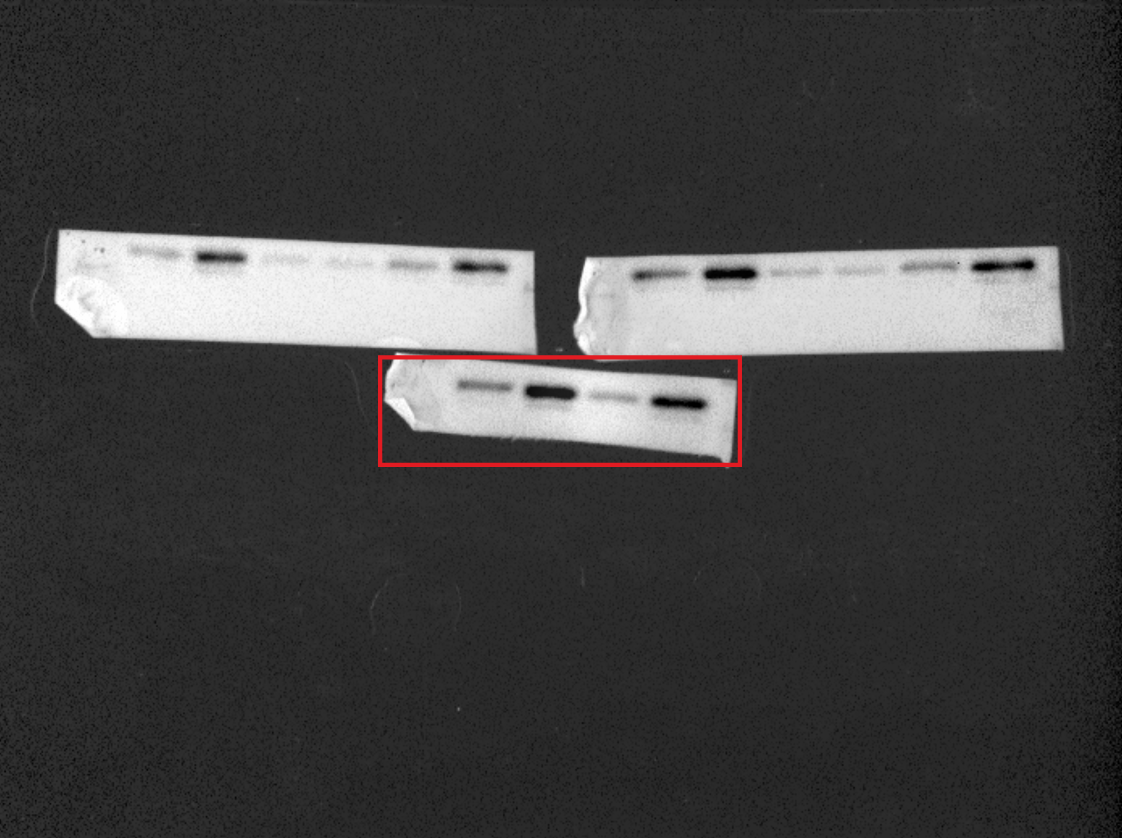

Supplement: Supplementary file 7 — Source data Fig. 5 [file 44319_2026_745_MOESM7_ESM.zip › Figure 5/5F/Repeats 5F/EXP3/p-STAT1_15.3sec+colori Gate.tif]

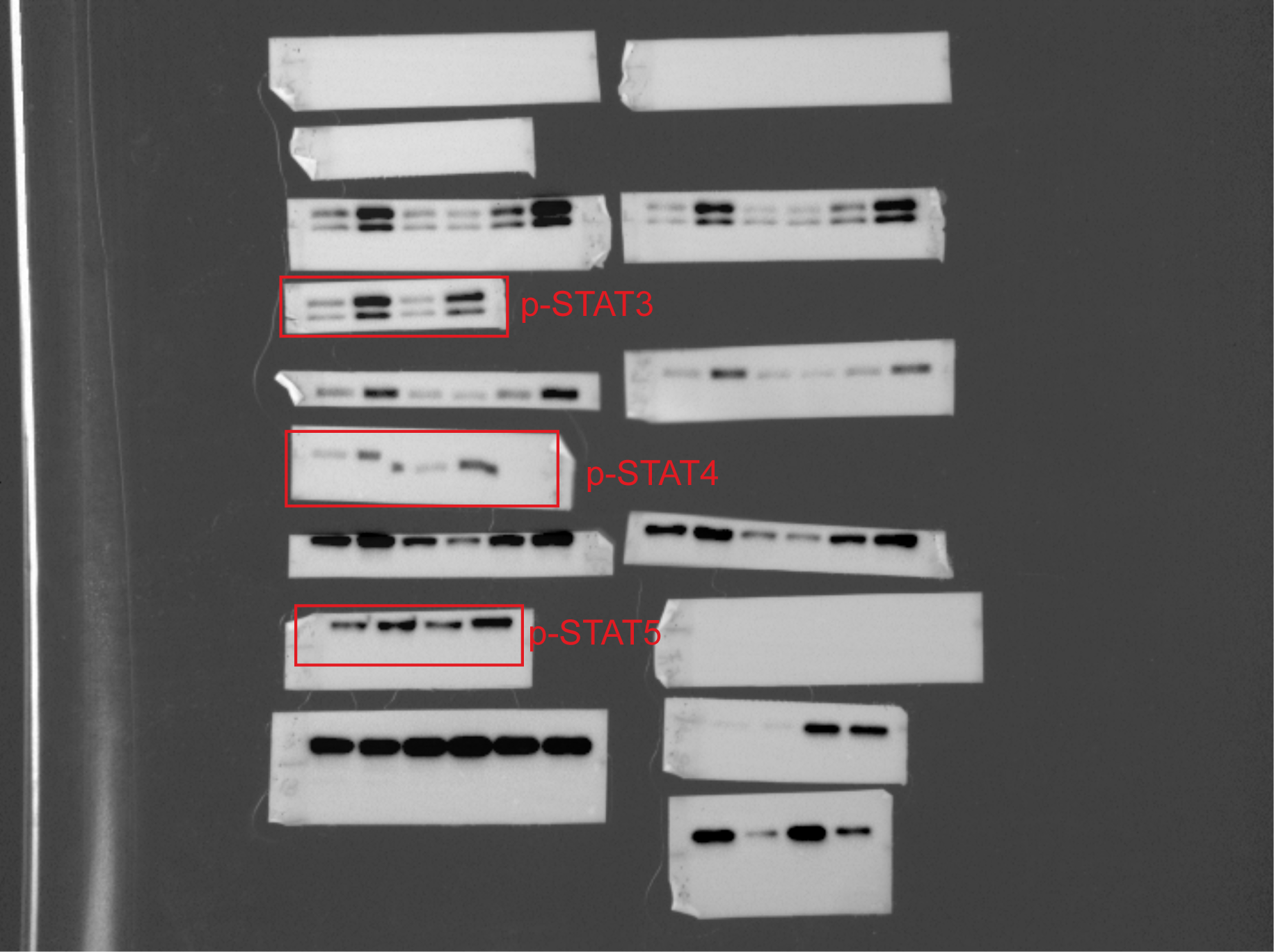

Supplement: Supplementary file 7 — Source data Fig. 5 [file 44319_2026_745_MOESM7_ESM.zip › Figure 5/5F/Repeats 5F/EXP3/pSTAT3, pSTAT4, pSTAT5_134.1sec+colori Gate.tif]

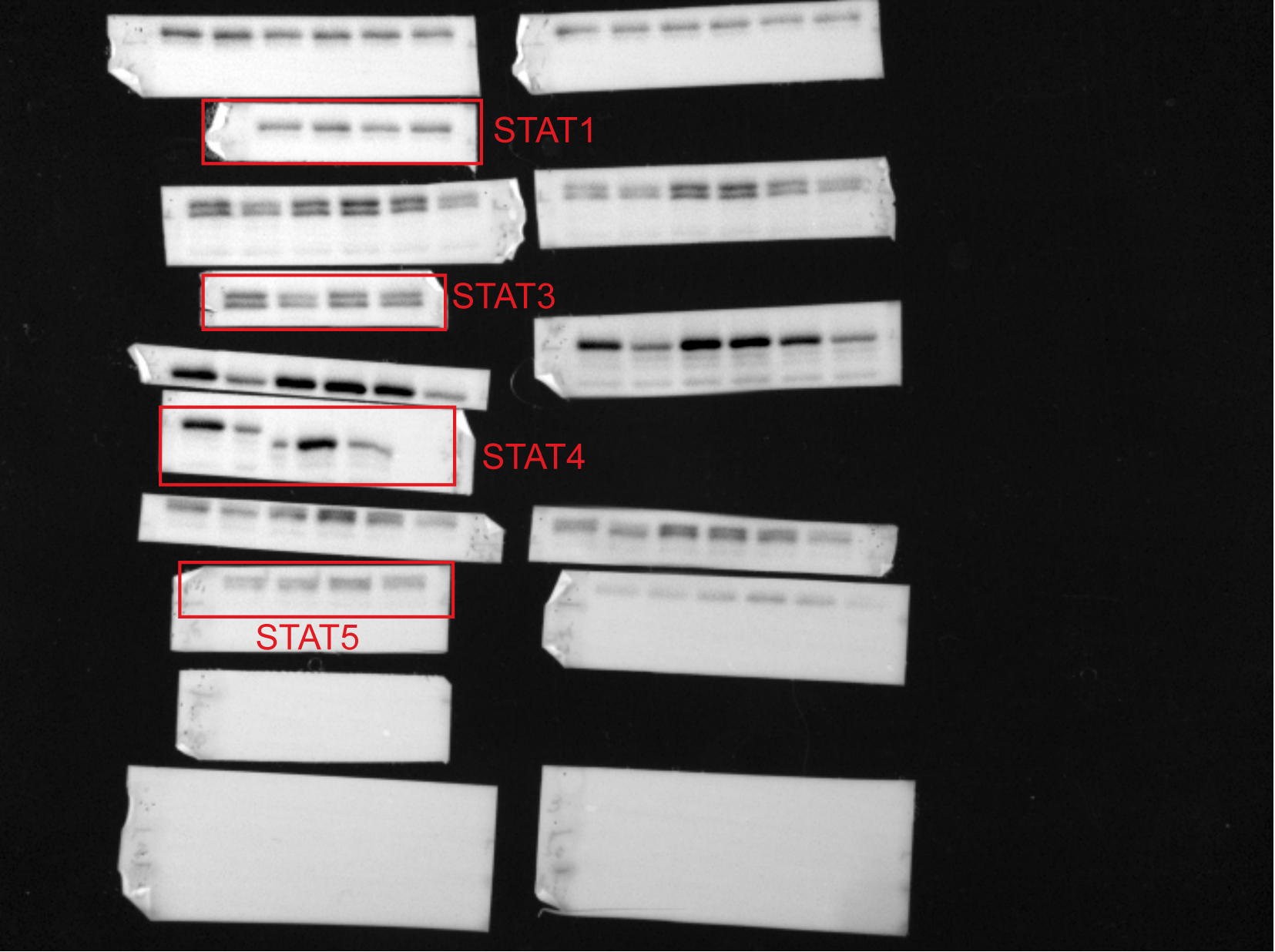

Supplement: Supplementary file 7 — Source data Fig. 5 [file 44319_2026_745_MOESM7_ESM.zip › Figure 5/5F/Repeats 5F/EXP3/Total STAT1 STAT3 STAT4 STAT5_3.0sec+COLORI Gate.tif]

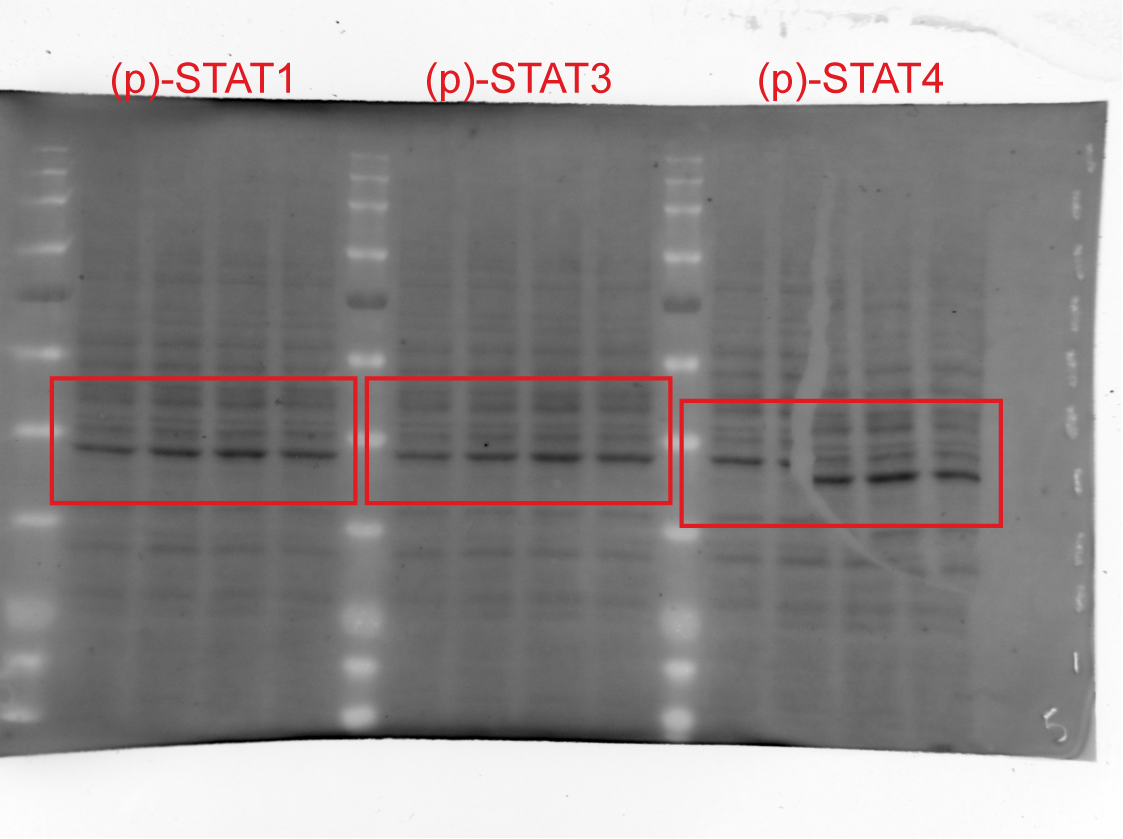

Supplement: Supplementary file 7 — Source data Fig. 5 [file 44319_2026_745_MOESM7_ESM.zip › Figure 5/5F/Repeats 5F/EXP3/Total Protein for TGFb_gel 5 Gate.tif]

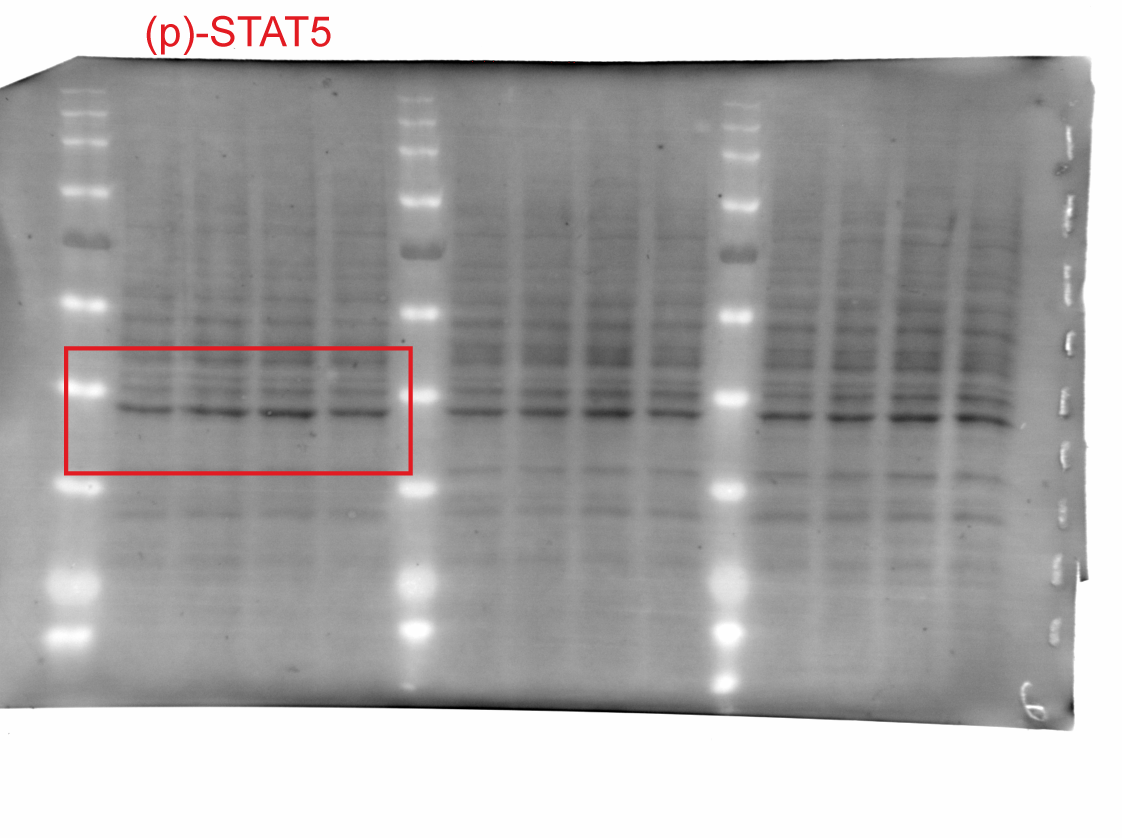

Supplement: Supplementary file 7 — Source data Fig. 5 [file 44319_2026_745_MOESM7_ESM.zip › Figure 5/5F/Repeats 5F/EXP3/Total Protein for TGFB_gel 6 Gate.tif]

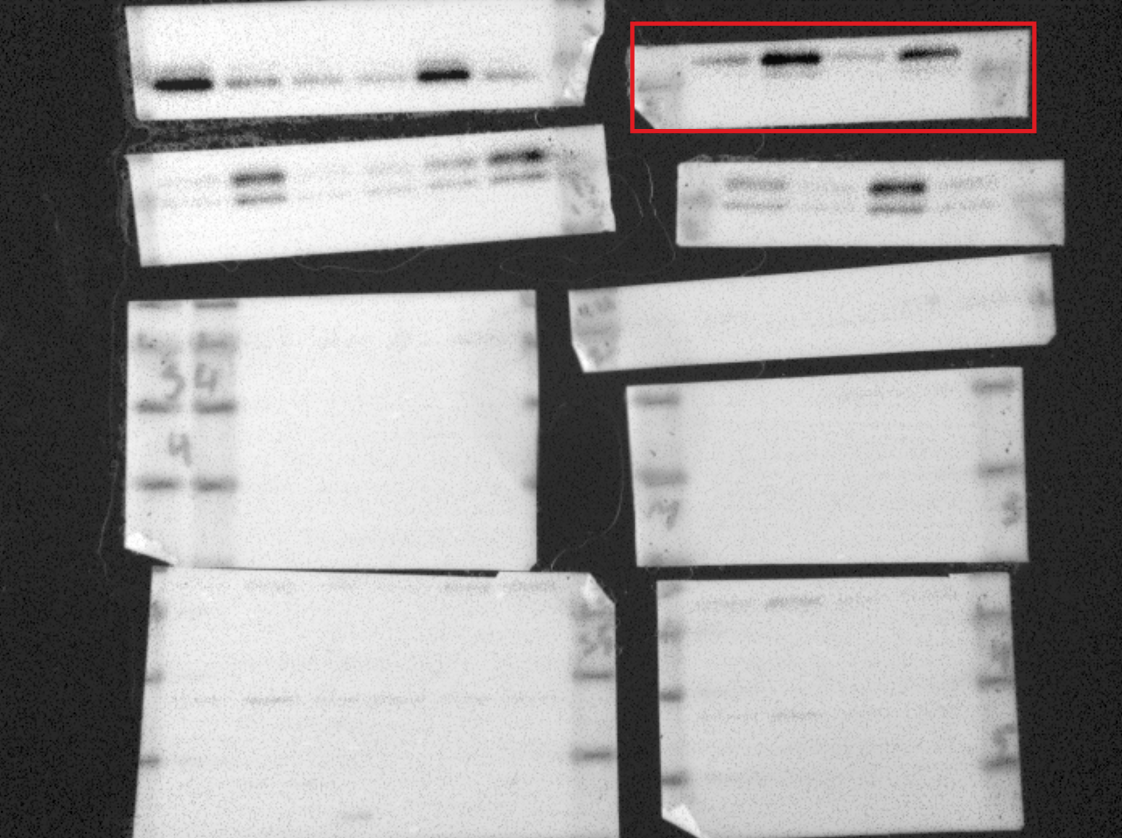

Supplement: Supplementary file 7 — Source data Fig. 5 [file 44319_2026_745_MOESM7_ESM.zip › Figure 5/5F/Repeats 5F/EXP1/pSTAT1_1.0sec+colori Gate.tif]

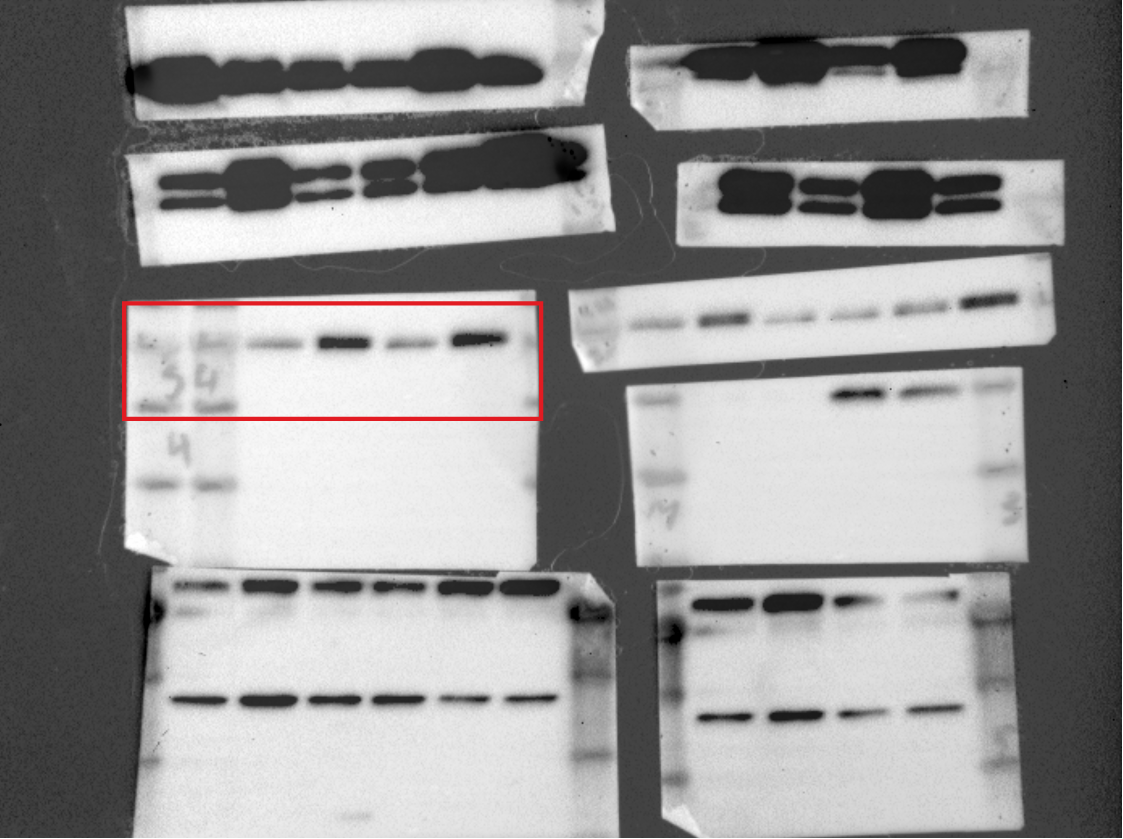

Supplement: Supplementary file 7 — Source data Fig. 5 [file 44319_2026_745_MOESM7_ESM.zip › Figure 5/5F/Repeats 5F/EXP1/pSTAT4_394.6sec+colori Gate.tif]

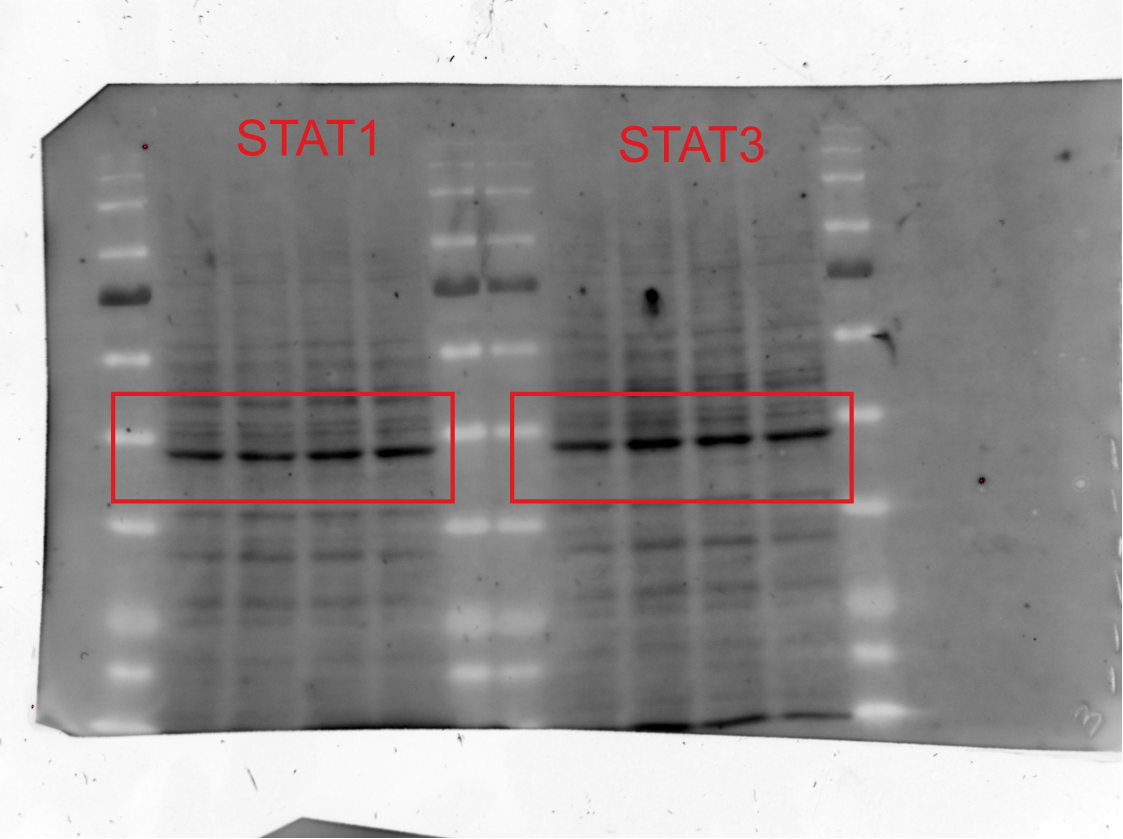

Supplement: Supplementary file 7 — Source data Fig. 5 [file 44319_2026_745_MOESM7_ESM.zip › Figure 5/5F/Repeats 5F/EXP1/TOTAL g3 tgfb1 l598 stat1 stat3 Gate.tif]

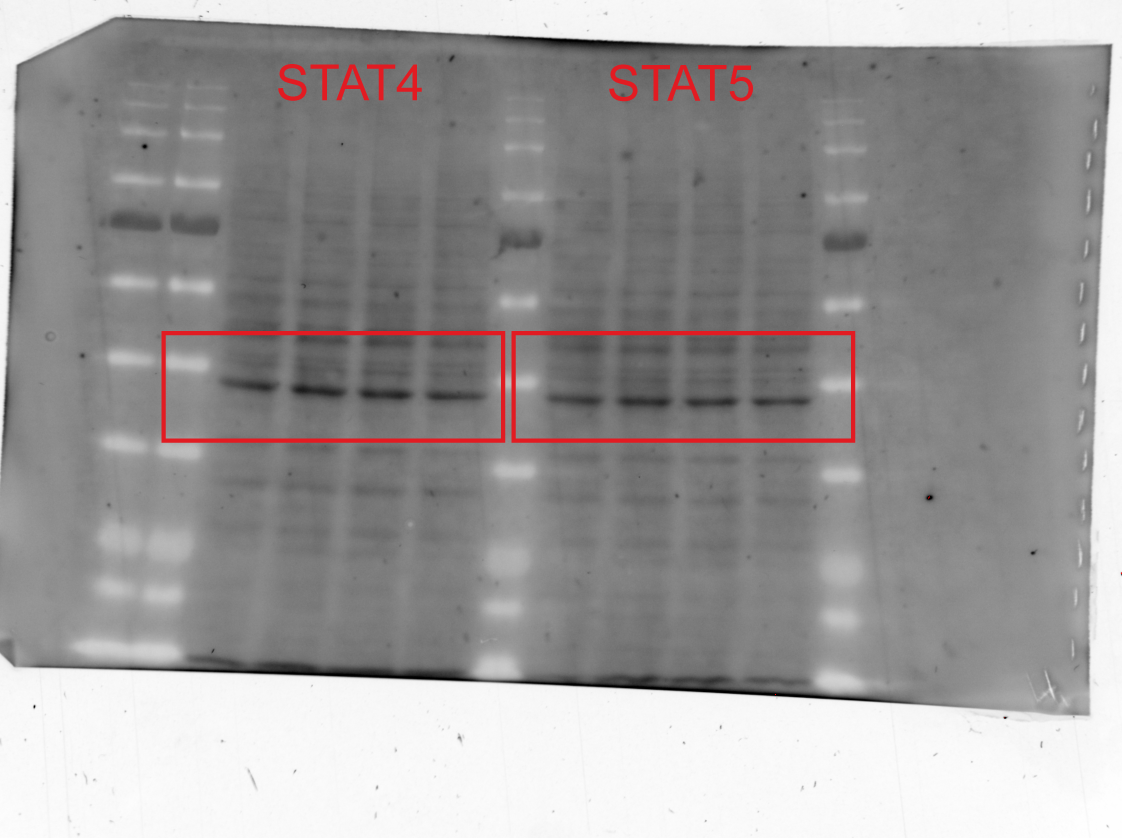

Supplement: Supplementary file 7 — Source data Fig. 5 [file 44319_2026_745_MOESM7_ESM.zip › Figure 5/5F/Repeats 5F/EXP1/TOTAL g4 tgfb1 l598 stat4 stat5 Gate.tif]

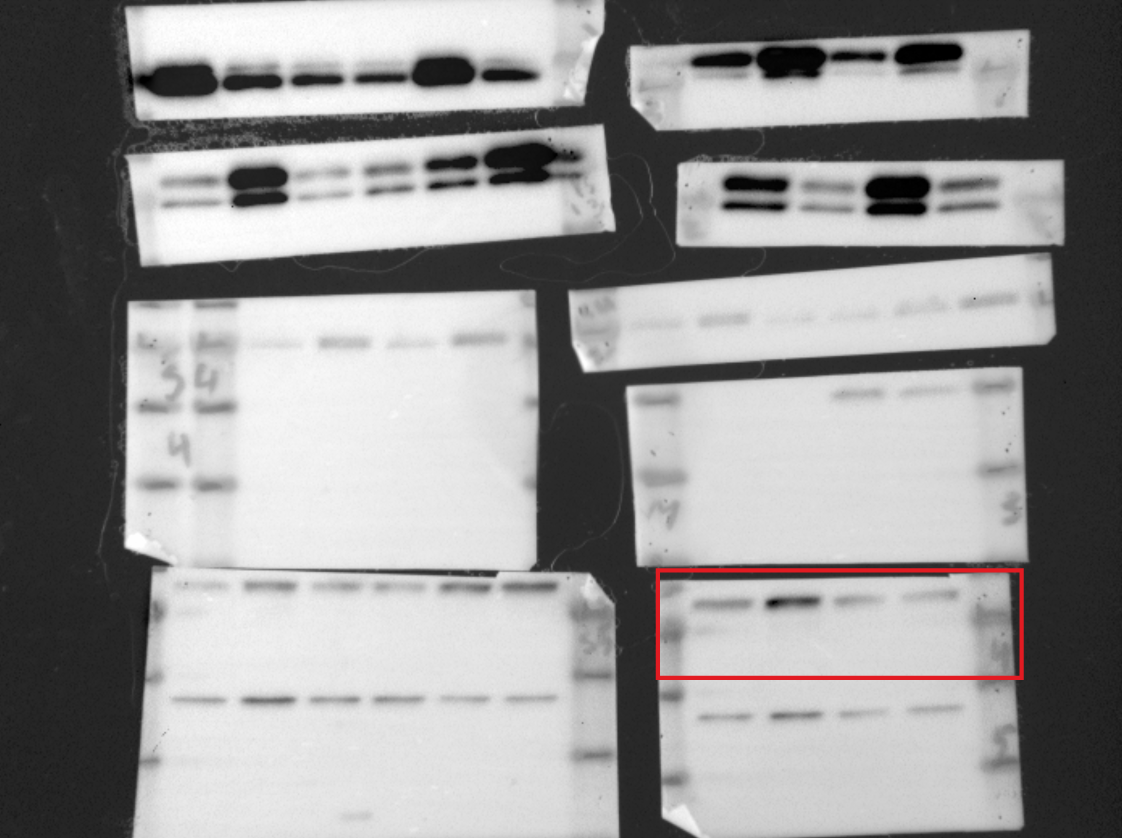

Supplement: Supplementary file 7 — Source data Fig. 5 [file 44319_2026_745_MOESM7_ESM.zip › Figure 5/5F/Repeats 5F/EXP1/pSTAT5_89.9sec+colori Gate NOT.tif]

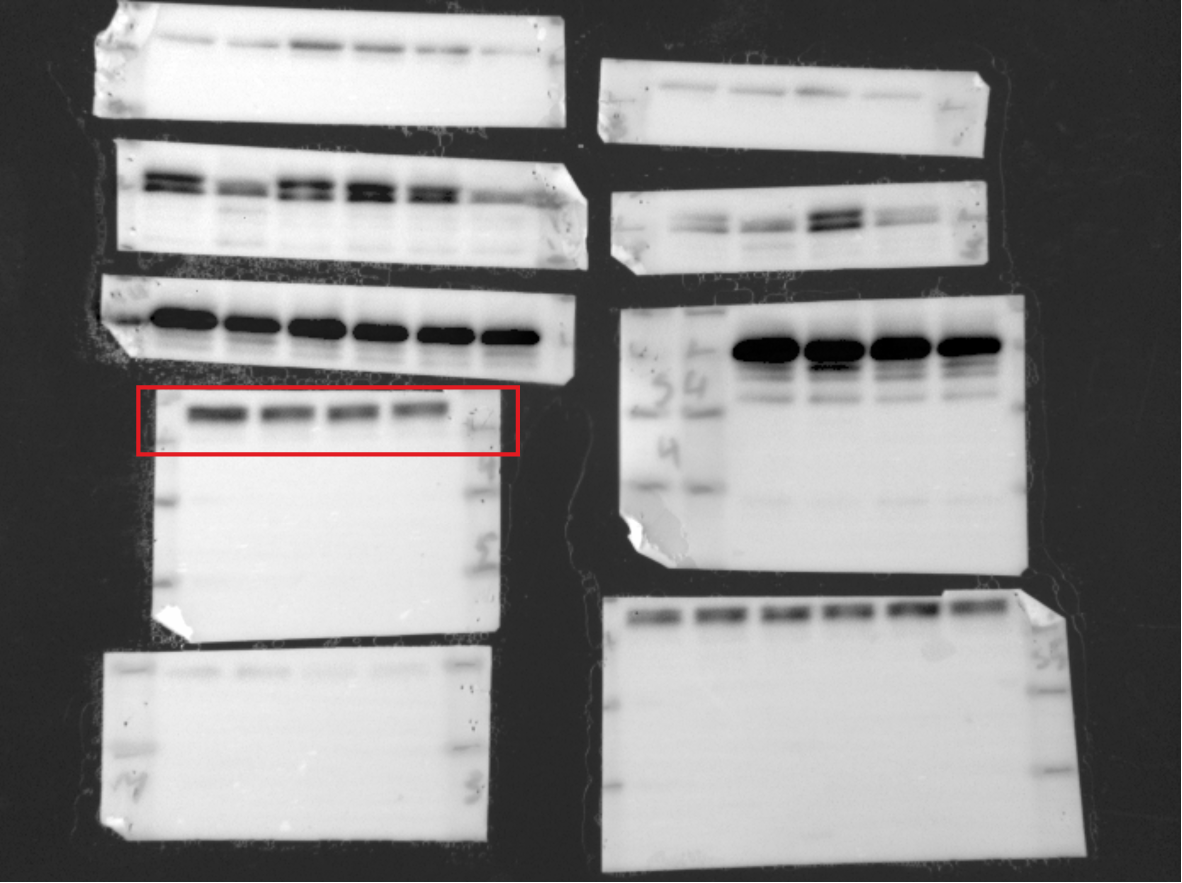

Supplement: Supplementary file 7 — Source data Fig. 5 [file 44319_2026_745_MOESM7_ESM.zip › Figure 5/5F/Repeats 5F/EXP1/tSTAT5_28.2sec+colori Gate.tif]

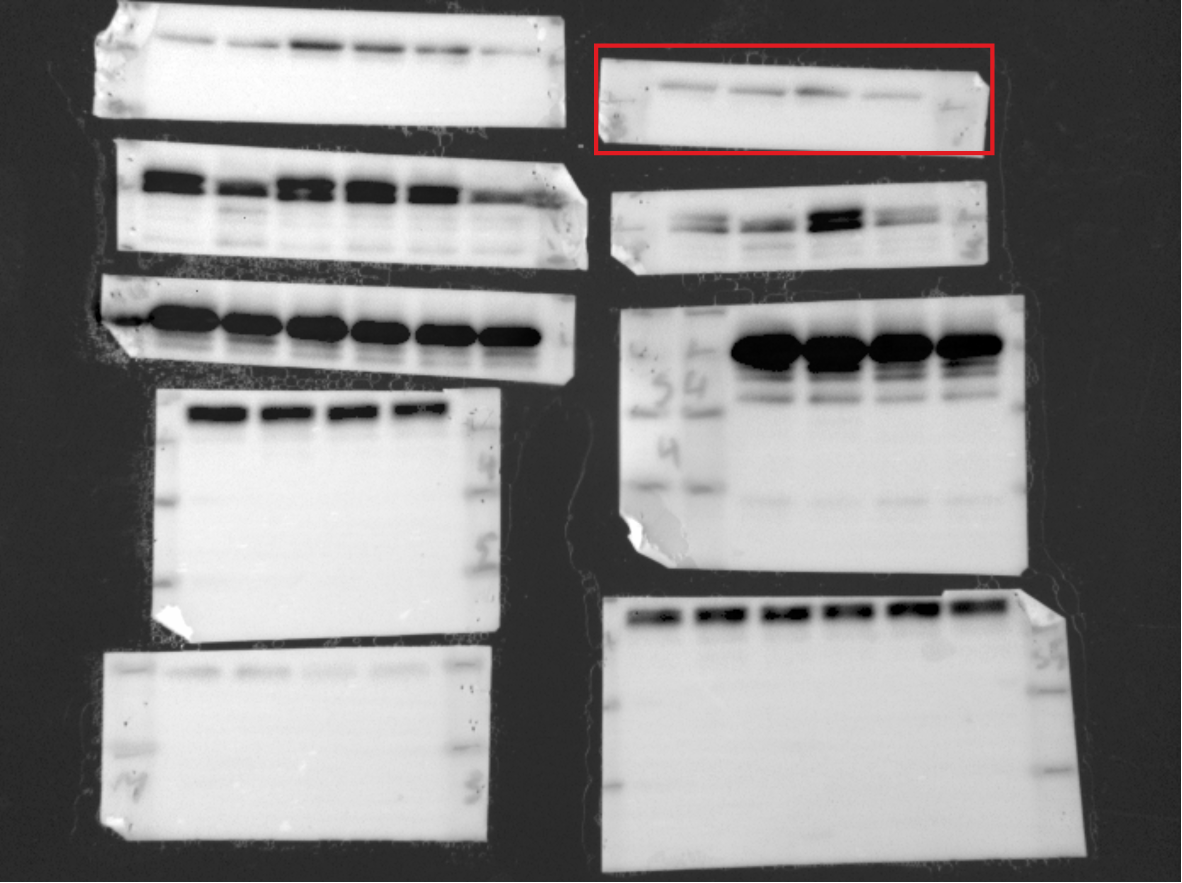

Supplement: Supplementary file 7 — Source data Fig. 5 [file 44319_2026_745_MOESM7_ESM.zip › Figure 5/5F/Repeats 5F/EXP1/tSTAT1_colori+1_43.3sec Gate.tif]

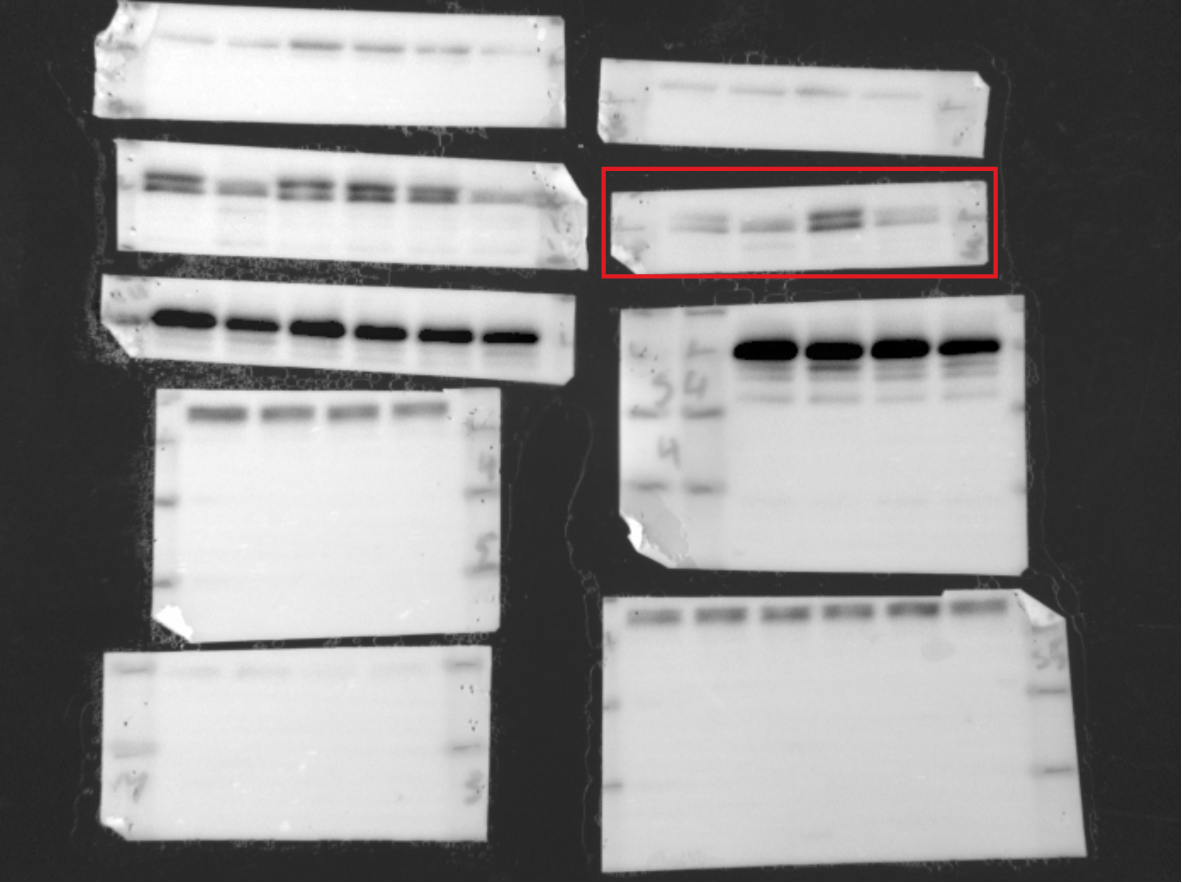

Supplement: Supplementary file 7 — Source data Fig. 5 [file 44319_2026_745_MOESM7_ESM.zip › Figure 5/5F/Repeats 5F/EXP1/tSTAT3_19.1sec+colori Gate.tif]

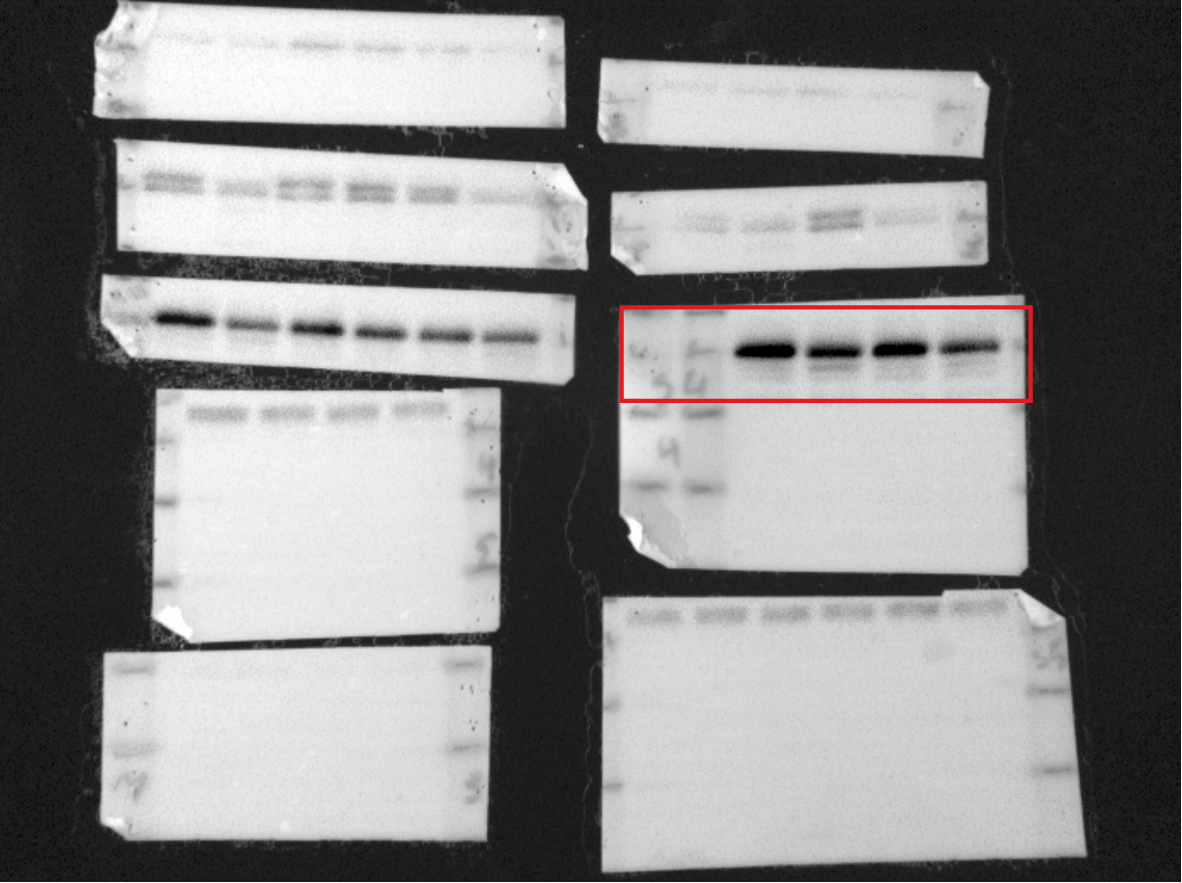

Supplement: Supplementary file 7 — Source data Fig. 5 [file 44319_2026_745_MOESM7_ESM.zip › Figure 5/5F/Repeats 5F/EXP1/tSTAT4_1.0sec+colori Gate.tif]

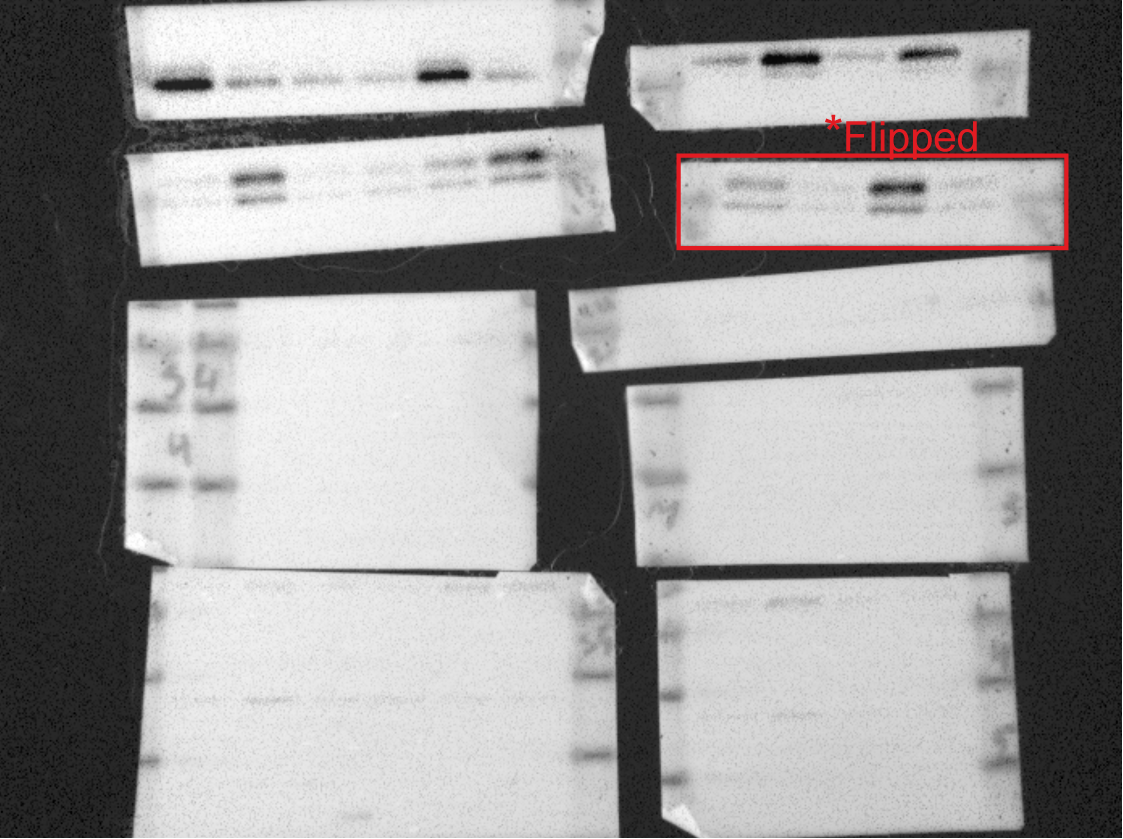

Supplement: Supplementary file 7 — Source data Fig. 5 [file 44319_2026_745_MOESM7_ESM.zip › Figure 5/5F/Repeats 5F/EXP1/pSTAT3_1.0sec+colori Gate.tif]

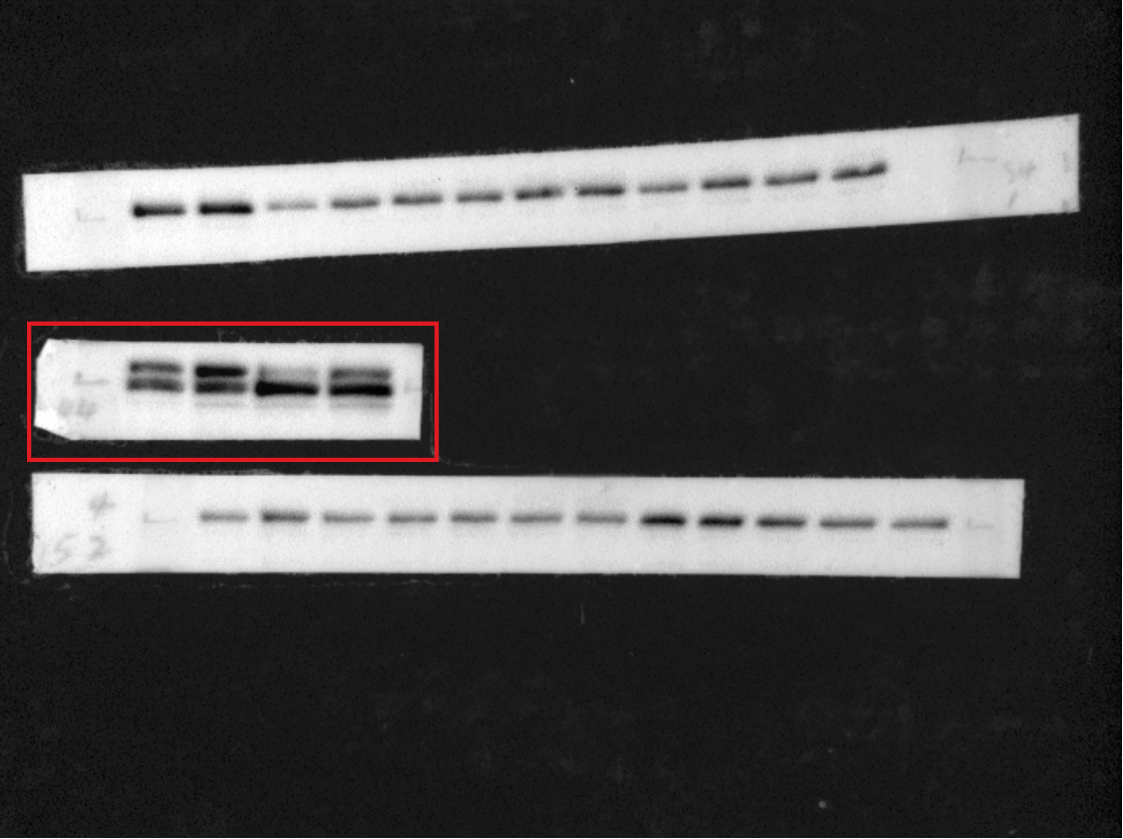

Supplement: Supplementary file 7 — Source data Fig. 5 [file 44319_2026_745_MOESM7_ESM.zip › Figure 5/5F/Repeats 5F/EXP2/p-STAT1_TGFB_53.3sec+COLORI 2 R Gate.tif]

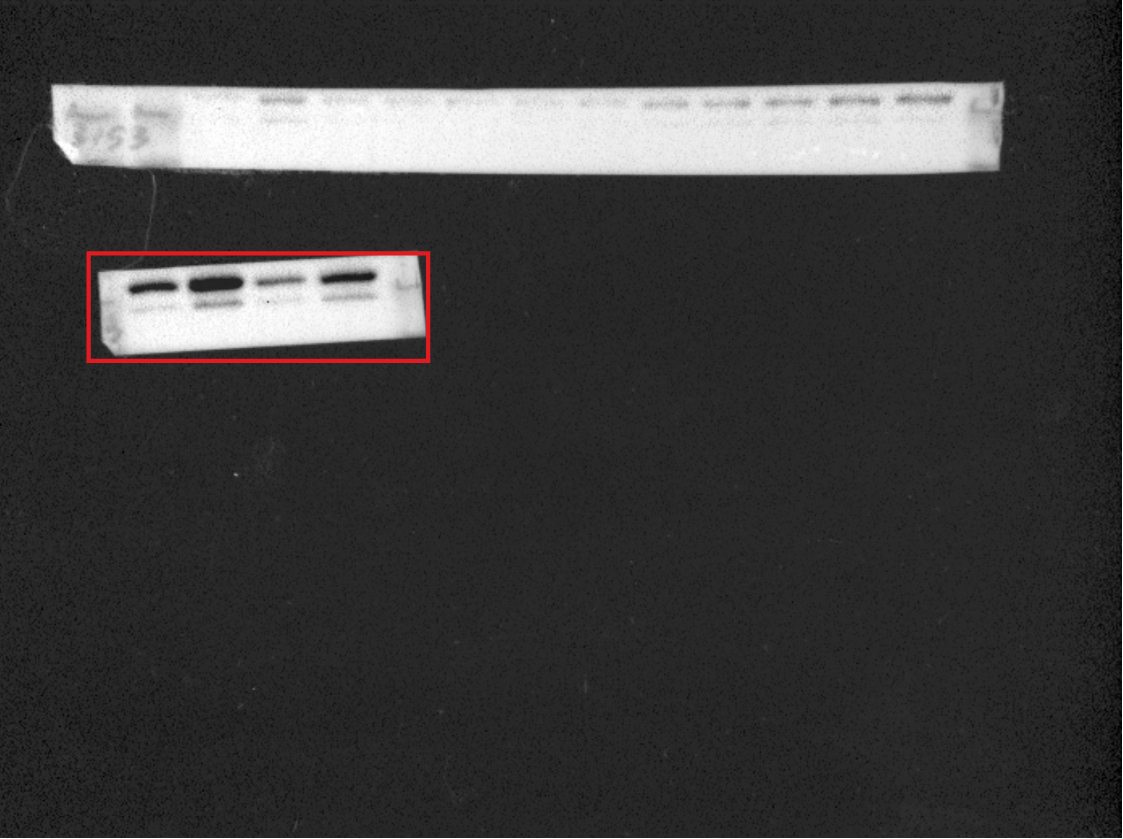

Supplement: Supplementary file 7 — Source data Fig. 5 [file 44319_2026_745_MOESM7_ESM.zip › Figure 5/5F/Repeats 5F/EXP2/p-STAT3 TGFb_204.3sec+COLORIn Gate.tif]

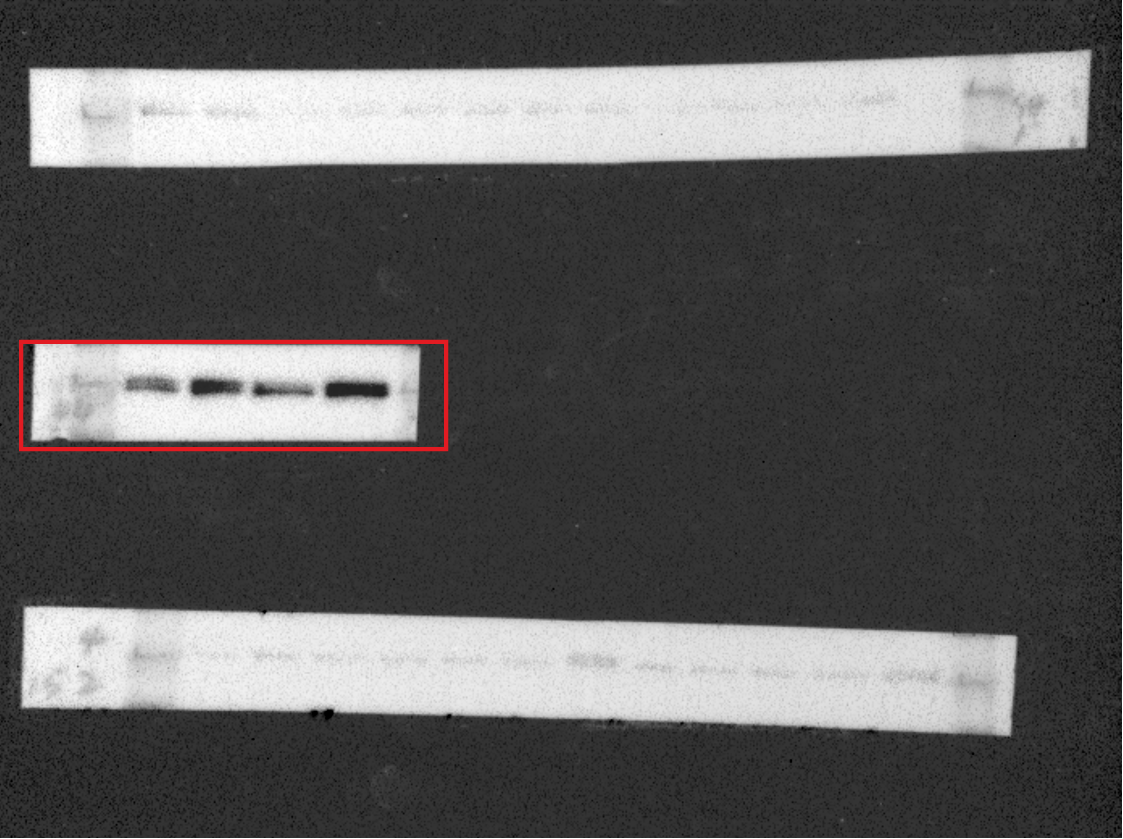

Supplement: Supplementary file 7 — Source data Fig. 5 [file 44319_2026_745_MOESM7_ESM.zip › Figure 5/5F/Repeats 5F/EXP2/pSTAT4_TGFB_colori+37.3sec Gate.tif]

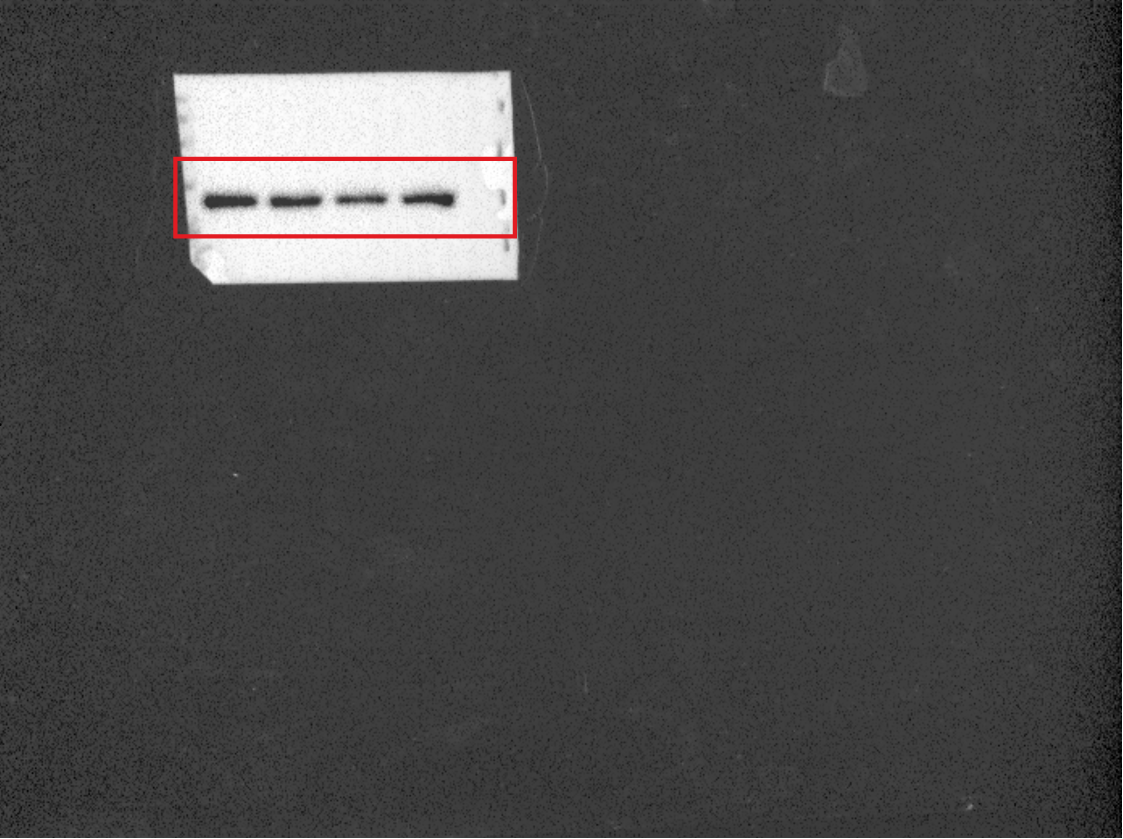

Supplement: Supplementary file 7 — Source data Fig. 5 [file 44319_2026_745_MOESM7_ESM.zip › Figure 5/5F/Repeats 5F/EXP2/pSTAT5_TGFB_117.0sec+colori Gate.tif]

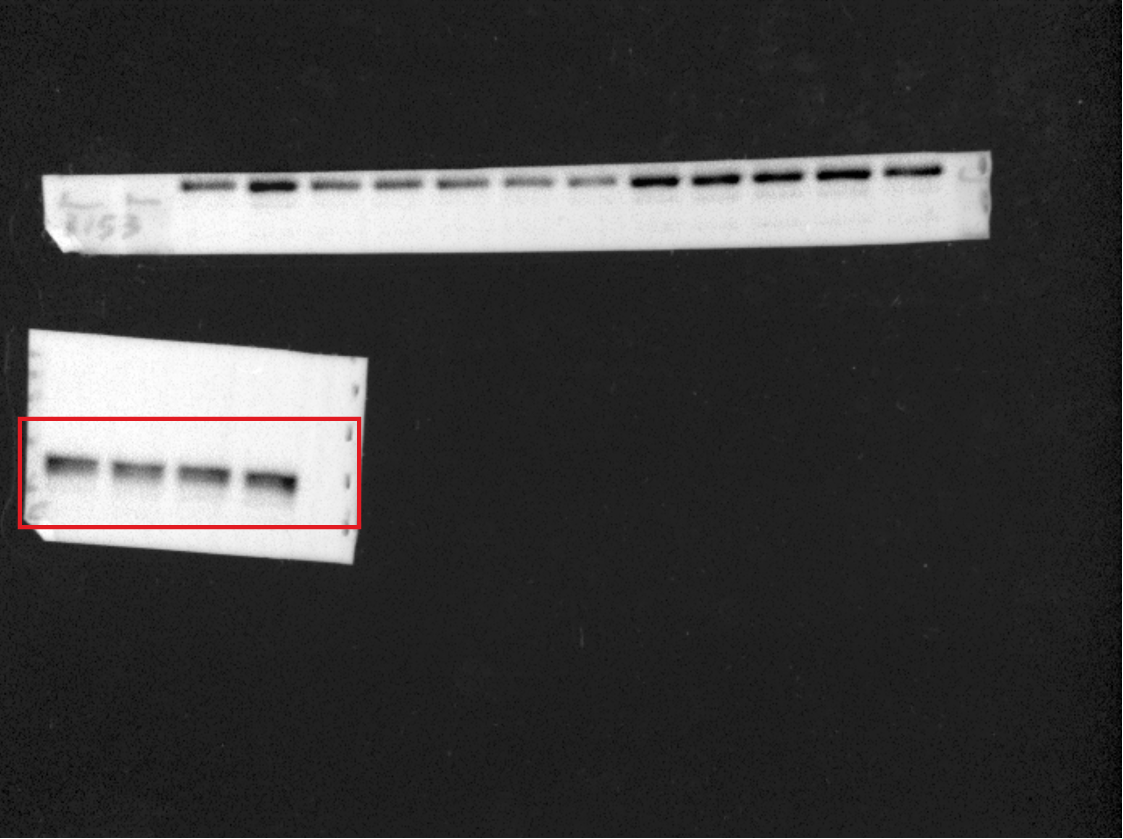

Supplement: Supplementary file 7 — Source data Fig. 5 [file 44319_2026_745_MOESM7_ESM.zip › Figure 5/5F/Repeats 5F/EXP2/total STAT5_TGFb_7.1sec+colori Gate.tif]

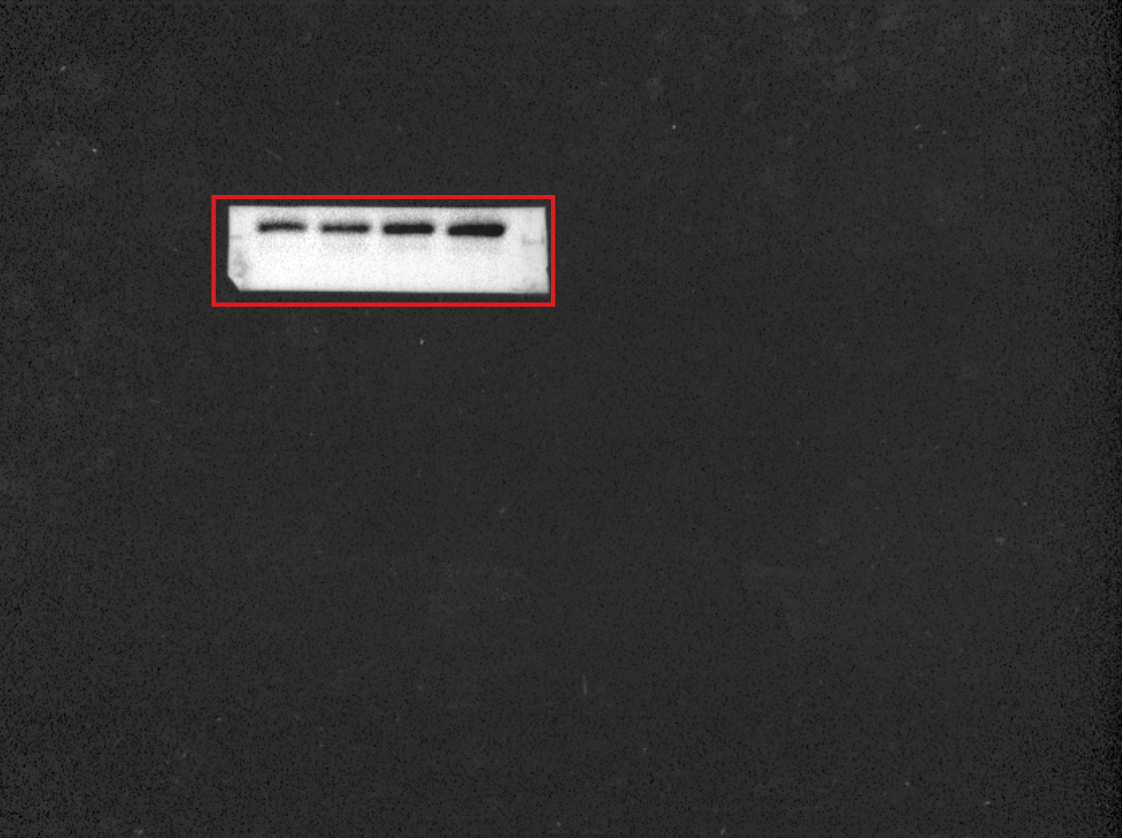

Supplement: Supplementary file 7 — Source data Fig. 5 [file 44319_2026_745_MOESM7_ESM.zip › Figure 5/5F/Repeats 5F/EXP2/Total STAT3_TGFB_2.4sec+colori Gate.tif]
