# Supplementary figures and images for: PTPN1/PTPN2 inhibition improves NK cancer therapy by enhancing IL-2 and mitigating TGFβ1 responses (part 3 of 3)
Source: EMBO Rep. 2026 Apr 15;27(10):2581–613. doi: 10.1038/s44319-026-00745-0 (PMC13219468; doi:10.1038/s44319-026-00745-0)

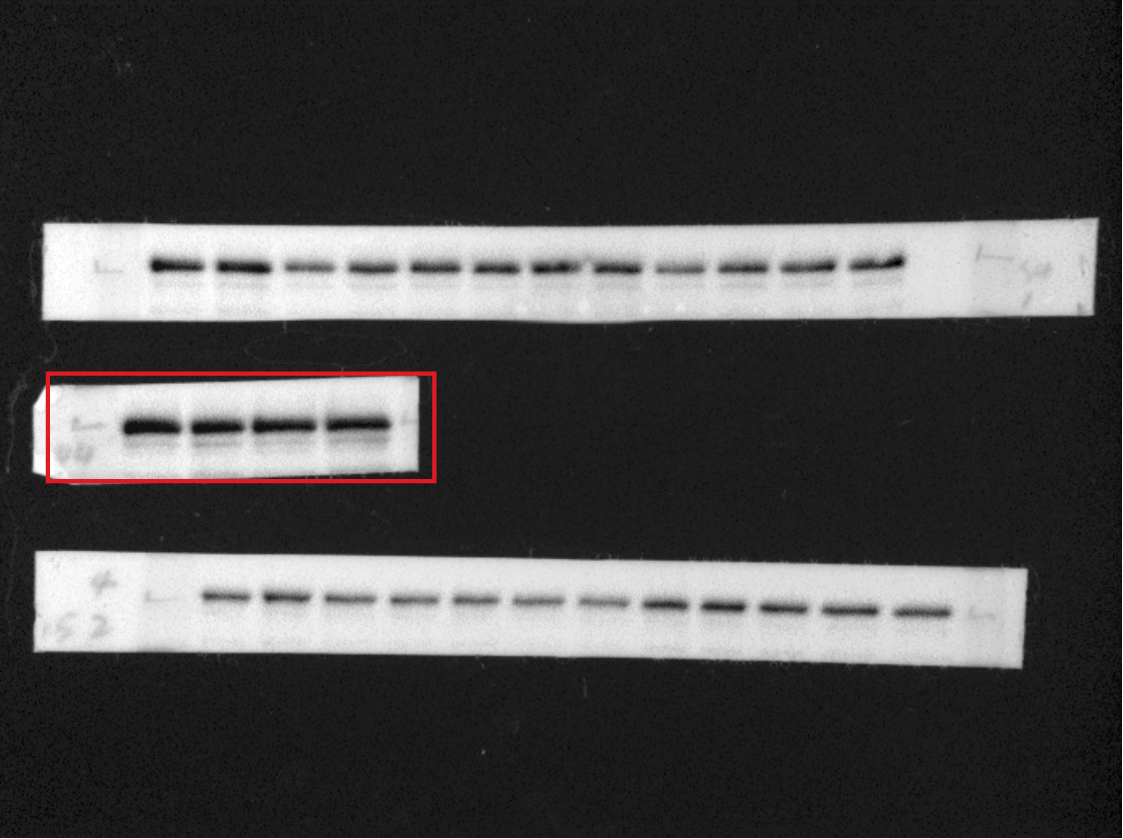

Supplement: Supplementary file 7 — Source data Fig. 5 [file 44319_2026_745_MOESM7_ESM.zip › Figure 5/5F/Repeats 5F/EXP2/Total STAT4_TGFb_2.6sec+COLORUI Gate.tif]

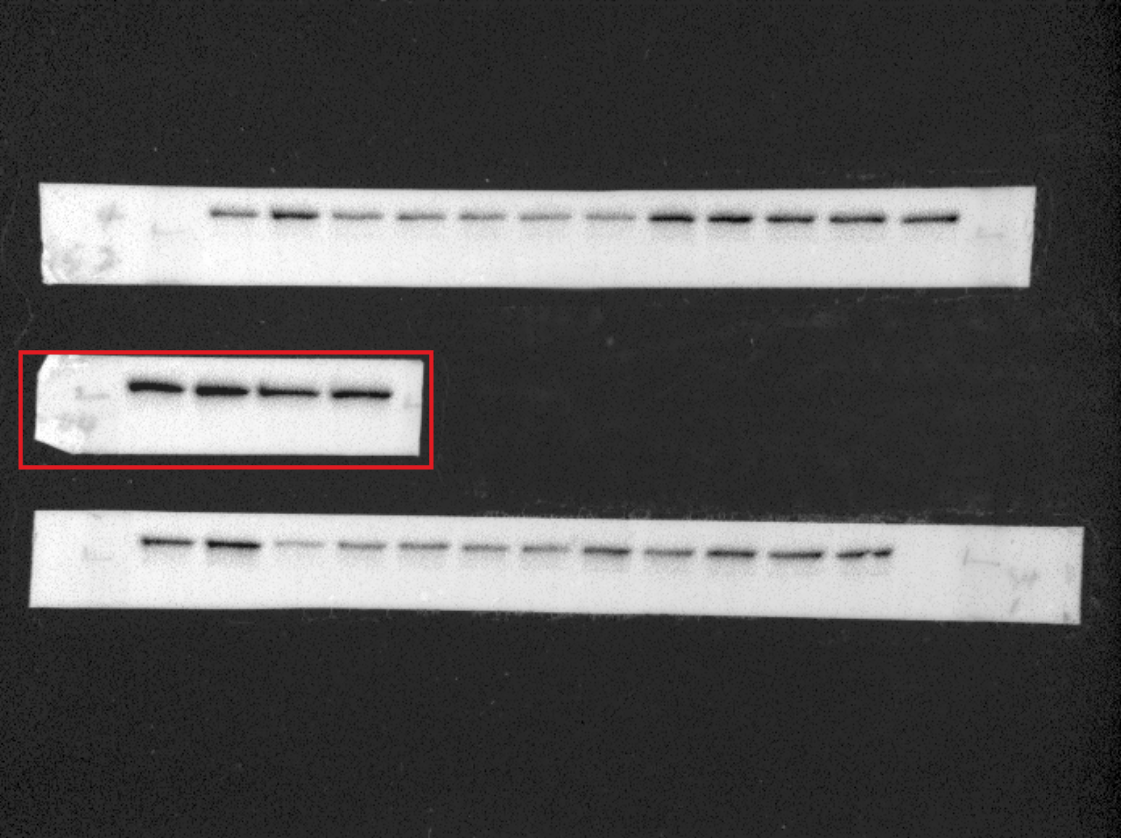

Supplement: Supplementary file 7 — Source data Fig. 5 [file 44319_2026_745_MOESM7_ESM.zip › Figure 5/5F/Repeats 5F/EXP2/Total STAT1_TGFB_8.0sec + colori Gate.tif]

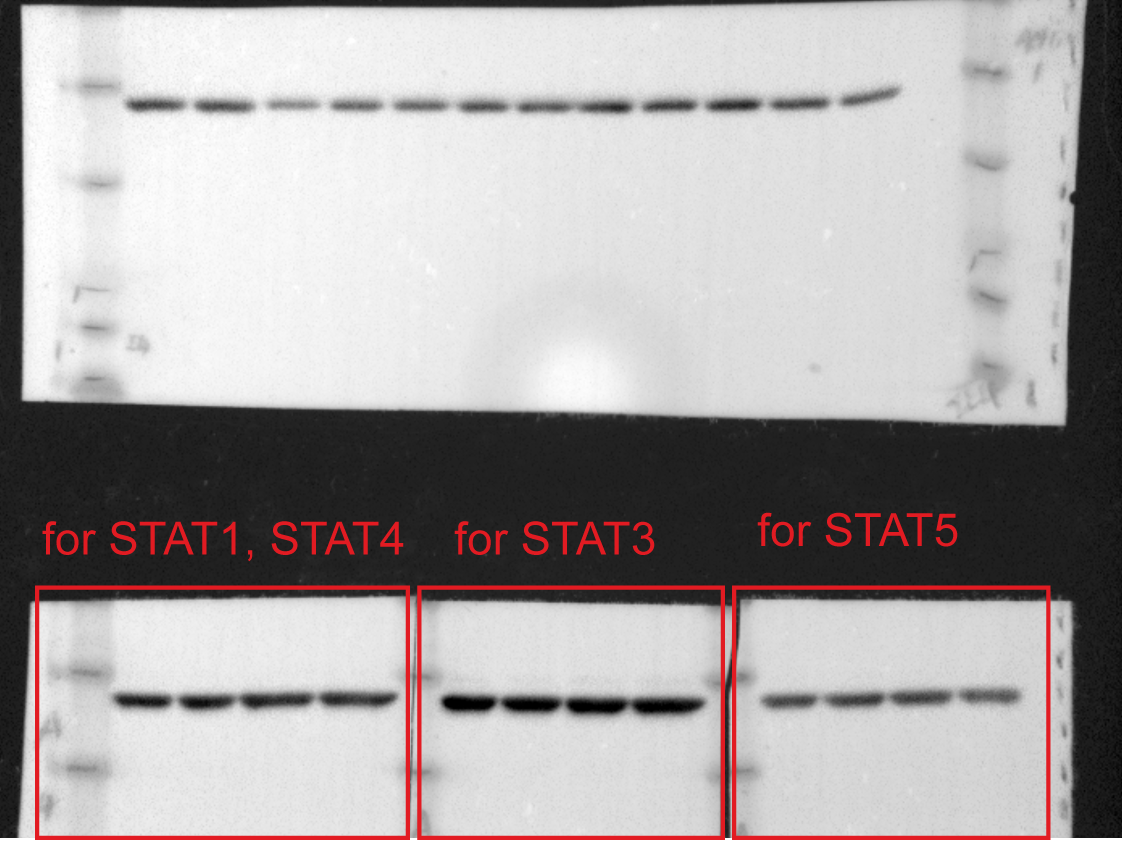

Supplement: Supplementary file 7 — Source data Fig. 5 [file 44319_2026_745_MOESM7_ESM.zip › Figure 5/5F/Repeats 5F/EXP2/B Actin_TGFB g4_3.4sec+colori_Gate.tif]

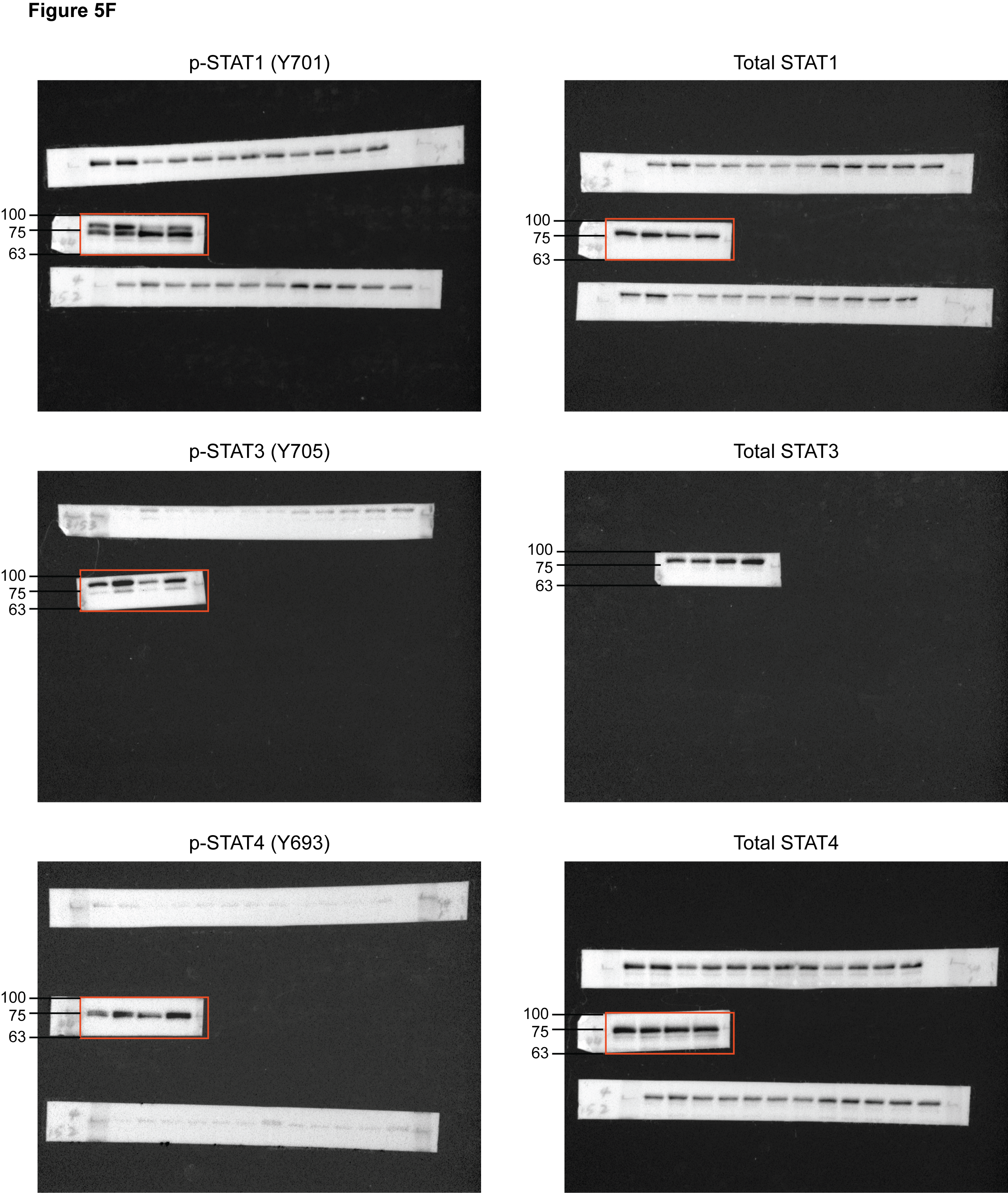

Supplement: Supplementary file 7 — Source data Fig. 5 [file 44319_2026_745_MOESM7_ESM.zip › Figure 5/5F/Repeats 5F/Molecular Weights/5F_STAT1_STAT3_STAT4.tif]

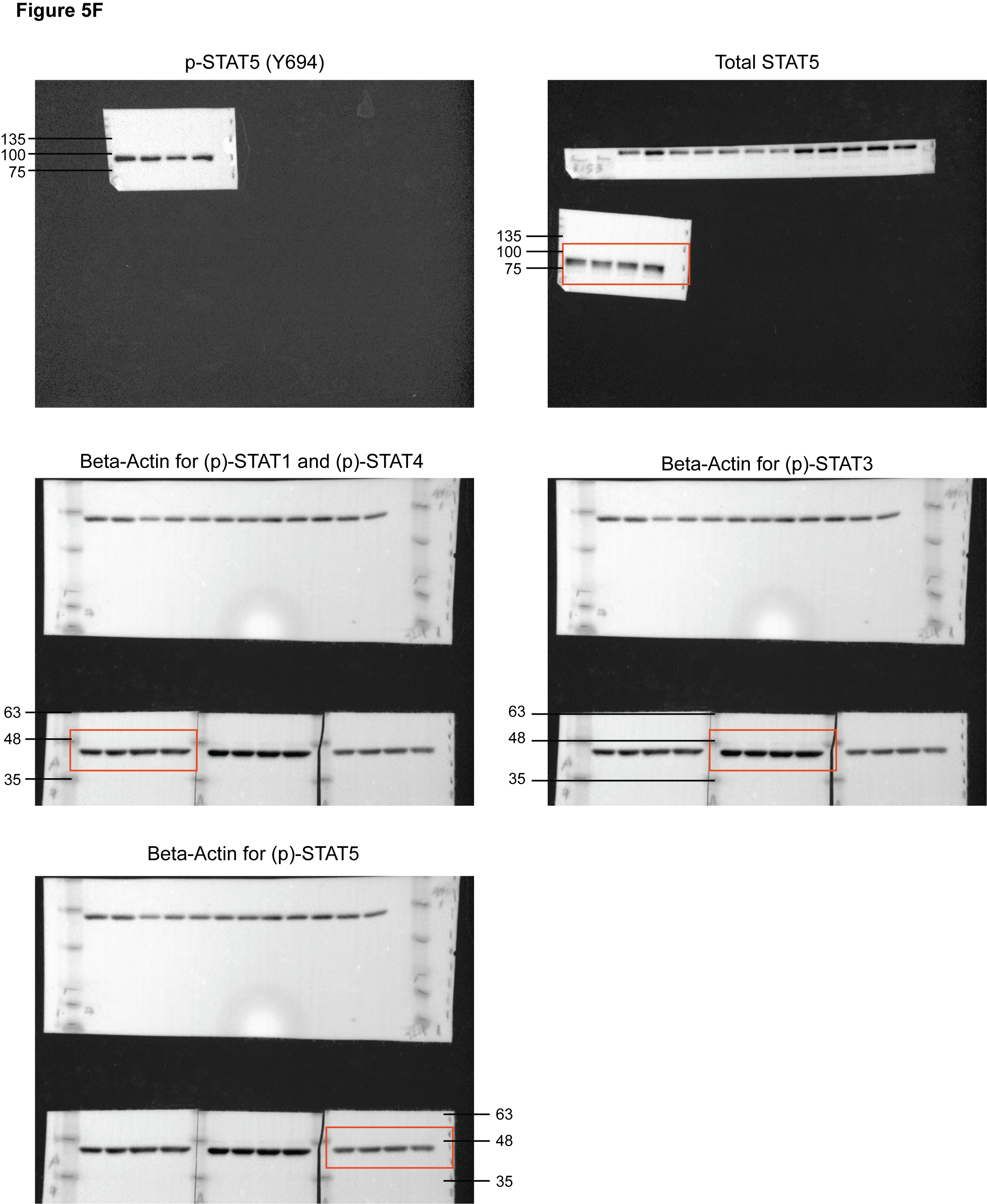

Supplement: Supplementary file 7 — Source data Fig. 5 [file 44319_2026_745_MOESM7_ESM.zip › Figure 5/5F/Repeats 5F/Molecular Weights/5F_STAT5_bActin.tif]

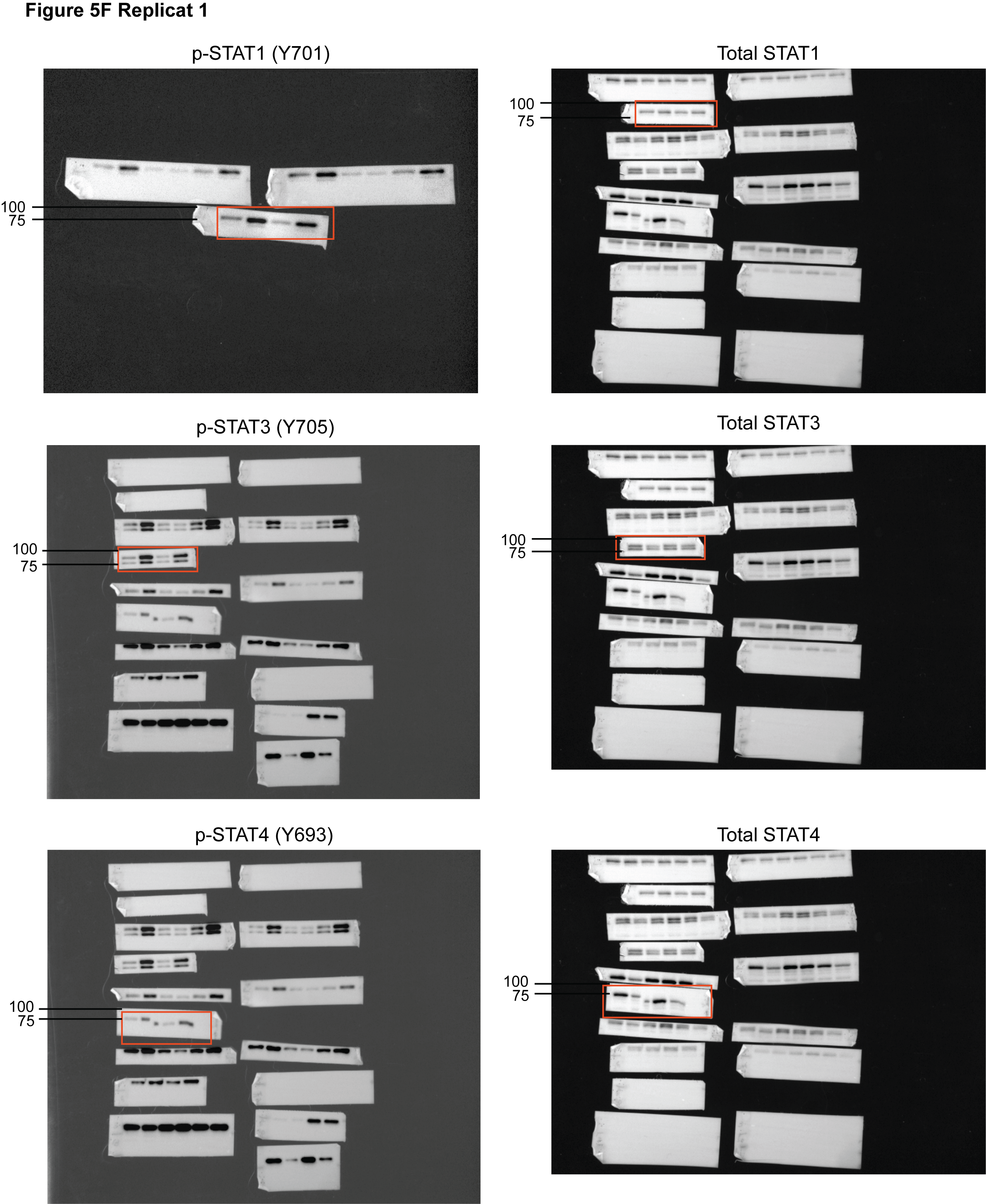

Supplement: Supplementary file 7 — Source data Fig. 5 [file 44319_2026_745_MOESM7_ESM.zip › Figure 5/5F/Repeats 5F/Molecular Weights/5F Replicate 1_STAT1_STAT3_STAT4.tif]

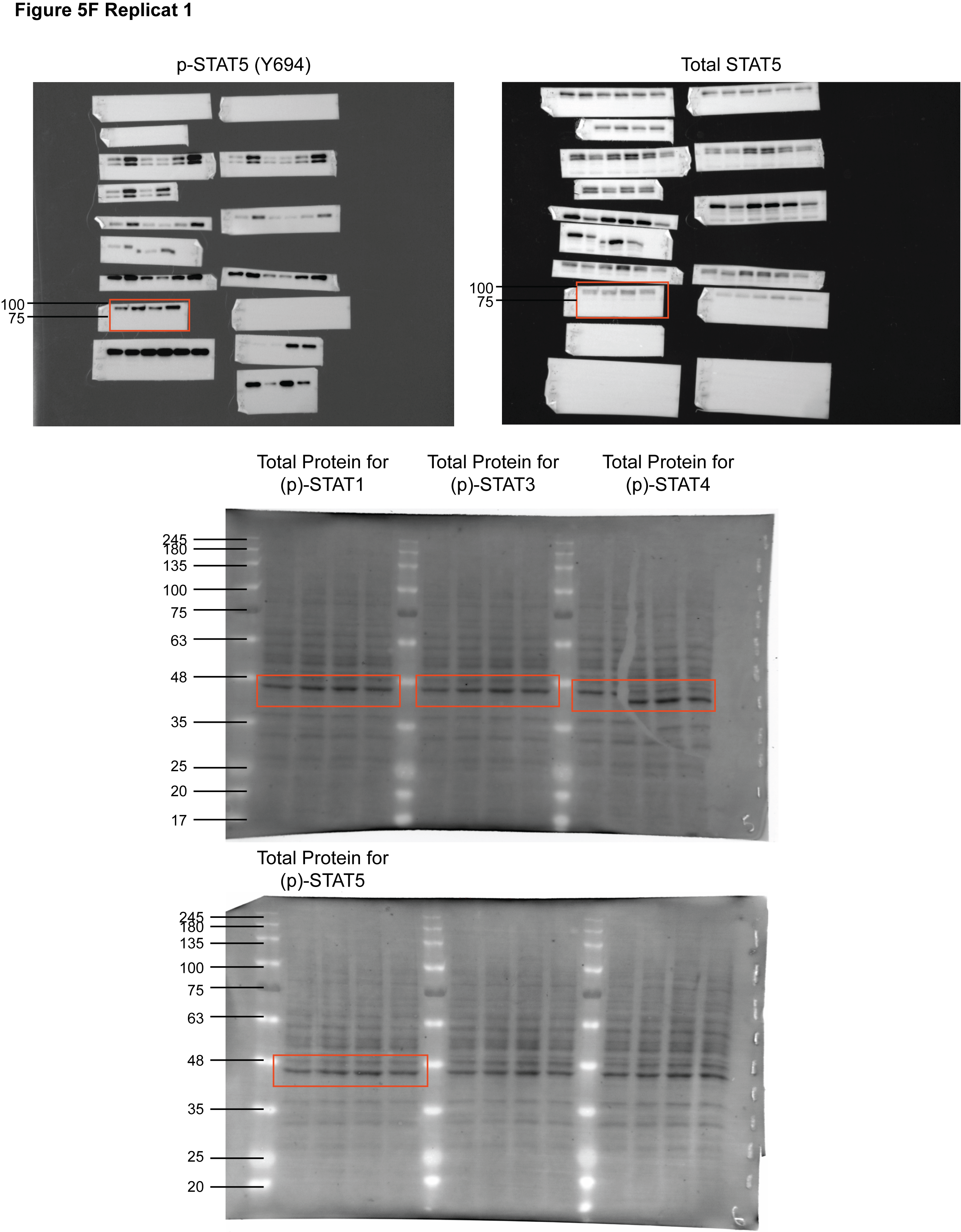

Supplement: Supplementary file 7 — Source data Fig. 5 [file 44319_2026_745_MOESM7_ESM.zip › Figure 5/5F/Repeats 5F/Molecular Weights/5F Replicate1_STAT5_Total Protein Loading Control.tif]

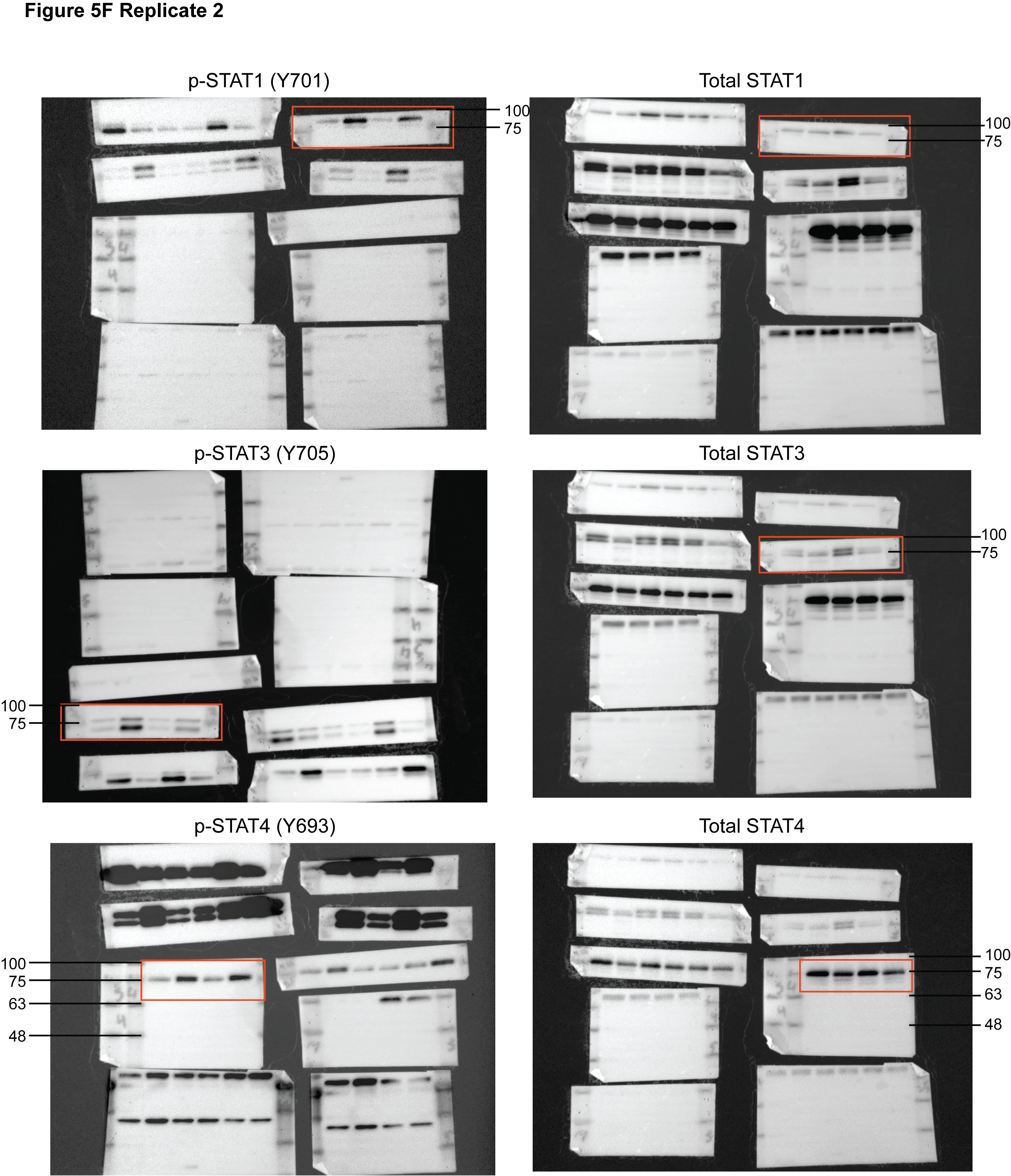

Supplement: Supplementary file 7 — Source data Fig. 5 [file 44319_2026_745_MOESM7_ESM.zip › Figure 5/5F/Repeats 5F/Molecular Weights/5F Replicate2_STAT1_STAT3_STAT4.tif]

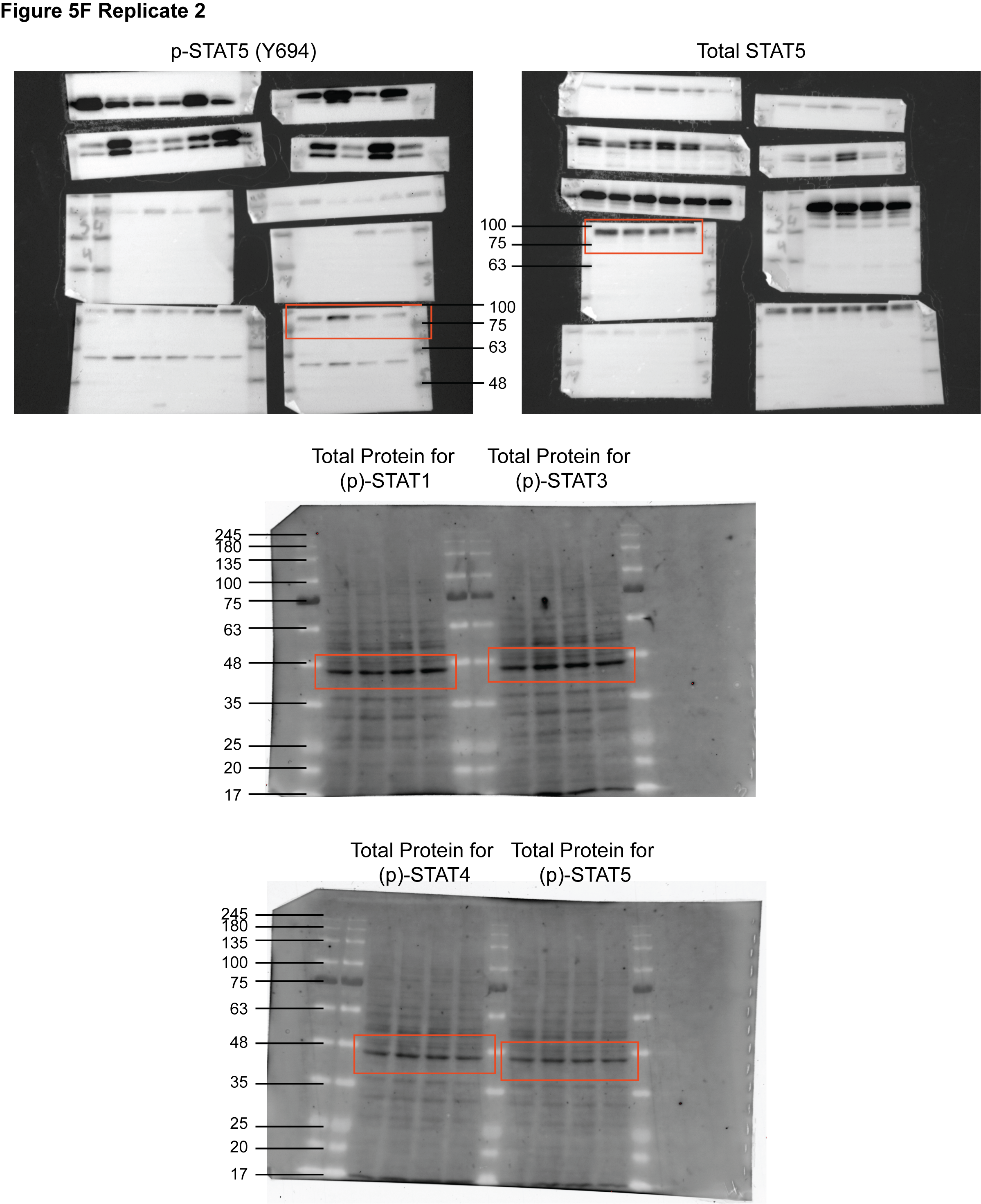

Supplement: Supplementary file 7 — Source data Fig. 5 [file 44319_2026_745_MOESM7_ESM.zip › Figure 5/5F/Repeats 5F/Molecular Weights/5F Replicate2_STAT5_Total Protein Loading Control.tif]

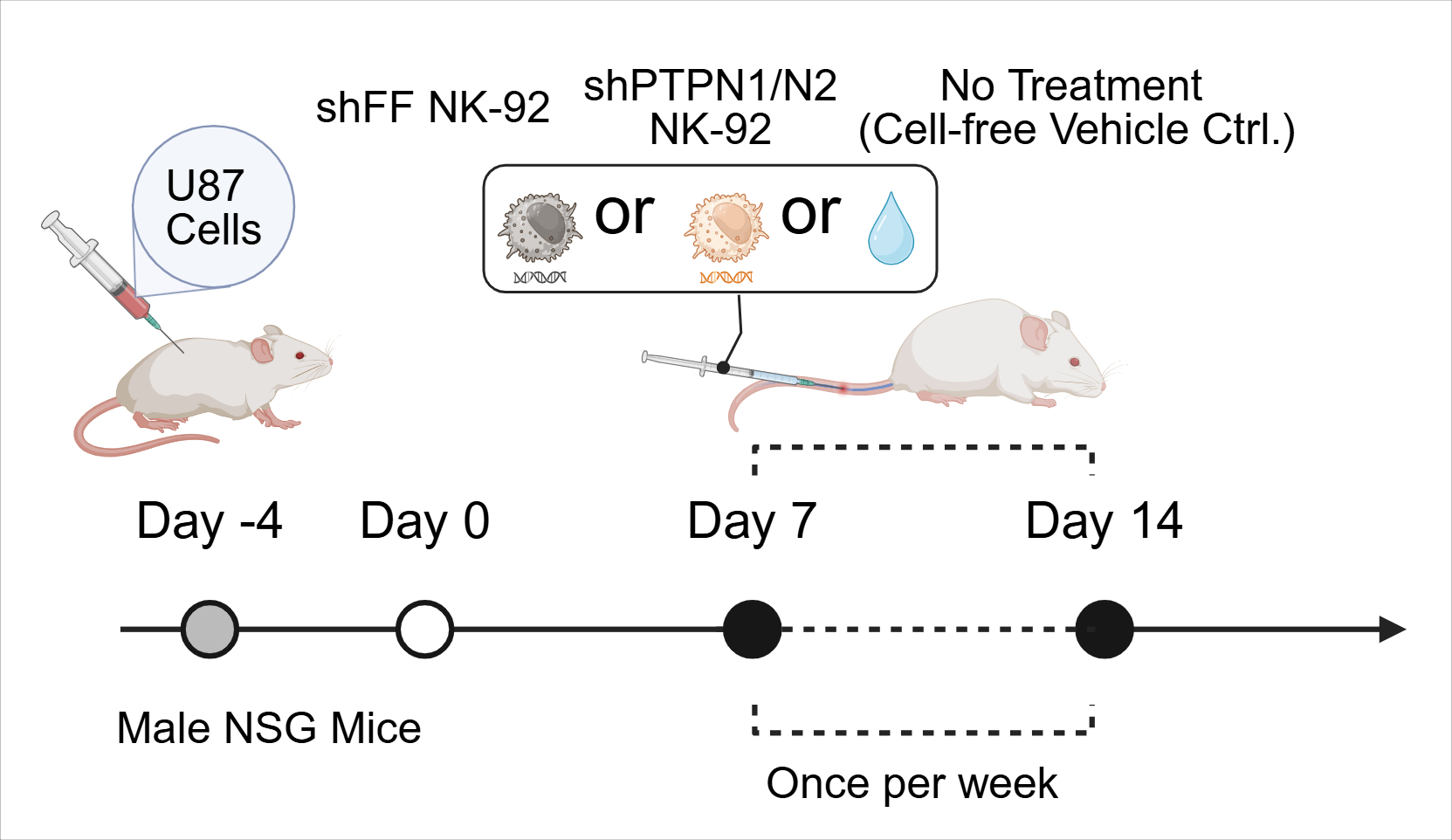

Supplement: Supplementary file 8 — Source data Fig. 6 [file 44319_2026_745_MOESM8_ESM.zip › Figure 6/6A/6A.jpeg]

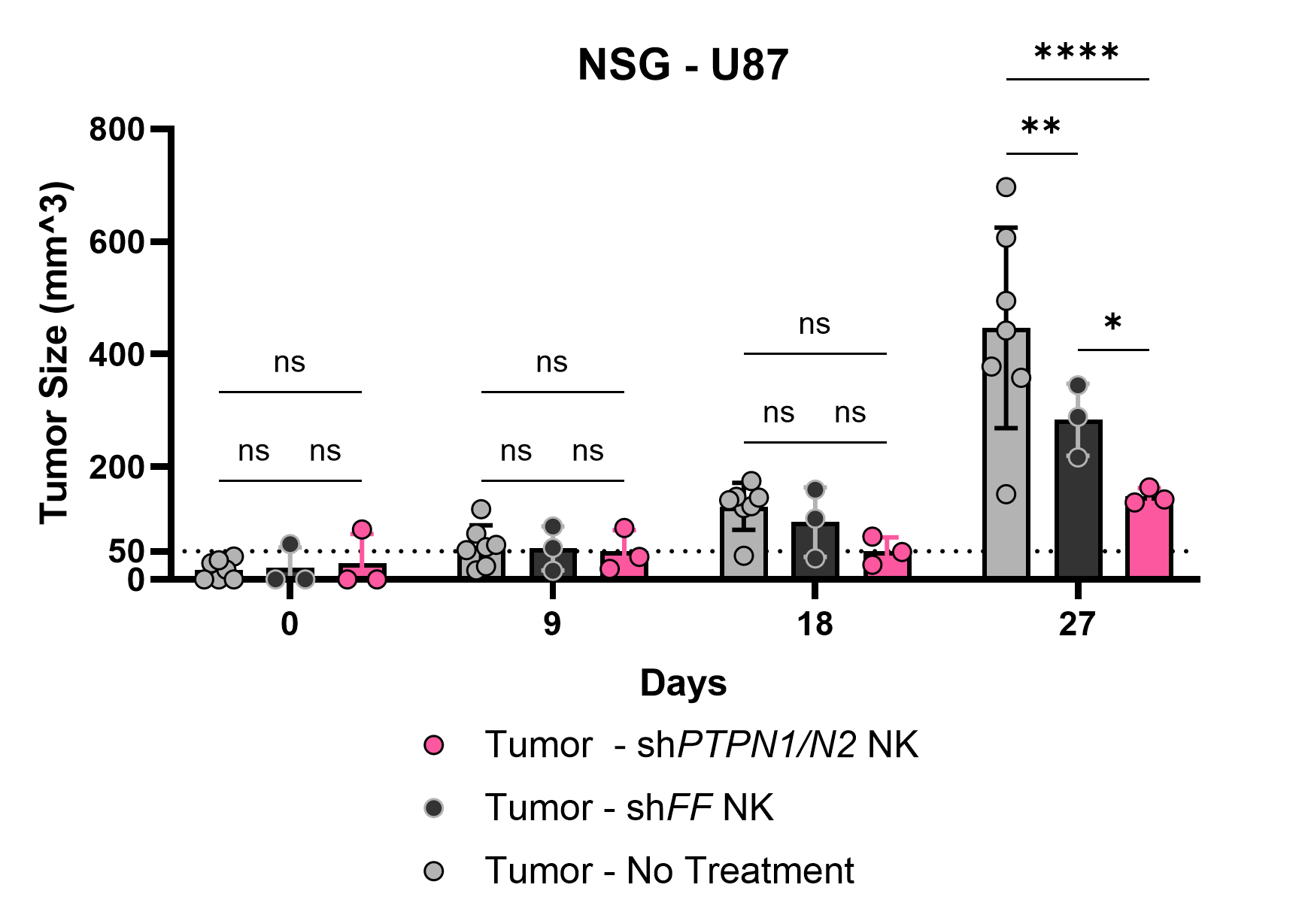

Supplement: Supplementary file 8 — Source data Fig. 6 [file 44319_2026_745_MOESM8_ESM.zip › Figure 6/6B/6B.tif]

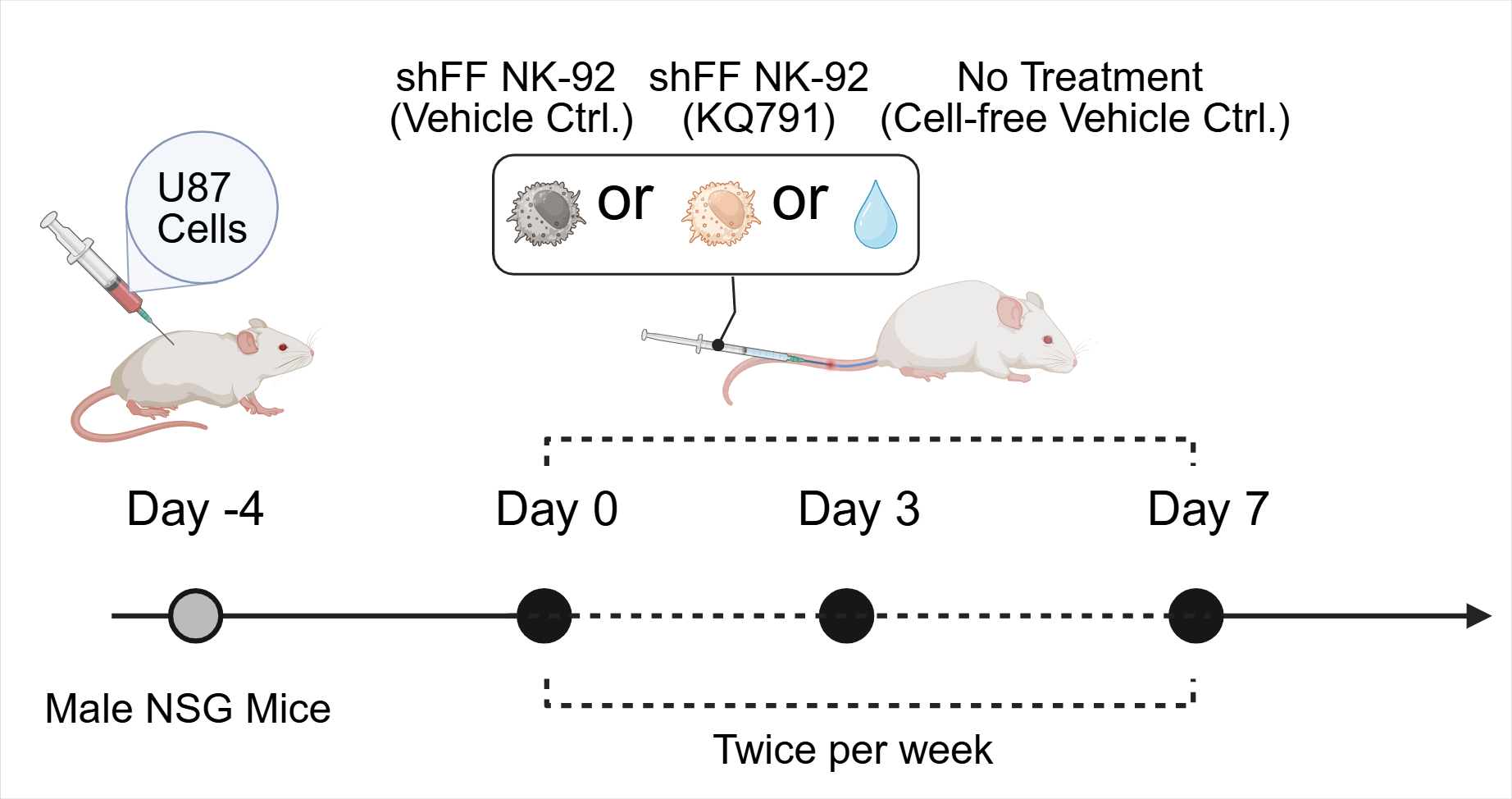

Supplement: Supplementary file 8 — Source data Fig. 6 [file 44319_2026_745_MOESM8_ESM.zip › Figure 6/6C/6C.jpeg]

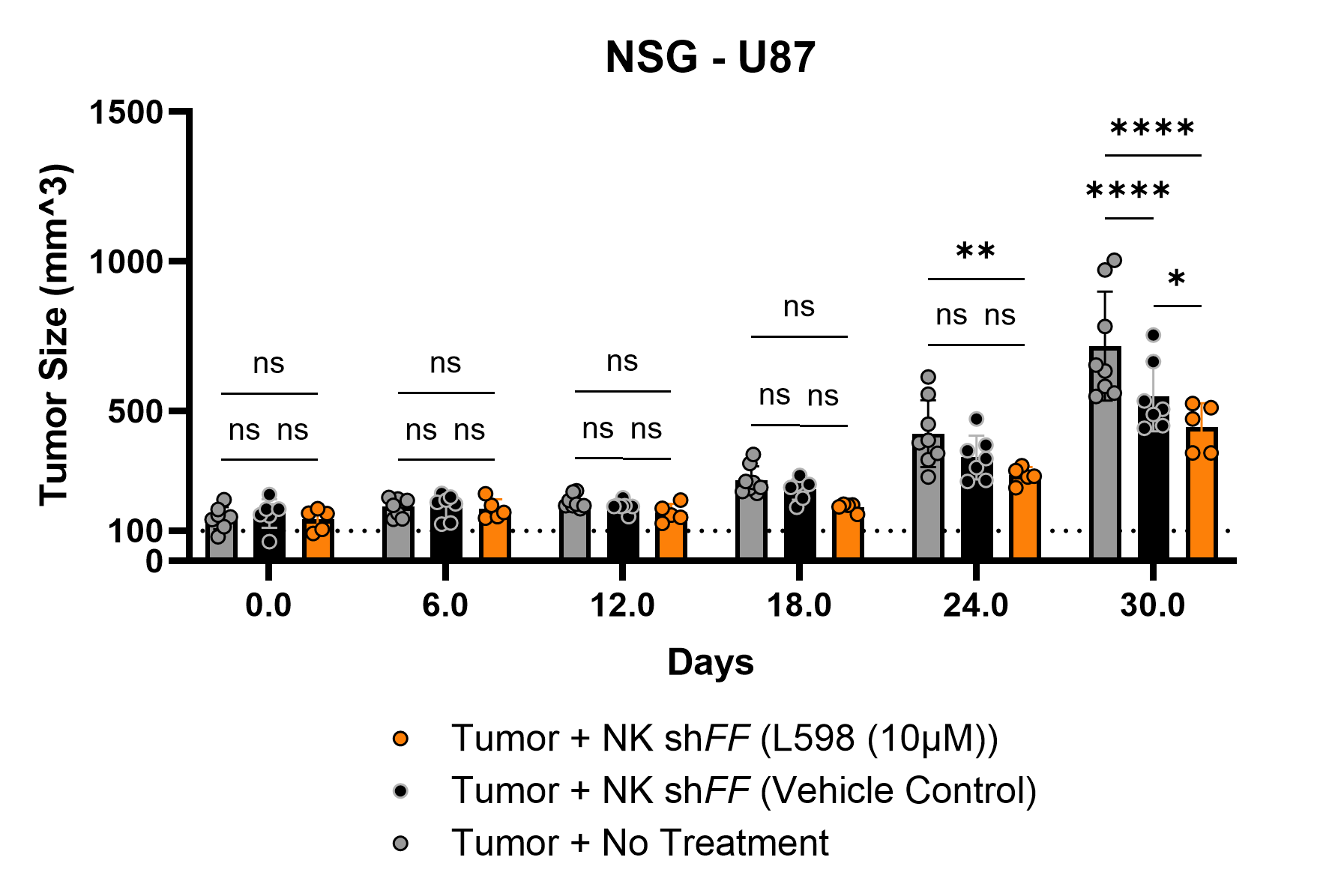

Supplement: Supplementary file 8 — Source data Fig. 6 [file 44319_2026_745_MOESM8_ESM.zip › Figure 6/6D/6D.tif]

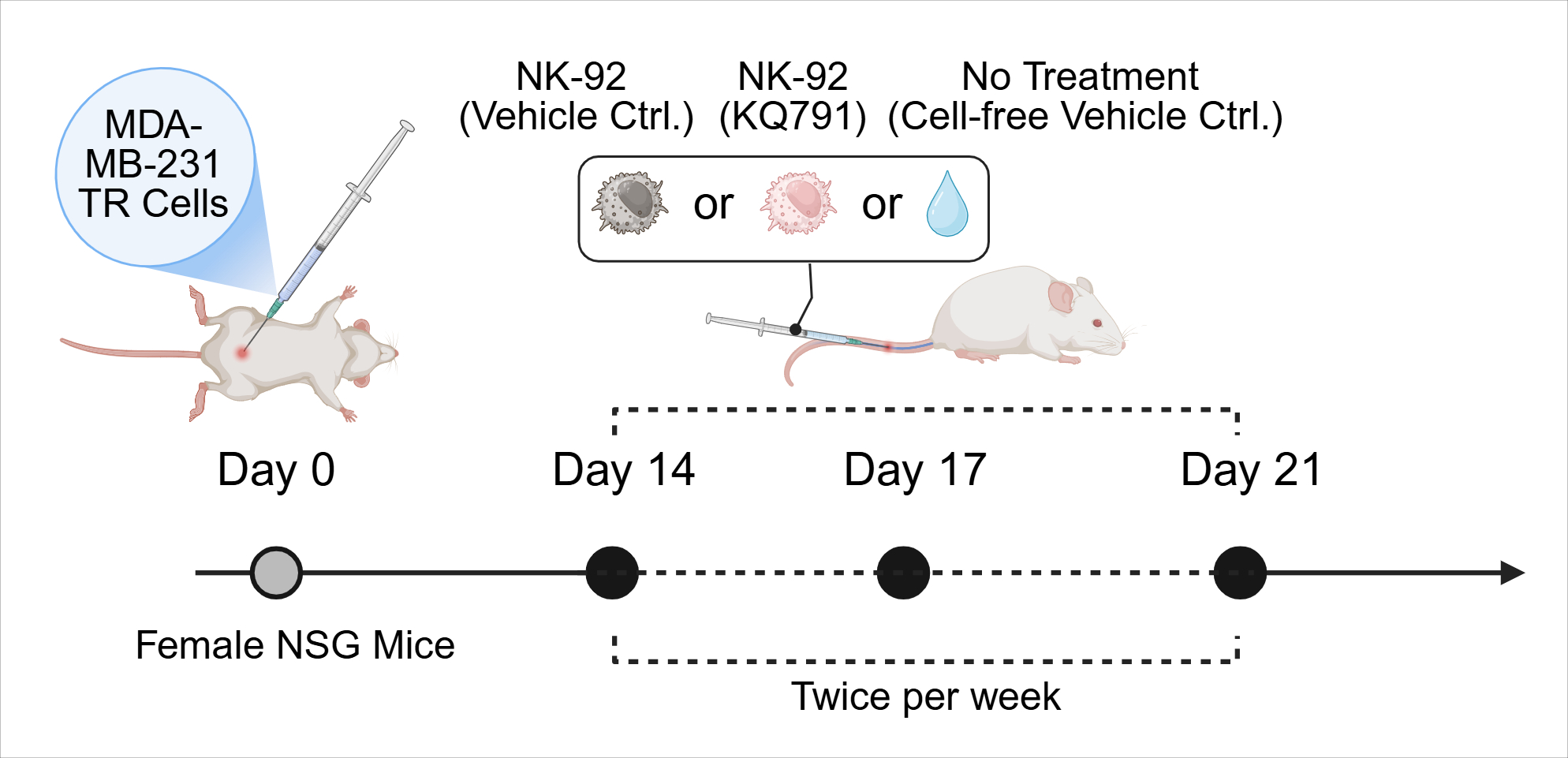

Supplement: Supplementary file 8 — Source data Fig. 6 [file 44319_2026_745_MOESM8_ESM.zip › Figure 6/6E/6E.jpeg]

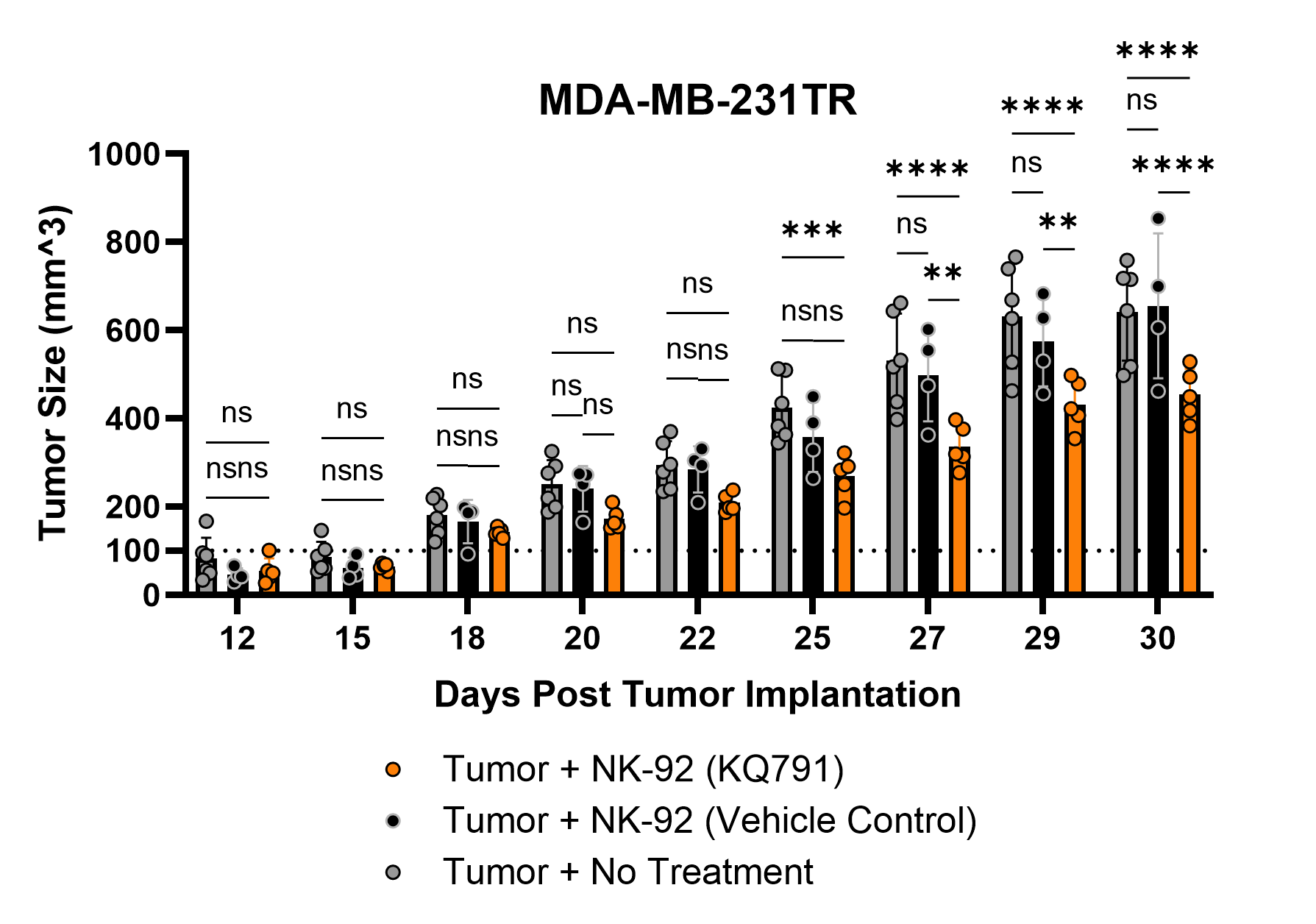

Supplement: Supplementary file 8 — Source data Fig. 6 [file 44319_2026_745_MOESM8_ESM.zip › Figure 6/6F/6F.tif]

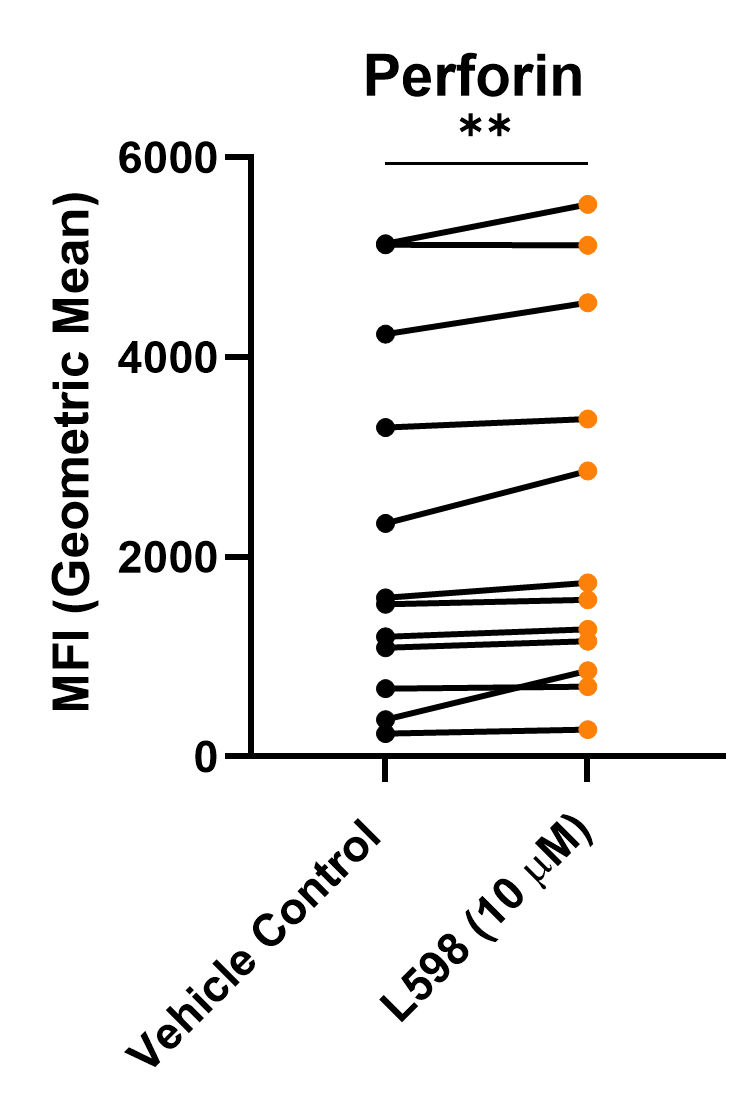

Supplement: Supplementary file 9 — Source data Fig. 7 [file 44319_2026_745_MOESM9_ESM.zip › Figure 7/7A/7A.tif]

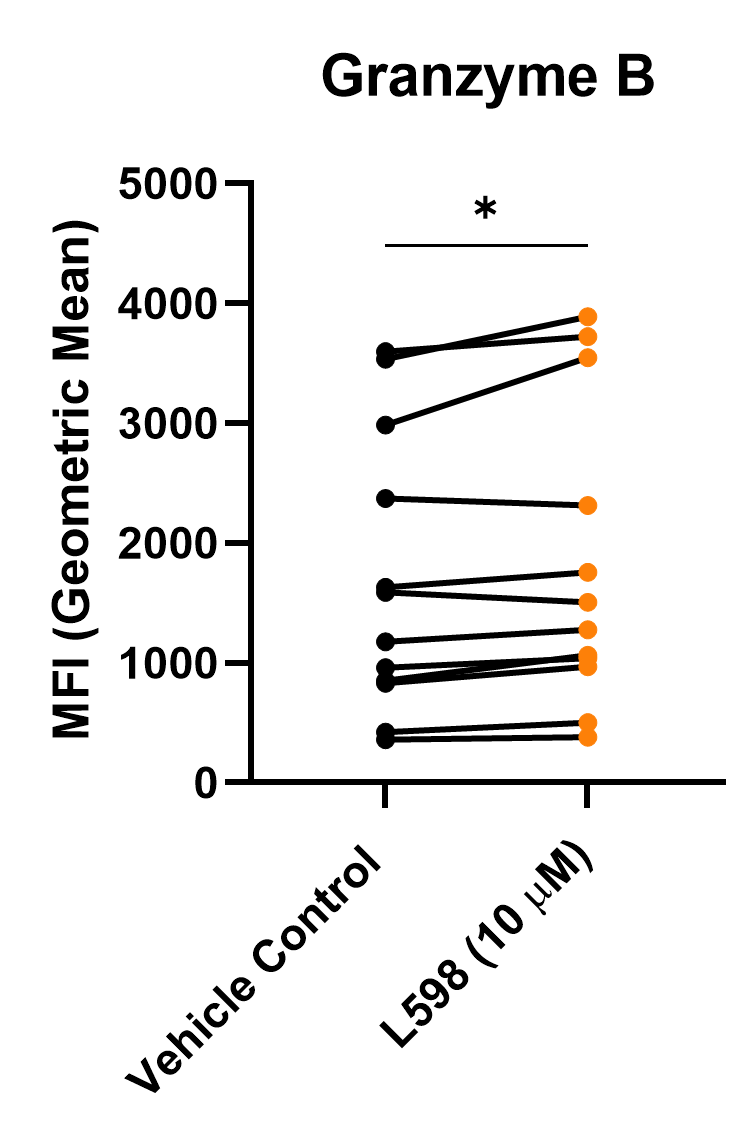

Supplement: Supplementary file 9 — Source data Fig. 7 [file 44319_2026_745_MOESM9_ESM.zip › Figure 7/7B/7B.tif]

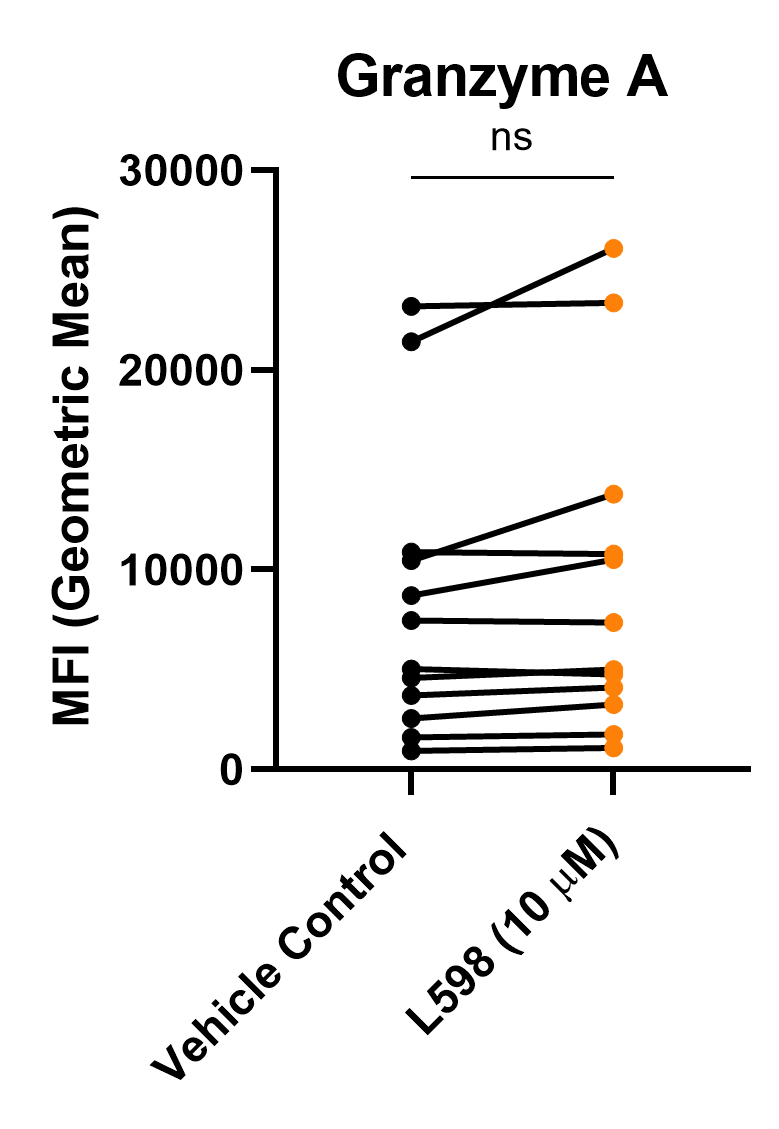

Supplement: Supplementary file 9 — Source data Fig. 7 [file 44319_2026_745_MOESM9_ESM.zip › Figure 7/7C/7C.tif]

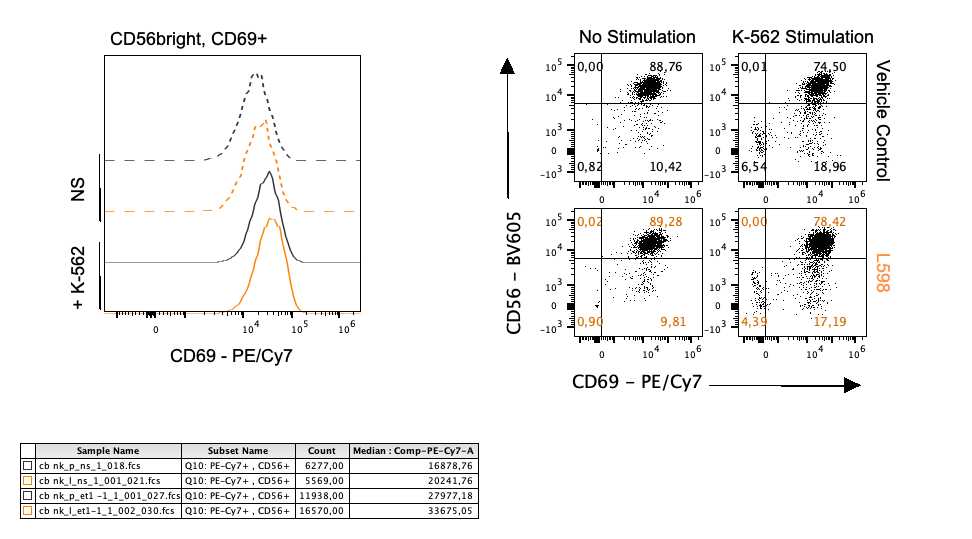

Supplement: Supplementary file 9 — Source data Fig. 7 [file 44319_2026_745_MOESM9_ESM.zip › Figure 7/7D/7D.tiff]

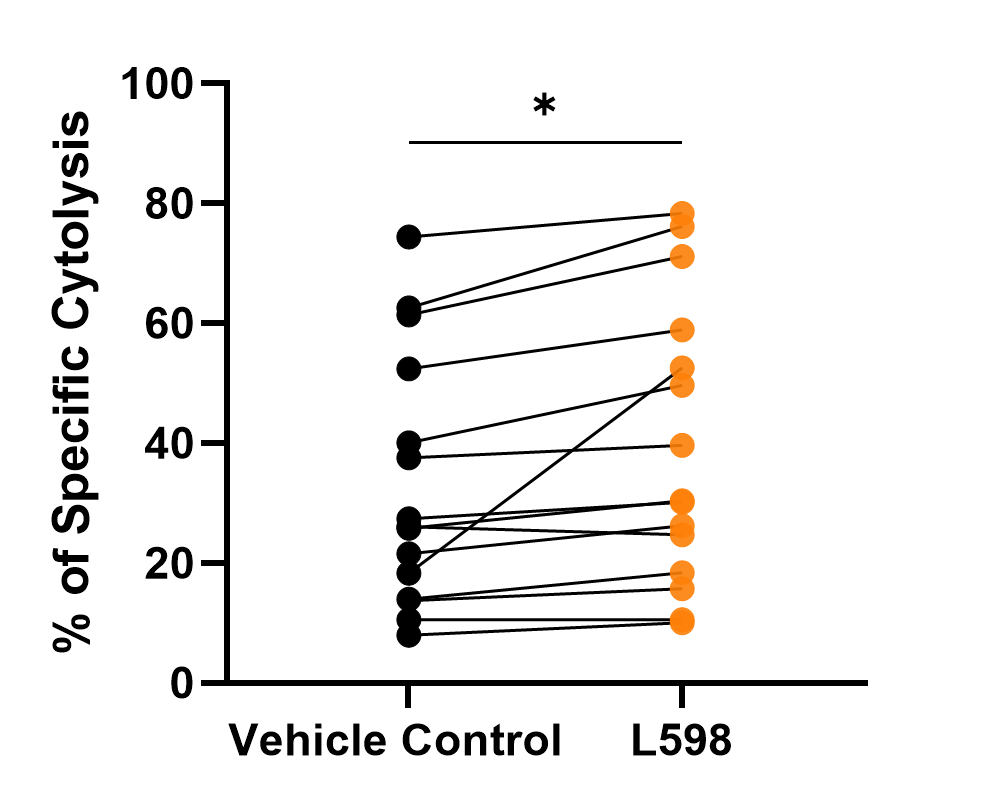

Supplement: Supplementary file 9 — Source data Fig. 7 [file 44319_2026_745_MOESM9_ESM.zip › Figure 7/7F/7F.tif]

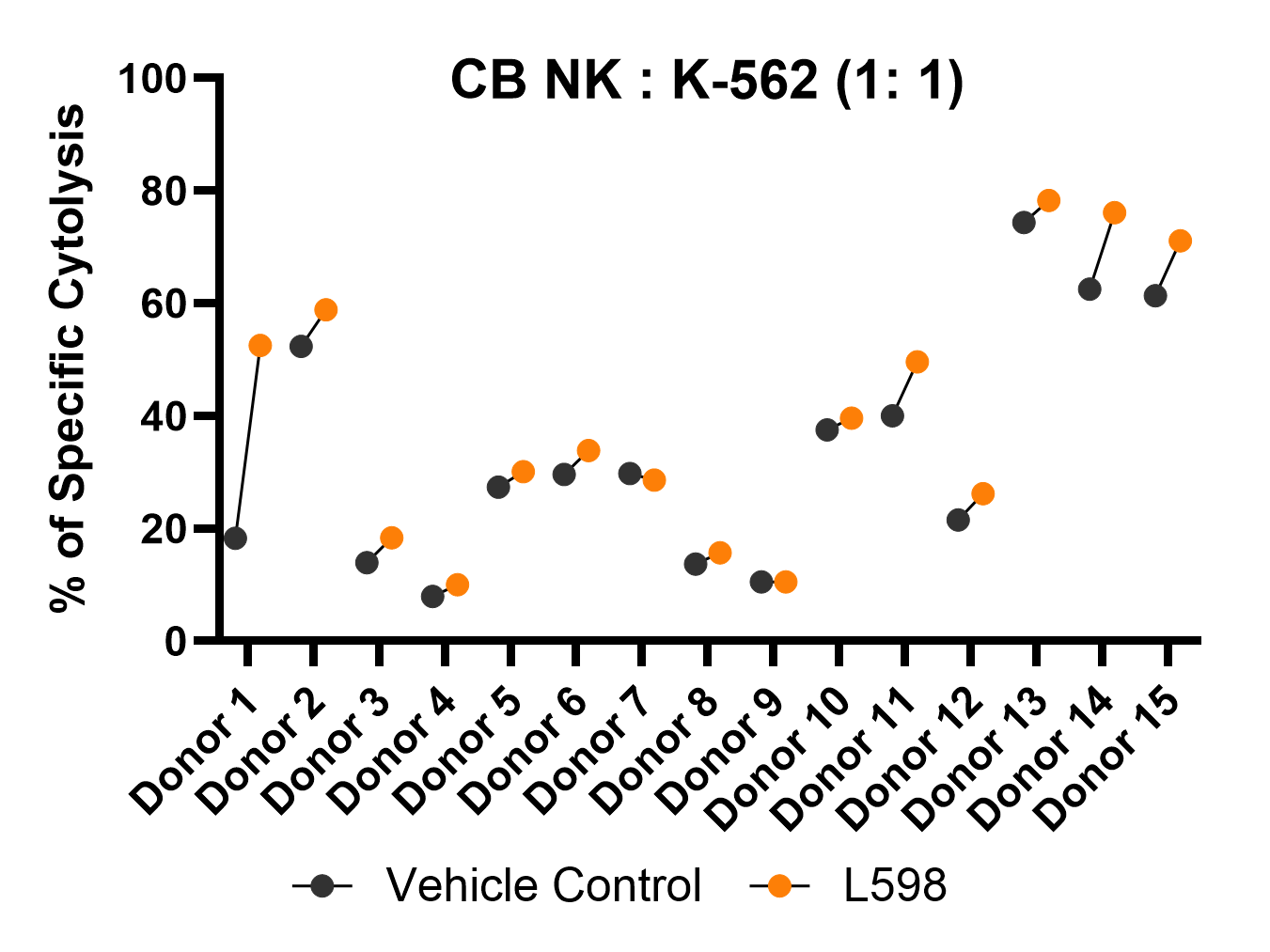

Supplement: Supplementary file 9 — Source data Fig. 7 [file 44319_2026_745_MOESM9_ESM.zip › Figure 7/7G/7G.tif]

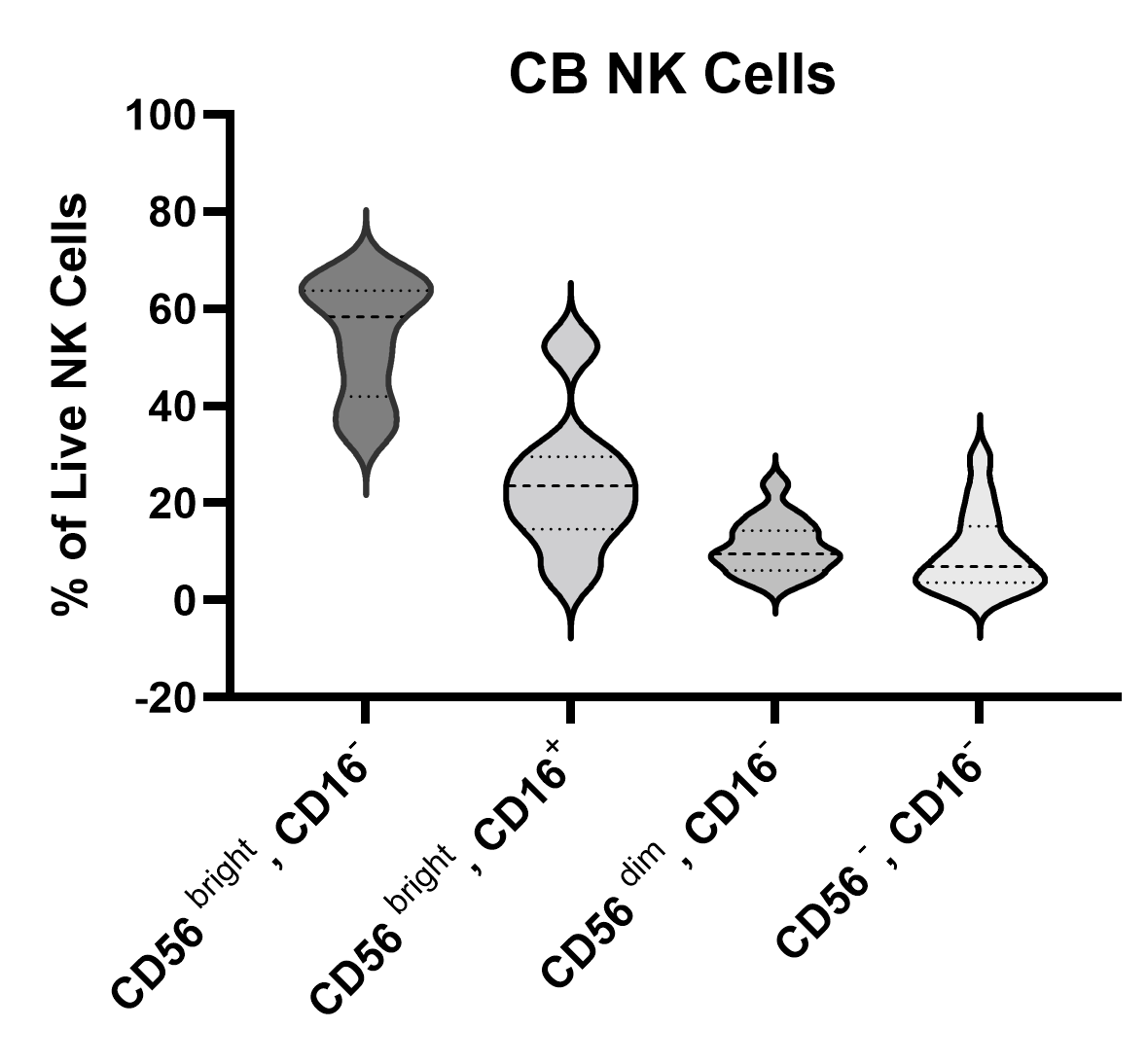

Supplement: Supplementary file 9 — Source data Fig. 7 [file 44319_2026_745_MOESM9_ESM.zip › Figure 7/7H/7H.tif]

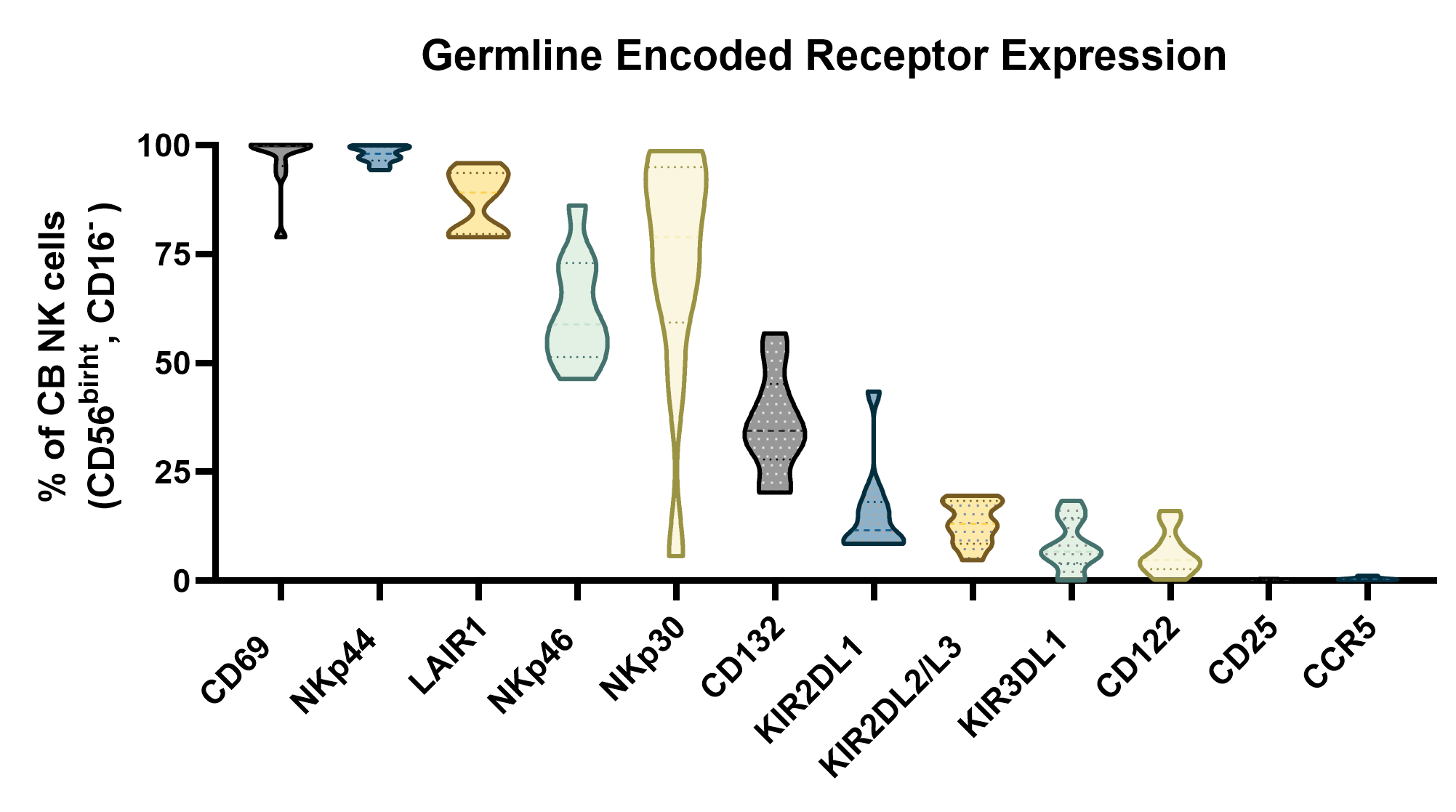

Supplement: Supplementary file 9 — Source data Fig. 7 [file 44319_2026_745_MOESM9_ESM.zip › Figure 7/7I/7I.tif]

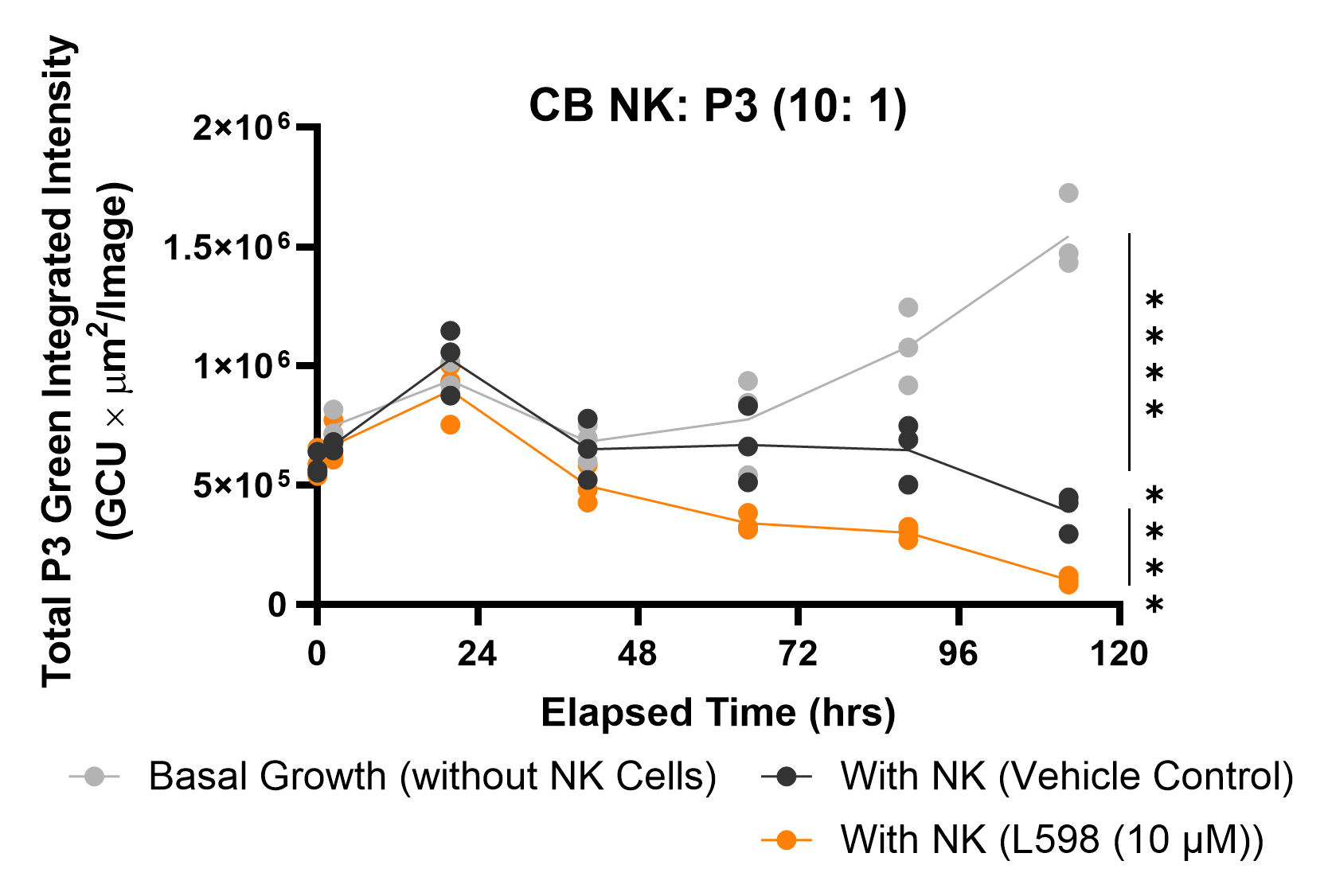

Supplement: Supplementary file 9 — Source data Fig. 7 [file 44319_2026_745_MOESM9_ESM.zip › Figure 7/7J/7J.tif]

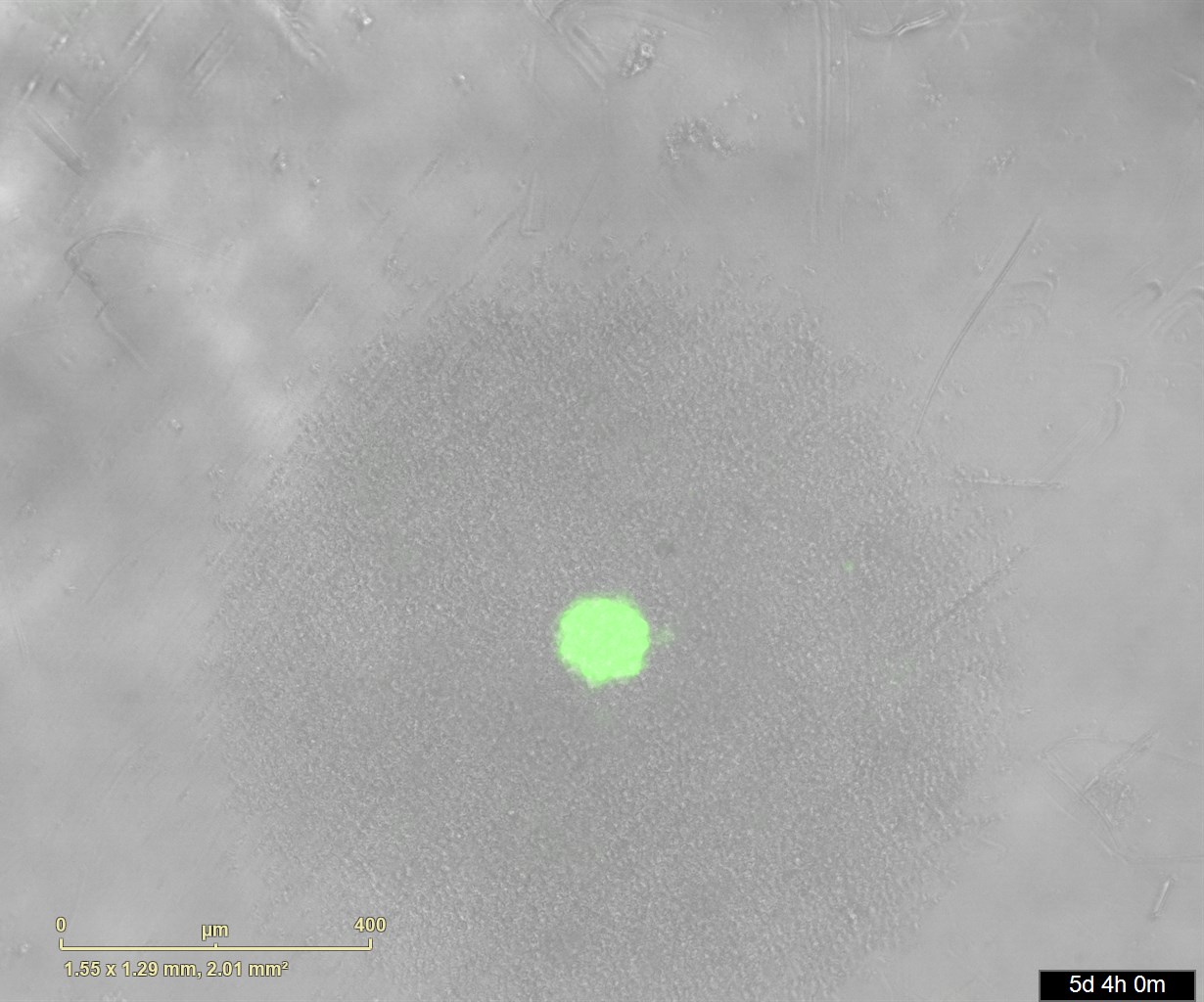

Supplement: Supplementary file 9 — Source data Fig. 7 [file 44319_2026_745_MOESM9_ESM.zip › Figure 7/7K/7K_L598_G4 well_05d04h00m.jpg]

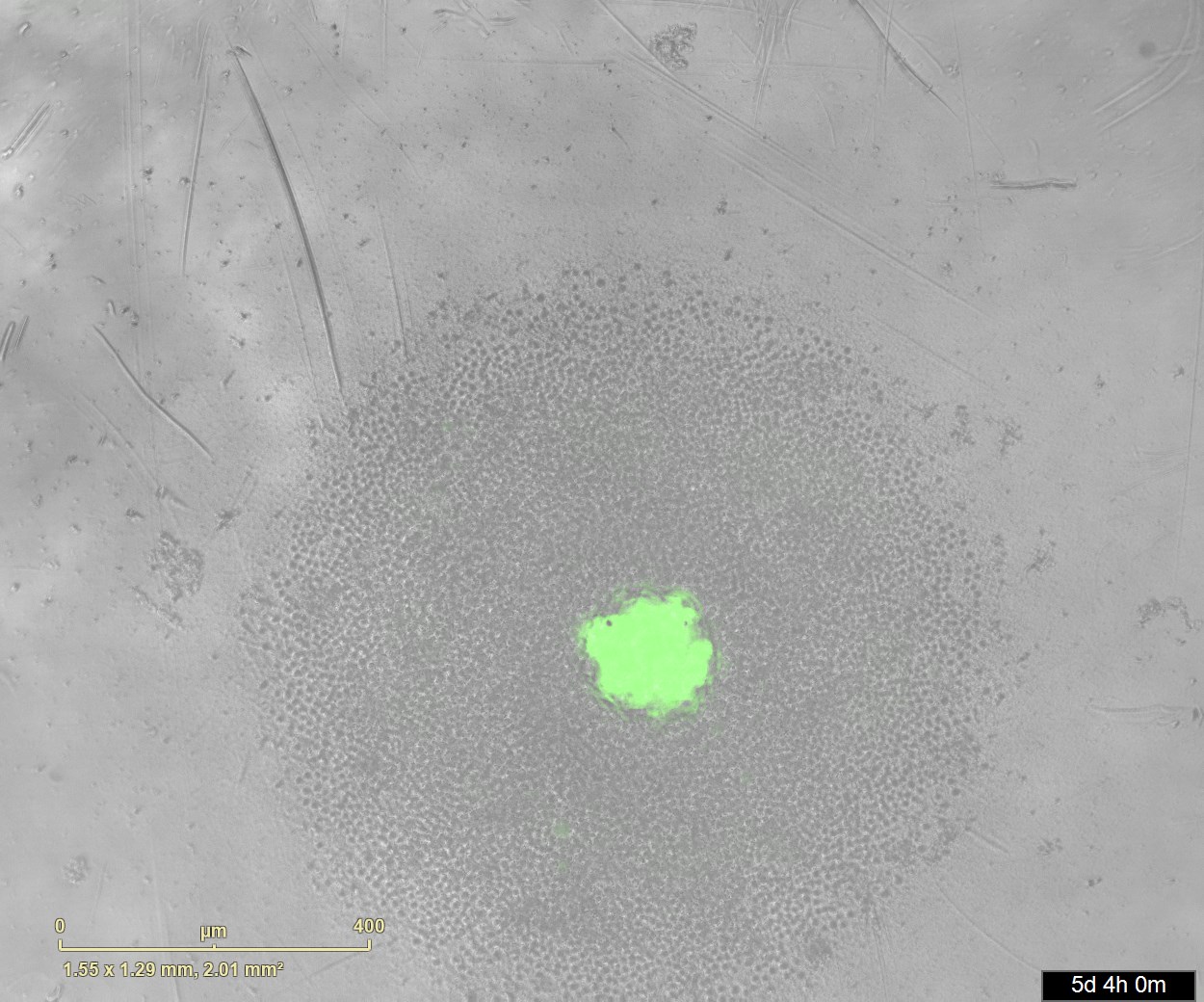

Supplement: Supplementary file 9 — Source data Fig. 7 [file 44319_2026_745_MOESM9_ESM.zip › Figure 7/7K/7K_PBS_D4 well_05d04h00m.jpg]

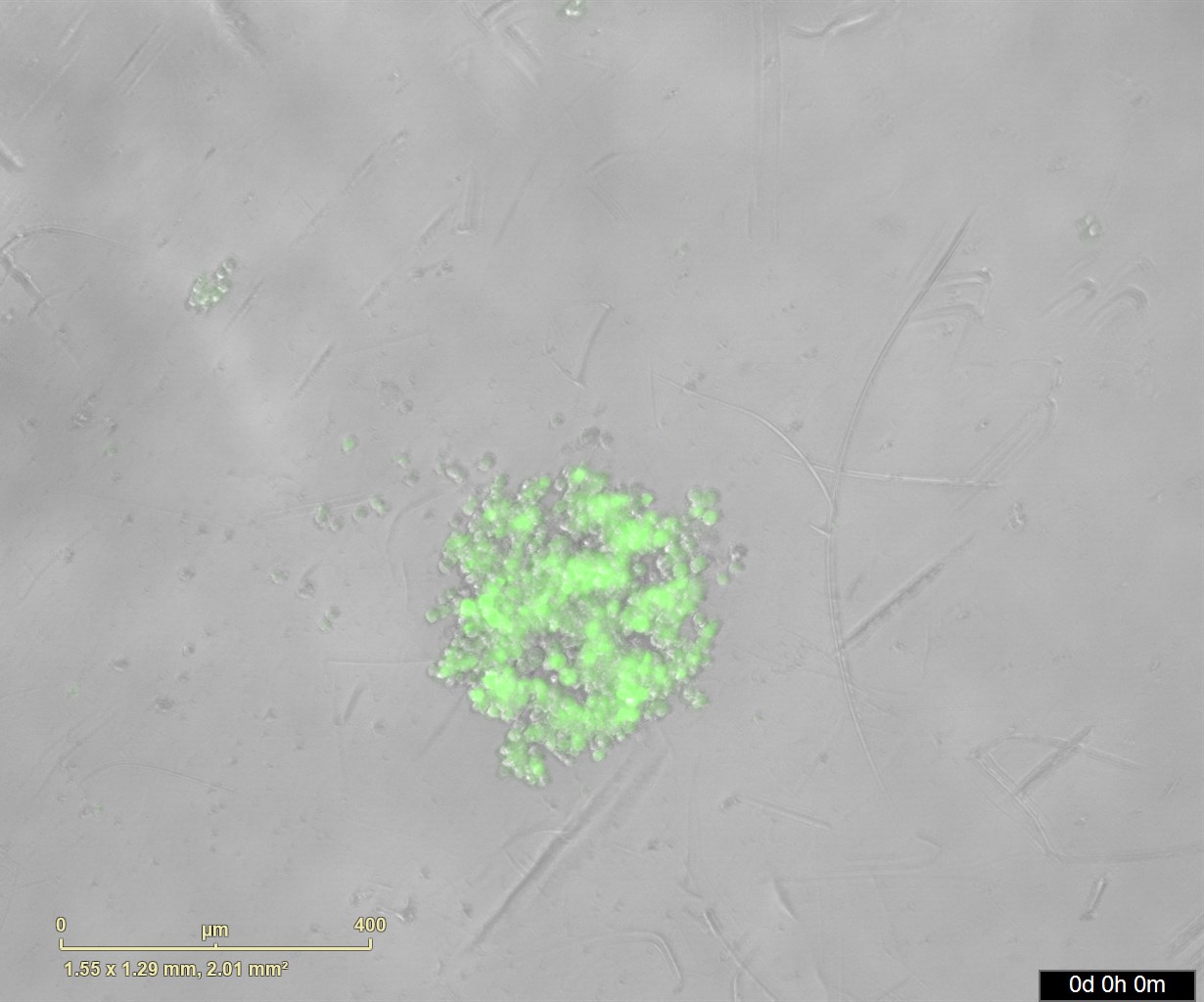

Supplement: Supplementary file 9 — Source data Fig. 7 [file 44319_2026_745_MOESM9_ESM.zip › Figure 7/7K/7K_L598_G4 well_00d00h00m.jpg]

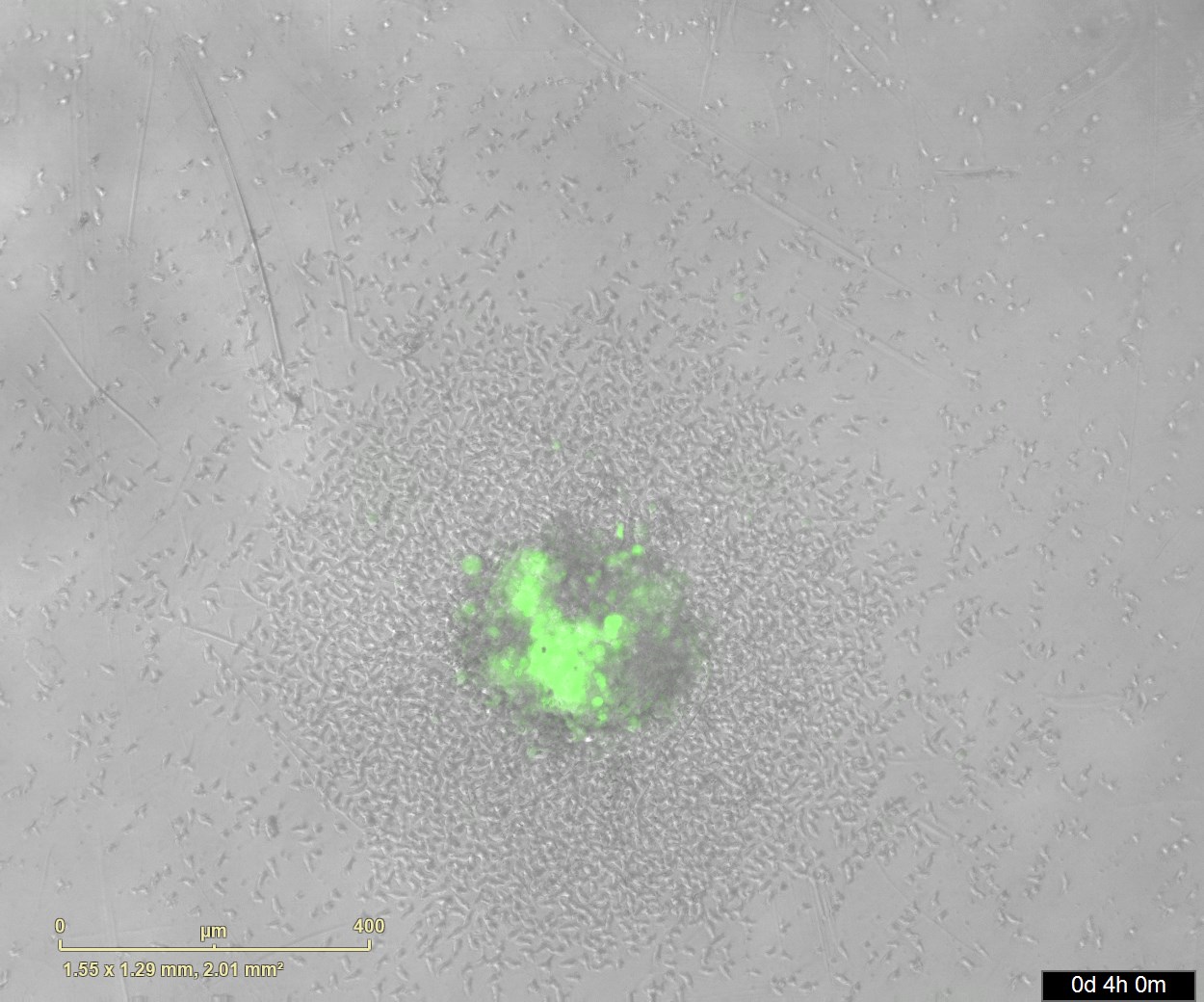

Supplement: Supplementary file 9 — Source data Fig. 7 [file 44319_2026_745_MOESM9_ESM.zip › Figure 7/7K/7K_PBS_D4 well_00d04h00m.jpg]

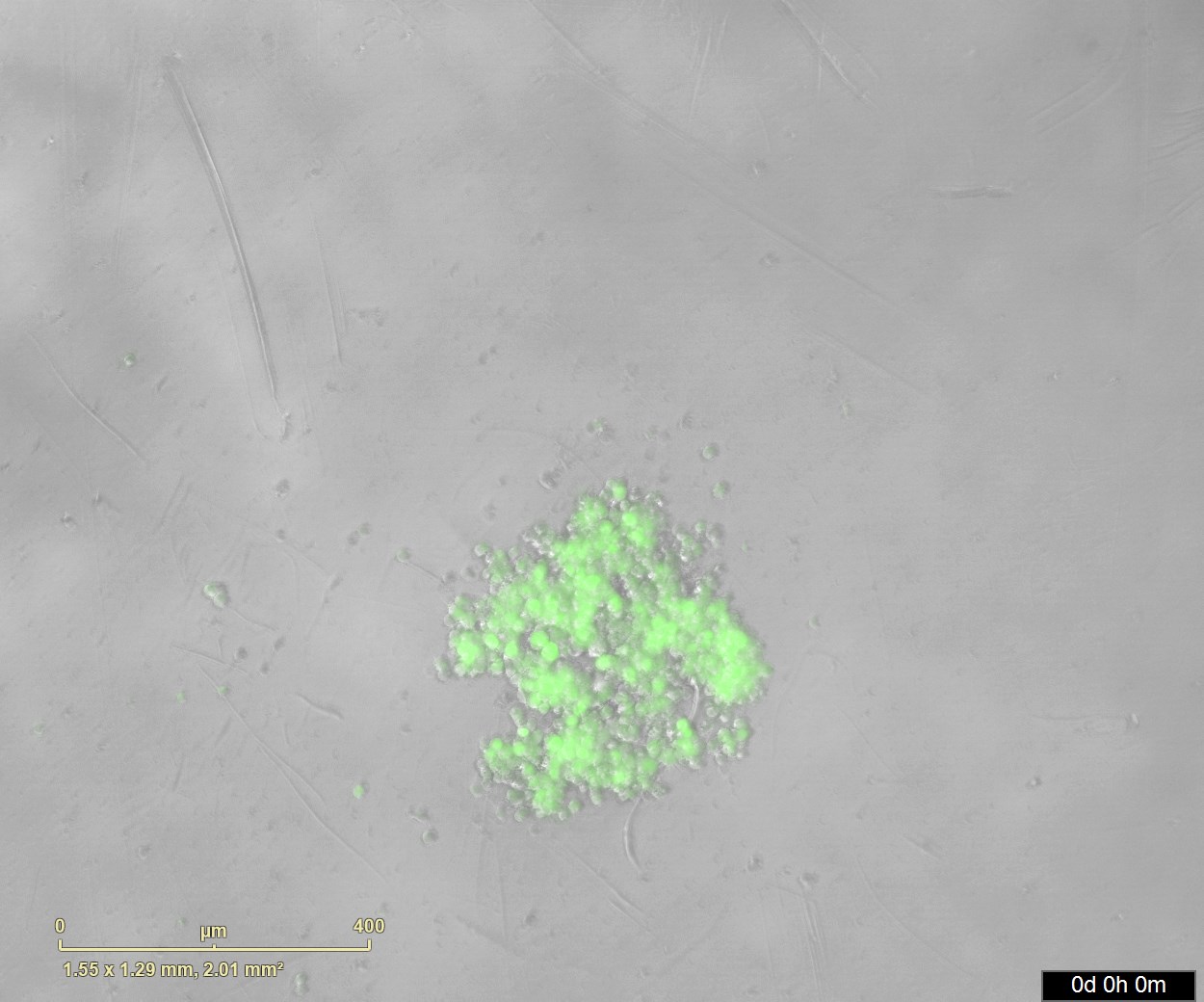

Supplement: Supplementary file 9 — Source data Fig. 7 [file 44319_2026_745_MOESM9_ESM.zip › Figure 7/7K/7K_PBS_D4 well_00d00h00m.jpg]

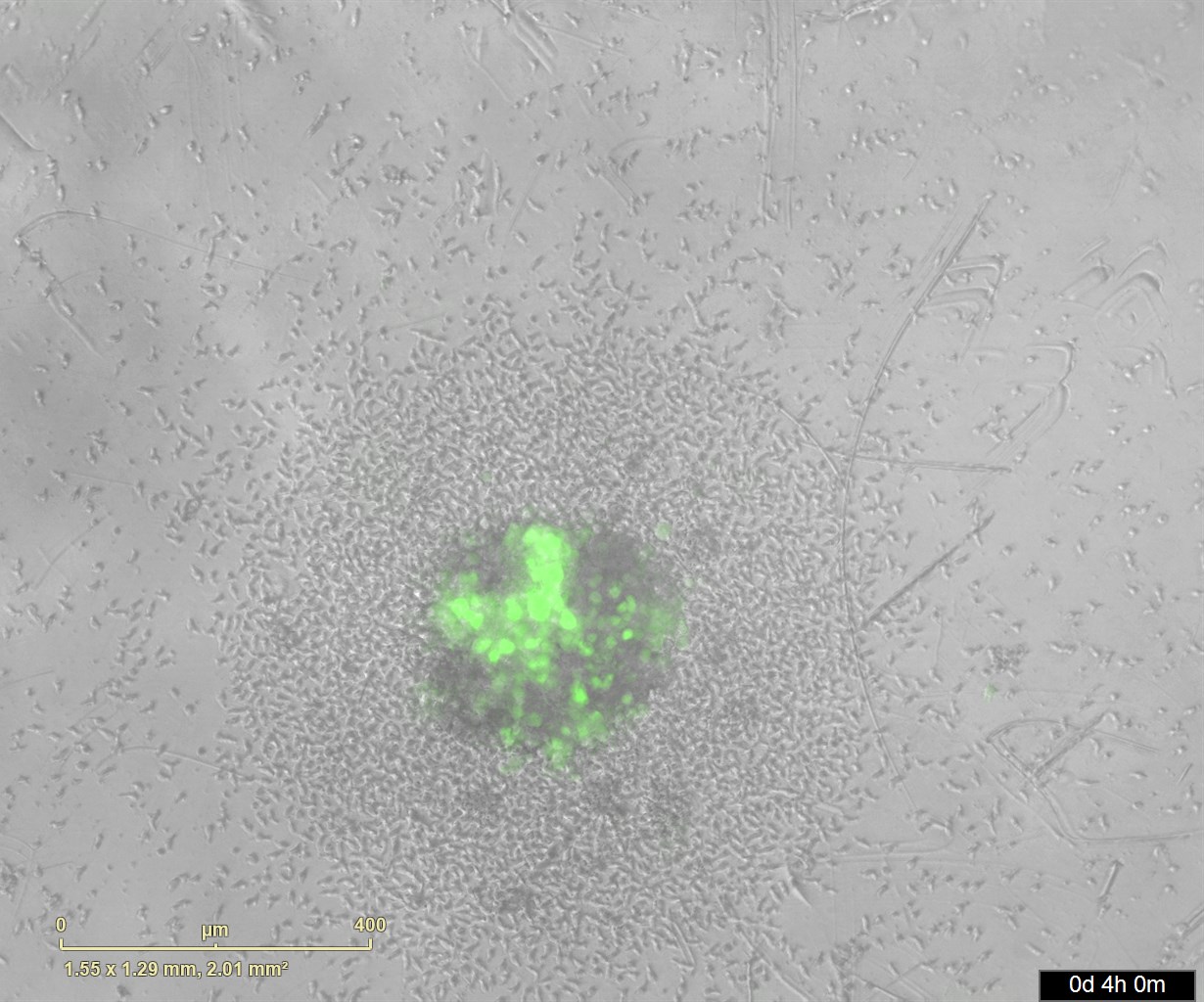

Supplement: Supplementary file 9 — Source data Fig. 7 [file 44319_2026_745_MOESM9_ESM.zip › Figure 7/7K/7K_L598_G4 well_00d04h00m.jpg]
