# Supplementary material for: PTPN1/PTPN2 inhibition improves NK cancer therapy by enhancing IL-2 and mitigating TGFβ1 responses
Source: EMBO Rep. 2026 Apr 15;27(10):2581–613. doi: 10.1038/s44319-026-00745-0 (PMC13219468; doi:10.1038/s44319-026-00745-0)
Supplement: Supplementary file 10 — Expanded View Figures [file 44319_2026_745_MOESM10_ESM.pdf]

Expanded View Figures

Figure EV1. Genetic knockdown of PTPN1 and PTPN2 promotes NK-92 cell activation.

(**A**, **B**) qRT-PCR analysis of PTPN1 and/or PTPN2 in shRNA-modified NK-92 cells. Results are from n = three biological replicates. Data are presented as mean ± SD. *P*-values (*PTPN1*) in (A): $p = 0.0025$ (**) for sh*PTPN1/PTPN2* vs sh*FF*, $p \leq 0.0001$ (****) for sh*PTPN1* vs sh*FF* (One-way ANOVA with Dunnett's test). *P*-values (*PTPN2*) in (**B**): $p = 0.0014$ (**) for sh*PTPN1/PTPN2* vs sh*FF*, $p \leq 0.0001$ (****) for sh*PTPN2* vs sh*FF* (One-way ANOVA with Dunnett's test). (**C**) Flow cytometry analysis of cell viability in sh*FF*, sh*PTPN1*, sh*PTPN2*, and *shPTPN1/PTPN2* cells in regular cell culture conditions. Results are from n = three biological replicates; each tested in three technical replicates). *P*-values: $p > 0.1234$ (ns) (One-way ANOVA with Tukey's test). (**D–F**) Cell images acquired in the bright phase using IncuCyte S3 were analyzed by ImageJ to quantify cell area for sh*FF* ($n = 707$ cells), sh*PTPN1* ($n = 700$ cells), sh*PTPN2* ($n = 594$ cells), and sh*PTPN1/PTPN2* ($n = 742$ cells) cells. Relative frequency in the percentage of cell area (bin size = 20) is presented in histography. *P*-values are indicated in the graph: $p < 0.0001$ (****) in (**D**); $p = 0.8277$ (ns) in (**E**); $p < 0.0001$ (****) in (**F**) (Unpaired t test). (**G–J**) Flow cytometry analyses of CD69 and CD25 expression on sh*FF* (control), sh*PTPN1*, sh*PTPN2* and sh*PTPN1*/sh*PTPN2* (dKD) cells following 5-h stimulation with tumor-target K562 (E:T = 1:2) or no stimulation. Geometric MFI and percentages of $CD69^+$ and $CD25^+$ live cells are shown in (**G–J**), with geoMFI in (**G**, **I**) and percentages in (**H**, **J**). Results are from n = three biological replicates; each tested in three technical replicates. *P*-values in (**G**): $p = 0.0002$ (***) for sh*FF* vs sh*PTPN1* at no stimulation, $p = 0.0004$ (***) for sh*FF* vs sh*PTPN2* at no stimulation, $p = 0.0024$ (**) for sh*FF* vs sh*PTPN1* at E:T (1:2), $p = 0.0022$ (**) for sh*FF* vs sh*PTPN2* at E:T (1:2), $p = 0.0001$ (***) for sh*FF* vs sh*PTPN1*/sh*PTPN2* at E:T (1:2), $p \leq 0.0001$ (****). *P*-values in (**H**): $p = 0.0001$ (***) for sh*FF* vs sh*PTPN2* at no stimulation, $p = 0.0626$ (ns), $p \leq 0.0001$ (****). *P*-values in (**I**): $p = 0.0434$ (*) for sh*FF* vs sh*PTPN1* at no stimulation, $p = 0.9986$ (ns) for sh*FF* vs sh*PTPN2* at no stimulation, $p = 0.0001$ (***) for sh*FF* vs sh*PTPN1* at E:T (1:2), $p = 0.9653$ (ns) for sh*FF* vs sh*PTPN2* at E:T (1:2), $p \leq 0.0001$ (****). P-values in (**J**): $p = 0.3513$ (ns) for sh*FF* vs sh*PTPN1* at no stimulation, $p = 0.5773$ (ns) for sh*FF* vs sh*PTPN2* at no stimulation, $p = 0.0004$ (***) for sh*PTPN1* vs sh*PTPN1*/sh*PTPN2* at no stimulation, $p = 0.0002$ (***) for sh*FF* vs sh*PTPN1* at E:T (1:2), $p = 0.9456$ (ns) for sh*FF* vs sh*PTPN2* at E:T (1:2), $p \leq 0.0001$ (****). (Two-way ANOVA with Šídák's multiple comparisons test).

© The Author(s)

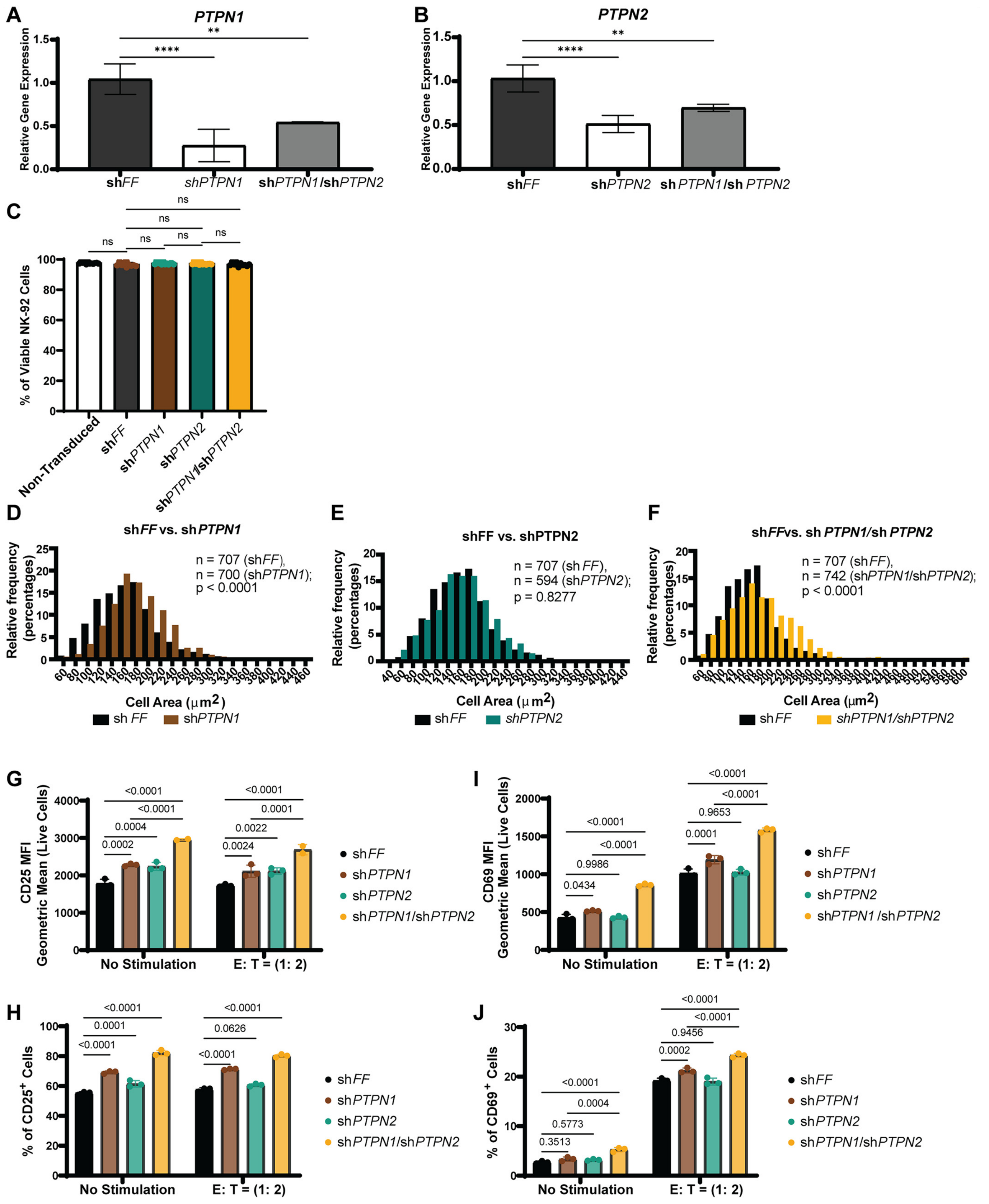
A
PTPN1
Relative Gene Expression
shFF
shPTPN1
shPTPN1/shPTPN2
B
PTPN2
shPTPN2
C
% of Viable NK-92 Cells
Non-Transduced
D
shFF vs. shPTPN1
n = 707 (shFF), n = 700 (shPTPN1); p < 0.0001
Relative frequency (percentages)
Cell Area (μm2)
E
shFF vs. shPTPN2
n = 707 (shFF), n = 594 (shPTPN2); p = 0.8277
F
shFFvs. shPTPN1/shPTPN2
n = 707 (shFF), n = 742 (shPTPN1/shPTPN2); p < 0.0001
G
CD25 MFI Geometric Mean (Live Cells)
No Stimulation
E: T = (1: 2)
I
CD69 MFI Geometric Mean (Live Cells)
H
% of CD25+ Cells
J
% of CD69+ Cells

 © The Author(s)

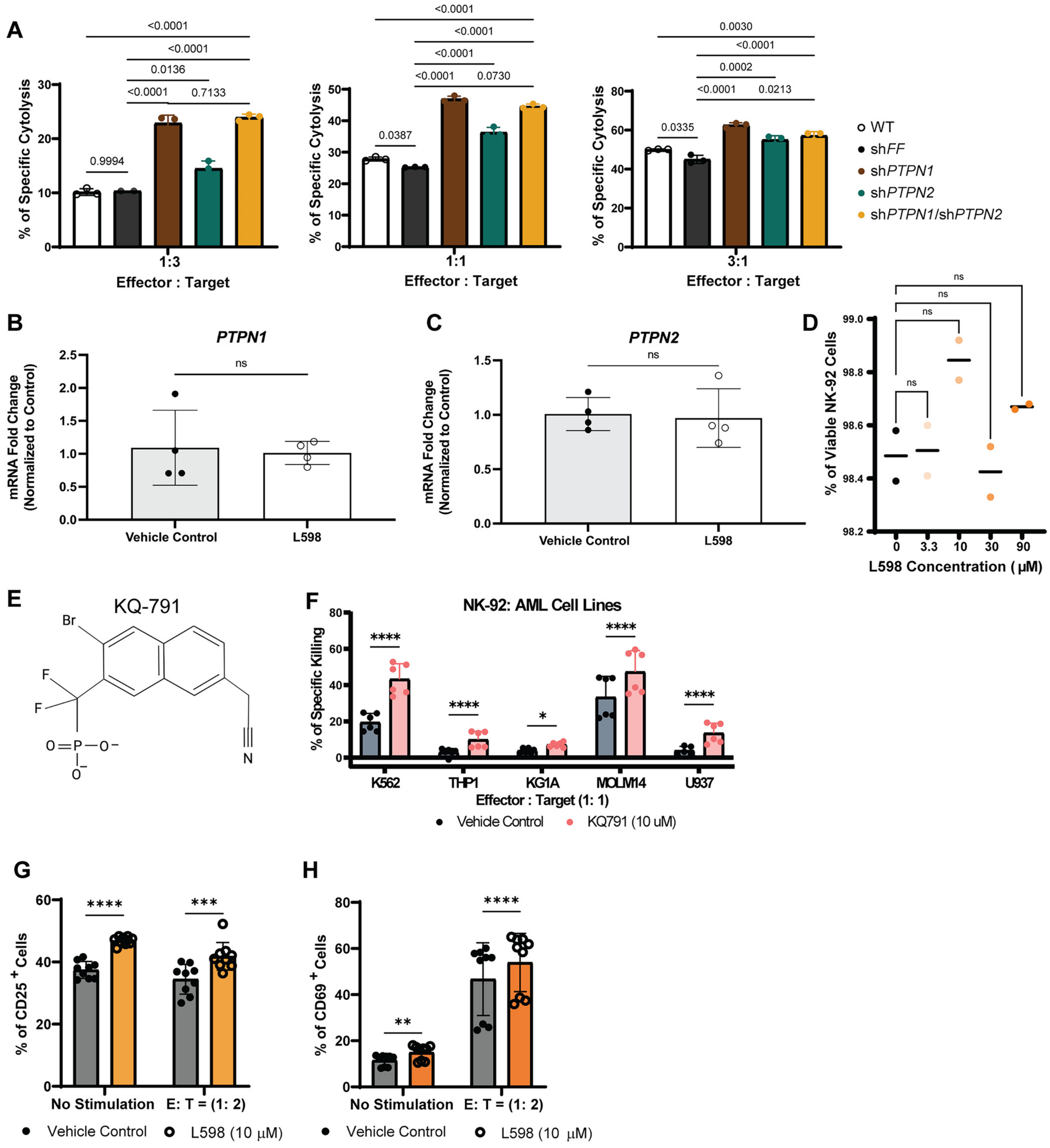
A
% of Specific Cytolysis
1:3
Effector : Target
1:1
Effector : Target
3:1
Effector : Target
WT
shFF
shPTPN1
shPTPN2
shPTPN1/shPTPN2
B
PTPN1
mRNA Fold Change (Normalized to Control)
Vehicle Control
L598
C
PTPN2
D
% of Viable NK-92 Cells
L598 Concentration (μM)
E
KQ-791
F
NK-92: AML Cell Lines
% of Specific Killing
K562
THP1
KG1A
MOLM14
U937
Effector : Target (1: 1)
Vehicle Control
KQ791 (10 uM)
G
% of CD25 + Cells
No Stimulation
E: T = (1: 2)
Vehicle Control
L598 (10 μM)
H
% of CD69 + Cells

Figure EV2. Dual targeting PTPN1 and/or PTPN2 enhances NK cell anti-tumor cytolysis against NK sensitive tumor targets.

(**A**) Statistical analysis of flow cytometry-based anti-tumor cytolysis assay (Fig. 2A) using shRNA knockdown NK-92 cells co-cultured with K-562 tumor targets at effector:target (E:T) ratios of 1:3 (left), 1:1 (middle), and 3:1 (right). A representative figure of $n =$ two biological replicates at 1:3 (E:T) ratio and three biological replicates at 1:1 and 3:1 (E:T) ratios, each tested in three technical replicates. Data are presented as mean ± SD. *P*-values (E:T = 1:3): $p = 0.9994$ (ns) for WT vs sh*FF*, $p = 0.7133$ (ns) for sh*PTPN1* vs sh*PTPN1*/sh*PTPN2*, $p = 0.0136$ (*) for sh*FF* vs sh*PTPN2*, $p \le 0.0001$ (****). *P*-values (E:T = 1:1): $p = 0.0387$ (*) for WT vs sh*FF*, $p = 0.073$ (ns) for sh*PTPN1* vs sh*PTPN1*/sh*PTPN2*, $p \le 0.0001$ (****). *P*-values (E:T = 3:1): $p = 0.0335$ (*) for WT vs sh*FF*, $p = 0.0213$ (*) for sh*PTPN1* vs sh*PTPN1*/sh*PTPN2*, $p = 0.003$ (**) for WT vs sh*PTPN1*/sh*PTPN2*, $p = 0.0002$ (***) for sh*FF* vs sh*PTPN2*, $p \le 0.0001$ (****). (One-way ANOVA with Tukey's multiple comparison test). (**B**, **C**) qRT-PCR analysis of *PTPN1* (**B**) and *PTPN2* (**C**) expression in NK-92 cells treated for 3 days with L598 (30 μM) or vehicle control. Data are presented as mean ± SD. *P*-values: $p = 0.7975$ (ns) for *PTPN1*; $p = 0.8186$ (ns) for *PTPN2* (Unpaired t-test). A representative figure of $n =$ two independent experiments, each tested in four technical replicates is shown. (**D**) Flow cytometry analysis of NK-92 cell viability following increasing doses of L598 treatment. Results are from two biological replicates; each tested in two or three technical replicates. *P*-values: $p > 0.1234$ (ns) (One-way ANOVA with Dunnett's test). (**E**) A structural representation of KQ791, a dual inhibitor of PTPN1/PTPN2. (**F**) Flow cytometry-based cytolysis assay of KQ791 (10 μM)-treated or vehicle control-treated NK-92 cells, co-cultured with various acute myeloid leukemia (AML) cell lines. Results are from $n =$ two biological replicates, each tested in three technical replicates. Data are presented as mean ± SD. *P*-values: $p = 0.0127$ (*) for KG1A cytolysis; $p \le 0.0001$ (****) (Two-way ANOVA with mixed effects and Šídák's multiple comparisons test). (**G**, **H**) Quantifications of the percentage of $CD25^+$ cells and $CD69^+$ NK-92 cells, following treatment with L598 (10 μM) or vehicle control, with or without K562 tumor cell stimulation (E:T = 1:2). Pooled results from $n =$ three biological replicates, each tested in three technical replicates. Data are presented as mean ± SD. *P*-values (CD25): $p = 0.0003$ (***) at E:T (1:2), $p \le 0.0001$ (****) at no stimulation. *P*-values (CD69): $p = 0.0025$ (**) at no stimulation, $p \le 0.0001$ (****) at E:T (1:2). (Two-way ANOVA with Šídák's multiple comparisons test).

 © The Author(s)

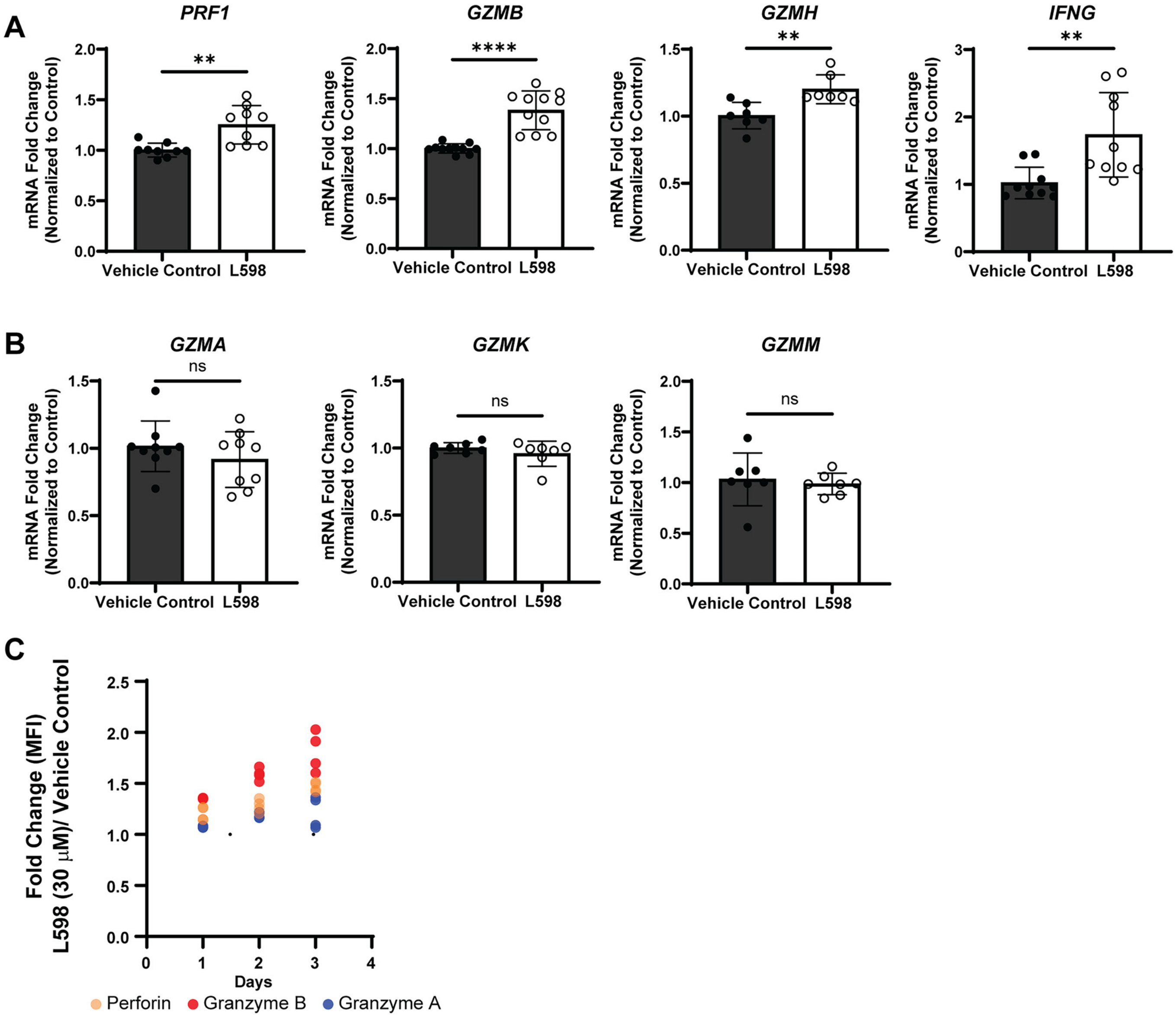

Figure EV3. Transcriptional expression of human granzymes (A, B, H, K, M), perforin, and interferon-γ in NK-92 cells with dual PTPN1/PTPN2 inhibition.

(**A**) mRNA expression of cytolytic effector molecules: perforin (*PRF1*), granzyme B (*GZMB*), granzyme H (*GZMH*) and IFN-γ (*IFNG*) in NK-92 cells after 3-days treatment of L598 (30 μM) or vehicle control. Results are from n = three biological replicates for *PRF1*, *GZMB* and *IFNG*, and from n = two biological replicates for *GZMH*, each tested in three or four technical replicates. Fold change was calculated against vehicle control of the Treatment group. Data are presented as mean ± SD. *P*-values: $p = 0.004$ (**) for *PRF1* and *GZMH*, $p = 0.0057$ (**) for *IFNG*, $p \le 0.0001$ (****) for *GZMB* (Unpaired t test with Welch's correction). (**B**) mRNA expression of granzyme A (*GZMA*), granzyme K (*GZMK*), and granzyme M (*GZMM*) did not significantly change after L598 (30 μM) treatment for 3 days compared with the vehicle control group in NK-92 cells. Results are from n = two biological replicates; each tested in three or four technical replicates. Data are presented as mean ± SD. *P*-values: $p = 0.3048$ (ns) for *GZMA*, $p = 0.3028$ (ns) for *GZMK*, $p = 0.6761$ (ns) for *GZMM*. (Unpaired t test with Welch's correction). (**C**) Fold changes in intracellular perforin, granzyme B and granzyme A protein expressions were measured by flow cytometry in NK-92 cells with L598 (30 μM) or vehicle control for 3 days. Results are from n = two biological replicates; each tested in two technical replicates.

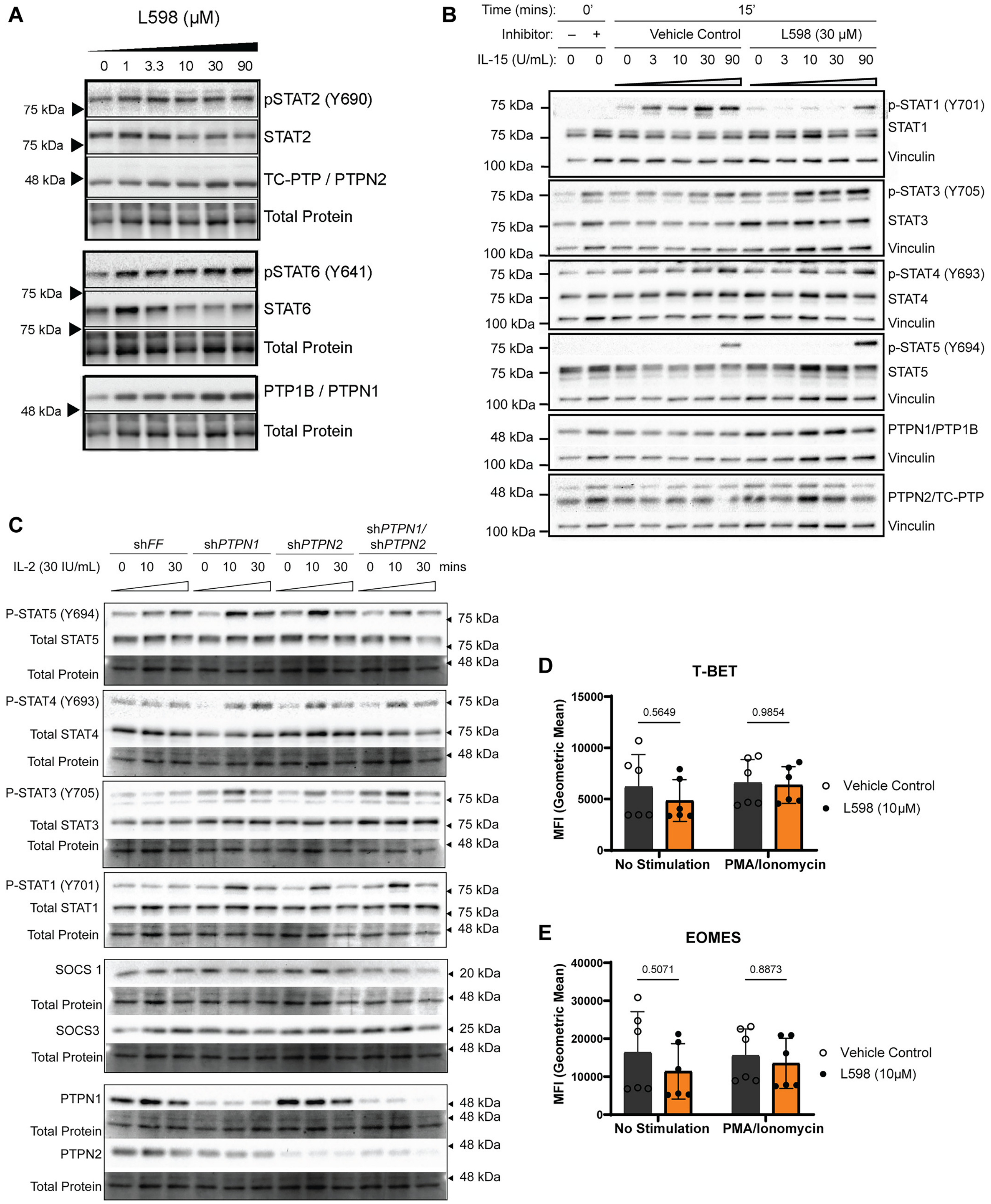
A
L598 (µM)
0 1 3.3 10 30 90
75 kDa
pSTAT2 (Y690)
75 kDa
STAT2
48 kDa
TC-PTP / PTPN2
Total Protein
pSTAT6 (Y641)
75 kDa
STAT6
75 kDa
Total Protein
PTP1B / PTPN1
48 kDa
Total Protein
B
Time (mins): 0' 15'
Inhibitor: – + Vehicle Control L598 (30 µM)
IL-15 (U/mL): 0 0 0 3 10 30 90 0 3 10 30 90
75 kDa p-STAT1 (Y701)
75 kDa STAT1
100 kDa Vinculin
75 kDa p-STAT3 (Y705)
75 kDa STAT3
100 kDa Vinculin
75 kDa p-STAT4 (Y693)
75 kDa STAT4
100 kDa Vinculin
75 kDa p-STAT5 (Y694)
75 kDa STAT5
100 kDa Vinculin
48 kDa PTPN1/PTP1B
100 kDa Vinculin
48 kDa PTPN2/TC-PTP
100 kDa Vinculin
C
shFF shPTPN1 shPTPN2 shPTPN1/ shPTPN2
IL-2 (30 IU/mL) 0 10 30 0 10 30 0 10 30 0 10 30 mins
P-STAT5 (Y694) 75 kDa
Total STAT5 75 kDa
Total Protein 48 kDa
P-STAT4 (Y693) 75 kDa
Total STAT4 75 kDa
Total Protein 48 kDa
P-STAT3 (Y705) 75 kDa
Total STAT3 75 kDa
Total Protein 48 kDa
P-STAT1 (Y701) 75 kDa
Total STAT1 75 kDa
Total Protein 48 kDa
SOCS 1 20 kDa
Total Protein 48 kDa
SOCS3 25 kDa
Total Protein 48 kDa
PTPN1 48 kDa
Total Protein 48 kDa
PTPN2 48 kDa
Total Protein 48 kDa
D
T-BET
MFI (Geometric Mean)
15000
10000
5000
0
0.5649
0.9854
No Stimulation
PMA/Ionomycin
Vehicle Control
L598 (10µM)
E
EOMES
MFI (Geometric Mean)
40000
30000
20000
10000
0
0.5071
0.8873
No Stimulation
PMA/Ionomycin
Vehicle Control
L598 (10µM)

 © The Author(s)

Figure EV4. Dual targeting of PTPN1 and PTPN2 modulates STAT signaling downstream of IL-2 or IL-15 in NK-92 cells.

(**A**) Western blot analysis of STAT2 (Y690) and STAT6 (Y641) phosphorylation in NK-92 cells treated with L598 (0–90 µM) and cultured in the presence of IL-2 (100 IU/mL) for 3 days. A representative figure from n = two biological replicates is shown. Total protein (~ 48 kDa) is used as the loading control. (p)-STAT6 (> 75 kDa) and Granzyme B (~ 35 kDa) shown in Fig. 2I, were detected on the same membrane; accordingly, the same total protein loading control is presented for both. Likewise, the same membrane was initially probed for (p)-STAT1 (Fig. 4A) and PTPN1 (Fig. EV4A), then stripped and re-probed for (p)-STAT2 and PTPN2 (both shown in Fig. EV4A); therefore, the same loading control is shown for all panels. (**B**) Western blot analysis of phosphorylated STAT signaling in NK-92 cells treated with dual inhibitor L598 (30 µM) or vehicle control, following 15-minute stimulation with increasing doses of IL-15 (0, 3, 10, 30, 90 IU/mL). A representative figure from n = two biological replicates is shown. (p)-STAT3 (> 75 kDa) and PTPN1 (> 48 kDa) were detected on the same membrane; accordingly, the same vinculin loading control is presented for both. Likewise, the same membrane was blotted for (p)-STAT5 (> 75 kDa) and PTPN2 (< 48 kDa) and the same vinculin loading control is presented for both. (**C**) Western blot analysis of phosphorylated STAT signaling in shRNA knockdown NK-92 cells stimulated with IL-2 (30 IU/mL) for 10 or 30 min. A representative figure from n = two biological replicates is shown. The same membrane was used to detect (p)-STAT1 (> 75 kDa) and SOCS1 (~ 20 kDa); therefore, the same total protein loading control is shown. Likewise, (p)-STAT4 (> 75 kDa) and PTPN1 (> 48 kDa) were detected on the same membrane, and (p)-STAT5 (> 75 kDa), PTPN2 (< 48 kDa), and SOCS3 (~ 25 kDa) were detected on the same membrane; therefore, the same total protein loading controls are shown for each group. (**D**, **E**) Flow cytometry analysis of transcription factors TBET and EOMES in NK-92 cells treated with L598 (10 µM) or vehicle control, under PMA/ionomycin stimulation or unstimulated conditions. N = two biological replicates, each tested in three technical replicates are shown. Data are presented as mean ± SD. P-values: $p = 0.5649$ (ns) for no stimulation; $p = 0.9854$ (ns) for PMA/ionomycin stimulation (Two-way ANOVA with Šídák's test).

© The Author(s)

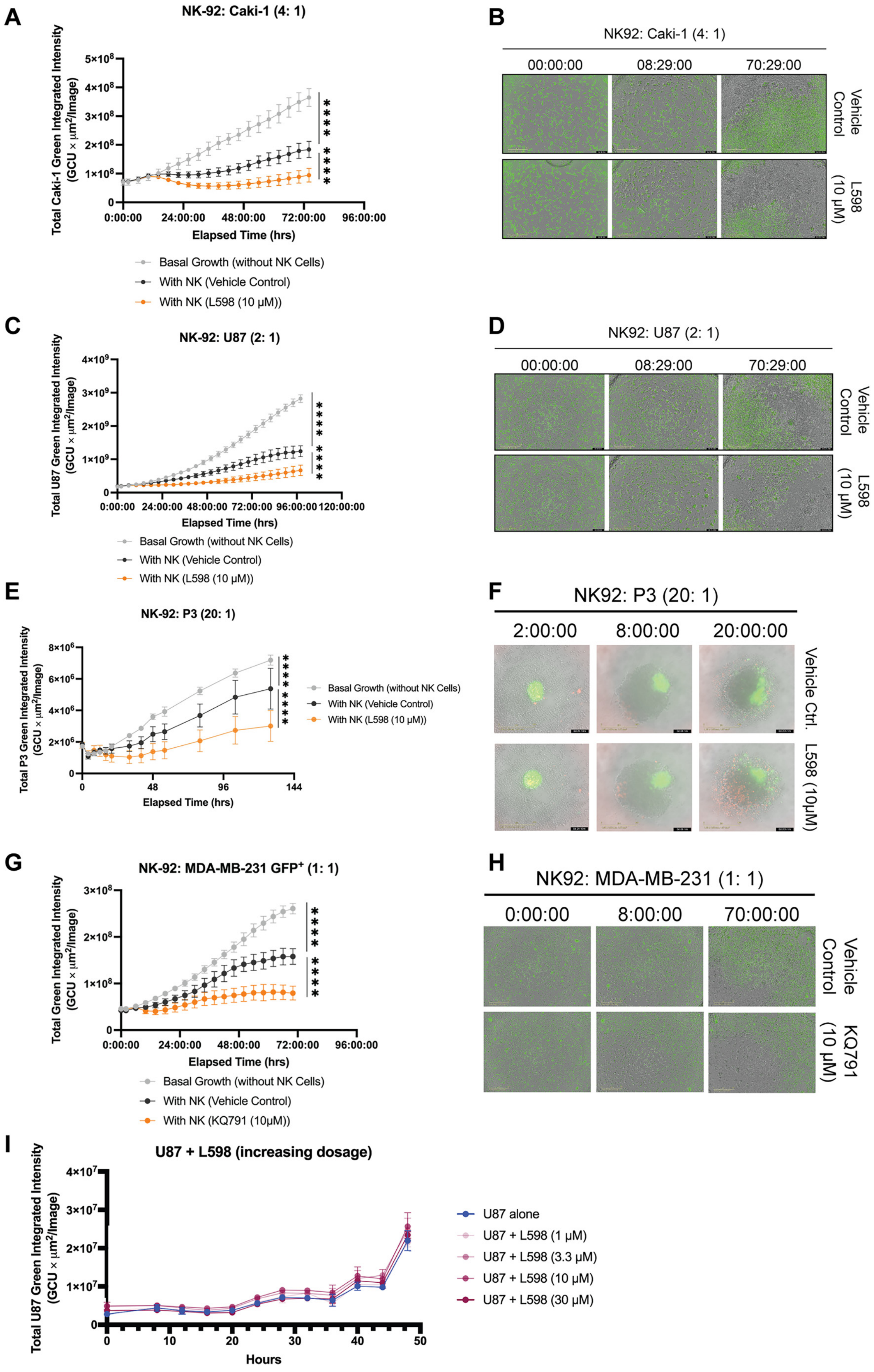
A
NK-92: Caki-1 (4: 1)
Total Caki-1 Green Integrated Intensity (GCU × µm²/Image)
5×10⁸
4×10⁸
3×10⁸
2×10⁸
1×10⁸
0
0:00:00 24:00:00 48:00:00 72:00:00 96:00:00
Elapsed Time (hrs)
****
****
Basal Growth (without NK Cells)
With NK (Vehicle Control)
With NK (L598 (10 µM))
B
NK92: Caki-1 (4: 1)
00:00:00 08:29:00 70:29:00
Vehicle Control
L598 (10 µM)
C
NK-92: U87 (2: 1)
Total U87 Green Integrated Intensity (GCU × µm²/Image)
4×10⁹
3×10⁹
2×10⁹
1×10⁹
0
0:00:00 24:00:00 48:00:00 72:00:00 96:00:00 120:00:00
Elapsed Time (hrs)
****
****
Basal Growth (without NK Cells)
With NK (Vehicle Control)
With NK (L598 (10 µM))
D
NK92: U87 (2: 1)
00:00:00 08:29:00 70:29:00
Vehicle Control
L598 (10 µM)
E
NK-92: P3 (20: 1)
Total P3 Green Integrated Intensity (GCU × µm²/Image)
8×10⁶
6×10⁶
4×10⁶
2×10⁶
0
0 48 96 144
Elapsed Time (hrs)
****
****
Basal Growth (without NK Cells)
With NK (Vehicle Control)
With NK (L598 (10 µM))
F
NK92: P3 (20: 1)
2:00:00 8:00:00 20:00:00
Vehicle Ctrl.
L598 (10µM)
G
NK-92: MDA-MB-231 GFP⁺ (1: 1)
Total Green Integrated Intensity (GCU × µm²/Image)
3×10⁸
2×10⁸
1×10⁸
0
0:00:00 24:00:00 48:00:00 72:00:00 96:00:00
Elapsed Time (hrs)
****
****
Basal Growth (without NK Cells)
With NK (Vehicle Control)
With NK (KQ791 (10µM))
H
NK92: MDA-MB-231 (1: 1)
0:00:00 8:00:00 70:00:00
Vehicle Control
KQ791 (10 µM)
I
U87 + L598 (increasing dosage)
Total U87 Green Integrated Intensity (GCU × µm²/Image)
4×10⁷
3×10⁷
2×10⁷
1×10⁷
0
0 10 20 30 40 50
Hours
U87 alone
U87 + L598 (1 µM)
U87 + L598 (3.3 µM)
U87 + L598 (10 µM)
U87 + L598 (30 µM)

 © The Author(s)

Figure EV5. Dual inhibition of PTPN1/PTPN2 enhances NK cell-mediated cytolysis of solid tumor cell lines in vitro.

(**A–D**) IncuCyte-based real-time cytolysis assay of NK-92 cells treated with L598 (10 μM) or vehicle control, co-cultured with eGFP⁺ Caki-1 kidney cancer cells (E:T = 4:1) in (**A**, **B**) or eGFP⁺ U87 glioblastoma cells (E:T = 2:1) in (**C**, **D**) over 3 days. Green fluorescence quantification (tumor cell viability) is shown in (**A**) and (**C**); representative merged images of bright-field and green fluorescence channels are shown in (**B**) and (**D**). Experiments were performed in n = three biological replicates, each tested in six technical replicates. Data are presented as mean ± SD. *P*-values: $p \leq 0.0001$ (****) (Two-way ANOVA with Tukey's test). (**E**, **F**) IncuCyte-based cytolysis assay of NK-92 cells treated with L598 (10 μM) or vehicle control against eGFP⁺ P3 tumor cells (E:T = 20:1) over 3 days. Quantification of green fluorescence is shown in (**E**). Representative live-cell images combining bright-field, green (eGFP), and red fluorescence (cleaved caspase-3/7 substrate) are shown in (**F**). Experiments were performed in *n* = three biological replicates, each tested in six technical replicates. Data are presented as mean ± SD. *P*-values: $p \leq 0.0001$ (****) (Two-way ANOVA with Tukey's test). (**G**, **H**) IncuCyte-based cytolysis assay of NK-92 cells pre-treated with KQ791 (10 μM) or vehicle control, co-cultured with eGFP⁺ MDA-MB-231 cells (E:T = 1:1) for 3 days. Quantification of green fluorescence is shown in (**G**), and representative images of merged bright-field and green fluorescence are shown in (**H**). Experiments were performed in *n* = three biological replicates, each tested in six technical replicates. Data are presented as mean ± SD. *P*-values: $p \leq 0.0001$ (****) (Two-way ANOVA with Tukey's test). (**I**) Tumor growth assay of eGFP⁺ U87 cells treated with increasing concentrations of L598 (1–30 μM). Experiments were performed in *n* = three biological replicates; each tested in two technical replicates. Data are presented as mean ± SD.

© The Author(s)
